# Adiposity and cancer: systematic review and meta-analysis

**DOI:** 10.1038/s42255-026-01542-8

**Published:** 2026-06-15

**Authors:** Eleanor L. Watts, Amparo Gonzalez-Feliciano, Marc J. Gunter, Nilanjan Chatterjee, Steven C. Moore

**Affiliations:** 1 Division of Cancer Epidemiology and Genetics, National Cancer Institute, Shady Grove, MD USA; 2 Cancer Epidemiology and Prevention Research Unit, School of Public Health, Imperial College London, London, UK; 3 Department of Biostatistics, Bloomberg School of Public Health, Johns Hopkins University, Baltimore, MD USA; 4 Department of Oncology, School of Medicine, Johns Hopkins University, Baltimore, MD USA

**Keywords:** Risk factors, Cancer, Metabolism, Metabolic diseases, Obesity

## Abstract

Obesity is a major health challenge. Here we show that body mass index is positively associated with risk of 19 cancers and inversely associated with 3, based on a systematic review and meta-analysis of prospective studies of 25 cancer types. We searched PubMed, EMBASE and Scopus through to 23 April 2025, identifying 226 articles comprising 1.5 million incident cancers. We identified positive associations for leukaemia, non-Hodgkin lymphoma, bladder cancer and glioma, not previously identified by major consensus reports. Associations differed according to region and sex, with stronger associations for postmenopausal breast and ovarian cancers in East Asia, weaker associations for gallbladder cancer in East Asia and in men, and stronger associations for colorectal cancer in men. Body mass index and waist circumference showed similar associations with cancer. We reviewed Mendelian randomization and imaging-based studies; genetic findings were generally consistent with observational associations, while imaging data were limited. Our findings underscore the impact of obesity on cancer risk.

## Abstract (web summary)

In a meta-analysis including 1.5 million incidences of cancer, positive associations between body mass index and 19 cancers were identified, underlining the impact of obesity on cancer risk.

## Main

Global economic development, urbanization and changes in diet and physical activity have driven a worldwide rise in body mass index (BMI). Once limited to high-income countries, obesity (BMI ≥ 30 kg m^−^^2^) is now a global challenge^1^, with low- and middle-income countries accounting for 75% of the world’s adult population with obesity, including China, which ranks second only to the United States in the number of individuals with obesity^2^. Among the health consequences of rising BMI is an increased risk of cancer^3^, and this may contribute to the rapidly growing cancer burden in low- and middle-income countries^4^.

Between 2016 and 2018, landmark reviews by the World Cancer Research Fund (WCRF)^3^, the International Agency for Research on Cancer (IARC)^5^ and others^6^ established that an elevated BMI is associated with increased risk of at least 13 types of cancer. While these reviews marked a major advance in our understanding of obesity and cancer, key questions remain. Chief among these questions is whether, and to what extent, these associations generalize across global regions. To our knowledge, only Renehan et al. systematically addressed this question^7^, but their analysis was limited by sparse data outside Europe and North America. If obesity–cancer associations differ according to region, this could substantially affect global estimates of cancer burden attributable to obesity and deepen—or complicate—our understanding of the underlying mechanisms. Other key uncertainties include whether BMI is associated with less common or smoking-related cancers, the extent of heterogeneity according to sex and whether risk estimates differ when alternative measures of adiposity, such as waist circumference, are used.

Since these reviews, major new epidemiological data have emerged from large, population-wide administrative healthcare databases, such as those from South Korea, and next-generation cohorts with deep phenotypic and genotypic data, including the UK Biobank (UKB). These resources offer new opportunities to characterize obesity–cancer associations across diverse global populations and to explore new avenues of enquiry, such as the use of Mendelian randomization (MR) to strengthen causal inference^8^. Yet despite these advances, BMI–cancer associations have not been comprehensively reassessed through a systematic review and meta-analysis. A precise, up-to-date understanding of obesity’s impact on cancer risk is especially critical in the current era of glucagon-like peptide-1 receptor agonists and other incretin-based therapies, as healthcare systems weigh their high costs against potential public health benefits.

We aimed to examine the current global epidemiological landscape of BMI and cancer by conducting a systematic review and meta-analysis of the prospective associations across 25 common cancer types. We compared our findings with previous WCRF reviews and with results from our meta-analyses of MR studies. We also explored the regional distribution of epidemiological studies to date, assessed whether associations vary according to region and sex, compared associations for BMI with those for waist circumference and summarized emerging evidence from imaging-derived adiposity measures.

## Results

### Characteristics of the studies included

**Extended Data Fig. 1:**
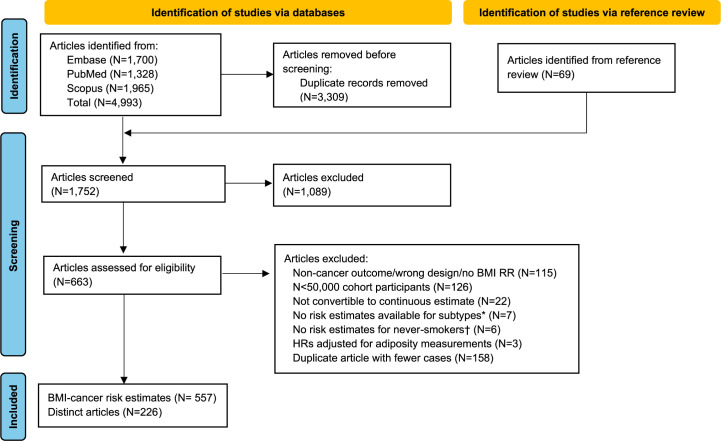
Studies identified by search strategy in observational studies. Numbers reflect the number of distinct articles. Some cancer-specific risk estimates may be excluded if they did not meet the inclusion criteria for individual cancer types (for example, an article may contribute risk estimates for one cancer type and be included in the total, but its estimates for another cancer may be excluded if superseded by a study based on the same cohort with more cases). Further details on the search strategy and exclusion criteria are provided in the Supplementary Methods. *Subtypes: breast cancer (pre and postmenopausal), CNS (glioma and meningioma), gastric (non-cardia and cardia), oesophageal (SCC and adenocarcinoma). † For smoking-related cancers only (lung, head and neck, bladder, oesophageal SCC). Abbreviations: CNS=central nervous system, HR=hazard ratio, PMID=PubMed ID.

We identified 4,993 potentially eligible articles through our database search and 69 additional potentially eligible articles from reference review. We excluded 3,309 duplicate articles, 1,089 ineligible articles based on title and abstract screening, and 279 after full-text review. An additional 158 articles were excluded because they were superseded by a more recent publication from the same population with larger case numbers. In total, 226 articles met our inclusion criteria, yielding 557 BMI–cancer associations (some studies reported on multiple cancer types) (Extended Data Fig. 1).

**Fig. 1:**
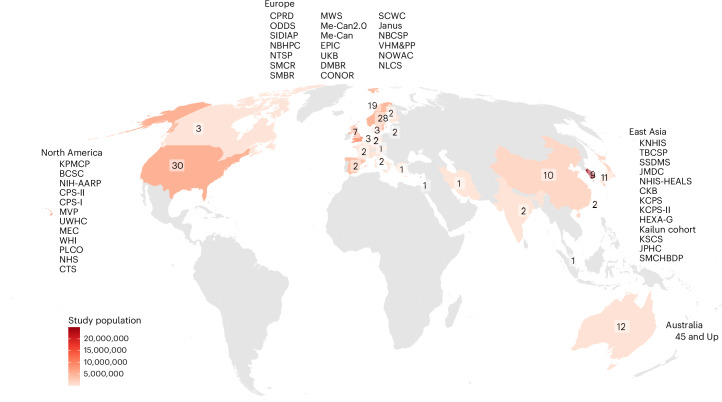
World map of the cohorts included. The numbers show the count of prospective cohorts with cancer data in each country or territory included in this meta-analysis. Study population reflects the total number of participants across cohorts within each country or territory. For cohorts reported in multiple publications with varying analytical sample sizes, the largest sample size was used. Text labels highlight prospective studies with over 100,000 participants in each region. Multi-country cohorts and pooling projects (for example, EPIC, Adventist Health Study 2) have been disaggregated according to country or territory based on participant numbers at each study centre. Where possible, pooling projects consisting of several smaller cohorts were further separated into their constituent cohorts. Cohorts in India report cancer mortality only. 45 and Up, the Sax Institute’s 45 and Up Study; BCSC, Breast Cancer Surveillance Consortium; CKB, China Kadoorie Biobank; CONOR, Cohort of Norway; CPRD, UK Clinical Practice Research Datalink; CPS, Cancer Prevention Study; CTS, California Teachers Study; DMBR, Danish Medical Birth Registry; EPIC, European Prospective Study into Cancer and Nutrition; HEXA, Health Examinees; Janus, Janus Serum Bank Cohort; JMDC, JMDC Claims Database; JPHC, Japan Public Health Center; KCPS, Korean Cancer Prevention Study; KNHIS, National Health Insurance Service of Korea; KPMCP, Kaiser Permanente Medical Care Program; KSCS, Kangbuk Samsung Cohort Study; MEC, Multiethnic Cohort Study; Me-Can, Metabolic syndrome and Cancer project; MVP, Million Veteran Program; MWS, Million Women Study; NBCSP, Norwegian Breast Cancer Screening Program; NBHPC, Norwegian BMI/Height Prospective Cohort 1963–2001; NHIS-HEALS, National Health Insurance Service-National Health Screening Cohort; NHS, Nurses’ Health Study; NIH-AARP, National Institutes of Health-AARP; NLCS, Netherlands Cohort Study; NOWAC, Norwegian Women and Cancer Study; NTSP, Norwegian Tuberculosis Screening Program; ODDS, Obesity and Disease Development Sweden; PLCO, Prostate, Lung, Colorectal and Ovarian Cancer Screening Trial; SCWC, Swedish Construction Workers Cohort; SIDIAP, Information System for Research in Primary Care; SMCHBDP, Shandong Multi-Center Healthcare Big Data Platform; SMBR, Swedish Medical Birth Register; SMCR, Swedish Military Conscription Register; SSDMS, Shanghai Standardized Diabetes Management System; TBCSP, Taiwanese Breast Cancer-Screening Program; UKB, UK Biobank; UWHC, University of Wisconsin Hospital and Clinics Electronic Health Record Study; VHM&PP, Vorarlberg Health Monitoring and Prevention Programme; WHI, Women’s Health Initiative.

The included cohorts originated from 23 countries and six major geographical regions (Fig. 1). For three countries—Australia, Lithuania and Israel—we included prospective cohorts with fewer than 50,000 participants to improve representation from their respective geographical regions. There were no studies of cancer incidence originating from Africa, South or Central America, Eastern Europe, South or Central Asia, the Caribbean or the Pacific Islands (excluding Hawaii) (Fig. 1). The countries included represent only 33% of the global population^9^, and include none of the global regions where obesity is most prevalent, particularly the Pacific Islands, the Arabian Peninsula and neighbouring Gulf states^2^.

**Fig. 2:**
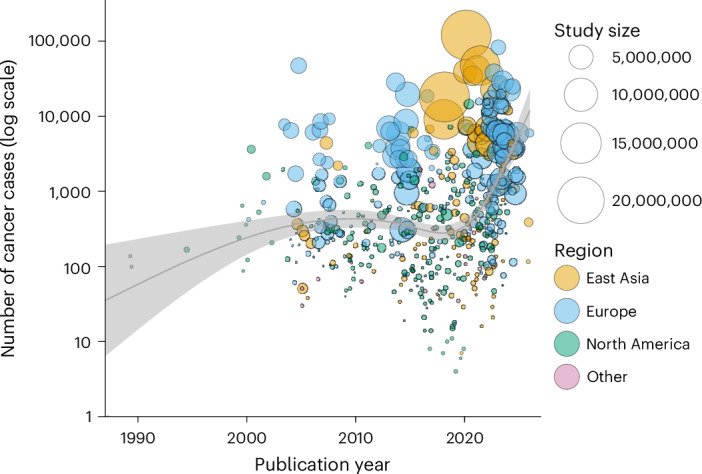
Prospective cohort cancer studies according to the number of incident cancer cases and publication year. Bubble size shows the number of participants for each BMI–cancer study. The trend line shows the average number of cancer cases according to publication year (modelled by spline); the grey band shows the 95% CIs.

In aggregate, this review includes data on 1,520,512 incident cancers across the 25 types of cancer, with the largest number of cases for colorectal cancer (*n* = 406,486) and the fewest for oesophageal squamous cell cancer (SCC) (*n* = 988; never-smokers only). This represents at least a twofold increase in the number of cases for each cancer type, and up to 27 times more for certain types, compared with the WCRF reports (Extended Data Table 1). Participant numbers increased in parallel; for example, the colorectal cancer analysis included 44.3 million participants, compared with 4.8 million in the prior WCRF review. This exponential increase since 2015 (Fig. 2) largely reflects the inclusion of large administrative healthcare database studies, such as those based on the National Health Insurance Service of Korea. We also included six additional cancer types not covered by the WCRF reports (thyroid, meningioma, multiple myeloma, leukaemia, non-Hodgkin lymphoma (NHL), bladder (never-smokers) and glioma).

**Fig. 3:**
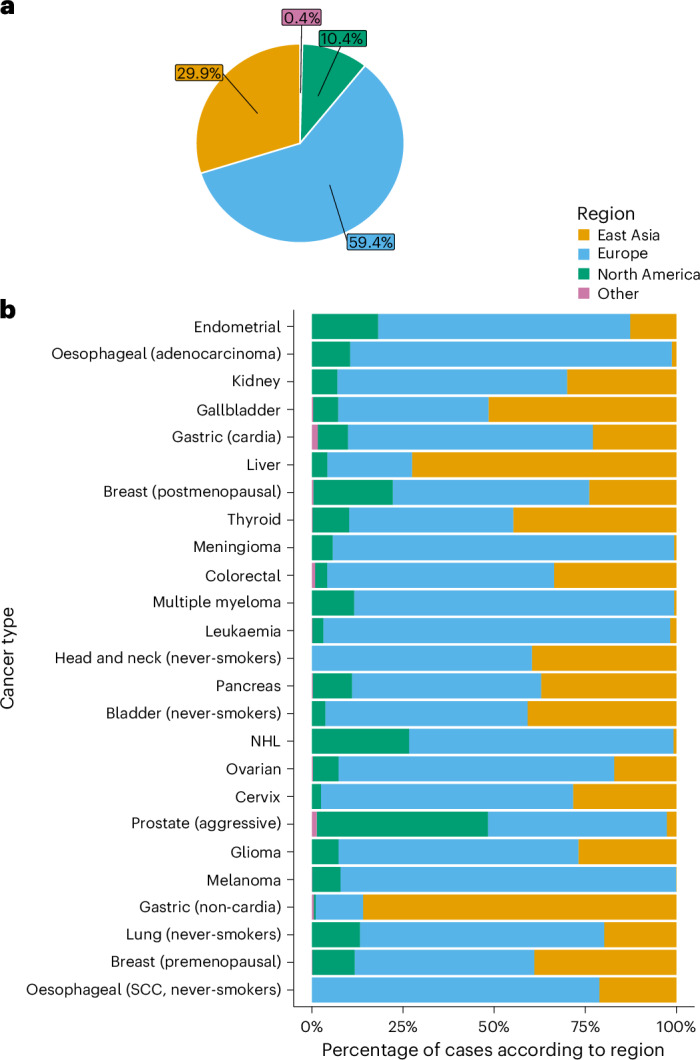
Percentage of cancer cases originating from each geographical region. **a**, Sum of the 25 types of incident cancers. **b**, Individual cancers. Estimates from pooled studies spanning multiple regions for which country-specific case numbers were not available were excluded from this figure (head and neck). Numbers may not sum because of rounding.

Cancer cases predominantly originated from Europe (59.4%), followed by East Asia (29.9%) and North America (10.4%). The remainder were from Australia, India, Iran and Israel (Fig. 3a). Notably, geographical distribution varied according to cancer type; for example, no large studies on oesophageal SCC in never-smokers were available from North America, and for many cancers, no data were available outside Europe, East Asia and North America (Fig. 3b).

**Extended Data Fig. 2:**
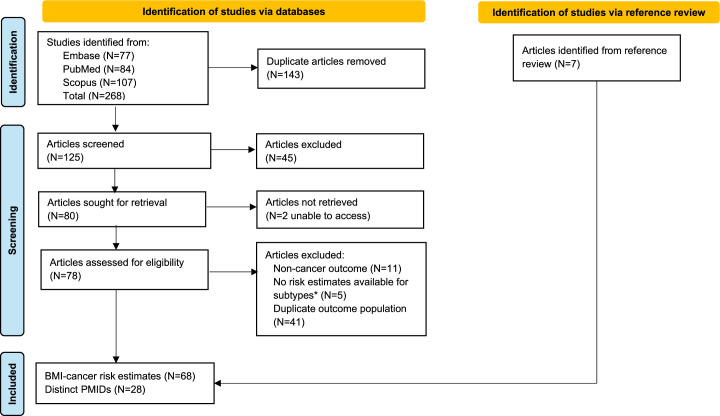
Flow diagram of search strategy and study selection for Mendelian randomisation studies. Further details on the search strategy and exclusion criteria are provided in the Supplementary Methods. *Subtypes: breast cancer (pre and postmenopausal), CNS (glioma and meningioma), gastric (non-cardia and cardia), oesophageal (SQ and adenocarcinoma). Abbreviations: CNS=central nervous system, PMID=PubMed ID, SQ=squamous cell carcinoma.

For the MR meta-analysis, our search identified 268 articles, with an additional seven articles identified from the reference review. We excluded 143 duplicate articles, 47 ineligible articles based on title and abstract screening, and 57 after full-text review. In total, 28 MR studies met our inclusion criteria, contributing 68 BMI–cancer risk estimates (Extended Data Fig. 2).

Across these risk estimates, 85% were based on genetic instruments for BMI derived from European ancestry populations, primarily using data from the Genetic Investigation of ANthropometric Traits consortium ± UKB, encompassing up to 806,834 individuals^10^. The remaining studies used instruments derived from East Asian ancestry populations, with sample sizes up to 236,117 individuals^11^. These MR studies included outcome genome-wide association studies that together included 383,897 cancer cases, of which 355,043 (92%) were from populations with a European ancestry, 28,577 (7%) from East Asian populations and 277 from a Chilean population^12^.

Details for each study, including model adjustments, number of cases, number of the analytical population, risk estimates, follow-up duration and originally reported units are available from the Supplementary Data.

### BMI–cancer associations

**Fig. 4:**
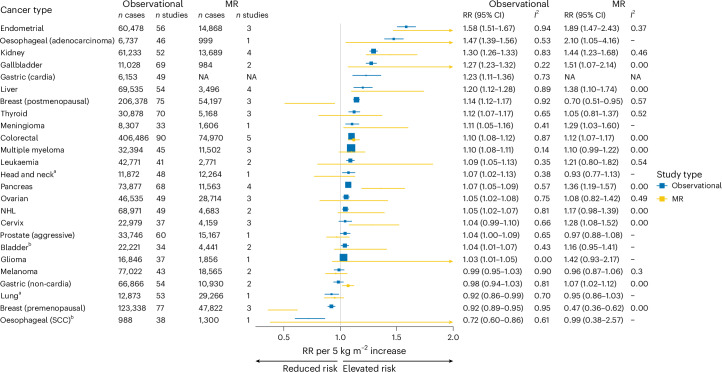
Associations between BMI with cancer risk, RR per 5 kg m^−^^2^ increase in BMI. RRs per 5 kg m^−^^2^ increase in BMI are represented by squares, with the error bars showing 95% CIs. Observational risk estimates were calculated using random-effects meta-analysis. Heterogeneity between studies is quantified using *I*^2^; an *I*^2^ value close to 100% indicates substantial heterogeneity but can be affected by the number of studies and the precision of individual study estimates. All statistical tests were two-sided. Further details of model adjustments, follow-up time and analytical population for each study are available from the Supplementary Data. ^a^Observational estimates are based on never-smokers; MR analyses were adjusted for genetically predicted smoking. ^b^Observational estimates are based on never-smokers; smoking-adjusted MR analyses were not available, so univariable MR results are shown. NA, not applicable.

Higher BMI was associated with varying magnitudes of increased cancer risk, with nearly a 20-fold difference in risks across cancer types. At the upper end, a 5 kg m^−^^2^ increase in BMI was associated with a 58% increased risk of endometrial cancer (95% confidence interval (CI) 1.51–1.67) and a 47% higher risk of oesophageal adenocarcinoma (1.39–1.56); at the other end, the same increment in BMI was associated with a 3% increased risk of glioma (1.01–1.05). BMI was also positively associated with risk of kidney (1.30, 1.26–1.33), gallbladder (1.27, 1.23–1.32), gastric (cardia) (1.23, 1.11–1.36), liver (1.20, 1.12–1.28), breast (postmenopausal) (1.14, 1.12–1.17), thyroid (1.12, 1.07–1.17), meningioma (1.11, 1.05–1.16), colorectal (1.10, 1.08–1.12), multiple myeloma (1.10, 1.08–1.11), leukaemia (1.09, 1.05–1.13), pancreatic (1.07, 1.05–1.09), ovarian (1.05, 1.02–1.08), NHL (1.05, 1.02–1.07) and prostate (aggressive) (1.04, 1.00–1.09) cancer. BMI was inversely associated with premenopausal breast cancer (0.92, 0.89–0.95) (Fig. 4). For cancers where smoking is the dominant risk factor, associations among never-smokers were positive for head and neck (1.07, 1.02–1.13) and bladder (1.04, 1.01–1.07) cancer, and inverse for oesophageal SCC (0.72, 0.60–0.86) and lung cancer (0.92, 0.86–0.99). The estimated false discovery rate across the 25 cancers was 5%, indicating a low likelihood that the findings occurred by chance.

For each cancer type, RRs were broadly consistent in direction across studies, although with some quantitative heterogeneity (that is, *I*^2^ > 0%; Supplementary Figs. 1–25). Heterogeneity was greatest for endometrial cancer, breast cancer (premenopausal and postmenopausal) and melanoma (*I*^2^ ≥ 90%). Despite the high *I*^2^ for endometrial and breast cancers, associations were directionally consistent and statistically significant in most large studies; for example, the interquartile range (IQR) of study-specific RRs for endometrial cancer was 1.47–1.75. In contrast, for melanoma, some studies reported weak inverse associations and others weak positive associations (IQR = 0.95–1.03).

For most cancer types, there was little evidence of publication bias. Among cancers with more than ten studies^13^, Egger’s intercept was statistically significant (*P* < 0.05) for colorectal, premenopausal breast and endometrial cancers, but the differences between the trim-and-fill RRs and the uncorrected RRs were small (maximum 7% change) (Supplementary Fig. 26). Overall, study quality was high (mean total score = 1.87 of 2, s.d. = 0.34). Most studies adequately adjusted for confounders (mean confounding control score = 0.97, s.d. = 0.17) and had follow-up durations longer than 5 years (mean follow-up score = 0.90, s.d. = 0.30). These findings suggest that reverse causation or confounding is unlikely to explain the observed associations. Scores according to cancer type are provided in Extended Data Table 2 and for each study in the Supplementary Data.

In the sensitivity analyses, restricting to cohorts that adjusted for smoking or were at low risk of bias had minimal effect on associations, with changes in RRs of less than 5% across all cancer types (Extended Data Table 3). Excluding administrative healthcare database studies substantially reduced the number of cases but yielded similar RRs for most cancers. A notable exception was oesophageal SCC, for which the association became more strongly inverse in analyses restricted to administrative healthcare databases (relative risk (RR) per 5 kg m^−^^2^ = 0.61, 95% CI 0.44–0.85) compared with traditional cohorts alone (RR = 0.75; Table 1).

**Table 1:** Associations of BMI with cancer risk according to study design compared with the primary analysis

	Traditional cohorts				Administrative healthcare study^a^				
Cancer type	*n* cases	*n* cohorts	RR per 5 kg m^−^^2^ (95 CI %)	*P*	*n* cases	*n* cohorts	RR per 5 kg m^−^^2^ (95 CI %)	*P*	ΔRR
Endometrial	18,812	25	1.60 (1.51–1.71)	2 × 10^−49^	41,666	31	1.53 (1.43–1.64)	1 × 10^−36^	0.07
Oesophageal (adenocarcinoma)	3,159	16	1.46 (1.33–1.61)	3 × 10^−15^	3,578	30	1.48 (1.37–1.61)	1 × 10^−20^	−0.02
Kidney	8,447	21	1.31 (1.27–1.34)	1 × 10^−84^	52,786	31	1.28 (1.20–1.37)	1 × 10^−14^	0.03
Gallbladder	2,594	39	1.24 (1.18–1.31)	3 × 10^−16^	8,434	30	1.29 (1.22–1.37)	1 × 10^−16^	−0.05
Gastric (cardia)	2,601	19	1.26 (1.11–1.43)	0.0004	3,552	30	1.17 (0.98–1.40)	0.08	0.09
Liver	7,207	23	1.21 (1.11–1.32)	2 × 10^−5^	62,328	31	1.18 (1.07–1.29)	0.0007	0.03
Breast (postmenopausal)	91,010	46	1.15 (1.13–1.18)	2 × 10^−29^	115,368	29	1.10 (1.03–1.19)	0.008	0.05
Thyroid	14,182	38	1.12 (1.06–1.18)	6 × 10^−5^	16,696	32	1.12 (1.07–1.17)	1 × 10^−6^	0
Meningioma	1,253	6	1.14 (1.05–1.24)	0.002	7,054	27	1.08 (1.02–1.14)	0.005	0.06
Colorectal	69,823	58	1.11 (1.09–1.14)	7 × 10^−19^	336,663	32	1.07 (1.04–1.10)	3 × 10^−7^	0.04
Multiple myeloma	4,957	12	1.08 (1.05–1.12)	6 × 10^−7^	27,437	33	1.10 (1.07–1.12)	2 × 10^−16^	−0.02
Leukaemia	5,264	9	1.09 (1.02–1.17)	0.01	37,507	32	1.09 (1.06–1.11)	2 × 10^−13^	0
Head and neck (never-smokers)	1,108	19	1.13 (1.05–1.23)	0.001	10,764	29	1.04 (0.98–1.10)	0.25	0.09
Pancreas	18,052	38	1.09 (1.06–1.11)	1 × 10^−11^	55,825	30	1.05 (1.01–1.09)	0.02	0.04
Ovarian	9,267	18	1.05 (1.02–1.09)	0.0004	37,268	31	1.05 (1.00–1.10)	0.07	0
Bladder (never-smokers)	1,610	5	1.04 (0.98–1.11)	0.21	20,611	29	1.05 (1.00–1.10)	0.07	−0.01
NHL	25,198	17	1.05 (1.01–1.09)	0.02	43,773	32	1.05 (1.00–1.09)	0.04	0
Cervix	1,624	6	1.03 (0.91–1.16)	0.66	21,355	31	1.04 (0.99–1.10)	0.14	−0.01
Prostate (aggressive)	19,964	33	1.04 (1.00–1.08)	0.08	13,782	27	1.16 (0.85–1.58)	0.34	−0.12
Glioma	2,420	9	1.02 (1.00–1.04)	0.045	14,426	28	1.08 (0.96–1.22)	0.21	−0.06
Melanoma	17,921	13	0.99 (0.95–1.03)	0.66	59,101	30	1.05 (0.86–1.28)	0.65	−0.06
Gastric (non-cardia)	16,449	24	0.98 (0.93–1.04)	0.52	50,417	30	0.99 (0.93–1.05)	0.74	−0.01
Lung (never-smokers)	5,329	25	0.93 (0.87–1.00)	0.07	7,544	28	0.89 (0.76–1.06)	0.19	0.04
Breast (premenopausal)	39,475	47	0.92 (0.89–0.96)	8 × 10^−5^	83,863	30	0.91 (0.85–0.98)	0.01	0.01
Oesophageal (SCC, never-smokers)	874	12	0.75 (0.61–0.93)	0.008	114	26	0.61 (0.44–0.85)	0.003	0.14

^a^Defined as cohorts including routinely collected health data generated for purposes other than epidemiological research. Some consortia studies included both traditional prospective cohorts and administrative healthcare studies but did not report results separately; in such cases, we classified the risk estimates as originating from administrative healthcare studies, reflecting the larger number of participants from registry-based cohorts, particularly the ODDS consortium^86^. ODDS, Obesity and Disease Development Sweden.

Risk estimates were broadly consistent with those from the previous WCRF review, although more precise (median absolute difference in RR = 0.03; Extended Data Table 4). The largest difference observed was for oesophageal SCC (Δ = 0.13), which may partially reflect our study’s substantially greater precision in estimating the risk estimate for this cancer, which is rare in never-smokers.

In the MR analysis, results were generally consistent with those from the observational analysis (Fig. 4). Of the 22 observational associations, nine showed statistically significant and directionally consistent associations in the MR analyses: endometrial (odds ratio per 5 kg m^−^^2^ = 1.89, 95% CI 1.47–2.43), oesophageal adenocarcinoma (2.10, 1.05–4.16), kidney (1.44, 1.23–1.68), gallbladder (1.51, 1.07–2.14), liver (1.38, 1.10–1.74), meningioma (1.29, 1.03–1.60), colorectal (1.12, 1.07–1.17) and pancreatic (1.36, 1.19–1.57) cancers, as well as an inverse association for premenopausal breast cancer (0.47, 0.36–0.62). For thyroid cancer, multiple myeloma, leukaemia, ovarian cancer, NHL and glioma, the MR estimates were generally similar or slightly larger than the observational estimates; however, reduced precision meant that these results were not statistically significant. The larger effect sizes are consistent with MR potentially reflecting the impact of lifelong exposure to elevated BMI. Notably, in contrast to the observational findings, the MR analyses reported an inverse association with postmenopausal breast cancer (0.70, 0.51–0.95) and a positive association with gastric non-cardia cancer (1.07, 1.02–1.12).

For smoking-related cancers, the multivariable MR estimates that accounted for genetically predicted smoking were only available for head and neck cancer and lung cancer. For head and neck cancer, the overall summary univariable MR estimate per 5 kg m^−^^2^ increase in BMI was 1.29 (0.69–2.40). In the single study that additionally adjusted for genetically predicted smoking, the risk estimate attenuated to 0.93 (0.77–1.13). Similarly, for lung cancer, the summary univariable estimate was 1.38 (1.17–1.63) but in the single study incorporating genetically predicted smoking, the estimate attenuated to 0.95 (0.86-1.03). For bladder cancer and oesophageal SCC only univariable MR estimates were available. The risk estimate for bladder cancer was 1.16 (0.95–1.41), directionally consistent with the observational association, while the estimate for oesophageal SCC was null, although imprecise (0.99, 0.38–2.57). Further study details and risk estimates are available in the Supplementary Data.

Based on our tailored certainty assessment, we had high certainty for 11 cancer types: endometrial, oesophageal (adenocarcinoma), kidney, gallbladder, gastric (cardia), liver, breast (postmenopausal), thyroid, meningioma, colorectal and multiple myeloma. There was moderate certainty for leukaemia and pancreatic and ovarian cancers, and NHL, and for the inverse associations with breast (premenopausal), lung and oesophageal (SCC) cancer. There was low certainty for head and neck cancer, prostate cancer (aggressive), bladder cancer (never-smokers) and glioma (Extended Data Table 5).

### Evaluation of regional heterogeneity

BMI–cancer associations were generally consistent across regions, particularly between Europe and North America (median absolute difference in RR = 0.02). Larger differences were observed between East Asia and Western regions, for example, the median difference between East Asia and Europe was 0.12. Comparatively few cases were available from other regions, precluding the analysis of obesity–cancer associations for much of the rest of the world, underscoring the need for far more data to capture the full breadth of BMI–cancer relationships globally.

**Fig. 5:**
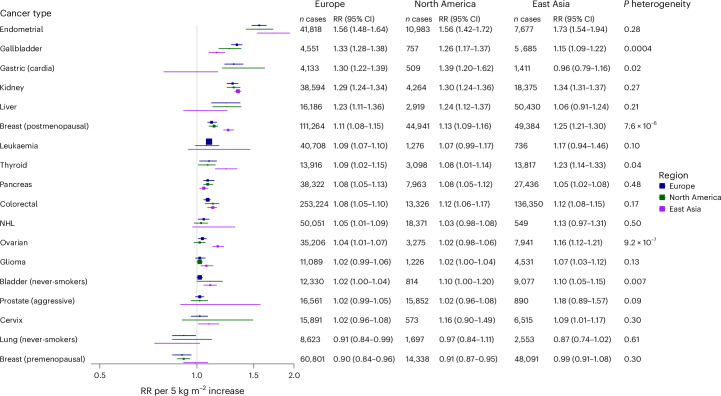
Associations between BMI and cancer risk according to region. This figure is restricted to cancer types with more than 500 cases per region and estimates available for Europe, North America and East Asia. Risk estimates were calculated using random-effects meta-analysis. Points represent RRs per 5 kg m^−^^2^ increase in BMI; the error bars show the 95% CIs. Between-group heterogeneity was assessed using the Cochran’s *Q* statistic, with corresponding *P* values reported in the figure. Analyses include Australia, South Asia and West Asia (not shown because of the low number of cancers). All statistical tests were two-sided; no adjustment for multiple comparisons was applied. Degrees of freedom were defined as d.f. = *k* − 1, where *k* is the number of regions included for each cancer type (see Supplementary Figs. 27–51 for the full results).

For several cancer types, we observed evidence of heterogeneity according to region, with particularly striking differences for postmenopausal breast cancer (Fig. 5). The RR per 5 kg m^−^^2^ increment was 1.13 in North America (95% CI 1.09–1.16) and 1.11 in Europe (1.08–1.15) but substantially higher—1.25—in East Asia (1.21–1.30), approximately double the excess risk. A similar pattern was observed for ovarian cancer, with stronger associations in East Asia (1.16, 1.12–1.21) than in North America (1.02, 0.98-1.06) or Europe (1.04, 1.01–1.07). Associations for thyroid cancer were also stronger in East Asia (1.23, 1.14–1.33) compared with North America (1.08, 1.01–1.14) and Europe (1.09, 1.02–1.15). For gallbladder cancer, the pattern was reversed, with a weaker association in East Asia (1.15, 1.09–1.22) compared with Europe (1.33, 1.28–1.38) and North America (1.26, 1.17–1.37). For gastric cardia, associations were null in East Asia (0.96, 0.79–1.16) and positive in Europe (1.30, 1.22–1.39) and North America (1.39, 1.20–1.62). For bladder cancer, the RR was weaker in Europe (1.02, 1.00–1.04) compared with East Asia and North America (RR = 1.10 in both regions).

Associations according to cancer, region and study are available in Supplementary Figs. 27–51.

### Evaluation of heterogeneity according to sex

Of the 391 BMI–cancer risk estimates for non-reproductive cancer types included in the primary analysis, 184 sex-specific estimates were available for men and 232 for women. We further excluded head and neck cancer and oesophageal SCC because of insufficient case numbers (<100 per sex), leaving 180 estimates for men including 331,712 cancer cases, and 223 estimates for women, including 354,877 cases (Supplementary Data).

**Fig. 6:**
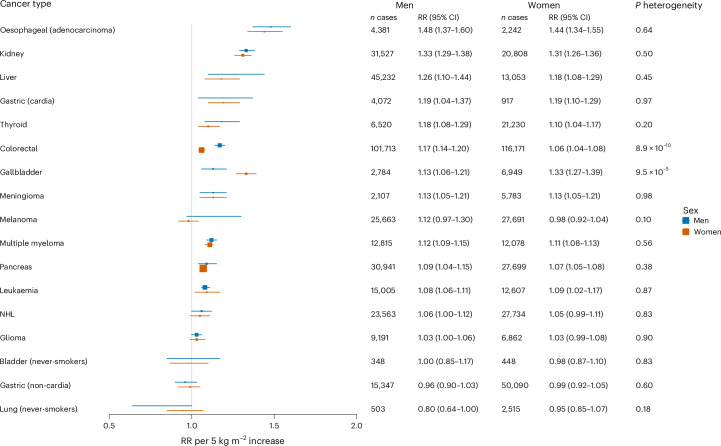
Associations between BMI and cancer risk according to sex. Risk estimates were calculated using random-effects meta-analysis. Points represent the RR per 5 kg m^−^^2^ increase in BMI; the error bars show the 95% CIs. Between-group heterogeneity was assessed using Cochran’s *Q* statistic (d.f. = 1; *P* values reported in the figure). All tests were two-sided and no adjustment for multiple comparisons was applied. Full results for each cancer type, study and sex are shown in Supplementary Figs. 52–68.

Overall, BMI–cancer associations were broadly consistent according to sex (median absolute difference in RR = 0.02). However, evidence of sex-specific heterogeneity was observed for two cancer types. Risk associations with colorectal cancer were substantially greater in men (RR per 5 kg m^−^^2^ = 1.17, 95% CI 1.14–1.20) than in women (1.06, 1.04–1.08), while associations with gallbladder cancer were greater in women (1.33, 1.27–1.39) than in men (1.13, 1.06–1.21) (Fig. 6).

Associations according to cancer, sex and study are shown in Supplementary Figs. 52–68.

### Waist circumference versus BMI and cancer risk

**Fig. 7:**
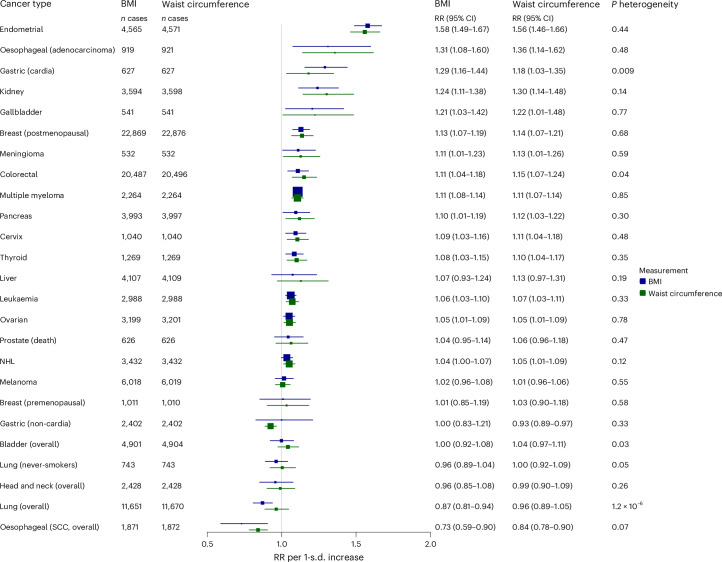
Associations of BMI, waist circumference and cancer risk per 1-s.d. increase. Associations were estimated using a random-effects meta-analysis. Points represent the RR per 1-s.d. increase in BMI and waist circumference; the error bars show the 95% CIs. Heterogeneity in associations according to adiposity measure was assessed using the Wald test (d.f. = 1), with corresponding *P* values reported in the figure. All tests were two-sided; no adjustment for multiple testing was applied. Full results for each cancer type, study and adiposity measure are shown in Supplementary Figs. 69–94.

We identified eight studies including up to 2.2 million participants and 122,000 cases across five countries (China, Spain, Sweden, UK and USA)^14–21^. Overall, differences in risk estimates between adiposity measures were small (median absolute difference in RR = 0.02). However, for several smoking-related cancers, we observed evidence of heterogeneity when analyses were not restricted to never-smokers, particularly for lung cancer (RR per 1-s.d. increase: BMI 0.87 (0.81–0.94) versus waist circumference 0.96 (0.89–1.05)); bladder cancer (1.00 (0.92–1.08) versus 1.04 (0.97–1.11)); and oesophageal SCC (0.73 (0.59–0.90) versus 0.84 (0.78–0.90)). For lung cancer, this heterogeneity was attenuated when analyses were restricted to never-smokers. However, similar data were not available for other smoking-related cancers. Statistically significant heterogeneity between adiposity measures was also observed for gastric cardia (BMI 1.29 (1.16–1.44) versus waist circumference 1.18 (1.03–1.35)) and colorectal cancer (1.11 (1.04–1.18) versus 1.15 (1.07–1.24)) (Fig. 7). Risk associations for each study and type of cancer are shown in Supplementary Figs. 69–94.

### Adiposity imaging studies

**Extended Data Fig. 3:**
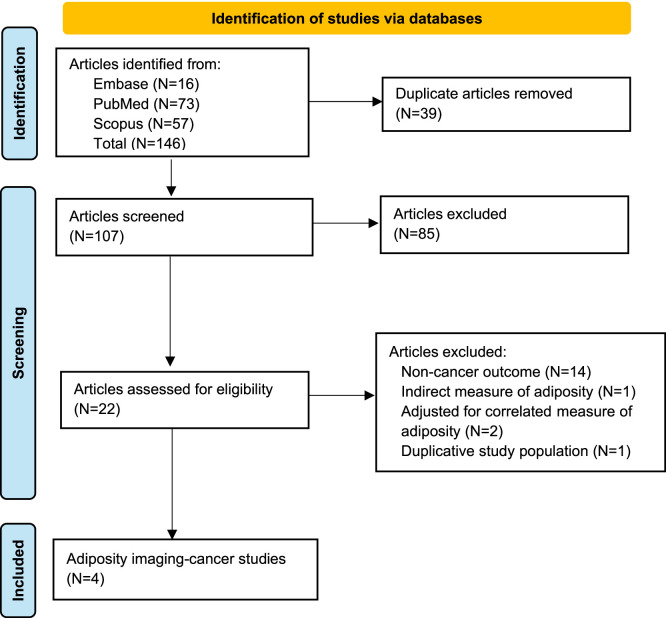
Flow diagram of search strategy and study selection for adiposity imaging studies. Further details on the search strategy and exclusion criteria are provided in the Supplementary Methods.

Our database search identified 107 non-duplicate articles, of which 85 were excluded during title and abstract screening and 18 after full-text review, leaving four articles (Extended Data Fig. 3).

Two studies from the Women’s Health Initiative evaluated the association between adiposity measured using dual-energy X-ray absorptiometry (DEXA) and cancer risk. In one study, which included 639 incident breast cancer cases, higher whole-body fat percentage (RR per 1 s.d. = 1.16, 95% CI 1.07–1.26) and trunk fat mass (1.21, 1.12–1.31) were associated with increased risk, similar to the effect for BMI (1.19, 1.10–1.28)^22^. In contrast, a separate study of 169 colorectal cancer cases found no statistically significant associations between BMI, DEXA-derived measures and colorectal cancer risk^23^. In the AGES-Reykjavik study, which used computed tomography imaging, one analysis examined prostate cancer risk, although statistical power was limited (43 aggressive prostate cancers)^24^. A more recent UKB study reported that higher intrapancreatic fat, measured using magnetic resonance imaging, was associated with increased pancreatic cancer risk (hazard ratio per one quintile increase = 1.55, 1.25–1.92). This association persisted after further adjustment for BMI and central obesity (1.41, 1.10–1.82)^25^. However, the study did not directly compare the magnitude of these associations with those for BMI. Risk estimates are available in Extended Data Table 6.

## Discussion

This comprehensive systematic review and meta-analysis of 226 prospective cohort studies, including 1.5 million incident cancers across 25 common types, found that higher BMI was associated with increased risk of 19 cancer types and inversely associated with three. Our findings substantially expand on prior reviews and offer four key insights: (1) obesity may be associated with a broader range of cancers than previously recognized; (2) while some associations are consistent across regions, risk estimates for breast and ovarian cancers may be higher in East Asia, whereas those for gallbladder cancer may be weaker; (3) BMI has stronger associations with colorectal cancer in men and weaker associations with gallbladder cancer compared with women; and (4) BMI and waist circumference show similar associations with cancer risk.

### BMI–cancer associations

Our primary finding, that BMI was positively associated with the risk of 19 cancer types, suggests that adiposity may be a risk factor for most cancers, emphasizing the importance of obesity prevention as a public health priority. By leveraging up to 27-fold more cases than earlier reports, we identified associations for leukaemia, NHL, bladder cancer and glioma, which had not been previously reported in IARC or WCRF reports. For other cancers, our findings largely reaffirm associations previously reported in major consensus reports. Notably, despite the substantially larger sample size, risk estimates for these established associations remained consistent. This stability suggests that BMI–cancer associations may have reached a point of convergence for many cancer types. Further data collection may yield limited additional insight unless it focuses on specific deficiencies, particularly the lack of prospective cohorts from most nations of the world. Presenting all the evidence together using uniform methods enables systematic comparison of BMI–cancer associations, which vary nearly 20-fold across cancer types, suggesting multiple underlying mechanisms with different tissues and organs exhibiting different levels of susceptibility. For oesophageal adenocarcinoma, the hypothesized mechanisms are mechanical, with excess weight compressing the stomach or relaxing the gastroesophageal sphincter, increasing the risk of gastro-oesophageal reflux^26^. For kidney, gallbladder, liver and pancreatic cancers, the proposed mechanisms involve metabolic and local inflammatory conditions, that is, hypertension, gallstones, non-alcoholic fatty liver disease and diabetes, respectively. For female reproductive cancers, obesity increases concentrations of circulating oestrogens through aromatization of testosterone and androstenedione in adipose tissue, with known carcinogenic effects in breast and endometrial tissues^27,28^. For other cancers, associations may be mediated by systemic inflammation, hyperinsulinaemia or other indirect downstream effects^29^. Differences in the magnitude of BMI–cancer associations across cancer types probably also arise in part from other strong, type-specific risk factors, for example, the dominant role of *Helicobacter pylori* in non-cardia gastric cancer^30^. In the coming years, application of omics technologies within human studies coupled with advanced experimental models are expected to enhance understanding of biological mechanisms linking adiposity and cancer^29^.

A long-standing observation is the inverse association between BMI and premenopausal breast cancer risk. Although recognized since the 1990s^31^, it has continued to perplex researchers. Recent evidence suggests that the timing of adiposity, rather than menopausal status per se, is critical, with higher childhood BMI associated with a reduced risk of both premenopausal and postmenopausal breast cancer^32^. This may help explain why genetically predicted BMI was associated with lower risk of both premenopausal and postmenopausal breast cancer in our study: genetically predicted BMI more closely reflects childhood body size through to adolescence than adult BMI, whereas observational associations are probably influenced by weight gain in later life^33^.

In contrast, associations between BMI and smoking-related cancers are inconsistent and difficult to disentangle from bias. Since the 1970s, studies have reported that a higher BMI is generally associated with a lower risk of smoking-related cancers^34–36^, particularly lung cancer^37,38^, although interpretation remains contentious. Smoking is the dominant risk factor for these malignancies, for example, current smokers have a more than tenfold higher risk of lung cancer compared with never-smokers^39^, and is generally associated with a lower BMI^40^, raising concerns on the potential for residual confounding^41^. Consequently, some studies suggest a protective effect of adiposity^42–45^, while others attribute these associations to incomplete adjustment for smoking^14,41,46–50^. To minimize residual confounding, we restricted analyses of smoking-related cancers to never-smokers. In this group, a higher BMI was associated with increased risks of bladder and head and neck cancers, a lower risk of lung cancer, and a markedly lower risk of oesophageal SCC. These findings suggest that residual confounding may explain earlier inverse associations previously observed for bladder and head and neck cancers, and may substantially contribute to those for lung cancer, but is unlikely to account for the strong inverse relationship observed for oesophageal SCC. A critical caveat is that lung cancer in never-smokers may represent a biologically distinct disease, characterized by histological and somatic mutation profiles that differ from those found in smokers^51–54^. Consequently, differences in the BMI–lung cancer association according to smoking status may reflect a real difference in effect, rather than merely the elimination of residual confounding^55,56^. Our MR analysis provides some reassurance, as the multivariable MR results, which accounted for smoking, aligned with our observational risk estimate for never-smokers. However, a previous multivariable MR study also reported histology-specific associations between BMI and lung cancer risk^57^; future studies into subtype-specific mechanisms will be essential for disentangling potential causal pathways.

### Evaluation of regional heterogeneity

It is important to recognize that scientific disciplines differ in how they approach heterogeneity, both in its assigned importance and in how they interpret its implications. In epidemiology, examining heterogeneity is considered fundamental. It can reveal potential cofactors underlying aetiological relationships^58^ and strengthen internal validity by demonstrating that findings are robust across varying confounding structures^8^. Regional analyses are particularly informative because they permit exploration of aetiological relationships across a full spectrum of human lifestyles and environments.

In our analysis, we observed substantial regional heterogeneity in BMI–cancer associations for female reproductive cancers (breast and ovarian cancer) and gallbladder cancer. From a public health perspective, the high degree of heterogeneity for the obesity–breast cancer association is particularly important. Current estimates of obesity-attributable breast cancer cases in East Asia are based on RRs from Western populations; our results suggest that these estimates may understate the true number of cases due to obesity by a factor of two or more, emphasizing the need to use region-specific estimates in future studies.

Regional variation in reproductive cancer associations may reflect differences in background risk factors. For instance, postmenopausal hormone therapy is less common in East Asia^59,60^ and BMI is more strongly associated with breast cancer risk among non-hormone-users^61^. Additionally, postmenopausal oestrogen levels may be lower in East Asian women^62–64^, which could amplify the relative impact of obesity on a women’s overall oestrogenic exposure. Variation in ovarian cancer may similarly relate to hormone therapy and oral contraceptive use, although interpretation is challenging given marked heterogeneity according to histological subtype^65,66^. Weaker BMI associations with gallbladder cancer in East Asia may reflect differences in gallstone aetiology. In Western populations, obesity is associated with cholesterol-rich gallstones, while in East Asia, brown pigment stones are more common than in Western populations and are associated with chronic infections and biliary stasis^67^. More broadly, regional differences may arise from variation in the prevalence of major risk factors (for example, hepatitis B and C virus or *H. pylori*) or in the distribution of tumour subtypes, which may have distinct aetiological pathways. Differences in RRs could also reflect variation in surveillance practices or confounding structures. For instance, if confounding is less pronounced in East Asian populations, stronger associations may more closely approximate the true effect of obesity on cancer risk, suggesting that earlier studies may have underestimated these relationships. Differences in life-course adiposity patterns may also contribute to heterogeneity.

Another potential explanation for variation in associations among East Asians is differences in body fat distribution, as individuals of East Asian ancestry tend to have higher body fat and waist circumference for a given BMI compared with those of European ancestry^68,69^. However, this is unlikely to explain the pattern of heterogeneity observed, as associations were both stronger and weaker across cancer types rather than showing a consistent geographical trend. Lower average BMI in East Asian populations^70^ could also contribute if BMI–cancer associations were non-linear (for example, U-shaped or J-shaped). However, unlike for mortality, the relationships between BMI and cancer are typically approximately linear^19,71,72^; therefore, the RR per unit BMI should remain consistent across populations.

### Evaluation of sex heterogeneity

Heterogeneity in BMI–cancer associations according to sex may reflect differences in sex steroid hormone levels, lifestyle factors such as smoking, alcohol consumption and physical activity, and health-seeking behaviours. Adiposity also varies according to sex; although men have lower total adiposity and greater lean mass for a given BMI, they store a higher proportion of fat as visceral adipose tissue, which may be more metabolically harmful than subcutaneous fat^73^.

We observed stronger associations between BMI and gallbladder cancer in women. Women have a higher prevalence of gallstones than men, probably because of oestrogen-stimulated biliary cholesterol secretion and subsequent cholesterol supersaturation of bile^74^. In this context, oestradiol may act as a synergistic effect modifier, leading to larger effects in women than men, thereby increasing the risk of gallstones independent of BMI. However, further research is needed to clarify the nature of these relationships. We also observed stronger associations between BMI and colorectal cancer in men than in women, a finding that has been consistently reported in observational studies^7,75^. Interpretation is complicated by MR studies, which report an opposing pattern, with positive associations between genetically predicted BMI and colorectal cancer in women but null associations in men^76^. Several mechanisms have been proposed to explain these sex differences, particularly effect modification by oestradiol and inflammatory pathways, although findings across studies remain inconsistent^75,77–79^.

### Waist circumference versus BMI and cancer risk

BMI is often critiqued for its inability to differentiate fat mass from lean muscle, and for not accounting for adiposity types, particularly visceral adipose tissue, an especially metabolically harmful fat depot stored in the abdominal cavity. Waist circumference has been proposed as a more informative alternative metric for investigating associations with cancer risk. However, findings across studies have been mixed; while some have reported stronger associations for waist circumference^20,37,79–81^, others have found little difference between the two measures^14,15,19,82,83^. In our analysis, we similarly observed minimal differences in associations with cancer risk, suggesting that substituting BMI for waist circumference, or vice versa, would yield broadly comparable conclusions. This probably reflects the high correlation between BMI and waist circumference at the population level (*r* ≈ 0.9), as well as evidence that both measures are more strongly associated with subcutaneous adipose tissue (*r* = 0.88 for BMI and 0.84 for waist circumference) than with visceral adipose tissue (0.77 and 0.79, respectively), and are moderately correlated with lean mass (0.75 and 0.58, respectively)^84^. These complexities make it challenging to disentangle their independent effects and highlight the limited specificity of waist circumference as a proxy for visceral adipose tissue. Nonetheless, we observed some modest differences between the two measures, particularly for smoking-related cancers, which may relate to smokers generally having lower BMI but higher waist circumference, greater body fat and reduced muscle mass^37,85^. Possibly, smoking may be less of a confounder for waist circumference.

Imaging data suggest that fat depots have weaker correlations with BMI and waist circumference^82^; therefore, it may be possible to disentangle independent effects. However, existing imaging studies are limited in statistical power. The UKB’s planned collection of DEXA and magnetic resonance imaging scans for 100,000 participants may enhance the ability to assess fat-depot-specific cancer risks and clarify the role of adipose tissue distribution in cancer development.

### Strengths, limitations and conclusions

This systematic review and meta-analysis has several key strengths. The large sample size enabled robust evaluation across a broad range of cancers, including several not previously assessed in WCRF reports or with limited prior evidence. We conducted detailed analyses of smoking-related cancers among never-smokers to minimize residual confounding. Most included studies were high quality, with long follow-up and appropriate adjustment for confounders. To strengthen causal inference, we triangulated observational findings with MR evidence^8^. Newly available data from East Asia also allowed assessment of geographical variation in risk estimates. We systematically compared risk estimates for waist circumference and BMI and incorporated emerging imaging-based measures of adiposity. Finally, to support transparency and future updates, we have made the formatted data publicly available. An important limitation is that, despite a comprehensive review, many world regions were not represented because of lack of prospective cancer studies, including Africa, South and Central America, Eastern Europe, South and Central Asia, the Caribbean and the Pacific Islands. Additionally, most genetic data were derived from populations with European ancestry. Expanding genetic data collection in populations with non-European ancestry is an essential next step towards evaluating ancestry-specific genetic instruments for BMI and assessing the generalizability of causal inferences. Aside from large administrative healthcare databases, most cohorts include individuals who are healthier and wealthier than the underlying population and lack adequate representation of minority groups, potentially limiting the generalizability to lower socioeconomic and underrepresented populations. Our analysis focused on the 25 most common cancers; however, many rarer cancers and subtypes are also probably associated with BMI^86^. Given the stability of many risk estimates and the availability of large prospective datasets, future research should prioritize understudied populations, and less common cancers and subtypes, while ensuring robust adjustment for confounding. Owing to the large volume of cancer–BMI literature, we applied more restrictive search terms than are conventionally applied; this may have reduced sensitivity. We also limited inclusion to studies with 50,000 or more participants in East Asia, Northern and Western Europe, and North America, thereby excluding some smaller cohorts; however, given the substantial participant numbers, this is unlikely to materially affect risk estimates.

In summary, BMI is positively associated with 19 common cancers and inversely associated with three. Notably, we identified positive associations for leukaemia, NHL, bladder and glioma cancers, as well as inverse associations for lung and oesophageal SCC, which were not previously reported in WCRF or IARC reviews. We also observed regional differences in associations, particularly for breast and ovarian cancers; however, many populations remain entirely unrepresented in obesity–cancer risk research. Collectively, these findings highlight the substantial and growing impact of obesity on cancer risk and the urgent need to address this modifiable risk factor worldwide.

## Methods

This systematic review adhered to the standard criteria for reporting meta-analyses as outlined in the PRISMA guidelines^87^. Institutional review board approval was not required because this study did not involve human participant research.

### BMI and cancer risk

Our meta-analysis includes prospective cohort studies that examined the association of BMI with the incidence of the 25 most common cancers according to global incidence. A systematic search was conducted using the PubMed, EMBASE and Scopus databases for articles published from database inception through to 23 April 2025. The search terms used are detailed in Supplementary Table 1. To verify completeness, we also searched the reference lists of recent studies. Additional details on cancer selection and the search strategy are provided in the Supplementary Methods.

We used Covidence for the screening process. We first deduplicated articles from the different databases. We then screened the titles and abstracts of articles for eligibility, excluded ineligible articles, reviewed the full text of the remainder and performed final eligibility exclusions. Screening and review were conducted independently by two authors (E.L.W. and S.C.M.); a third author (A.G.-F.) broke the ties.

Eligible studies met the following criteria: (1) prospective risk estimates of adult (≥18 years) BMI–cancer associations; (2) 50,000 or more participants (unless the cohort was from an underrepresented geographical region, that is, outside North America, North, West and Central Europe, and East and Southeast Asia); (3) continuous risk estimates or three or more categories of BMI; (4) provided estimates for subtype-specific cancers when pertinent (that is, aggressive prostate cancer, gastric (cardia and non-cardia), breast (premenopausal and postmenopausal), oesophageal (adenocarcinoma and SCC)); (5) risk estimates for never-smokers (only for head and neck, lung, oesophageal SCC and bladder cancers); (6) models did not adjust for early-life BMI or other measures of adiposity; (7) non-duplicative population (when duplicate populations were identified, we used the risk estimate based on the greatest number of cases).

We extracted risk estimates and study information, including hazard ratios, odds ratios, RRs and their corresponding 95% CIs for the association between BMI and cancer risk. We prioritized estimates derived from continuous modelling of BMI and those most comprehensively adjusted for confounders, provided no adjustment for other adiposity measures was made (for example, waist circumference, BMI from an earlier age). Risk estimates were converted to the 5 kg m^−^^2^ scale to enable comparison across studies. We also extracted sample size (cases and analytical population), cohort, country, number of studies, duration of follow-up and model adjustments. Quality for each study and cancer type was assessed using a two-point grading system: (1) adjustment for confounders relevant for that type of cancer; and (2) a minimum follow-up period of 5 years to minimize the risk of reverse causation (Supplementary Table 2).

To understand how the landscape of cancer epidemiology has evolved, we extracted data from the WCRF reports and compared the risk estimates, number of countries and sample size to each cancer type in our analysis. We focused on comparing with the WCRF reports because, unlike narrative or umbrella reviews, they conducted de novo meta-analyses using primary data, making them more directly comparable in terms of methods and outputs. To examine geographical patterns in risk estimates, we grouped cohorts according to country and region, summarizing findings both overall and according to cancer type. We also evaluated trends in study size over time across geographical regions using a bubble plot, with trends modelled using natural cubic splines with three degrees of freedom.

Summary risk estimates for the association between BMI and each cancer type were estimated using a random-effects meta-analysis, which weights studies based on within-study and between-study variability and produces a weighted average of individual study effect sizes. Between-study heterogeneity was assessed using *I*^2^, tau^2^ (estimated using the Paule–Mandel method^88^) and the IQR (25th and 75th percentiles) of RRs for each cancer type. Publication bias was evaluated using Egger’s test and the trim-and-fill method^89^. Meta-analyses were conducted using log-transformed effect estimates; we assume that effect-size estimates follow a normal distribution assuming a large sample size for individual studies.

In the sensitivity analyses, we examined the impact of excluding risk estimates from studies that (1) did not adjust for smoking, (2) were based on large administrative healthcare databases and (3) received the maximum quality assessment score (2) (Supplementary Methods).

We also meta-analysed studies that investigated the associations of BMI with cancer using MR approaches and compared risk estimates with the observational findings. Our systematic search was conducted in PubMed, EMBASE and Scopus, covering articles published from database inception through 6 February 2025. Eligible studies met the following criteria: (1) MR-based risk estimates of BMI–cancer associations; (2) estimates for subtype-specific cancers (where needed); (3) risk estimate based on a linear estimate; and (4) a non-duplicative study population (in the outcome population only). Univariable MR risk estimates based on the primary analytical model were extracted, generally based on the inverse-variance-weighted method, standardized to the 5 kg m^−^^2^ scale for comparability across studies, and meta-analysed. Heterogeneity between studies was assessed using *I*^2^. For smoking-related cancers, we also extracted multivariable MR estimates adjusted for genetically predicted smoking, where available, to account for the effect of smoking on risk estimates.

### Assessment of certainty

We applied a tailored certainty assessment adapted from GRADE^90^, emphasizing the domains most relevant to observational epidemiology: magnitude of effect, confounding or reverse causation, consistency, publication bias, and concordance with MR evidence. Certainty was classified as high when all criteria were met: a large effect (RR > abs (10%)); robustness to bias (associations remained consistent when restricted to studies with the lowest risk of bias); consistency across studies (25th and 75th percentiles of study-specific RRs aligned directionally with the summary estimate); robustness to publication bias (trim-and-fill analyses yielded estimates consistent with the summary); and MR findings directionally consistent with the summary estimate. Moderate certainty was assigned when the effect size was more than 5% and all other criteria were met, while low certainty was assigned to associations that were nominally significant (*P* < 0.05) but did not satisfy the thresholds for high or moderate certainty.

### Evaluation of heterogeneity according to region and sex

To assess heterogeneity in BMI–cancer associations according to geographical region, we categorized each risk estimate in the primary analysis according to the following regions—Australia, East Asia, South Asia, the Middle East, Europe and North America—based on the study’s country. Sex-specific heterogeneity was evaluated by extracting male-specific and female-specific risk estimates for each cancer type included in the primary analysis. Sex was defined by individual studies and was based on self-report or medical records. Between-group heterogeneity was assessed using Cochran’s *Q* test.

### Comparison of BMI and waist circumference

To systematically compare how BMI and waist circumference relate to cancer risk, we selected from our meta-analysis only studies that met the following criteria: (1) reported results for both BMI and waist circumference using the same models and populations, to eliminate random and systematic variability that could arise from using different studies for each exposure; (2) investigated five or more cancer types, to minimize publication bias as broader analyses are less prone to selective reporting (for example, reporting only cancers where one measure ‘outperforms’ the other)^91,92^; (3) did not mutually adjust for waist circumference or vice versa as mutual adjustment can distort associations between body composition measures and cancer risk^93^; and (4) reported results per s.d. or in continuous units convertible to s.d. to ensure comparable estimates on the same scale.

These criteria ensured that comparisons between BMI and waist circumference were as methodologically consistent and unbiased as possible. We also included risk estimates for smoking-related cancers without restriction to never-smokers. This is because smokers generally have greater abdominal fat relative to their BMI^37,85,94^, and thus any differences between BMI and waist circumference in relation to disease outcomes may be most evident among current smokers^37,95,96^. Heterogeneity in the risk estimates according to BMI and waist circumference were assessed using the Wald test.

### Adiposity imaging and cancer

We also evaluated associations between imaging-based adiposity and the risk of the top 25 cancer types. Our literature search was conducted in PubMed, EMBASE and Scopus through to 6 February 2025.

Eligible studies met the following criteria: (1) prospective risk estimates of adiposity imaging and cancer; (2) estimates for subtype-specific cancers, where relevant; (3) risk estimates for never-smokers, where relevant; (4) risk estimates based on participants who underwent imaging (studies using predictive models of adiposity based on a subset or an independent population were excluded); (5) no adjustment for a correlated adiposity measure; and (6) non-duplicative study populations (see the Supplementary Methods for details).

### Statistics and reproducibility

No statistical methods were used to predetermine sample size because this meta-analysis relied on sample sizes from previously published studies. No data were excluded from the analyses unless they did not meet prespecified selection criteria. The analyses were not randomized and investigators were not blinded to allocation or outcome assessment.

### Reporting summary

Further information on research design is available in the Nature Portfolio Reporting Summary linked to this article.

## Supplementary information

Supplementary Information Supplementary Methods, Tables 1 and 2 and Figs. 1–94.

Reporting Summary

Supplementary Data Supplementary Data

PRISMA checklist

## Figures and Tables

**Extended Data Table 1:**
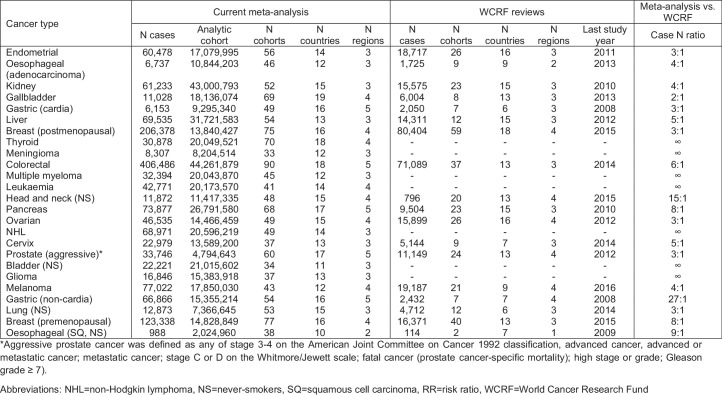
Comparison of sample sizes between the current meta-analysis and the WCRF review

**Extended Data Table 2:**
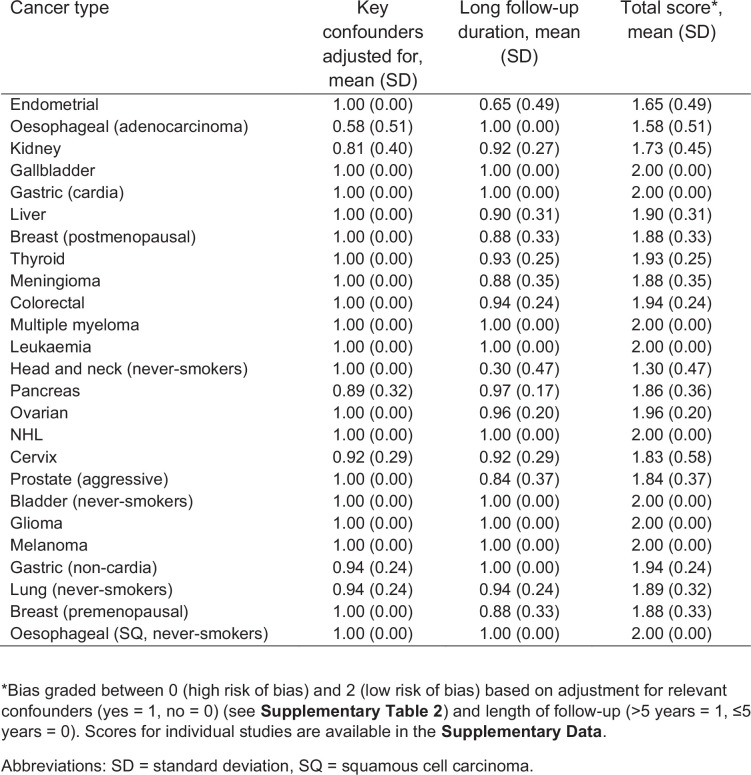
Bias assessment score for each cancer type

**Extended Data Table 3:**
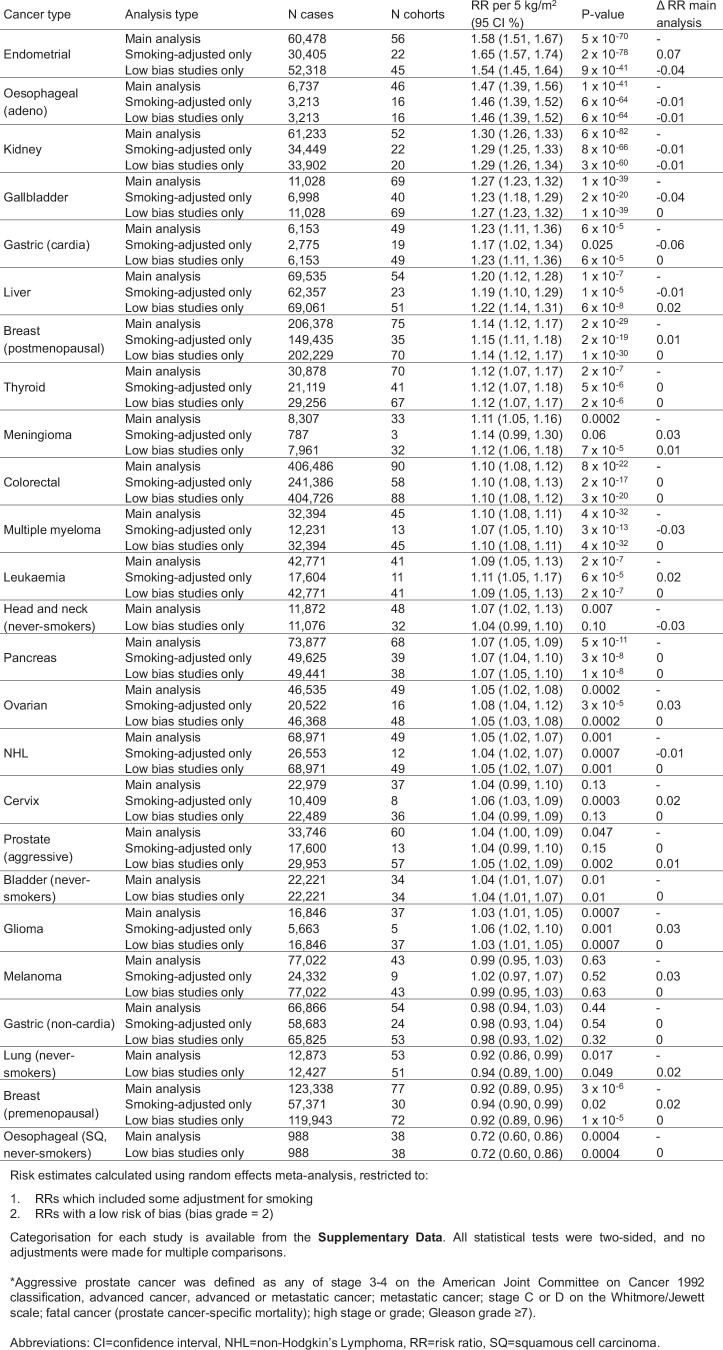
Sensitivity analyses by smoking adjustment and study bias

**Extended Data Table 4:**
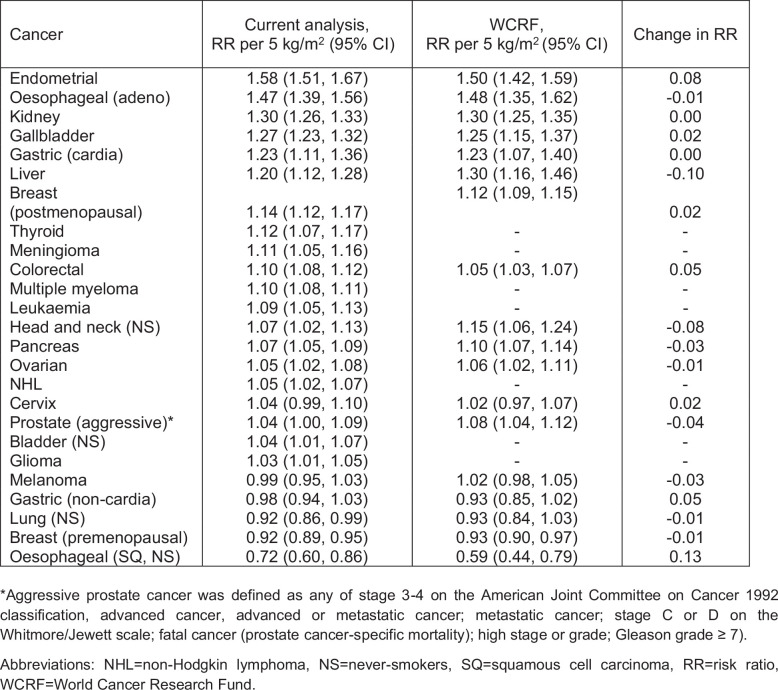
Comparison of current meta-analysis results with the WCRF review

**Extended Data Table 5:**
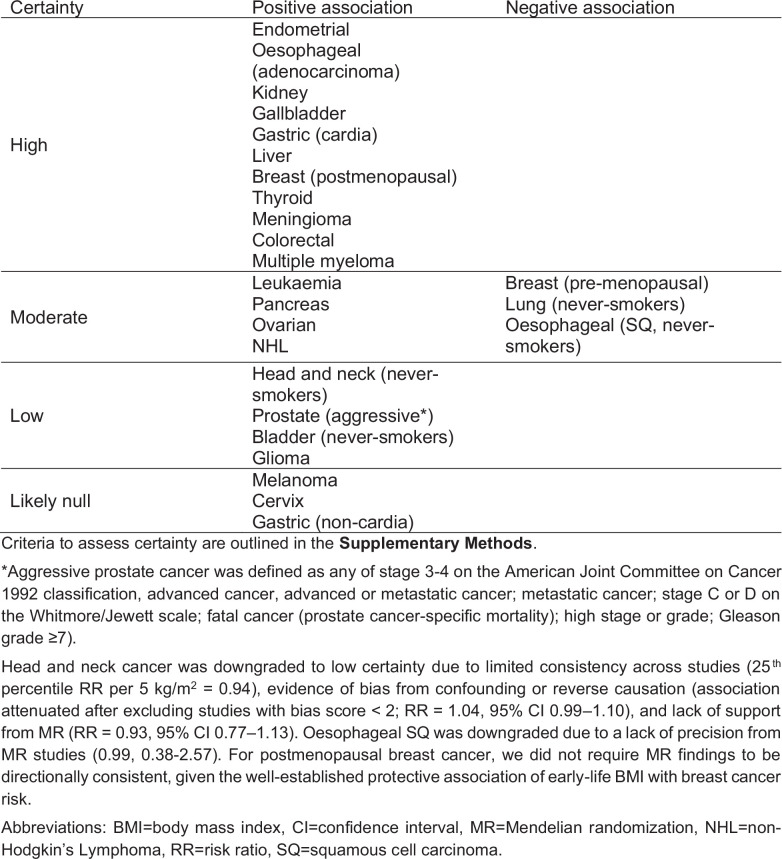
Certainty assessment from meta-analysis of BMI and cancer risk

**Extended Data Table 6:**
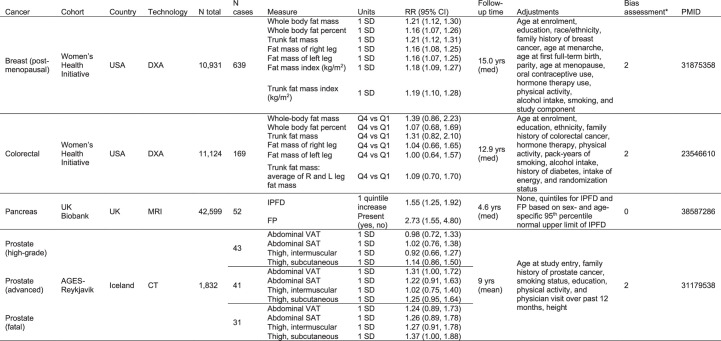
Adiposity imaging studies identified by search strategy

## Data Availability

All data synthesized in this meta-analysis are provided in the Supplementary Data files.

## Code availability

The statistical code created for this analysis can be obtained at https://github.com/elliewatts90/BMI_cancer_meta_analysis.
