# Supplementary material for: Adiposity and cancer: systematic review and meta-analysis
Source: Nat Metab. 2026 Jun 15;8(6):1426–39. doi: 10.1038/s42255-026-01542-8 (PMC13303081; doi:10.1038/s42255-026-01542-8)
Supplement: Supplementary file 1 — Supplementary Methods, Tables 1 and 2 and Figs. 1–94. [file 42255_2026_1542_MOESM1_ESM.pdf]

nature metabolism

Supplementary information https://doi.org/10.1038/s42255-026-01542-8

Adiposity and cancer: systematic review and meta-analysis

In the format provided by the authors and unedited

Table of Contents

Supplementary Methods 2
1. Cancer selection 2
2. Search Strategy 2
3. Inclusion and exclusion criteria 3
3. Selection process 3
4. Data extraction 4
5. Risk harmonisation 5
6. Statistical methods 5
Meta-analysis 5
Heterogeneity by region 6
Heterogeneity in the associations of BMI and waist circumference with cancer risk 6
Assessment of study bias 6
Assessment of publication bias 7
Sensitivity analyses 8
7. Mendelian randomisation meta-analysis 8
Exclusion criteria 9
Data extraction and meta-analysis 9
8. Assessment of certainty 10
9. Imaging studies 10
10. Software and packages 12
11. References 118

Supplementary Tables

Supplementary Table 1: Literature search terms 13
Supplementary Table 2: Criteria for assessing key confounders for each cancer type 17

Supplementary Figures

Supplementary Figure 1: Meta-analysis of prospective studies on the risk of endometrial cancer in relation to BMI 20
Supplementary Figure 2: Meta-analysis of prospective studies on the risk of oesophageal adenocarcinoma cancer in relation to BMI 21
Supplementary Figure 3: Meta-analysis of prospective studies on the risk of kidney cancer in relation to BMI 22
Supplementary Figure 4: Meta-analysis of prospective studies on the risk of gallbladder cancer in relation to BMI 23
Supplementary Figure 5: Meta-analysis of prospective studies on the risk of gastric cancer (cardia) in relation to BMI 24
Supplementary Figure 6: Meta-analysis of prospective studies on the risk of liver cancer in relation to BMI 25
Supplementary Figure 7: Meta-analysis of prospective studies on the risk of breast (postmenopausal) cancer in relation to BMI 26
Supplementary Figure 8: Meta-analysis of prospective studies on the risk of thyroid cancer in relation to BMI 27
Supplementary Figure 9: Meta-analysis of prospective studies on the risk of meningioma in relation to BMI 28
Supplementary Figure 10: Meta-analysis of prospective studies on the risk of colorectal cancer in relation to BMI 29
Supplementary Figure 11: Meta-analysis of prospective studies on the risk of multiple myeloma in relation to BMI 30

Supplementary Figure 12: Meta-analysis of prospective studies on the risk of leukaemia in relation to BMI 31
Supplementary Figure 13: Meta-analysis of prospective studies on the risk of head and neck cancer (restricted to never-smokers) in relation to BMI 32
Supplementary Figure 14: Meta-analysis of prospective studies on the risk of pancreatic cancer in relation to BMI 33
Supplementary Figure 15: Meta-analysis of prospective studies on the risk of ovarian cancer in relation to BMI 34
Supplementary Figure 16: Meta-analysis of prospective studies on the risk of NHL in relation to BMI... 35
Supplementary Figure 17: Meta-analysis of prospective studies on the risk of cervical cancer in relation to BMI 36
Supplementary Figure 18: Meta-analysis of prospective studies on the risk of prostate cancer (aggressive) in relation to BMI 37
Supplementary Figure 19: Meta-analysis of prospective studies on the risk of bladder cancer (restricted to never-smokers) in relation to BMI 38
Supplementary Figure 20: Meta-analysis of prospective studies on the risk of glioma in relation to BMI 39
Supplementary Figure 21: Meta-analysis of prospective studies on the risk of melanoma in relation to BMI 40
Supplementary Figure 22: Meta-analysis of prospective studies on the risk of gastric cancer (non-cardia) in relation to BMI 41
Supplementary Figure 23: Meta-analysis of prospective studies on the risk of lung cancer (restricted to never-smokers) in relation to BMI 42
Supplementary Figure 24: Meta-analysis of prospective studies on the risk of breast cancer (premenopausal) in relation to BMI 43
Supplementary Figure 25: Meta-analysis of prospective studies on the risk of oesophageal (SQ, restricted to never-smokers) in relation to BMI 44
Supplementary Figure 26: Trim-and-fill for publication bias and Egger's intercept test for the 25 common cancers 45
Supplementary Figure 27: Meta-analysis of prospective studies on the risk of endometrial cancer in relation to BMI, by region 50
Supplementary Figure 28: Meta-analysis of prospective studies on the risk of oesophageal adenocarcinoma cancer in relation to BMI, by region 51
Supplementary Figure 29: Meta-analysis of prospective studies on the risk of kidney cancer in relation to BMI, by region 52
Supplementary Figure 30: Meta-analysis of prospective studies on the risk of gallbladder cancer in relation to BMI, by region 53
Supplementary Figure 31: Meta-analysis of prospective studies on the risk of gastric cancer (cardia) in relation to BMI, by region 54
Supplementary Figure 32: Meta-analysis of prospective studies on the risk of liver cancer in relation to BMI, by region 55
Supplementary Figure 33: Meta-analysis of prospective studies on the risk of breast cancer (postmenopausal) in relation to BMI, by region 56
Supplementary Figure 34: Meta-analysis of prospective studies on the risk of thyroid cancer in relation to BMI, by region 57
Supplementary Figure 35: Meta-analysis of prospective studies on the risk of meningioma cancer in relation to BMI, by region 58
Supplementary Figure 36: Meta-analysis of prospective studies on the risk of colorectal cancer in relation to BMI, by region 59
Supplementary Figure 37: Meta-analysis of prospective studies on the risk of multiple myeloma in relation to BMI, by region 60

Supplementary Figure 38: Meta-analysis of prospective studies on the risk of leukaemia in relation to BMI, by region 61
Supplementary Figure 39: Meta-analysis of prospective studies on the risk of head and neck cancer (restricted to never-smokers) in relation to BMI, by region 62
Supplementary Figure 40: Meta-analysis of prospective studies on the risk of pancreatic cancer in relation to BMI, by region 63
Supplementary Figure 41: Meta-analysis of prospective studies on the risk of ovarian cancer in relation to BMI, by region 64
Supplementary Figure 42: Meta-analysis of prospective studies on the risk of NHL in relation to BMI, by region 65
Supplementary Figure 43: Meta-analysis of prospective studies on the risk of cervical cancer in relation to BMI, by region 66
Supplementary Figure 44: Meta-analysis of prospective studies on the risk of aggressive prostate cancer in relation to BMI, by region 67
Supplementary Figure 45: Meta-analysis of prospective studies on the risk of bladder cancer (restricted to never-smokers) in relation to BMI, by region 68
Supplementary Figure 46: Meta-analysis of prospective studies on the risk of glioma in relation to BMI, by region 69
Supplementary Figure 47: Meta-analysis of prospective studies on the risk of melanoma in relation to BMI, by region 70
Supplementary Figure 48: Meta-analysis of prospective studies on the risk of gastric cancer (non-cardia) in relation to BMI, by region 71
Supplementary Figure 49: Meta-analysis of prospective studies on the risk of lung cancer (restricted to never-smokers) in relation to BMI, by region 72
Supplementary Figure 50: Meta-analysis of prospective studies on the risk of breast cancer (premenopausal) in relation to BMI, by region 73
Supplementary Figure 51: Meta-analysis of prospective studies on the risk of oesophageal SQ in relation to BMI, by region 74
Supplementary Figure 52: Meta-analysis of prospective studies on the risk of oesophageal adenocarcinoma in relation to BMI, by sex 75
Supplementary Figure 53: Meta-analysis of prospective studies on the risk of kidney cancer in relation to BMI, by sex 76
Supplementary Figure 54: Meta-analysis of prospective studies on the risk of gallbladder cancer in relation to BMI, by sex 77
Supplementary Figure 55: Meta-analysis of prospective studies on the risk of gastric (cardia) cancer in relation to BMI, by sex 78
Supplementary Figure 56: Meta-analysis of prospective studies on the risk of liver cancer in relation to BMI, by sex 79
Supplementary Figure 57: Meta-analysis of prospective studies on the risk of thyroid cancer in relation to BMI, by sex 80
Supplementary Figure 58: Meta-analysis of prospective studies on the risk of meningioma cancer in relation to BMI, by sex 81
Supplementary Figure 59: Meta-analysis of prospective studies on the risk of colorectal cancer in relation to BMI, by sex 82
Supplementary Figure 60: Meta-analysis of prospective studies on the risk of multiple myeloma in relation to BMI, by sex 83
Supplementary Figure 61: Meta-analysis of prospective studies on the risk of leukaemia in relation to BMI, by sex 84
Supplementary Figure 62: Meta-analysis of prospective studies on the risk of pancreatic cancer in relation to BMI, by sex 85

Supplementary Figure 63: Meta-analysis of prospective studies on the risk of NHL in relation to BMI, by sex 86
Supplementary Figure 64: Meta-analysis of prospective studies on the risk of bladder cancer (never-smokers) in relation to BMI, by sex 87
Supplementary Figure 65: Meta-analysis of prospective studies on the risk of glioma in relation to BMI, by sex 88
Supplementary Figure 66: Meta-analysis of prospective studies on the risk of melanoma in relation to BMI, by sex 89
Supplementary Figure 67: Meta-analysis of prospective studies on the risk of gastric (non-cardia) cancer in relation to BMI, by sex 90
Supplementary Figure 68: Meta-analysis of prospective studies on the risk of lung cancer (never-smokers) in relation to BMI, by sex 91
Supplementary Figure 69: Associations of BMI, waist circumference and endometrial cancer risk, per 1 SD increase 92
Supplementary Figure 70: Associations of BMI, waist circumference and oesophageal adenocarcinoma risk, per 1 SD increase 93
Supplementary Figure 71: Associations of BMI, waist circumference and kidney cancer risk, per 1 SD increase 94
Supplementary Figure 72: Associations of BMI, waist circumference and gallbladder cancer risk, per 1 SD increase 95
Supplementary Figure 73: Associations of BMI, waist circumference and gastric cancer (cardia) risk, per 1 SD increase 96
Supplementary Figure 74: Associations of BMI, waist circumference and liver cancer risk, per 1 SD increase 97
Supplementary Figure 75: Associations of BMI, waist circumference and breast (postmenopausal) cancer risk, per 1 SD increase 98
Supplementary Figure 76: Associations of BMI, waist circumference and thyroid cancer risk, per 1 SD increase 99
Supplementary Figure 77: Associations of BMI, waist circumference and meningioma risk, per 1 SD increase 100
Supplementary Figure 78: Associations of BMI, waist circumference and colorectal cancer risk, per 1 SD increase 101
Supplementary Figure 79: Associations of BMI, waist circumference and multiple myeloma risk, per 1 SD increase 102
Supplementary Figure 80: Associations of BMI, waist circumference and leukaemia risk, per 1 SD increase 103
Supplementary Figure 81: Associations of BMI, waist circumference and head and neck cancer risk, per 1 SD increase 104
Supplementary Figure 82: Associations of BMI, waist circumference and pancreatic cancer risk, per 1 SD increase 105
Supplementary Figure 83: Associations of BMI, waist circumference and ovarian cancer risk, per 1 SD increase 106
Supplementary Figure 84: Associations of BMI, waist circumference and NHL risk, per 1 SD increase 107
Supplementary Figure 85: Associations of BMI, waist circumference and cervical cancer risk, per 1 SD increase 108
Supplementary Figure 86: Associations of BMI, waist circumference and prostate cancer risk, per 1 SD increase 109
Supplementary Figure 87: Associations of BMI, waist circumference and fatal prostate cancer risk, per 1 SD increase 110
Supplementary Figure 88: Associations of BMI, waist circumference and bladder cancer risk, per 1 SD increase 111

Supplementary Figure 89: Associations of BMI, waist circumference and melanoma risk, per 1 SD increase 112
Supplementary Figure 90: Associations of BMI, waist circumference and gastric cancer (non-cardia) risk, per 1 SD increase 113
Supplementary Figure 91: Associations of BMI, waist circumference and lung cancer risk, per 1 SD increase 114
Supplementary Figure 92: Associations of BMI, waist circumference and lung cancer risk (restricted to never-smokers), per 1 SD increase 115
Supplementary Figure 93: Associations of BMI, waist circumference and breast (premenopausal) cancer risk, per 1 SD increase 116
Supplementary Figure 94: Associations of BMI, waist circumference and oesophageal SQ cancer risk, per 1 SD increase 117

Supplementary Methods

1. Cancer selection

We selected cancer types *a priori* based on the 25 most common cancers by global incidence (https://www.wcrf.org/cancer-trends/worldwide-cancer-data/). Among these, we grouped cancers of the lip, oral cavity, larynx, nasopharynx, oropharynx, and hypopharynx as head and neck cancers per common convention. We subdivided cancers known to have etiologically distinct subtypes ("subtype-specific cancers") as follows: gastric (cardia, non-cardia), breast (pre- and post-menopausal), oesophageal (adenocarcinoma and squamous cell carcinoma), and central nervous system (CNS; glioma and meningioma).

We conducted special analyses for two groups of cancers, smoking-related cancers and prostate cancer, to address biases of particular importance for these outcomes. For the four cancers most strongly related to smoking (head and neck, lung, oesophageal squamous cell carcinoma (SQ) and bladder), per Freedman et al.[1], we restricted analyses to never smokers to eliminate or reduce residual confounding by smoking. For prostate cancer, we restricted analyses to aggressive cancers to minimise screening bias, which can occur when healthier men are more likely to be screened and have indolent prostate cancers detected[2]. Aggressive prostate cancer was defined as any of stage 3-4 on the American Joint Committee on Cancer 1992 classification, advanced cancer, advanced or metastatic cancer; metastatic cancer; stage C or D on the Whitmore/Jewett scale; fatal cancer (prostate cancer-specific mortality); high stage or grade; Gleason grade ≥ 7). This definition was based on previous World Cancer Research Fund (WCRF) reviews[3].

Ultimately, the following 25 cancer types were investigated: bladder (never-smokers only), breast cancer (pre and postmenopausal), cervix, CNS (glioma and meningioma), colorectal, endometrial, gallbladder, gastric (non-cardia and cardia), head and neck (never-smokers only), kidney, leukaemia, liver, lung (never smokers only), melanoma, multiple myeloma, non-Hodgkin lymphoma (NHL), oesophageal (SQ never-smokers only and adenocarcinoma), ovarian, pancreas, prostate (aggressive), and thyroid.

2. Search Strategy

We conducted a systematic search to identify relevant studies using the PubMed, EMBASE, and Scopus databases. The search covered articles published from the databases' inception through April 23rd, 2025. For each cancer type, studies examining the relationship between BMI and cancer risk were identified using specific search terms, which are detailed in **Supplementary Table 1**.

To ensure our literature search was complete, we searched reference lists of Larrson et al.[4], which included a narrative review of studies published from 2018-2021, and the most recent article for each cancer type. Additionally, we reviewed the WCRF reports and included relevant articles not identified by our initial search strategy. Our methodology adhered to the standard criteria for reporting meta-analyses as outlined in the Preferred Reporting Items for Systematic reviews and

Meta-Analyses (PRISMA) guidelines[5]. Our analysis was not pre-registered because it is a revision of an invited meta-analysis. As a result, it was not possible to prospectively register the protocol.

3. Inclusion and exclusion criteria

Our inclusion criteria were for original large-scale prospective analyses utilising individual-level data that explored associations between adult (≥18 years) BMI and the risk of the 25 most common cancers. We did not impose any language restrictions in our search.

Studies were excluded based on the following criteria:

1) Non-cancer incidence outcome (e.g., survival/prognosis/wrong outcome) or non-prospective design.
2) N<50,000 participants in the study cohort or pooled study (unless outside North America, North/West/Central Europe, or East/Southeast Asia).
3) Risk estimates not convertible to continuous estimates, such as where only two BMI categories were reported (e.g., risk estimates comparing obese and non-obese participants only).
4) No risk estimates available for subtypes for subtype-specific cancers: breast cancer (pre and postmenopausal), CNS (glioma and meningioma), gastric (non-cardia and cardia), oesophageal (SQ and adenocarcinoma). In East and Southeast Asia >90% of oesophageal cancers are SQ and >80% of gastric cancers are non-cardia[7], therefore for cohorts in this region, cancers were categorised into respective subtypes if not otherwise stated.
5) No risk estimates for never-smokers for smoking-related cancers: head and neck, lung, oesophageal SQ and bladder.
6) Risk estimates were based on models which adjusted for other measures of adiposity (such as waist circumference) or early-life body size only (due to collinearity of variables with adult BMI).
7) Duplicate study population. Where one cohort had multiple publications identified, the publication with the greatest number of cases was included.

The number of studies excluded based on each exclusion criterion is detailed in **Extended Data Figure 1.** For studies identified in the literature search that reported results for multiple cancers, BMI-cancer associations were extracted for each reported cancer type and screened using the process outlined above.

3. Selection process

We utilized Covidence web-based software (https://www.covidence.org/) for the screening process. After removing duplicates, titles and abstracts were screened against established eligibility criteria. Articles that met these criteria in the initial screening were then subjected to a full-text review, where the eligibility criteria were applied once again. Screening and review were

conducted independently by two authors (ELW and SCM), in the case of discrepancies AGF acted as the tie-break.

Eligible articles were extracted into Excel and categorised by cohort and cancer type. For cohorts with multiple publications on the association between BMI and a specific cancer type, the analysis with the largest number of cases was selected by ELW.

4. Data extraction

Data was extracted by ELW and AGF independently. For each study, risk estimates (hazard ratios [HRs], risk ratios [RRs] or odds ratios [ORs]) and their 95% confidence intervals (CIs) for the association of BMI with incident cancer risk were extracted, preferentially selecting estimates based on a continuous modelling of BMI. If continuous risk estimates were unavailable and BMI had at least three categories, categorical estimates were extracted. The estimates with the most comprehensive adjustments for confounders that did not also adjust for other measures of adiposity (such as waist circumference), or BMI from an earlier age were extracted.

Where estimates originated from pooled analyses arising from many different cohorts, study-specific risk estimates were extracted whenever possible. However, 15 articles provided only pooled estimates, precluding us from extracting study-specific estimates. For these consortia, we contacted the corresponding authors, who provided us with study-specific estimates for three articles[8–10]. For the remaining articles, we used pooled estimates, provided that the number of cases in the pooled study exceeded those reported in the individual study publications.

When multiple analyses published from the same cohort for each cancer type (e.g., Korean National Health Insurance Service [KNHIS] database), we extracted risk estimates from the study with the most cases. Where studies reported risk estimates for specific cancer subtypes (e.g., colon and rectal cancer) but not for the broader cancer group (e.g., colorectal cancer), we extracted and meta-analysed associations for each subtype to produce a single pooled estimate. This approach was adopted to maximise the use of available data and to avoid inflating heterogeneity in the primary meta-analysis.

For haematological cancers, which have been recently reclassified[11], we adhered to ICD-10 conventions where possible to ensure consistency across studies and to allow the inclusion of earlier research. The classifications used were as follows: NHL (C82-C85), multiple myeloma (C90), leukaemia (C91-C95).

The following information was extracted for each study: citation details (first author and publication year), study design (country, geographic region, N cohorts, follow-up duration [median preferred], large administrative health database study [yes; no]), outcome (cancer type and subtype, if applicable, N cases, N analytic set and the scale reported in the model), and adjustment covariates.

For studies included in our BMI-cancer analysis that investigated ≥5 cancer types and reported risk estimates for both BMI and waist circumference on the same scale or provided continuous risk estimates that could be converted to standard deviations (SDs), we extracted both sets of risk estimates. We also reviewed studies flagged by our search strategy but excluded from the main BMI-cancer analysis due to overlapping study populations, as exclusion based on missing waist circumference data occasionally resulted in smaller analytic cohorts and complementary findings[12]. In this analysis, we included risk estimates for smoking-related cancers regardless of smoking status, given evidence that smoking may differentially influence overall adiposity and fat distribution[13].

5. Risk harmonisation

Risk estimates were converted to the 5 kg/m^2 scale to enable comparison across studies. Where estimates were reported in a different continuous scale (e.g., per 1 SD), HRs and 95% CIs were rescaled to HR per 5 kg/m^2 using the formula: HR per 5 kg/m^2= $HR_x{}^{5/x}$, where x is equal to the originally reported units. The same method was used for the upper and lower CIs. For HRs reported as categorical estimates, we converted associations to a continuous scale using methods described in detail elsewhere[14]. This process involved estimating the midpoints of each BMI category (when not explicitly stated in the original article) and extracting sample sizes for each category and the continuous dose-response association was then estimated using restricted maximum likelihood via the dosresmeta R package[15].

For our investigation comparing BMI and waist circumference measures, continuous risk estimates were converted to SDs to allow comparison. One study also applied regression dilution corrections for BMI (regression dilution ratio [RDR]= 0.93) and waist circumference (0.81), which would amplify differences between the two measures[12]. To ensure comparability on the same scale, we removed this correction by applying the inverse transformation to the reported RRs and 95% CIs using the formula: $RR_{uncorrected} = (RR_{corrected})^{RDR}$.

All results are available in a harmonised excel file in the **Supplementary Data**.

6. Statistical methods

Meta-analysis

Summary risk estimates for the association of BMI with each cancer type were estimated using random effects meta-analysis, which weights studies based on within-study and between-study variability and calculated a weighted average of individual study effect size. Heterogeneity across studies was assessed using I^2, tau^2, and the 25th and 75th percentile (interquartile range) of RRs for each cancer type. I^2 estimates the percent of total variation due to true differences in effect sizes between studies; an I^2 close to 100% indicates that chance plays little role in observed differences. However, I^2 is highly sensitive to sample size, when studies are large, even small differences can yield large I^2 values[16]. Tau^2 more directly quantifies differences in effect sizes, it is the between-

study variance of the estimated underlying distribution of true effect sizes. A value close to 0 suggests that the true effect size varies little between populations. This metric was calculated using the Paule-Mandel estimator[17]. Finally, we present the interquartile range as a more intuitive metric, showing the typical spread of estimates.

Heterogeneity by region

We assessed heterogeneity in the associations of BMI and each cancer type by region. Cohorts were clustered into geographic regions: Oceania (Australia), North America (USA and Canada), Europe (Austria, Denmark, Finland, France, Germany, Greece, Italy, Netherlands, Norway, Lithuania, Spain, Sweden, UK), East Asia (Japan, South Korea, China, Singapore), South Asia (India), Middle East (Iran and Israel). Although Singapore is geographically located in Southeast Asia, the only study identified was the Singapore Chinese Health Study, which had a relatively small study population (N=51,000) and was specifically recruited from the Chinese population of Singapore. Therefore, Singapore was grouped with East Asia. Heterogeneity in the associations by geographic region was quantified using the Q-statistic.

Heterogeneity by sex

For each BMI-cancer risk association included in the primary analysis, we searched the corresponding article for sex-specific BMI-cancer estimates. Analyses conducted in single-sex cohorts were also included (e.g., the Nurses' Health Study). Between-sex heterogeneity was assessed using the Q-statistic.

Heterogeneity in the associations of BMI and waist circumference with cancer risk

The associations of BMI and waist circumference were based on the same population; therefore, heterogeneity was tested using the Wald statistic:

$$Wald\ statistic = \frac{\beta_1 - \beta_2}{\sqrt{Var(\beta_1) + Var(\beta_2) - 2Cov(\beta_1, \beta_2)}}$$

Where:

β_1 = log RR per 1 standard deviation increase in BMI

β_2= log RR per 1 standard deviation increase in waist circumference

$Cov = r \times \sqrt{Var(\beta_1) \times Var(\beta_2)}$

r= correlation coefficient of BMI and waist circumference (r= 0.87)[18]

Assessment of study bias

We assessed study bias using a simplified version of the Berrington de González et al. framework originally developed to review ionizing radiation and cancer risk[19]. Briefly, this framework considers four major sources of epidemiological bias and evaluates their potential relevance to study findings, including the likely direction and magnitude of any bias. In our assessment, substantial selection bias, information bias (referred to as "dose error"), and outcome

ascertainment bias were considered unlikely, as our analysis was restricted to prospective cohort studies. Confounding was deemed a more serious concern, along with the possibility of reverse causation, whereby preclinical cancer might lead to weight loss, inducing a spurious inverse association between BMI and cancer risk.

To evaluate these concerns, we applied a two-point grading system to each study, evaluating:

i) adjustment for major confounders, and
ii) whether follow-up exceeded 5-years, which should minimize reverse causation.

To develop our lists of cancer-specific confounders, we considered both the direction and, importantly the magnitude of confounding needed to explain BMI-cancer associations, as recommended by Berrington de González et al. We specifically adopted the approach of Ding and VanderWeele[20], which enables estimation of the magnitude of confounder-disease associations needed to account for exposure-disease associations. For our calculations, we assumed a RR of 1.2 for obesity-cancer associations (vs a reference group of normal weight). This is comparable to a RR of ~1.1 per 5 kg/m^2, assuming a 10-unit BMI difference between individuals with obesity and those with normal weight). We also assumed a RR of 1.5 for obesity-confounder associations, a strong but plausible magnitude of effect. Under these assumptions, a confounder would need an RR of 2 or greater with cancer to fully account for the observed association, and we therefore considered any cancer risk factor with a reported RR ≥ 2 as a potential major confounder.

To ensure a streamlined, high-quality, and systematic approach, we identified candidate confounders by reviewing type-specific chapters in the Schottenfeld and Fraumeni *Cancer Epidemiology and Prevention 4th edition*[21], a widely used text offering standardised expert assessments of cancer risk factors. For assessment of sex as a confounder a recent UK Biobank pan-cancer analysis was used[22]. For those risk factors with a RR ≥ 2 (**Supplementary Table 2**), we excluded likely mediators (to avoid overadjustment), rare exposures (e.g. liver flukes, which are uncommon in the populations we studied), and exposures not consistently associated with BMI (e.g. ionizing radiation). Occupational exposures were also excluded due to low prevalence. Remaining risk factors were classified as important potential confounders. We also included age a key confounder for all cancers and required that studies either adjusted for age or used age as the underlying time scale in Cox models for assessment of bias[23]. Studies that adjusted for all key confounders (or for which no important confounders were identified) received 1 point; otherwise, they received 0 points.

We also assessed whether studies had sufficient follow-up time to mitigate the possibility of reverse causation. Studies with at least five years of follow-up were awarded 1 point, while those with shorter follow-up received 0 points. If information on confounder adjustment or follow-up duration was not reported, no grade was assigned for that criterion.

<u>Assessment of publication bias</u>

We used Egger's test to assess publication bias and applied the trim-and-fill method to re-estimate the effect size by accounting for potentially missing studies. The trim-and-fill method

identifies and removes outlying studies to recalculate the overall effect size. Hypothetical studies are then imputed to restore symmetry to the funnel plot, mirroring the distribution of observed studies, and an adjusted effect size is subsequently calculated[24].

Sensitivity analyses

In sensitivity analyses, we restricted the meta-analysis to studies that adjusted for smoking, a factor independently associated with both BMI and many cancers[1]. Some individual study risk estimates were derived entirely from national health databases rather than traditional prospective cohorts, which tend to have more limited control for residual confounding. For example, the Clinical Practice Research Datalink (CPRD) has ~50% missing smoking data[25]. Additionally, these databases are often not linked to gold-standard cancer registry data, increasing the possibility of misclassification biases[26,27]. These databases typically comprise large populations that can influence summary estimates. Therefore, we additionally meta-analysed BMI-cancer associations stratified by study design (large administrative healthcare databases vs traditional cohort studies) and compared risk estimates. We also examined changes in risk estimates after restricting analyses to studies with a low risk of bias (quality assessment score = 2).

Inclusion details for each study and sensitivity analysis are provided in the **Supplementary Data**.

7. Mendelian randomisation meta-analysis

In secondary analyses, we also investigated associations of BMI with cancer using Mendelian randomisation (MR) approaches. MR leverages genetic variants as instrumental variables to assess potential causal relationships, reducing the risk of confounding and reverse causation that can affect observational studies[29].

Search strategy

For each of the cancer types included in our BMI-cancer meta-analysis, we conducted a systematic search for MR studies. The literature search was conducted in PubMed, EMBASE, and Scopus, covering articles published from the databases' inception through February 6, 2025.

The search strategy included the following terms:

PubMed: cancer*[tiab] NOT "non-cancer*" [tiab] AND ("Mendelian randomization"[tiab] OR "Mendelian randomization"[ot]) AND ("body mass index"[ti] OR bmi[ti] OR ((obesity[ti] OR adipos*[ti] OR body fat*[ti] OR body weight[ti] OR body mass[ti] OR body composition[ti] OR lifestyle[ti] OR anthropom*[ti]) AND (bmi[tiab] OR bmi[ot] OR "body mass index"[tiab:~0] OR "body mass index"[ot]))) NOT (patient*[ti] OR prognosis[ti] OR surg*[ti] OR complication*[ti] OR surviv*[ti] OR screen*[ti] OR infection*[ti] OR virus[ti])

EMBASE: 'cancer*':ti,ab,kw AND 'Mendelian randomization':ti,ab,kw AND ('body mass index':ti OR 'bmi':ti OR (('obesity':ti OR 'adipos*':ti OR 'body fat*':ti OR 'body weight':ti OR 'body mass':ti OR 'body composition':ti OR 'lifestyle':ti OR 'anthropom*':ti) AND ('bmi':ti,ab,kw OR 'body mass index':ti,ab,kw))) NOT ('patient*':ti OR 'prognosis':ti OR 'surg*':ti OR 'complication*':ti OR 'surviv*':ti OR 'screen*':ti OR 'infection*':ti OR 'virus':ti) AND 'article'/it

Scopus: TITLE-ABS-KEY(cancer*) AND NOT TITLE-ABS-KEY("non-cancer") AND TITLE-ABS-KEY("Mendelian randomization") AND (TITLE ("body mass index" OR {bmi}) OR (TITLE("obesity" OR "adipos*" OR "body fat*" OR "body weight" OR "body mass" OR "body composition" OR "lifestyle" OR "anthropom*") AND TITLE-ABS-KEY({bmi} OR {body mass index}))) AND NOT TITLE(patient* OR "prognosis" OR surg* OR complication* OR surviv* OR screen* OR infection* OR "virus") AND (LIMIT-TO(DOCTYPE,"ar")

MR studies not captured by these searches were identified using reference review.

Exclusion criteria

The exclusion criteria were based on the BMI-cancer analysis, with the following criteria applied:

1) Non-cancer incidence outcome, not top 25 cancer, or no BMI RRs.
2) No risk estimates available for subtypes for subtype-specific cancers.
3) Non-linear ORs.
4) Duplicative study population in the cancer GWAS population.

Articles underwent title and abstract screening by ELW, with those meeting the inclusion criteria proceeding to full-text review. Data was extracted by ELW, given prior experience in this methodology.

Data extraction and meta-analysis

The following information was extracted for each study: citation details (first author and publication year), genetic instrument (study, N participants, ancestry, PMID of original GWAS), outcome (study, cancer type, N cases, N controls, ancestry, PMID of original GWAS), and results (OR, 95% CI, model [e.g., inverse-variance weighted random effects], originally reported units). Univariable MR risk estimates were extracted, with preference given to those based on the inverse variance weighted method, converted to the 5 kg/m^2 scale for comparability across studies, and meta-analysed using random effects models (as described in **Section 6**). Heterogeneity between studies was assessed using I^2.

For smoking-related cancers, when studies provided multivariable MR analyses adjusted for genetically predicted smoking, we preferentially extracted these estimates (lung and head and neck cancers), else univariable MR estimates were extracted (bladder and oesophageal SQ).

The number of included studies for each cancer was low (maximum five) and primarily limited to European ancestry populations. Therefore, we did not assess publication bias or heterogeneity by ancestry. Additionally, as this analysis is secondary, we did not include a study bias evaluation.

8. Assessment of certainty

Standard tools such as GRADE (Grading of Recommendations Assessment, Development, and Evaluation) are designed for intervention studies and automatically rate observational evidence as "low certainty"[30]. For exposures like BMI, where randomised controlled trials are not feasible, this could undervalue robust prospective data. We therefore applied a tailored certainty assessment that emphasises domains most relevant to observational epidemiology, adapted from the GRADE framework: large effect size, risk of bias from confounding or reverse causation, consistency, risk of publication bias, and concordance with complementary MR evidence. GRADE also considers three additional domains—precision, directness, and dose-response. However, because we restricted inclusion to studies with analytic populations of ≥50,000, we did not apply an additional minimum case-count criterion. Moreover, all included studies directly addressed the exposure–outcome question of interest, so we did not consider indirectness to be a serious limitation We also did not assess dose–response separately, as our primary analyses already modelled BMI continuously per 5 kg/m^2. Our certainty criteria were as follows:

Certainty level	Criteria
High	Large effect size: RR > abs(10%)<br>Risk of bias: Association remains following exclusion of studies with bias score < 2<br>Consistency:<br>- For positive associations (RR > 1): 25th percentile of study-specific RRs > 1<br>- For inverse associations (RR < 1): 75th percentile of study-specific RRs < 1<br>Publication bias: association remains following fill-and-trim analysis<br>Concordance with complementary MR evidence: MR studies are directionally consistent*
Moderate	RR > abs(5%) but all other criteria from "High" are met
Low	Association nominally significant ($p < 0.05$) but did not meet the criteria for "High" or "Moderate" certainty
Likely null	Limited evidence of an association ($p \geq 0.05$)

*Cancer types supported by strong observational data were not downgraded if MR analyses were unavailable. For postmenopausal breast cancer, we did not require MR findings to be directionally consistent, given the well-established protective association of early-life BMI with breast cancer risk[31].

9. Imaging studies

In secondary analyses, we also assessed the evidence for associations between imaging-based body composition measures and cancer risk.

Search strategy

Our literature search was conducted in PubMed, EMBASE, and Scopus, covering articles published from the databases' inception through February 6th, 2025.

The search strategy included the following terms:

PubMed: cancer*[tiab] AND ((adiposity) OR (body fat) OR (body composition)) AND ((DXA scan) OR (DEXA scan) OR (DEXA) OR (DXA) OR (CT scan) OR (MRI)) AND (RR[tiab] OR RR[ot] OR "relative risk"[tiab] OR "relative risk"[ot] OR "hazard ratio"[tiab] OR "hazard ratio"[ot] OR ("HR"[tiab] NOT "heart rate"[tiab])) NOT (child*[ti] OR patient*[ti] OR prognosis[ti] OR surg*[ti] OR complication*[ti] OR surviv*[ti] OR screen*[ti] OR infection*[ti] OR virus[ti])

EMBASE: 'cancer*':ti,ab,kw AND ('adiposity':ti,ab,kw OR 'body fat':ti,ab,kw OR 'body composition':ti,ab,kw) AND ('DXA scan':ti,ab,kw OR 'DEXA scan':ti,ab,kw OR 'DEXA':ti,ab,kw OR 'DXA':ti,ab,kw OR 'CT scan':ti,ab,kw OR 'MRI':ti,ab,kw) AND ('RR':ti,ab,kw OR ‘relative risk’:ti,ab,kw OR 'hazard ratio':ti,ab,kw OR ('HR':ti,ab NOT 'heart rate':ti,ab)) NOT ('child*':ti OR 'patient*':ti OR 'prognosis':ti OR 'surg*':ti OR 'complication*':ti OR 'surviv*':ti OR 'screen*':ti OR 'infection*':ti OR 'virus':ti) AND 'article'/it

Scopus: TITLE-ABS-KEY(cancer*) AND TITLE-ABS-KEY("adipos*" OR "body fat*" OR "body composition") AND TITLE-ABS-KEY(“DXA scan” OR “DEXA scan” OR “DEXA” OR “DXA” OR “CT scan” OR “MRI”) AND (TITLE-ABS-KEY(“RR” OR “relative risk” OR “hazard ratio”) OR TITLE-ABS("HR" AND NOT “heart rate”)) AND NOT TITLE(child* OR patient* OR "prognosis" OR surg* OR complication* OR surviv* OR screen* OR infection* OR "virus") AND (LIMIT-TO(DOCTYPE,"ar")

The exclusion criteria were aligned with those used in the BMI-cancer analyses, with the following criteria applied:

1) Non-cancer incidence outcome or non-prospective design.
2) No risk estimates available for subtypes for subtype-specific cancers.
3) No risk estimates for never-smokers for smoking-related cancer.
4) Indirect measure of adiposity imaging – e.g., estimated adiposity derived from a subset of participants with imaging data rather than requiring direct imaging for all participants.
5) Adjusted for a correlated measure of adiposity.
6) Duplicate study population.

We did not exclude studies based on the size and did not require risk estimates to be continuous or >2 categories.

Articles first underwent title and abstract screening, with those meeting the inclusion criteria proceeding to full-text review. This process was conducted independently by ELW and SCM, with AGF serving as adjudicator in cases of disagreement.

Data were extracted by ELW following the same rubric used for the BMI-cancer analysis. Given the lack of overlapping data across cancer types, a meta-analysis was not conducted.

10. Software and packages

Data were analysed in R (v.4.3.1) using the 'meta' (v7.0.0)[32], and dosresmeta (v4.3.1)[15] R packages, a two-tailed $p<0.05$ was considered statistically significant. Figures were created using ggplot (v 4.0.0), maps (v3.4.3), forestploter (1.1.3), and meta (v7.0.0) R packages.

Supplementary Tables

Supplementary Table 1: Literature search terms

Cancer type	PubMed search terms	EMBASE search terms	Scopus search terms
BODY OF SEARCH	AND ("body mass index"[ti] OR bmi[ti] OR ((obesity[ti] OR adipos*[ti] OR body fat*[ti] OR body weight[ti] OR body mass[ti] OR body composition[ti] OR lifestyle[ti] OR anthropom*[ti]) AND (bmi[tiab] OR bmi[ot] OR "body mass index"[tiab:~0] OR "body mass index"[ot]))) AND (cohort*[tiab] OR cohort*[ot] OR pool*[tiab] OR million[tiab] OR risk*[ti] OR incidence[ti] OR associat*[ti]) NOT (child*[ti] OR patient*[ti] OR prognosis[ti] OR surg*[ti] OR complication*[ti] OR surviv*[ti] OR screen*[ti] OR infection*[ti] OR virus[ti] OR odds[tiab] OR "ORs"[tiab]) NOT ("bmi-1"[ti] OR genome[ti] OR genetic[ti] OR gene[ti] OR Mendelian random*[ti] OR polymorphism*[ti] OR pathway*[ti] OR expression[ti] OR DNA[ti] OR RNA[ti] OR microRNA[ti] OR mRNA[ti] OR "prediction model"[ti]) AND ("journal article"[pt] OR "systematic review"[pt] OR "meta-analysis"[pt]) NOT review[pt]	AND ('body mass index':ti OR 'bmi':ti OR (('obesity':ti OR 'adipos*':ti OR 'body fat*':ti OR 'body weight':ti OR 'body mass':ti OR 'body composition':ti OR 'lifestyle':ti OR 'anthropom*':ti) AND ('bmi':ti,ab,kw OR 'body mass index':ti,ab,kw))) AND ('cohort*':ti,ab,kw OR 'pool*':ti,ab OR 'million':ti,ab OR 'risk*':ti OR 'incidence':ti OR 'associat*':ti) NOT ('child*':ti OR 'patient*':ti OR 'prognosis':ti OR 'surg*':ti OR 'complication*':ti OR 'surviv*':ti OR 'screen*':ti OR 'infection*':ti OR 'virus':ti OR 'odds':ti,ab OR 'ORs':ti,ab) NOT ('bmi-1':ti OR 'genome':ti OR 'genetic':ti OR 'gene':ti OR 'mendelian random*':ti OR 'polymorphism*':ti OR 'pathway*':ti OR 'expression':ti OR 'DNA':ti OR 'RNA*':ti OR 'microRNA':ti OR 'mRNA':ti OR 'prediction model':ti) AND 'article'/it NOT 'review':it	AND (TITLE ("body mass index" OR {bmi}) OR (TITLE("obesity" OR "adipos*" OR "body fat*" OR "body weight" OR "body mass" OR "body composition" OR "lifestyle" OR "anthropom*") AND TITLE-ABS-KEY({bmi} OR {body mass index}))) AND (TITLE-ABS-KEY(cohort*) OR TITLE-ABS(pool* OR {million}) OR TITLE(risk* OR {incidence} OR associat*)) AND NOT (TITLE(child* OR patient* OR "prognosis" OR surg* OR complication* OR surviv* OR screen* OR infection* OR "virus") OR TITLE-ABS("ORs" OR {odds})) AND NOT TITLE("BMI-1" OR "genome" OR "genetic" OR "gene" OR "mendelian random*" OR polymorphism* OR pathway* OR "expression" OR "DNA" OR "RNA*" OR "microRNA" OR "mRNA" OR "prediction model") AND (LIMIT-TO(DOCTYPE,"ar")
**Cancer-specific search terms. Add database-specific *body of search* to each (see below)**			

Cancer type	PubMed search terms	EMBASE search terms	Scopus search terms
Stomach / gastric: cardia, non-cardia OR Kidney / renal OR Liver / hepatic OR Lung / pulmonary OR Bladder OR Thyroid OR Ovarian / ovary OR Cervical / cervix OR Endometrial / endometrium	"stomach neoplasms"[MeSH] OR "stomach cancer*"[tiab] OR "gastric cancer*"[tiab] OR "gastric cardia"[tiab] OR "gastric non cardia"[tiab] OR "gastric noncardia"[tiab] OR "stomach cancer*"[ot] OR "gastric cancer*"[ot] OR "gastric cardia"[ot] OR "gastric non cardia"[ot] OR "gastric noncardia"[ot] OR "kidney neoplasms"[MeSH:noexp] OR "carcinoma, renal cell"[MeSH] OR "kidney cancer*"[tiab] OR "renal cancer*"[tiab] OR "renal carcinoma*"[tiab] OR "kidney cancer*"[ot] OR "renal cancer*"[ot] OR "renal carcinoma*"[ot] OR "liver neoplasms"[MeSH:noexp] OR "carcinoma, hepatocellular"[MeSH] OR "liver cancer*"[tiab] OR hepatocarcinoma*[tiab] OR hepatoma*[tiab] OR "liver cell carcinoma*"[tiab] OR "liver cancer*"[ot] OR hepatocarcinoma*[ot] OR hepatoma*[ot] OR "liver cell carcinoma*"[ot] OR "lung neoplasms"[MeSH] OR "lung cancer*"[tiab] OR "pulmonary cancer*"[tiab] OR "lung cancer*"[ot] OR "pulmonary cancer*"[ot] OR "urinary bladder neoplasms"[MeSH] OR "bladder cancer*"[tiab] OR "bladder cancer*"[ot] OR "thyroid neoplasms"[MeSH:noexp] OR "Thyroid Cancer, Papillary"[MeSH] OR "thyroid cancer*"[tiab] OR "thyroid papillary cancer"[tiab] OR "thyroid cancer*"[ot] OR "thyroid papillary cancer"[ot] OR "ovarian neoplasms"[MeSH:noexp] OR "ovarian cancer*"[tiab] OR "ovary cancer*"[tiab] OR "ovarian cancer*"[ot] OR "ovary cancer*"[ot] OR "uterine cervical neoplasms"[MeSH] OR "cervical cancer*"[tiab] OR "cervix cancer*"[tiab] OR "cervical cancer*"[ot] OR "cervix cancer*"[ot] OR "endometrial neoplasms"[MeSH] OR "endometr* cancer*"[tiab] OR "endometr* cancer*"[ot]	'stomach cancer'/exp OR 'stomach cancer*':ti,ab,kw OR 'gastric cancer*':ti,ab,kw OR 'gastric cardia':ti,ab,kw OR 'gastric non cardia':ti,ab,kw OR 'gastric noncardia':ti,ab,kw OR 'kidney cancer'/de OR 'renal cell carcinoma'/exp OR 'kidney cancer*':ti,ab,kw OR 'renal cancer*':ti,ab,kw OR 'renal carcinoma*':ti,ab,kw OR 'liver cancer'/de OR 'liver cell carcinoma'/exp OR 'liver cancer*':ti,ab,kw OR 'hepatocarcinoma*':ti,ab,kw OR 'hepatoma*':ti,ab,kw OR 'liver cell carcinoma*':ti,ab,kw OR 'lung cancer'/exp OR 'lung cancer*':ti,ab,kw OR 'pulmonary cancer*':ti,ab,kw OR 'bladder cancer'/exp OR 'bladder cancer*':ti,ab,kw OR 'thyroid cancer'/de OR 'thyroid papillary carcinoma'/exp OR 'thyroid cancer*':ti,ab,kw OR 'thyroid papillary cancer':ti,ab,kw OR 'ovary cancer'/de OR 'ovarian cancer*':ti,ab,kw OR 'ovary cancer*':ti,ab,kw OR 'uterine cervix cancer'/exp OR 'cervical cancer*':ti,ab,kw OR 'cervix cancer*':ti,ab,kw OR 'endometrium cancer'/exp OR 'endometr* cancer*':ti,ab,kw	TITLE-ABS-KEY ("stomach cancer*" OR "gastric cancer*" OR "gastric cardia" OR "gastric non cardia" OR "gastric noncardia" OR "kidney cancer*" OR "renal cancer*" OR "renal carcinoma*" OR "liver cancer*" OR "hepatocarcinoma*" OR "hepatoma*" OR "liver cell carcinoma*" OR "lung cancer*" OR "pulmonary cancer*" OR "bladder cancer*" OR "thyroid cancer*" OR "thyroid papillary cancer*" OR "ovarian cancer*" OR "ovary cancer*" OR "cervical cancer*" OR "cervix cancer*" OR "endometr* cancer*")

Cancer type	PubMed search terms	EMBASE search terms	Scopus search terms
Melanoma OR Multiple myeloma OR Non-Hodgkin lymphoma OR Leukaemia / Leukemia OR meningioma, glioma	"melanoma"[MeSH:noexp] OR melanoma*[tiab] OR melanoma*[ot] OR "multiple myeloma"[MeSH] OR "multiple myeloma*"[tiab] OR "multiple myeloma*"[ot] OR "lymphoma, non hodgkin"[MeSH] OR "non-Hodgkin* lymphoma*"[tiab] OR "nonHodgkin* lymphoma*"[tiab] OR "non-Hodgkin* lymphoma*"[ot] OR "nonHodgkin* lymphoma*"[ot] OR "leukemia"[MeSH] OR "leukemia*"[tiab] OR "leukaemia*"[tiab] OR "leukemia*"[ot] OR "leukaemia*"[ot] OR "meningioma"[MeSH] OR "glioma"[MeSH] OR "meningioma*"[tiab] OR "glioma*"[tiab] OR "meningioma*"[ot] OR "glioma*"[ot]	'melanoma'/de OR 'melanoma*':ti,ab,kw OR 'multiple myeloma'/exp OR 'multiple myeloma*':ti,ab,kw OR 'non-Hodgkin lymphoma'/exp OR 'non-hodgkin* lymphoma*':ti,ab,kw OR 'nonHodgkin* lymphoma*':ti,ab,kw OR 'leukemia'/exp OR 'leukemia*':ti,ab,kw OR 'leukaemia*':ti,ab,kw OR 'meningioma'/exp OR 'glioma'/exp OR 'meningioma*':ti,ab,kw OR 'glioma*':ti,ab,kw	TITLE-ABS-KEY (melanoma* OR "multiple myeloma*" OR "non-Hodgkin* lymphoma*" OR "nonHodgkin* lymphoma*" OR "leukemia*" OR "leukaemia*" OR "meningioma*" OR "glioma*")…AND body of search…
Gallbladder / part of biliary tract	("gallbladder neoplasms"[MeSH] OR "gallbladder cancer"[tiab:~1] OR "gall bladder cancer"[tiab:~1] OR "biliary tract cancer"[tiab:~1] OR "gallbladder cancers"[tiab:~1] OR "gall bladder cancers"[tiab:~1] OR "biliary tract cancers"[tiab:~1] OR "gallbladder cancer*"[ot] OR "gall bladder cancer*"[ot] OR "biliary tract cancer*"[ot]) NOT ("gallbladder stone*"[ti])	('gallbladder cancer'/exp OR 'gallbladder cancer*':ti,ab,kw OR 'gall bladder cancer*':ti,ab,kw OR 'biliary tract cancer*':ti,ab,kw) NOT ('gallbladder stone*':ti)	TITLE-ABS-KEY (" gallbladder cancer*" OR "gall bladder cancer*" OR "biliary tract cancer*") AND NOT TITLE("gallbladder stone*")
Pancreatic / pancreas	("pancreatic neoplasms"[MeSH:noexp] OR "carcinoma, pancreatic ductal"[MeSH] OR "pancrea* cancer*"[tiab] OR "pancrea* carcinoma*"[tiab] OR "pancrea* adenocarcinoma*"[tiab] OR "pancrea* cancer*"[ot] OR "pancrea* carcinoma*"[ot] OR "pancrea* adenocarcinoma*"[ot]) NOT (pancreatectomy [ti])	('pancreas cancer'/de OR 'pancreas adenocarcinoma'/exp OR 'pancrea* cancer*':ti,ab,kw OR 'pancrea* carcinoma*':ti,ab,kw OR 'pancrea* adenocarcinoma*':ti,ab,kw) NOT ('pancreatectomy':ti)	TITLE-ABS-KEY ("pacrea* cancer*" OR "pancrea* carcinoma*" OR "pancrea* adenocarcinoma*" OR "pancrea* ductal") AND NOT TITLE("pancreatectomy")
Colorectal / colorectum	("colorectal neoplasms"[MeSH:noexp] OR "rectal neoplasms"[MeSH] OR "colorect* cancer*"[tiab] OR "colon cancer*"[tiab] OR "rectal cancer*"[tiab] OR "rectum cancer*"[tiab] OR "colorect* cancer*"[ot] OR "colon cancer*"[ot] OR "rectal cancer*"[ot] OR "rectum cancer*"[ot]) NOT ("adenoma*" [ti] OR "polyp*"[ti])	('colorectal cancer'/de OR 'rectum cancer'/exp OR 'colorect* cancer*':ti,ab,kw OR 'colon cancer*':ti,ab,kw OR 'rectal cancer*':ti,ab,kw OR 'rectum cancer*':ti,ab,kw) NOT ('adenoma*':ti OR 'polyp*':ti)	TITLE-ABS-KEY ("colorect* cancer*" OR "colon cancer*" OR "rectal cancer*" OR "rectum cancer*") AND NOT TITLE("adenoma*" OR "polyp*")

Cancer type	PubMed search terms	EMBASE search terms	Scopus search terms
Oesophagus / Esophagus: adenocarcinoma, squamous cell	"esophageal neoplasms"[MeSH] OR "esophageal squamous cell carcinoma"[MeSH] OR "esophag* cancer*" [tiab] OR "oesophag* cancer*"[tiab] OR "esophag* adenocarcinoma*" [tiab] OR "oesophag* adenocarcinoma*"[tiab] OR "esophag* squamous cell carcinoma*" [tiab] OR "oesophag* squamous cell carcinoma*"[tiab] OR "esophag* cancer*" [ot] OR "oesophag* cancer*"[ot] OR "esophag* adenocarcinoma*"[ot] OR "oesophag* adenocarcinoma*"[ot] OR "esophag* squamous cell carcinoma*"[ot] OR "oesophag* squamous cell carcinoma*"[ot]	esophagus cancer'/exp OR 'esophageal squamous cell carcinoma'/exp OR 'esophag* cancer*':ti,ab,kw OR 'oesophag* cancer*':ti,ab,kw OR 'esophag* adenocarcinoma*':ti,ab,kw OR 'oesophag* adenocarcinoma*':ti,ab,kw OR 'esophag* squamous cell carcinoma*':ti,ab,kw OR 'oesophag* squamous cell carcinoma*':ti,ab,kw	TITLE-ABS-KEY ("esophag* cancer*" OR "oesophag* cancer*" OR "esophag* adenocarcinoma*" OR "oesophag* adenocarcinoma*" OR "esophag* squamous cell carcinoma*" OR "oesophag* squamous cell carcinoma*")
Head and neck	"Nose Neoplasms"[Mesh] OR "Mouth Neoplasms"[Mesh] OR "Laryngeal Neoplasms"[Mesh] OR "Pharyngeal Neoplasms"[Mesh] OR "nose cancer*"[tiab] OR "nasal cancer*"[tiab] OR "mouth cancer*"[tiab] OR "oral cancer*"[tiab] OR "laryn* cancer*"[tiab] OR "pharyn* cancer*"[tiab] OR "nose cancer*"[ot] OR "nasal cancer*"[ot] OR "mouth cancer*"[ot] OR "oral cancer*"[ot] OR "laryn* cancer*"[ot] OR "pharyn* cancer*"[ot] OR "Head and Neck Neoplasms"[Mesh:noexp] OR "Head Neck cancer"[tiab:~1] OR "Head Neck cancers"[tiab:~1]	nose cancer'/exp OR 'mouth cancer'/exp OR 'larynx cancer'/exp OR 'pharynx cancer'/exp OR 'nose cancer*':ti,ab,kw OR 'nasal cancer*':ti,ab,kw OR 'mouth cancer*':ti,ab,kw OR 'oral cancer*':ti,ab,kw OR 'laryn* cancer*':ti,ab,kw OR 'pharyn* cancer*':ti,ab,kw OR 'head neck cancer*':ti,ab	TITLE-ABS-KEY ("nose cancer*" OR "nasal cancer*" OR "mouth cancer*" OR "oral cancer*" OR "laryn* cancer*" OR "pharyn* cancer*") OR TITLE-ABS(head W/2 neck W/2 cancer*)
Breast: pre-, post-menopausal	("breast neoplasms"[MeSH:noexp] OR "breast cancer*"[tiab] OR "breast cancer*"[ot]) AND ("premenopaus*" OR "postmenopau*" OR "pre-menopaus*" OR "post-menopaus*" OR "menopaus*")	('breast cancer'/de OR 'breast cancer*':ti,ab,kw) AND ('premenopaus*' OR 'postmenopau*' OR 'pre-menopaus*' OR 'post-menopaus*' OR 'menopaus*')	TITLE-ABS-KEY ("breast cancer*") AND ("premenopaus*" OR "postmenopau*" OR "pre-menopaus*" OR "post-menopaus*" OR "menopaus*")
Prostatic / Prostate: aggressive	("prostatic neoplasms"[MeSH] OR "prostat* cancer*"[tiab] OR "prostat* cancer*"[ot]) AND ("aggressive*" OR "lethal" OR "fatal" OR "death" OR "mortality" OR "advanced" OR "metastatic")	('prostate cancer'/exp OR 'prostat* cancer*':ti,ab,kw) AND ('aggressive*' OR 'lethal' OR 'fatal' OR 'death' OR 'mortality' OR 'advanced' OR 'metastatic')	TITLE-ABS-KEY ("prostat* cancer*") AND ("aggressive*" OR "lethal" OR "fatal" OR "death" OR "mortality" OR "advanced" OR "metastatic")

Supplementary Table 2: Criteria for assessing key confounders for each cancer type

Cancer type	Risk factors with strong associations	Reason for exclusion	Confounders for bias check
Endometrial	Age<br>Endogenous oestrogens<br>Oestrogen-only therapy	 Mediator<br>Uncommon among women with intact uterus	Age
Oesophageal (adenocarcinoma)	Age<br>Sex<br>Smoking<br>Race/ethnicity<br>GERD	 <br><br><br>Mediator	Age, sex, smoking, race/ethnicity*
Kidney	Age<br>Smoking<br>Hypertension	 <br>Mediator	Age, smoking
Gallbladder	Age<br>Gallstones<br>Liver flukes	 Mediator<br>Low prevalence in regions examined, incl. in Asia	Age
Gastric (cardia)	Age<br>Sex<br>GERD<br>Barrett's oesophagus	 <br>Mediator<br>Mediator	Age, sex
Liver cancer	Age<br>Race/ethnicity<br>Hepatitis C virus, Hepatitis B virus<br>Alcohol	 <br>Not associated with BMI<br>Not associated with BMI	Age, race/ethnicity*
Breast (postmenopausal)	Age<br>Endogenous oestrogens<br>Benign breast disease and other precursors<br>Nulliparity / older age at menopause<br>Older age at first live birth	 Mediator<br>Mediator<br>Not strongly associated with BMI<br>Confounding in opposite direction - older age at first birth is associated with lower BMI[33]	Age
Thyroid	Age<br>Sex<br>Goiter<br>Genetic susceptibilities (e.g., acromegaly)<br>Ionizing radiation	 <br>Mediator<br>Low prevalence<br>Not associated with BMI	Age, sex
Meningioma	Age<br>Central nervous system exposure to radiation therapy	 Not associated with BMI	Age
Colorectal	Age		Age
	Inflammatory bowel disease	Not strongly associated with BMI and possible mediator	

Multiple myeloma	Age<br>Race / ethnicity<br>MGUS and other precursors	 <br>Mediator	Age, race/ethnicity*
Leukaemia	Age<br>Ionizing radiation<br>Severe immune dysregulation	 Not associated with BMI<br>Low prevalence	Age
Head and neck	Age<br>Sex<br>Smoking<br>Smokeless tobacco<br>Alcohol<br>Human papillomavirus	 <br><br>Low prevalence and no clear association with BMI<br>Not associated with BMI<br>Not associated with BMI	Age, sex, smoking
Pancreas	Age<br>Smoking<br>Chronic pancreatitis<br>Diabetes<br>ABO blood type	 <br>Mediator<br>Mediator<br>Weak evidence for an association with BMI	Age, smoking
Ovarian	Age		Age
NHL	Age<br>Epstein-Barr virus<br>Human immunodeficiency virus	Age<br>Practically ubiquitous—and no strong evidence for associations with BMI[34]<br>Lower than 1% prevalence in each of N. America, Europe, and Asia	Age
Cervix	Age<br>Human papillomavirus	 Not associated with BMI[35]	Age
Prostate (aggressive)	Age		Age
Bladder	Age<br>Sex<br>Smoking<br>*Schistosoma haematobium*<br>Occupational exposures	 <br><br>Lower than 1% prevalence in each of N. America, Europe, and Asia<br>Low prevalence	Age, sex, smoking
Glioma	Age<br>Central nervous system exposure to radiation therapy	 No association with BMI	Age
Melanoma	Age<br>Race/ethnicity<br>Sun exposure	 <br>No consistent association with BMI	Age. race/ethnicity*
Gastric non-cardia	Age<br>*H. pylori*<br>Gastritis	 Not associated with BMI[36,37]<br>Mediator	Age
Lung	Age<br>Smoking<br>Occupational exposures (e.g. asbestos)	 <br>Low prevalence	Age, smoking

	Radon	Not strongly associated with BMI[38]	
	Indoor air pollution	Low prevalence (common in rural China, but included Chinese studies are urban)	
Breast (premenopausal)	Age<br>Endogenous oestrogens	Mediator	Age
Oesophageal squamous cell carcinoma	Age<br>Smoking<br>Race/ethnicity		Age, smoking, race/ethnicity*
	High dose ionizing radiation (>35 Gy)	Low prevalence	
	Alcohol	No strong association with BMI	
	Betel nut chewing	Low prevalence in regions of interest	
	Exceptionally hot beverages	Low prevalence in regions of interest	

*Race/ethnicity criterion applied only to cohorts with >80% racial diversity. All non-US cohorts where race was a potentially key confounder were >90% racially homogenous.

Abbreviations: BMI=body mass index, GERD=gastro-oesophageal reflux disease, MGUS=monoclonal gammopathy of undetermined significance, NHL=non-Hodgkin's Lymphoma, RR=risk ratio

Supplementary Figures

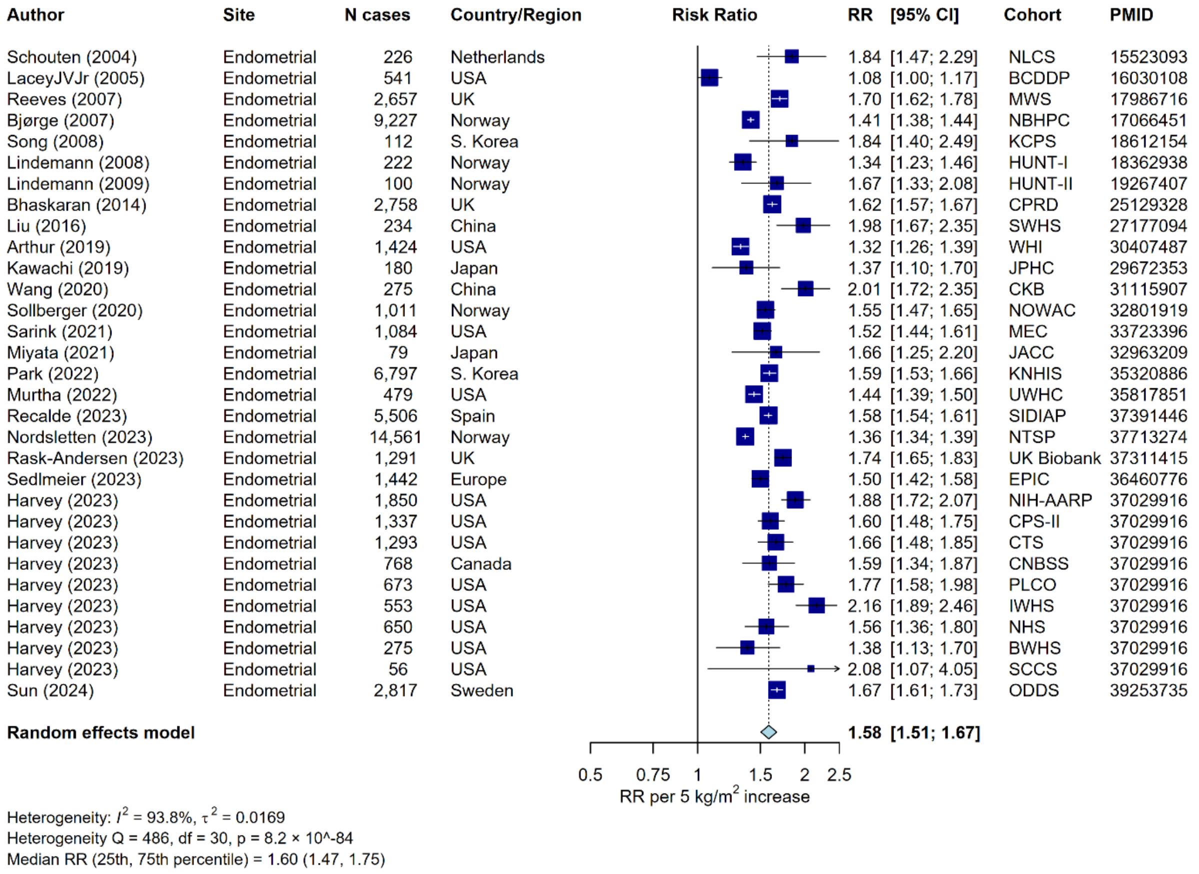

Supplementary Figure 1: Meta-analysis of prospective studies on the risk of endometrial cancer in relation to BMI

Study-specific RRs are represented by squares (with their 95% CIs as lines). RRs were combined using weighted averages of the log RRs in the separate studies, with weights assigned based on within and between study variance, yielding a pooled risk estimate and its 95% CI (diamond). Heterogeneity across studies was assessed using I^2, tau^2, and the 25th and 75th percentile of the RRs. Further details of model adjustments, follow-up time, analytic population for each study are available from **Supplementary Data**.

Abbreviations: BMI=body mass index, CI=confidence interval, PMID=PubMed ID, RR=risk ratio

Author	Site	N cases	Country/Region	RR	[95% CI]	Cohort	PMID
Engeland (2004)	Oesophageal (adeno)	575	Norway	1.56	[1.39; 1.75]	NBHPC	15456997
Lindblad (2005)	Oesophageal (adeno)	187	UK	1.38	[1.12; 1.71]	CPRD	15947880
Merry (2007)	Oesophageal (adeno)	133	Netherlands	1.93	[1.47; 2.59]	NLCS	17337464
Corley (2008)	Oesophageal (adeno)	101	USA	1.61	[1.22; 2.19]	KPNC	18268119
Petrick (2017)	Oesophageal (adeno)	433	USA	1.40	[1.28; 1.54]	NIH-AARP	28196067
Petrick (2017)	Oesophageal (adeno)	171	USA	1.54	[1.28; 1.76]	PLCO	28196067
Sanikini (2020)	Oesophageal (adeno)	220	Europe	1.37	[1.17; 1.61]	EPIC	31050823
Sweetland (2023)	Oesophageal (adeno)	1,386	UK	1.48	[1.40; 1.57]	MWS	37437897
Rask-Andersen (2023)	Oesophageal (adeno)	730	UK	1.51	[1.38; 1.66]	UK Biobank	37311415
Koyanagi (2023)	Oesophageal (adeno)	86	Japan	1.01	[0.69; 1.48]	Japan cohorts (10)	37013939
Sun (2024)	Oesophageal (adeno)	1,193	Sweden	1.61	[1.49; 1.74]	ODDS	39253735
Saeed (2025)	Oesophageal (adeno)	1,522	Norway	1.33	[1.23; 1.43]	NTSP	40091197
**Random effects model**				**1.47**	**[1.39; 1.56]**		

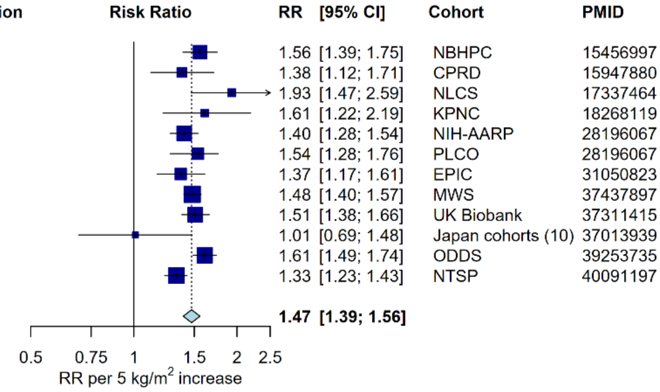

Heterogeneity: $I^2 = 53.1\%$, $\tau^2 = 0.0049$
Heterogeneity Q = 23, df = 11, p = 0.02
Median RR (25th, 75th percentile) = 1.50 (1.38, 1.57)

Supplementary Figure 2: Meta-analysis of prospective studies on the risk of oesophageal adenocarcinoma cancer in relation to BMI

Study-specific RRs are represented by squares (with their 95% CIs as lines). RRs were combined using weighted averages of the log RRs in the separate studies, with weights assigned based on within and between study variance, yielding a pooled risk estimate and its 95% CI (diamond). Heterogeneity across studies was assessed using I^2, tau^2, and the 25th and 75th percentile of the RRs. Further details of model adjustments, follow-up time, analytic population for each study are available from **Supplementary Data**.

Abbreviations: BMI=body mass index, CI=confidence interval, PMID=PubMed ID, RR=risk ratio

Author	Site	N cases	Country/Region	RR	[95% CI]	Cohort	PMID
Bjørge (2004)	Renal cell carcinoma	6,453	Norway	1.28	[1.23; 1.32]	NBHPC	15583369
vanDijk (2004)	Renal cell carcinoma	275	Netherlands	1.40	[1.10; 1.76]	NLCS	15583368
Reeves (2007)	Kidney	723	UK	1.24	[1.13; 1.36]	MWS	17986716
Setiawan (2007)	Renal cell carcinoma	347	USA	1.34	[1.18; 1.54]	MEC	17656615
Adams (2008)	Renal cell carcinoma	1,366	USA	1.37	[1.29; 1.47]	NIH-AARP	18544571
Sawada (2010)	Kidney	139	Japan	1.17	[0.88; 1.56]	JPHC	20470974
Andreotti (2010)	Kidney	106	USA	1.05	[0.81; 1.37]	AgHealth	20730623
Macleod (2013)	Renal cell carcinoma	238	USA	1.18	[0.99; 1.40]	VITAL	23665301
Bhaskaran (2014)	Kidney	1,906	UK	1.25	[1.19; 1.31]	CPRD	25129328
Sanfilippo (2014)	Kidney	407	USA	1.22	[1.11; 1.33]	WHI	24637660
Guo (2014)	Kidney	69	China	1.28	[0.91; 1.80]	Kailun Cohort	24831616
Song (2014)	Kidney	195	Finland	1.34	[1.10; 1.63]	FINRISK	24997743
Liu (2016)	Renal cell carcinoma	131	China	1.26	[0.97; 1.63]	SWHS	27177094
daSilva (2018)	Kidney	211	Norway	1.33	[1.15; 1.54]	NOWAC	30202086
Nam (2019)	Kidney	18,036	S. Korea	1.34	[1.31; 1.37]	KNHIS	31231120
Everatt (2019)	Kidney	79	Lithuania	1.40	[1.05; 1.93]	KRIS, MIHDPS	31951685
Murtha (2022)	Kidney	478	USA	1.22	[1.16; 1.29]	UWHC	35817851
Graff (2022)	Renal cell carcinoma	394	USA	1.41	[1.25; 1.59]	NHS	35715363
Graff (2022)	Renal cell carcinoma	110	USA	1.45	[1.19; 1.75]	NHS-II	35715363
Graff (2022)	Renal cell carcinoma	291	USA	1.28	[1.05; 1.56]	HPFS	35715363
Recalde (2023)	Kidney	7,065	Spain	1.15	[1.12; 1.19]	SIDIAP	37391446
Nordsletten (2023)	Kidney	13,133	Norway	1.25	[1.22; 1.28]	NTSP	37713274
Rask-Andersen (2023)	Kidney	1,462	UK	1.37	[1.29; 1.45]	UK Biobank	37311415
Sedlmeier (2023)	Kidney	899	Europe	1.31	[1.21; 1.42]	EPIC	36460776
Deng (2023)	Renal cell carcinoma	527	USA	1.37	[1.26; 1.49]	PLCO	37624040
Sun (2024)	Renal cell carcinoma	6,193	Sweden	1.45	[1.40; 1.50]	ODDS	39253735
**Random effects model**				**1.30**	**[1.26; 1.33]**		

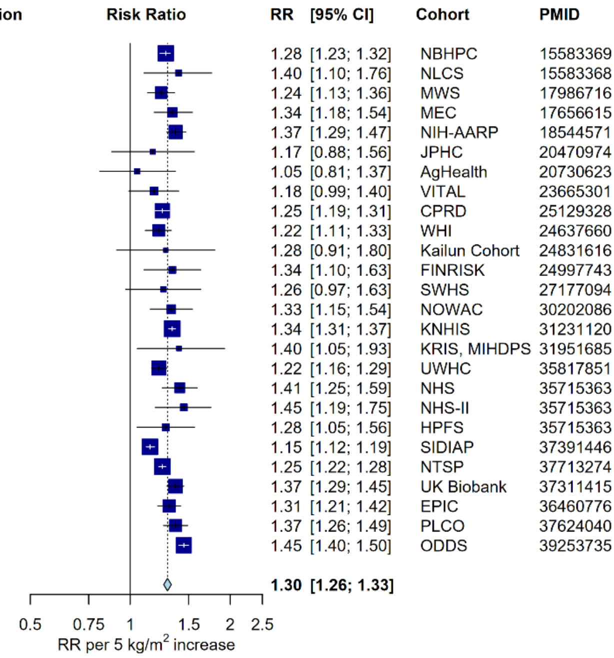

Heterogeneity: I^2 = 83.0%, τ^2 = 0.0021
Heterogeneity Q = 147, df = 25, p = 3.0 × 10^-19
Median RR (25th, 75th percentile) = 1.29 (1.24, 1.37)

Supplementary Figure 3: Meta-analysis of prospective studies on the risk of kidney cancer in relation to BMI

Study-specific RRs are represented by squares (with their 95% CIs as lines). RRs were combined using weighted averages of the log RRs in the separate studies, with weights assigned based on within and between study variance, yielding a pooled risk estimate and its 95% CI (diamond). Heterogeneity across studies was assessed using I^2, tau^2, and the 25th and 75th percentile of the RRs. Further details of model adjustments, follow-up time, analytic population for each study are available from **Supplementary Data**.

Abbreviations: BMI=body mass index, CI=confidence interval, PMID=PubMed ID, RR=risk ratio

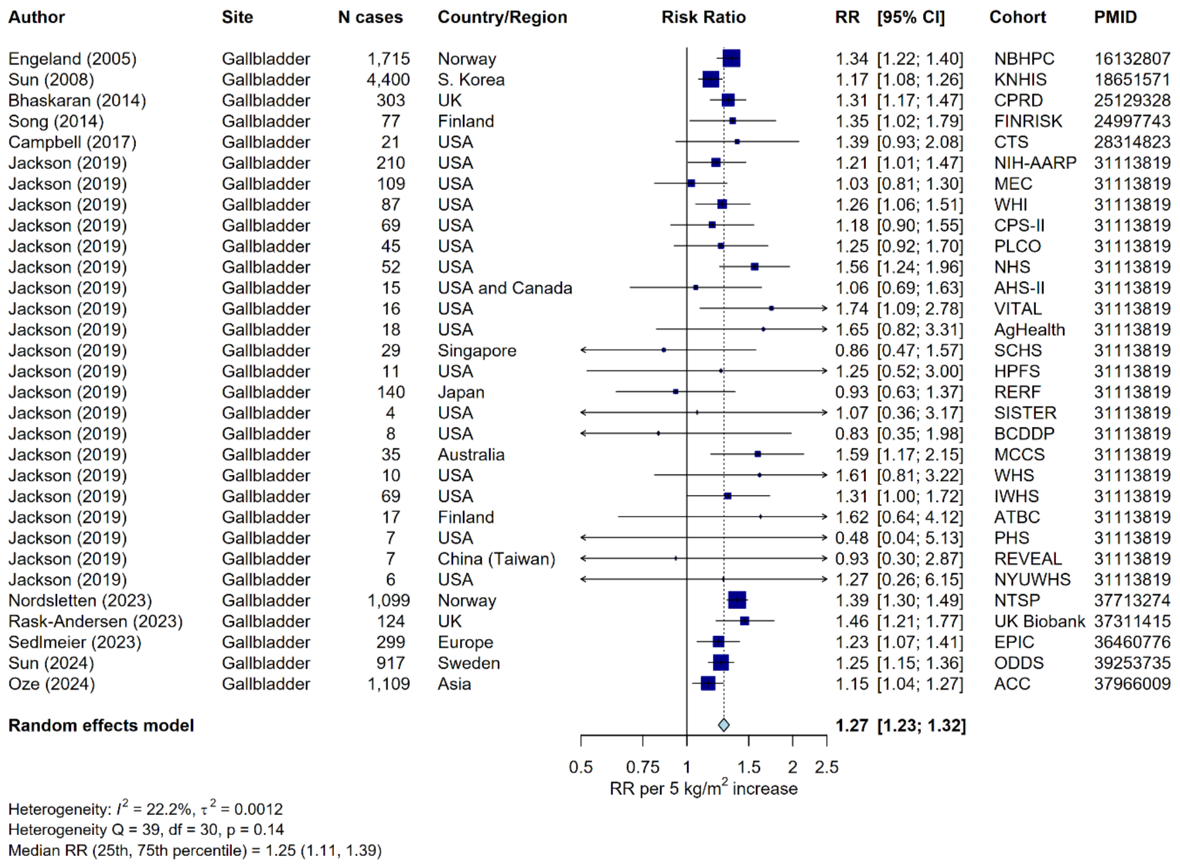

Supplementary Figure 4: Meta-analysis of prospective studies on the risk of gallbladder cancer in relation to BMI

Study-specific RRs are represented by squares (with their 95% CIs as lines). RRs were combined using weighted averages of the log RRs in the separate studies, with weights assigned based on within and between study variance, yielding a pooled risk estimate and its 95% CI (diamond). Heterogeneity across studies was assessed using I^2, tau^2, and the 25th and 75th percentile of the RRs. Further details of model adjustments, follow-up time, analytic population for each study are available from **Supplementary Data**.

Abbreviations: BMI=body mass index, CI=confidence interval, PMID=PubMed ID, RR=risk ratio

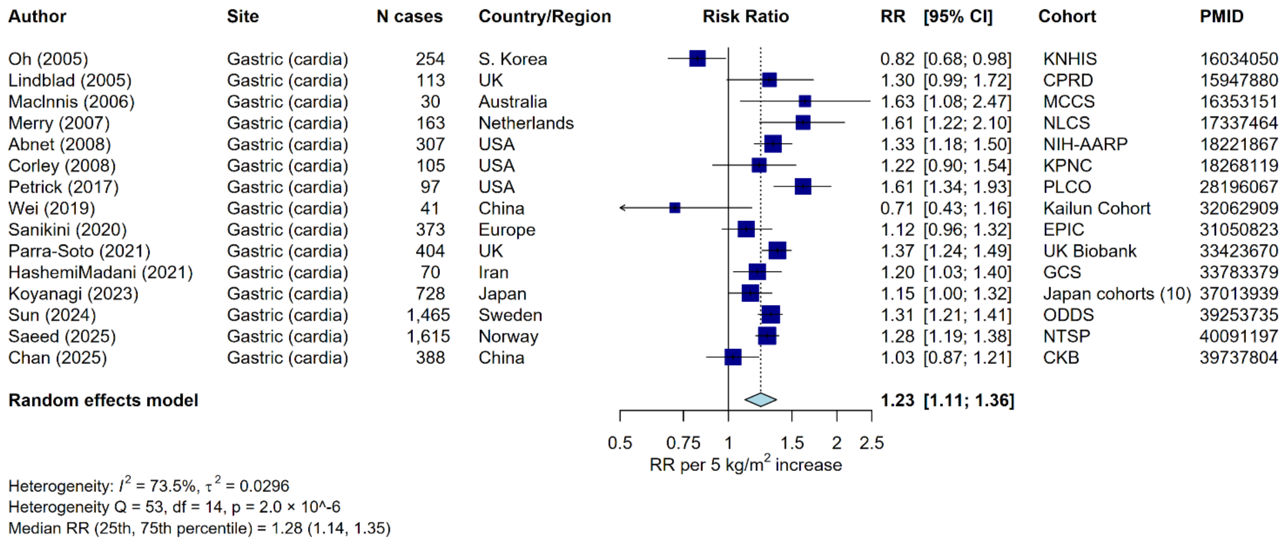

Supplementary Figure 5: Meta-analysis of prospective studies on the risk of gastric cancer (cardia) in relation to BMI

Study-specific RRs are represented by squares (with their 95% CIs as lines). RRs were combined using weighted averages of the log RRs in the separate studies, with weights assigned based on within and between study variance, yielding a pooled risk estimate and its 95% CI (diamond). Heterogeneity across studies was assessed using I^2, tau^2, and the 25th and 75th percentile of the RRs. Further details of model adjustments, follow-up time, analytic population for each study are available from **Supplementary Data**.

Abbreviations: BMI=body mass index, CI=confidence interval, PMID=PubMed ID, RR=risk ratio

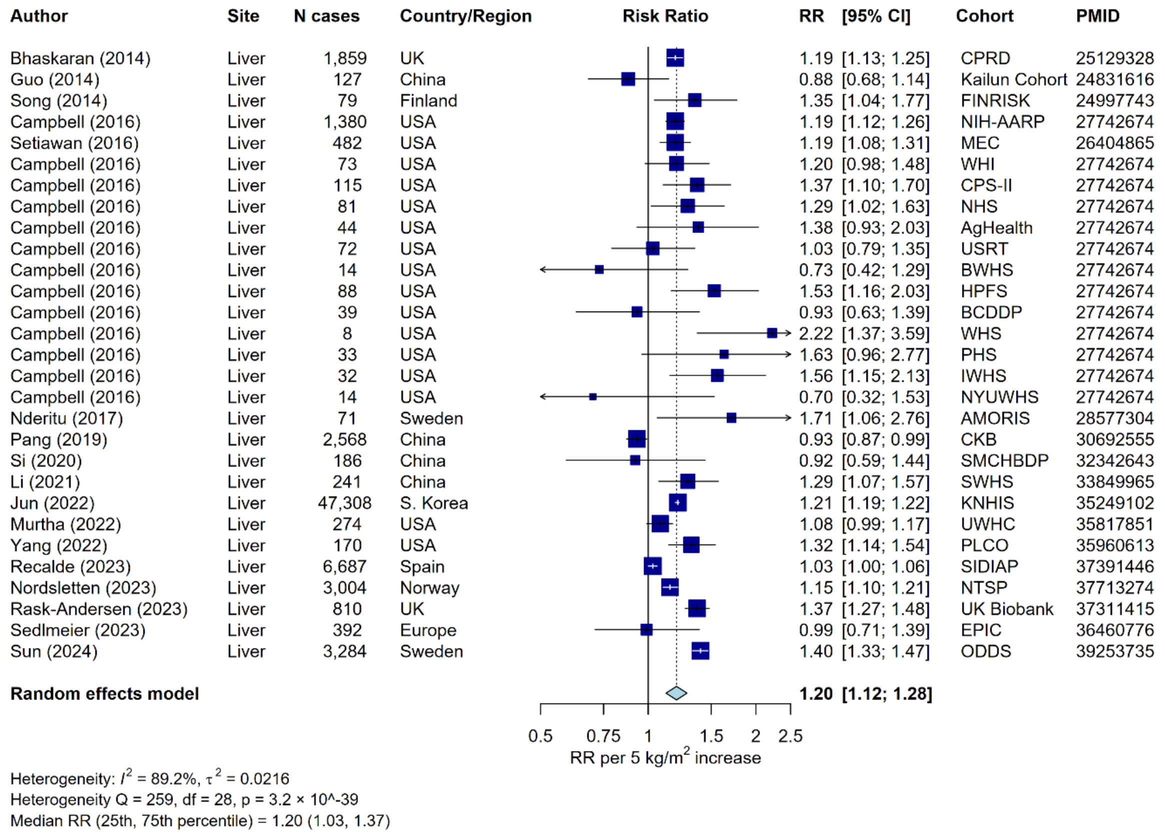

Supplementary Figure 6: Meta-analysis of prospective studies on the risk of liver cancer in relation to BMI

Study-specific RRs are represented by squares (with their 95% CIs as lines). RRs were combined using weighted averages of the log RRs in the separate studies, with weights assigned based on within and between study variance, yielding a pooled risk estimate and its 95% CI (diamond). Heterogeneity across studies was assessed using I^2, tau^2, and the 25th and 75th percentile of the RRs. Further details of model adjustments, follow-up time, analytic population for each study are available from **Supplementary Data**.

Abbreviations: BMI=body mass index, CI=confidence interval, PMID=PubMed ID, RR=risk ratio

Author	Site	N cases	Country/Region	RR	[95% CI]	Cohort	PMID
Vatten (1990)	Breast (postmenopausal)	99	Norway	0.90	[0.68; 1.18]	NCS	2307535
van den Brandt (2000)	Breast (postmenopausal)	420	Netherlands	0.99	[0.83; 1.17]	NLCS	10997541
van den Brandt (2000)	Breast (postmenopausal)	242	Canada	1.14	[0.89; 1.47]	CNBSS	10997541
van den Brandt (2000)	Breast (postmenopausal)	643	USA	1.14	[1.05; 1.24]	IWHS	10997541
van den Brandt (2000)	Breast (postmenopausal)	358	USA	1.05	[0.94; 1.19]	NYSC	10997541
van den Brandt (2000)	Breast (postmenopausal)	87	USA	1.23	[0.96; 1.58]	AHS-I	10997541
Feigelson (2004)	Breast (postmenopausal)	1,934	USA	1.10	[1.05; 1.15]	CPS-II	14973094
Tehard (2006)	Breast (postmenopausal)	1,037	France	1.15	[1.02; 1.30]	E3N	16231021
Reinier (2007)	Breast (postmenopausal)	572	USA	1.38	[1.23; 1.53]	VMC	17063272
Lacey (2009)	Breast (postmenopausal)	2,063	USA	1.06	[1.02; 1.10]	PLCO	19292893
Andreotti (2010)	Breast (postmenopausal)	464	USA	1.16	[1.05; 1.34]	AgHealth	20730623
Sczaniecka (2012)	Breast (postmenopausal)	741	USA	1.06	[0.98; 1.16]	VITAL	23137008
Bhaskaran (2014)	Breast (postmenopausal)	28,409	UK	1.05	[1.03; 1.07]	CPRD	25129328
Guo (2014)	Breast (postmenopausal)	57	China	1.32	[0.95; 1.83]	Kailun Cohort	24831616
Catsburg (2014)	Breast (postmenopausal)	541	Canada	1.13	[1.00; 1.29]	CSDLH	24781974
Heo (2015)	Breast (postmenopausal)	6,798	USA	1.10	[1.09; 1.13]	WHI	25587642
White (2015)	Breast (postmenopausal)	1,582	USA	1.10	[1.05; 1.16]	SISTER	26193782
Chen (2016)	Breast (postmenopausal)	6,023	China (Taiwan)	1.21	[1.17; 1.25]	TBCSP	26443343
Freisling (2017)	Breast (postmenopausal)	125	Germany	1.19	[0.99; 1.44]	ESTHER	28441380
Freisling (2017)	Breast (postmenopausal)	22	Greece	1.15	[0.73; 1.82]	EPIC (Greece)	28441380
Freisling (2017)	Breast (postmenopausal)	64	Norway	1.32	[0.96; 1.84]	Tromso	28441380
Cifu (2018)	Breast (postmenopausal)	7,095	USA	1.08	[1.05; 1.11]	NIH-AARP	30309689
daSilva (2018)	Breast (postmenopausal)	1,336	Norway	1.07	[1.00; 1.15]	NOWAC	30202086
Sandvei (2019)	Breast (postmenopausal)	2,080	Norway	1.10	[1.10; 1.16]	NBCSP	30083811
Bissell (2020)	Breast (postmenopausal)	14,357	USA	1.17	[1.16; 1.18]	BCSC	32727722
Wang (2020)	Breast (postmenopausal)	1,202	China	1.29	[1.18; 1.40]	CKB	31115907
Renehan (2020)	Breast (postmenopausal)	829	UK	1.24	[1.15; 1.34]	PROCAS	32203222
Park (2021)	Breast (postmenopausal)	39,485	S. Korea	1.23	[1.21; 1.25]	KNHIS	33010023
Houghton (2021)	Breast (postmenopausal)	2,909	USA	1.20	[1.13; 1.28]	NHS, NHS-II	33367714
Yang (2021)	Breast (postmenopausal)	789	Australia	1.16	[1.09; 1.24]	MCCS	33268487
Yang (2022)	Breast (postmenopausal)	15,506	UK	1.20	[1.18; 1.22]	MWS	35255844
Recalde (2023)	Breast (postmenopausal)	28,178	Spain	1.08	[1.06; 1.09]	SIDIAP	37391446
Sedlmeier (2023)	Breast (postmenopausal)	4,988	Europe	1.21	[1.12; 1.31]	EPIC (not Greece)	36460776
Gram (2023)	Breast (postmenopausal)	1,919	Denmark	1.10	[1.04; 1.15]	DCHS	36750141
Sun (2024)	Breast (postmenopausal)	19,296	Sweden	1.06	[1.04; 1.08]	ODDS	39253735
Lu (2024)	Breast (postmenopausal)	6,956	UK	1.12	[1.09; 1.15]	UK Biobank	39150790
Wada (2024)	Breast (postmenopausal)	1,685	Japan	1.33	[1.24; 1.42]	ACC (Japan)	39543702
Wada (2024)	Breast (postmenopausal)	759	China	1.33	[1.21; 1.47]	SWHS	39543702
Wada (2024)	Breast (postmenopausal)	173	S. Korea	1.05	[0.82; 1.34]	ACC (Korea)	39543702
Ogbenna (2025)	Breast (postmenopausal)	4,555	USA	1.13	[1.09; 1.17]	MEC	40112020
**Random effects model**				**1.14**	**[1.12; 1.17]**		

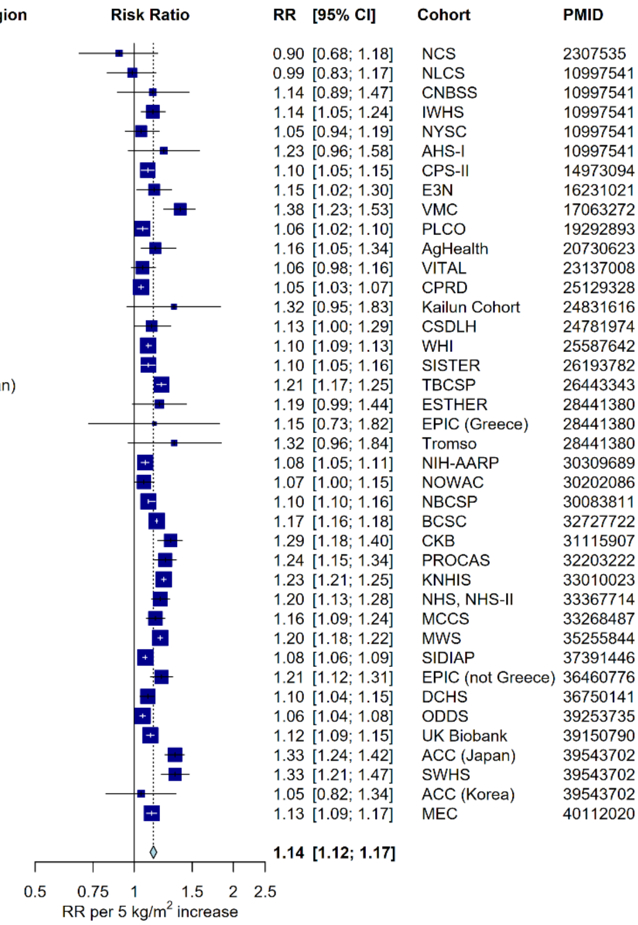

Heterogeneity: I^2 = 91.9%, τ^2 = 0.0037
Heterogeneity Q = 479, df = 39, p = 4.1 × 10^-77
Median RR (25th, 75th percentile) = 1.14 (1.08, 1.21)

Supplementary Figure 7: Meta-analysis of prospective studies on the risk of breast (postmenopausal) cancer in relation to BMI

Study-specific RRs are represented by squares (with their 95% CIs as lines). RRs were combined using weighted averages of the log RRs in the separate studies, with weights assigned based on within and between study variance, yielding a pooled risk estimate and its 95% CI (diamond). Heterogeneity across studies was assessed using I^2, tau^2, and the 25th and 75th percentile of the RRs. Further details of model adjustments, follow-up time, analytic population for each study are available from **Supplementary Data**.

Abbreviations: BMI=body mass index, CI=confidence interval, PMID=PubMed ID, RR=risk ratio

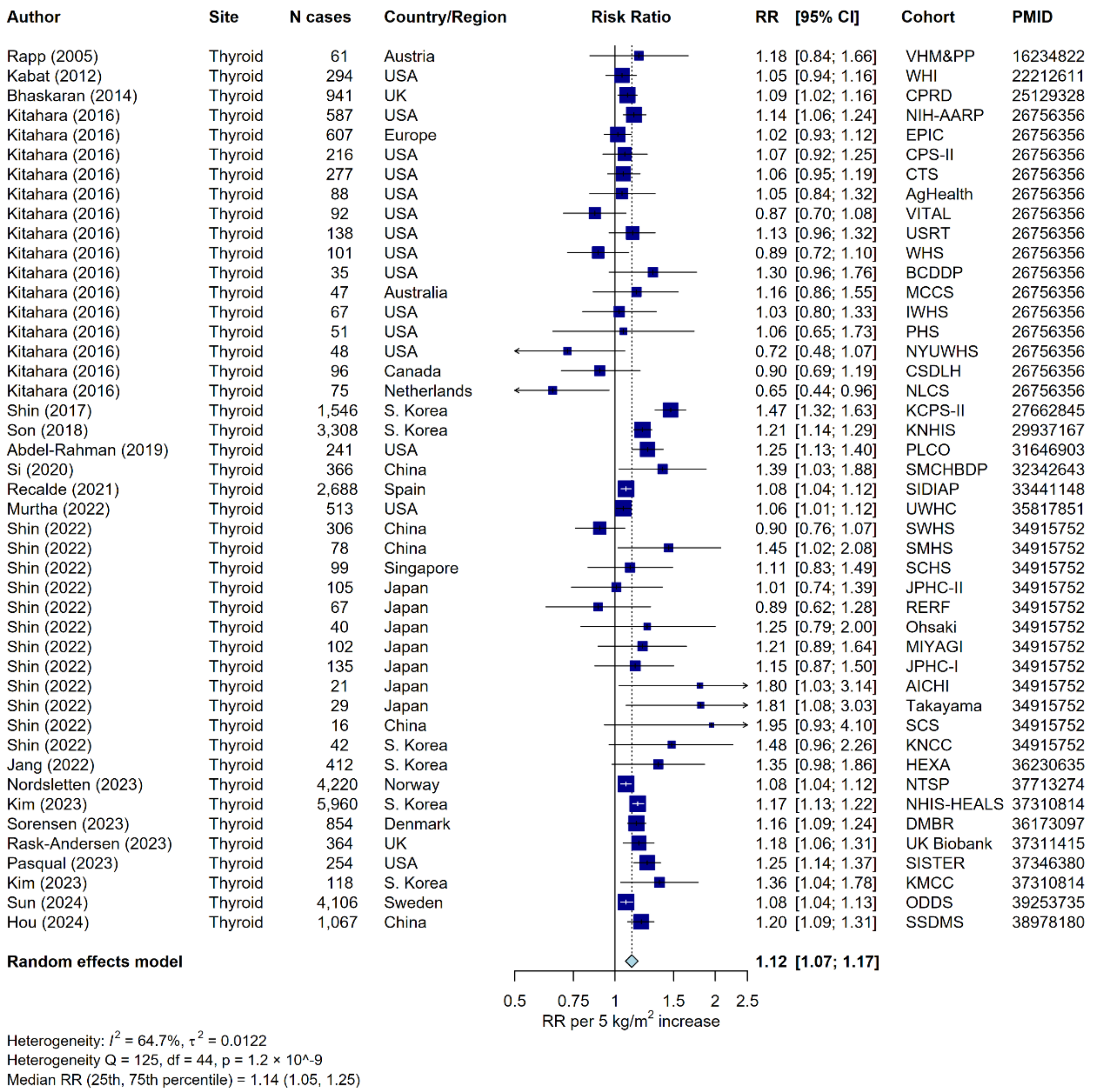

Supplementary Figure 8: Meta-analysis of prospective studies on the risk of thyroid cancer in relation to BMI

Study-specific RRs are represented by squares (with their 95% CIs as lines). RRs were combined using weighted averages of the log RRs in the separate studies, with weights assigned based on within and between study variance, yielding a pooled risk estimate and its 95% CI (diamond). Heterogeneity across studies was assessed using I^2, tau^2, and the 25th and 75th percentile of the RRs. Further details of model adjustments, follow-up time, analytic population for each study are available from **Supplementary Data**.

Abbreviations: BMI=body mass index, CI=confidence interval, PMID=PubMed ID, RR=risk ratio

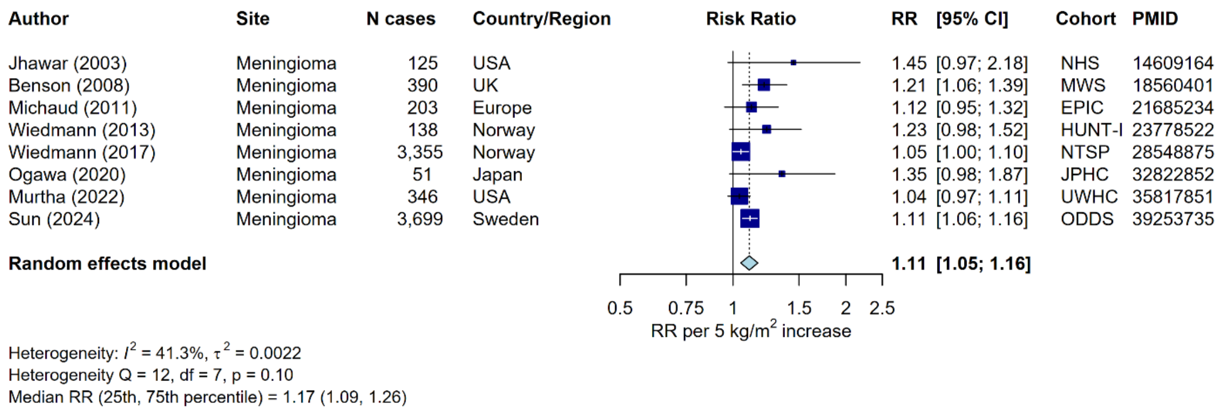

Supplementary Figure 9: Meta-analysis of prospective studies on the risk of meningioma in relation to BMI

Study-specific RRs are represented by squares (with their 95% CIs as lines). RRs were combined using weighted averages of the log RRs in the separate studies, with weights assigned based on within and between study variance, yielding a pooled risk estimate and its 95% CI (diamond). Heterogeneity across studies was assessed using I^2, tau^2, and the 25th and 75th percentile of the RRs. Further details of model adjustments, follow-up time, analytic population for each study are available from **Supplementary Data**.

Abbreviations: BMI=body mass index, CI=confidence interval, PMID=PubMed ID, RR=risk ratio

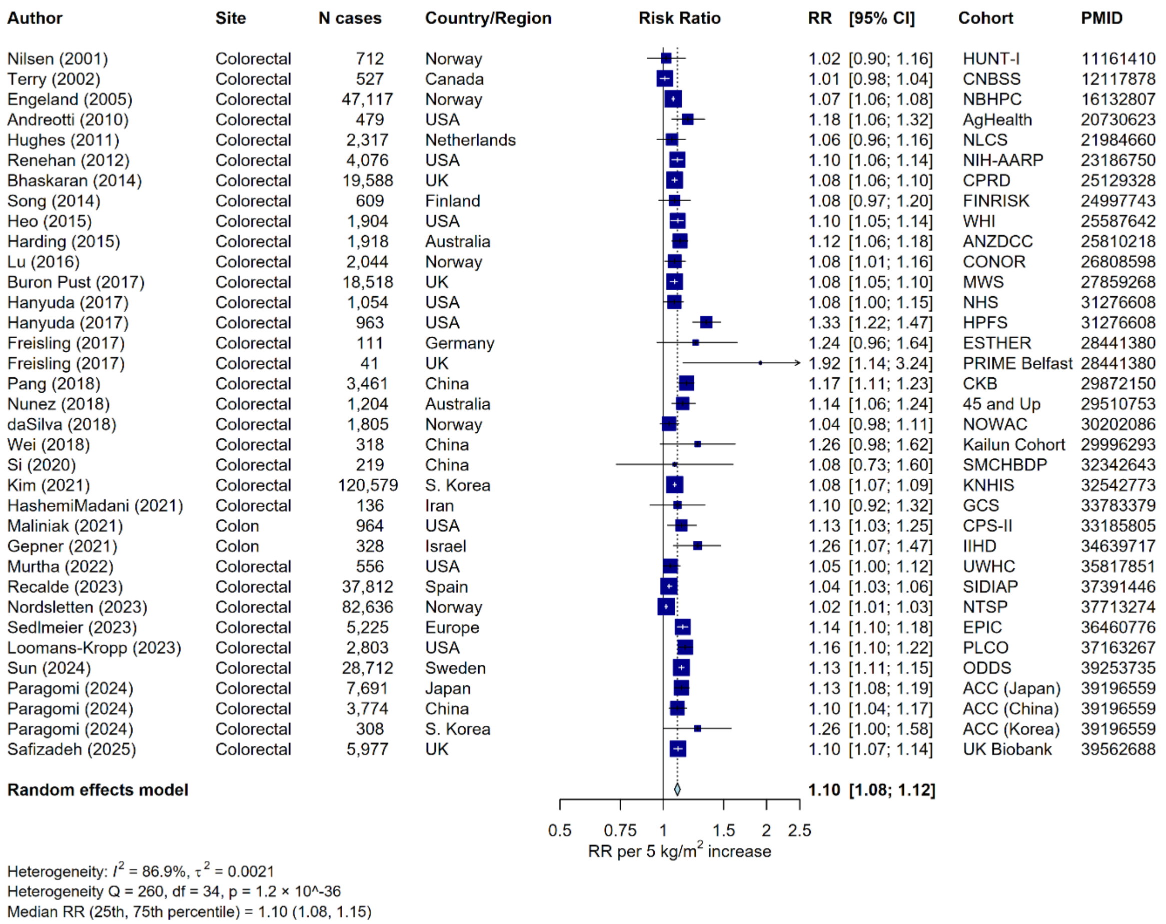

Supplementary Figure 10: Meta-analysis of prospective studies on the risk of colorectal cancer in relation to BMI

Study-specific RRs are represented by squares (with their 95% CIs as lines). RRs were combined using weighted averages of the log RRs in the separate studies, with weights assigned based on within and between study variance, yielding a pooled risk estimate and its 95% CI (diamond). Heterogeneity across studies was assessed using I^2, tau^2, and the 25th and 75th percentile of the RRs. Further details of model adjustments, follow-up time, analytic population for each study are available from **Supplementary Data**.

Abbreviations: BMI=body mass index, CI=confidence interval, PMID=PubMed ID, RR=risk ratio

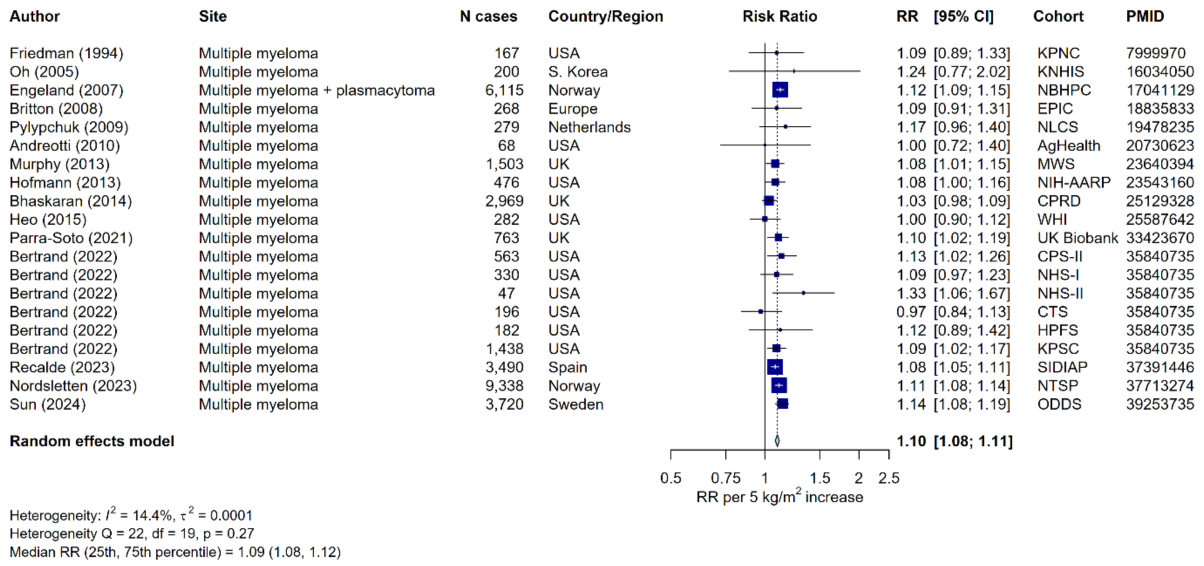

Supplementary Figure 11: Meta-analysis of prospective studies on the risk of multiple myeloma in relation to BMI

Study-specific RRs are represented by squares (with their 95% CIs as lines). RRs were combined using weighted averages of the log RRs in the separate studies, with weights assigned based on within and between study variance, yielding a pooled risk estimate and its 95% CI (diamond). Heterogeneity across studies was assessed using I^2, tau^2, and the 25th and 75th percentile of the RRs. Further details of model adjustments, follow-up time, analytic population for each study are available from **Supplementary Data**.

Abbreviations: BMI=body mass index, CI=confidence interval, PMID=PubMed ID, RR=risk ratio

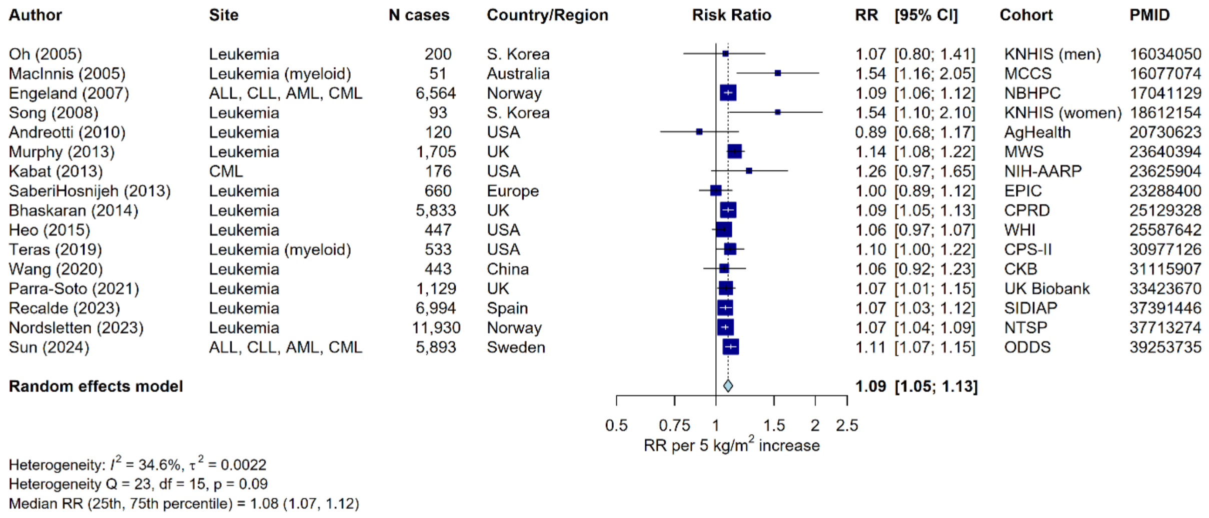

Supplementary Figure 12: Meta-analysis of prospective studies on the risk of leukaemia in relation to BMI

Study-specific RRs are represented by squares (with their 95% CIs as lines). RRs were combined using weighted averages of the log RRs in the separate studies, with weights assigned based on within and between study variance, yielding a pooled risk estimate and its 95% CI (diamond). Heterogeneity across studies was assessed using I^2, tau^2, and the 25th and 75th percentile of the RRs. Further details of model adjustments, follow-up time, analytic population for each study are available from **Supplementary Data**.

Abbreviations: ALL=acute lymphocytic leukaemia, AML=acute myeloid leukaemia, BMI=body mass index, CI=confidence interval, CLL=chronic lymphocytic leukaemia, CML=chronic myeloid leukaemia, PMID=PubMed ID, RR=risk ratio

Author	Site	N cases	Country/Region	RR	[95% CI]	Cohort	PMID
Bhaskaran (2014)	Head and neck (never-smokers)	302	UK	1.07	[0.95; 1.21]	CPRD	25129328
Gaudet (2015)	Head and neck (never-smokers)	NA	USA	1.09	[0.94; 1.25]	NIH-AARP	26050257
Gaudet (2015)	Head and neck (never-smokers)	NA	China	0.84	[0.49; 1.41]	SWHS	26050257
Gaudet (2015)	Head and neck (never-smokers)	NA	USA	1.39	[1.05; 1.83]	CTS	26050257
Gaudet (2015)	Head and neck (never-smokers)	NA	USA	1.03	[0.78; 1.35]	CPS-II	26050257
Gaudet (2015)	Head and neck (never-smokers)	NA	USA	1.28	[0.94; 1.75]	PLCO	26050257
Gaudet (2015)	Head and neck (never-smokers)	NA	USA	1.18	[0.70; 2.00]	USRT	26050257
Gaudet (2015)	Head and neck (never-smokers)	NA	USA	1.48	[0.99; 2.20]	VITAL	26050257
Gaudet (2015)	Head and neck (never-smokers)	NA	USA	1.74	[1.09; 2.78]	AgHealth	26050257
Gaudet (2015)	Head and neck (never-smokers)	NA	USA	0.75	[0.33; 1.70]	SISTER	26050257
Gaudet (2015)	Head and neck (never-smokers)	NA	USA	0.91	[0.66; 1.28]	IWHS	26050257
Gaudet (2015)	Head and neck (never-smokers)	NA	Australia	0.90	[0.57; 1.41]	MCCS	26050257
Gaudet (2015)	Head and neck (never-smokers)	NA	USA	0.84	[0.35; 1.97]	BCDDP	26050257
Gaudet (2015)	Head and neck (never-smokers)	NA	USA	1.12	[0.66; 1.89]	WHS	26050257
Gaudet (2015)	Head and neck (never-smokers)	NA	China	2.33	[0.64; 8.45]	SMHS	26050257
Gaudet (2015)	Head and neck (never-smokers)	NA	USA	1.43	[0.91; 2.27]	PHS	26050257
Gaudet (2015)	Head and neck (never-smokers)	NA	USA	0.69	[0.17; 2.82]	NYUWHS	26050257
Maasland (2015)	Head and neck (never-smokers)	55	Netherlands	1.00	[0.62; 1.54]	NLCS	26634678
Ward (2017)	Head and neck (never-smokers)	141	Europe	1.24	[0.98; 1.57]	EPIC	28183827
Recalde (2021)	Head and neck (never-smokers)	5,199	Spain	1.09	[1.04; 1.14]	SIDIAP	33441148
Kim (2022)	Head and neck (never-smokers)	4,273	S. Korea	0.96	[0.91; 1.01]	KNHIS	36010881
Sun (2024)	Head and neck (never-smokers)	990	Sweden	1.05	[0.95; 1.15]	ODDS	39253735
Suzuki (2025)	Head and neck (never-smokers)	116	Japan	1.05	[0.82; 1.33]	JPHC	39183034
**Random effects model**				**1.07**	**[1.02; 1.13]**		

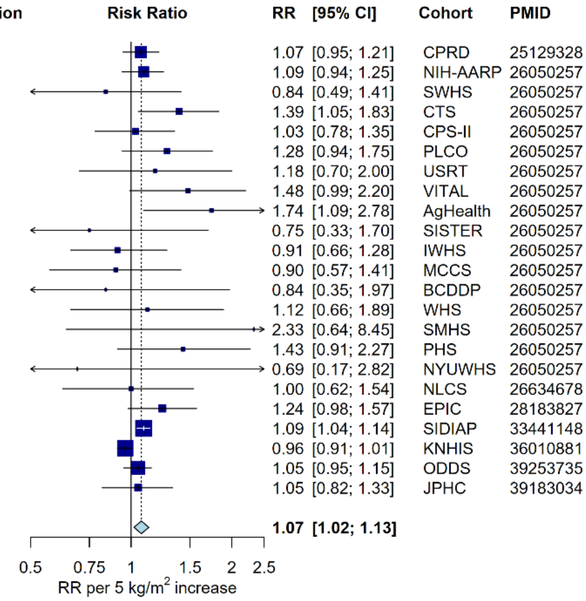

Heterogeneity: I^2 = 38.3%, τ^2 = 0.0028
Heterogeneity Q = 36, df = 22, p = 0.03
Median RR (25th, 75th percentile) = 1.07 (0.94, 1.26)

Supplementary Figure 13: Meta-analysis of prospective studies on the risk of head and neck cancer (restricted to never-smokers) in relation to BMI

Study-specific RRs are represented by squares (with their 95% CIs as lines). RRs were combined using weighted averages of the log RRs in the separate studies, with weights assigned based on within and between study variance, yielding a pooled risk estimate and its 95% CI (diamond). Heterogeneity across studies was assessed using I^2, tau^2, and the 25th and 75th percentile of the RRs. Further details of model adjustments, follow-up time, analytic population for each study are available from **Supplementary Data**. Cases of head and neck cancers for never-smokers were not available in individual studies (N cases total=796).

Abbreviations: BMI=body mass index, CI=confidence interval, PMID=PubMed ID, RR=risk ratio

Author	Site	N cases	Country/Region	RR	[95% CI]	Cohort	PMID
Verhage (2007)	Pancreas	423	Netherlands	1.23	[1.05; 1.45]	NLCS	17585056
Berrington de Gonzalez (2008)	Pancreas	2,194	S. Korea	0.99	[0.92; 1.06]	KCPS	18268120
Stevens (2009)	Pancreas	1,338	UK	1.09	[1.03; 1.16]	MWS	19165860
Jiao (2010)	Pancreas	137	USA	1.09	[0.89; 1.34]	USRT	20383573
Jiao (2010)	Pancreas	95	USA	1.08	[0.85; 1.37]	AgHealth	20383573
Jiao (2010)	Pancreas	124	USA	1.16	[0.96; 1.40]	BCDDP	20383573
Jiao (2010)	Pancreas	338	Finland	1.08	[0.93; 1.24]	ATBC	20383573
Arslan (2010)	Pancreas	83	USA	1.75	[1.02; 3.00]	CLUE-II	20458087
Arslan (2010)	Pancreas	40	USA	1.22	[0.64; 2.33]	WHS	20458087
Genkinger (2011)	Pancreas	369	USA	1.23	[1.09; 1.39]	CPS-II	21105029
Genkinger (2011)	Pancreas	103	USA	1.01	[0.82; 1.25]	CTS	21105029
Genkinger (2011)	Pancreas	173	USA	1.17	[1.01; 1.37]	NHS	21105029
Genkinger (2011)	Pancreas	135	USA	1.12	[0.89; 1.40]	NYSC	21105029
Genkinger (2011)	Pancreas	193	USA	1.10	[0.89; 1.38]	HPFS	21105029
Genkinger (2011)	Pancreas	63	Australia	1.01	[0.76; 1.35]	MCCS	21105029
Genkinger (2011)	Pancreas	171	USA	0.97	[0.82; 1.15]	IWHS	21105029
Bhaskaran (2014)	Pancreas	3,851	UK	1.05	[1.01; 1.09]	CPRD	25129328
Song (2014)	Pancreas	218	Finland	1.22	[1.01; 1.46]	FINRISK	24997743
Liu (2016)	Pancreas	195	China	0.92	[0.74; 1.14]	SWHS	27177094
Pang (2017)	Pancreas	595	China	1.11	[0.97; 1.27]	CKB	28900029
Koyanagi (2018)	Pancreas	1,593	Japan	1.02	[0.92; 1.13]	Japan cohorts (9)	29225297
daSilva (2018)	Pancreas	324	Norway	1.11	[0.96; 1.27]	NOWAC	30202086
Arthur (2019)	Pancreas	1,040	USA	1.08	[1.02; 1.14]	WHI	30449532
Farias (2021)	Pancreas	1,328	USA	1.10	[1.00; 1.16]	MEC	33998145
HashemiMadani (2021)	Pancreas	93	Iran	1.08	[0.87; 1.35]	GCS	33783379
Park (2022)	Pancreas	22,543	S. Korea	1.06	[1.04; 1.08]	KNHIS	35444288
Murtha (2022)	Pancreas	184	USA	1.00	[0.90; 1.11]	UWHC	35817851
Arjani (2022)	Pancreas	3,092	USA	1.09	[1.04; 1.13]	NIH-AARP	36255251
Hoyt (2022)	Pancreas	696	USA	1.00	[0.92; 1.09]	PLCO	36156459
Luu (2022)	Pancreas	316	Singapore	1.06	[0.84; 1.34]	SCHS	34642175
Recalde (2023)	Pancreas	6,592	Spain	0.98	[0.95; 1.01]	SIDIAP	37391446
Nordsletten (2023)	Pancreas	17,330	Norway	1.04	[1.02; 1.06]	NTSP	37713274
Rask-Andersen (2023)	Pancreas	1,302	UK	1.17	[1.10; 1.24]	UK Biobank	37311415
Sedlmeier (2023)	Pancreas	1,097	Europe	1.12	[1.03; 1.21]	EPIC	36460776
Sun (2024)	Pancreas	5,509	Sweden	1.11	[1.07; 1.16]	ODDS	39253735
**Random effects model**				**1.07**	**[1.05; 1.09]**		

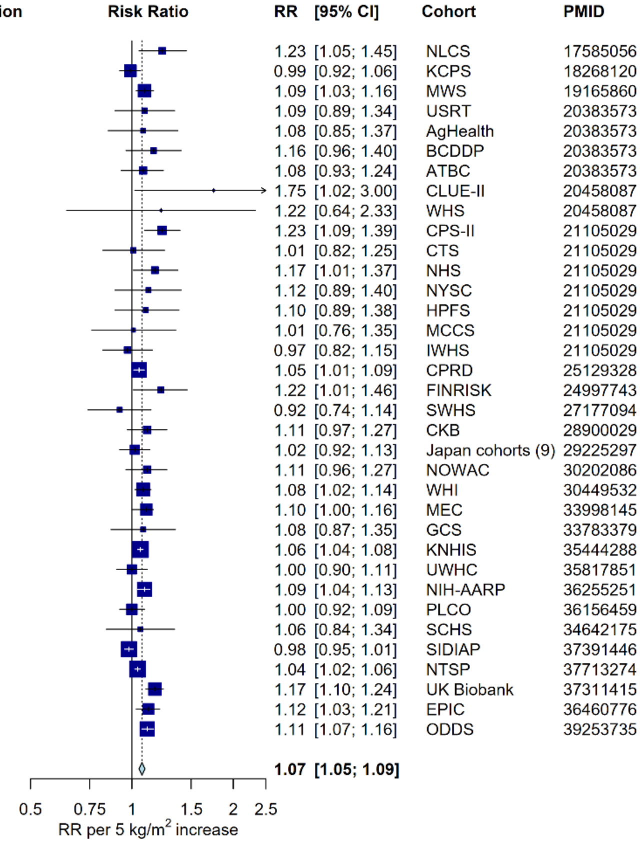

Heterogeneity: I^2 = 57.3%, τ^2 = 0.0013
Heterogeneity Q = 80, df = 34, p = 1.6 × 10^-5
Median RR (25th, 75th percentile) = 1.09 (1.03, 1.12)

Supplementary Figure 14: Meta-analysis of prospective studies on the risk of pancreatic cancer in relation to BMI

Study-specific RRs are represented by squares (with their 95% CIs as lines). RRs were combined using weighted averages of the log RRs in the separate studies, with weights assigned based on within and between study variance, yielding a pooled risk estimate and its 95% CI (diamond). Heterogeneity across studies was assessed using I^2, tau^2, and the 25th and 75th percentile of the RRs. Further details of model adjustments, follow-up time, analytic population for each study are available from **Supplementary Data**.

Abbreviations: BMI=body mass index, CI=confidence interval, PMID=PubMed ID, RR=risk ratio

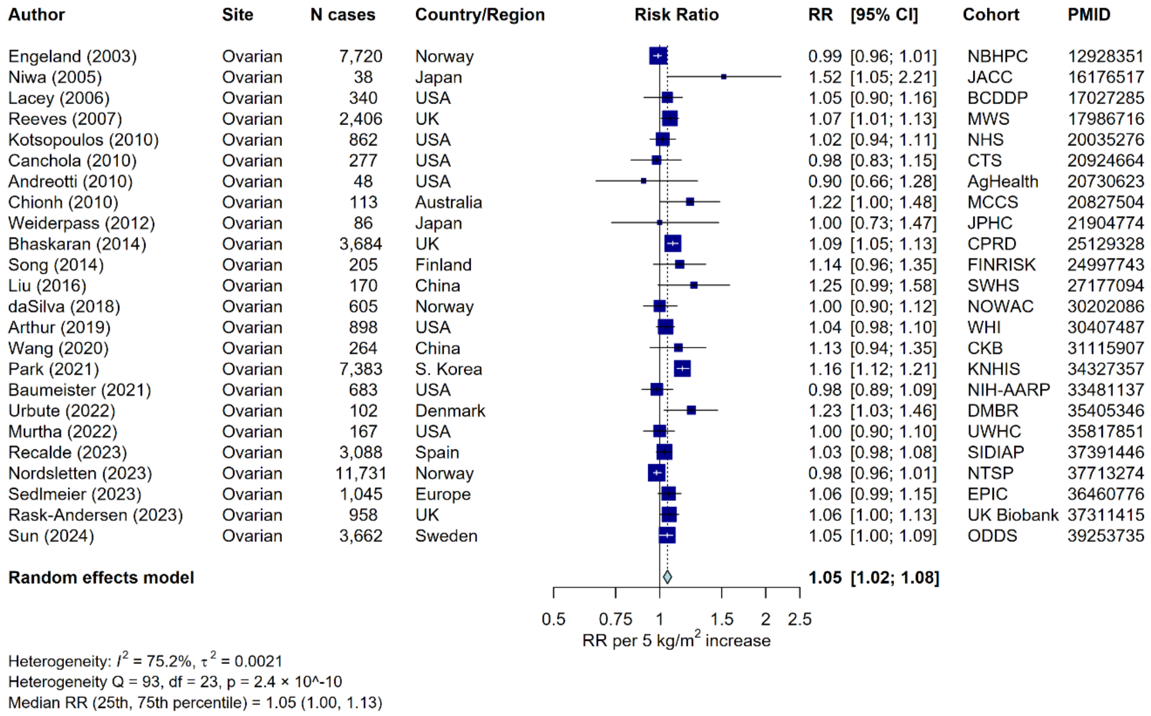

Supplementary Figure 15: Meta-analysis of prospective studies on the risk of ovarian cancer in relation to BMI

Study-specific RRs are represented by squares (with their 95% CIs as lines). RRs were combined using weighted averages of the log RRs in the separate studies, with weights assigned based on within and between study variance, yielding a pooled risk estimate and its 95% CI (diamond). Heterogeneity across studies was assessed using I^2, tau^2, and the 25th and 75th percentile of the RRs. Further details of model adjustments, follow-up time, analytic population for each study are available from **Supplementary Data**.

Abbreviations: BMI=body mass index, CI=confidence interval, PMID=PubMed ID, RR=risk ratio

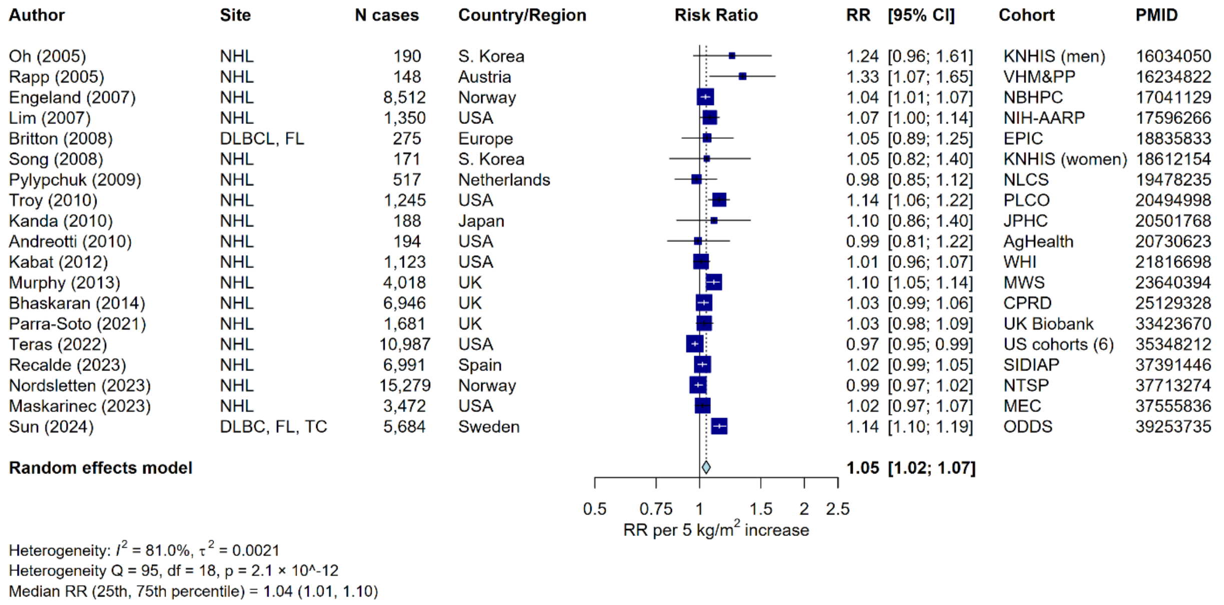

Supplementary Figure 16: Meta-analysis of prospective studies on the risk of NHL in relation to BMI

Study-specific RRs are represented by squares (with their 95% CIs as lines). RRs were combined using weighted averages of the log RRs in the separate studies, with weights assigned based on within and between study variance, yielding a pooled risk estimate and its 95% CI (diamond). Heterogeneity across studies was assessed using I^2, tau^2, and the 25th and 75th percentile of the RRs. Further details of model adjustments, follow-up time, analytic population for each study are available from **Supplementary Data**.

Abbreviations: BMI=body mass index, CI=confidence interval, DLBCL=diffuse large B-cell lymphoma, FL=follicular lymphoma, NHL=non-Hodgkin lymphoma, PMID=PubMed ID, RR=risk ratio, TC=T-cell lymphoma

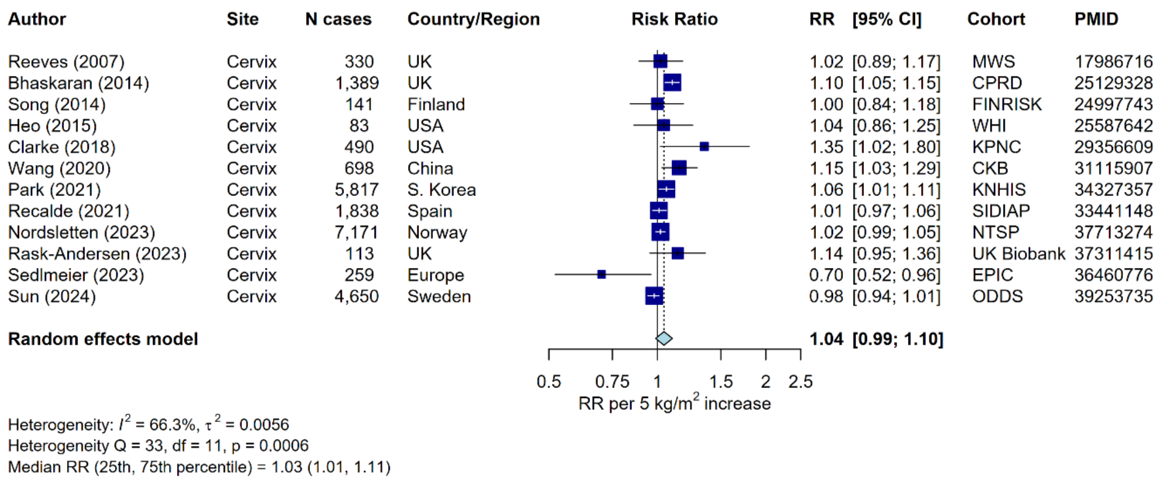

Supplementary Figure 17: Meta-analysis of prospective studies on the risk of cervical cancer in relation to BMI

Study-specific RRs are represented by squares (with their 95% CIs as lines). RRs were combined using weighted averages of the log RRs in the separate studies, with weights assigned based on within and between study variance, yielding a pooled risk estimate and its 95% CI (diamond). Heterogeneity across studies was assessed using I^2, tau^2, and the 25th and 75th percentile of the RRs. Further details of model adjustments, follow-up time, analytic population for each study are available from **Supplementary Data**.

Abbreviations: BMI=body mass index, CI=confidence interval, PMID=PubMed ID, RR=risk ratio

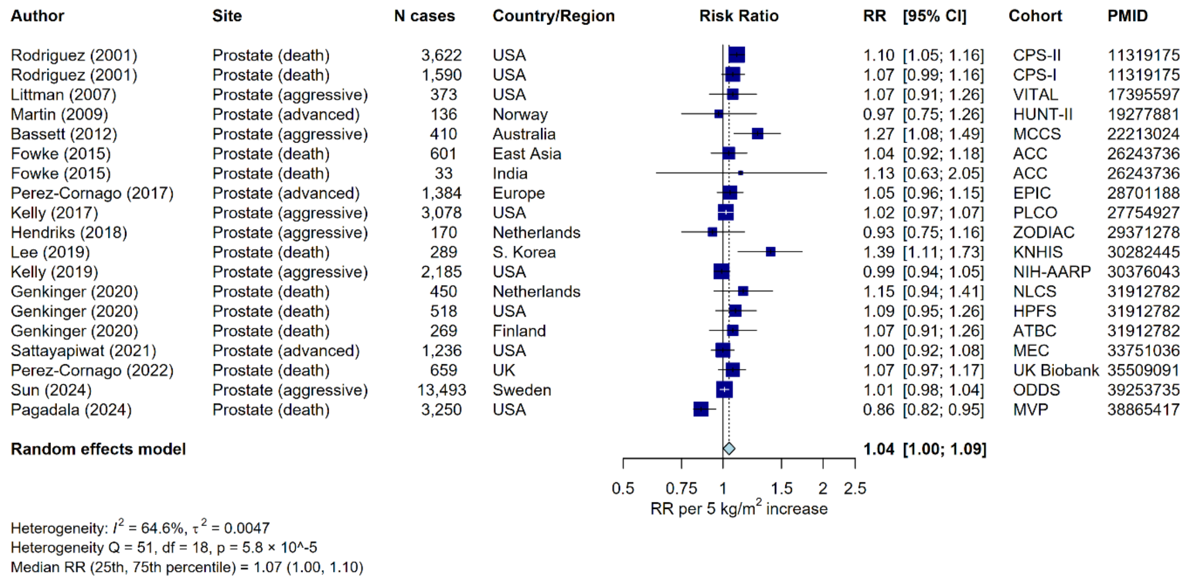

Supplementary Figure 18: Meta-analysis of prospective studies on the risk of prostate cancer (aggressive) in relation to BMI

Aggressive prostate cancer was defined as any of stage 3-4 on the American Joint Committee on Cancer 1992 classification, advanced cancer, advanced or metastatic cancer; metastatic cancer; stage C or D on the Whitmore/Jewett scale; fatal cancer (prostate cancer-specific mortality); high stage or grade; Gleason grade ≥ 7). Study-specific RRs are represented by squares (with their 95% CIs as lines). RRs were combined using weighted averages of the log RRs in the separate studies, with weights assigned based on within and between study variance, yielding a pooled risk estimate and its 95% CI (diamond). Heterogeneity across studies was assessed using I^2, tau^2, and the 25th and 75th percentile of the RRs. Further details of model adjustments, follow-up time, analytic population for each study are available from **Supplementary Data**.

Abbreviations: BMI=body mass index, CI=confidence interval, PMID=PubMed ID, RR=risk ratio

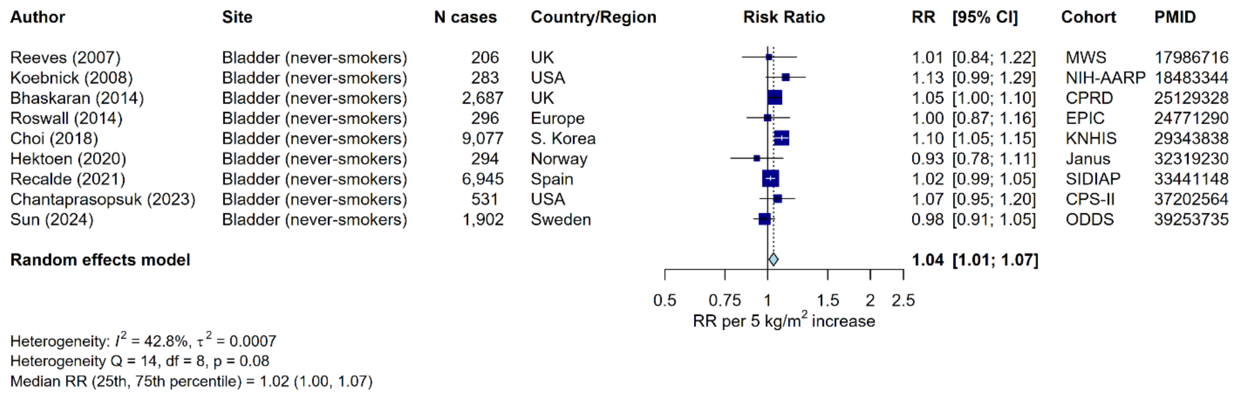

Supplementary Figure 19: Meta-analysis of prospective studies on the risk of bladder cancer (restricted to never-smokers) in relation to BMI

Study-specific RRs are represented by squares (with their 95% CIs as lines). RRs were combined using weighted averages of the log RRs in the separate studies, with weights assigned based on within and between study variance, yielding a pooled risk estimate and its 95% CI (diamond). Heterogeneity across studies was assessed using I^2, tau^2, and the 25th and 75th percentile of the RRs. Further details of model adjustments, follow-up time, analytic population for each study are available from **Supplementary Data**.

Abbreviations: BMI=body mass index, CI=confidence interval, PMID=PubMed ID, RR=risk ratio

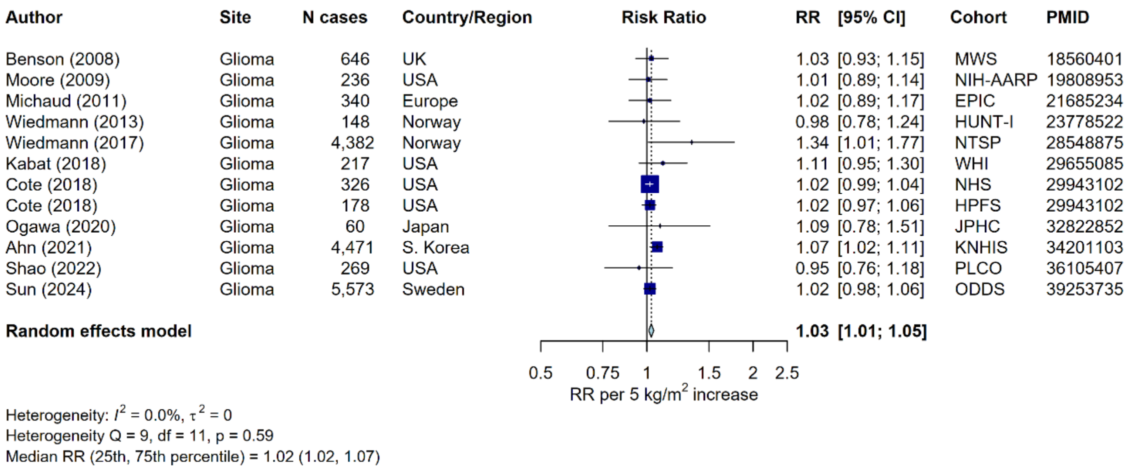

Supplementary Figure 20: Meta-analysis of prospective studies on the risk of glioma in relation to BMI

Study-specific RRs are represented by squares (with their 95% CIs as lines). RRs were combined using weighted averages of the log RRs in the separate studies, with weights assigned based on within and between study variance, yielding a pooled risk estimate and its 95% CI (diamond). Heterogeneity across studies was assessed using I^2, tau^2, and the 25th and 75th percentile of the RRs. Further details of model adjustments, follow-up time, analytic population for each study are available from **Supplementary Data**.

Abbreviations: BMI=body mass index, CI=confidence interval, PMID=PubMed ID, RR=risk ratio

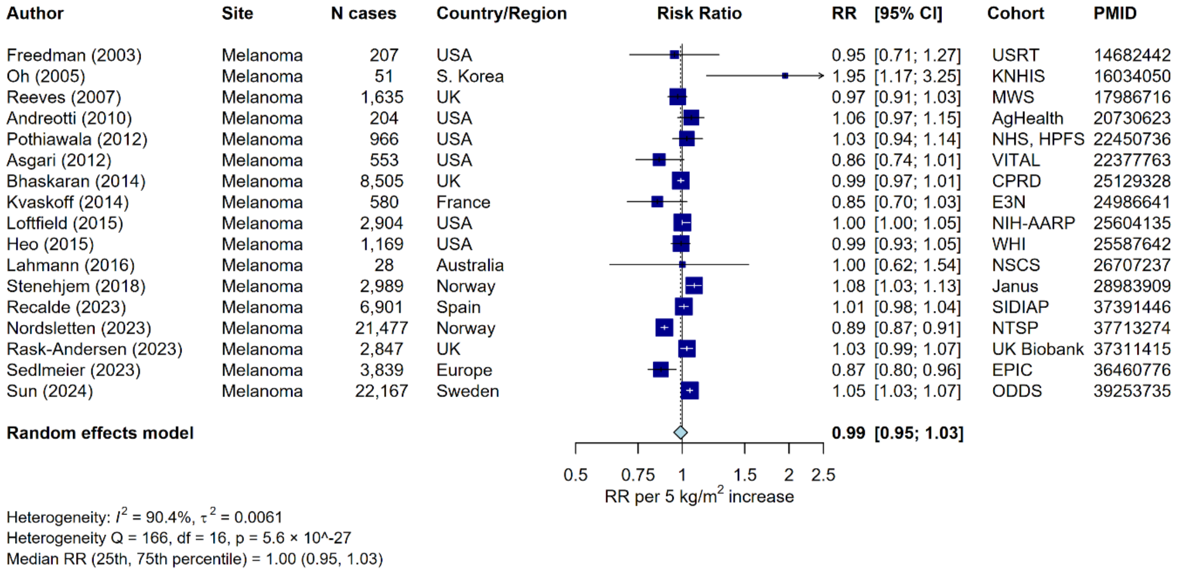

Supplementary Figure 21: Meta-analysis of prospective studies on the risk of melanoma in relation to BMI

Study-specific RRs are represented by squares (with their 95% CIs as lines). RRs were combined using weighted averages of the log RRs in the separate studies, with weights assigned based on within and between study variance, yielding a pooled risk estimate and its 95% CI (diamond). Heterogeneity across studies was assessed using I^2, tau^2, and the 25th and 75th percentile of the RRs. Further details of model adjustments, follow-up time, analytic population for each study are available from **Supplementary Data**.

Abbreviations: BMI=body mass index, CI=confidence interval, PMID=PubMed ID, RR=risk ratio

Author	Site	N cases	Country/Region	RR	[95% CI]	Cohort	PMID
Lindblad (2005)	Gastric (non-cardia)	192	UK	0.90	[0.74; 1.11]	CPRD	15947880
MacInnis (2006)	Gastric (non-cardia)	68	Australia	0.95	[0.70; 1.30]	MCCS	16353151
Merry (2007)	Gastric (non-cardia)	235	Netherlands	0.95	[0.73; 1.22]	NLCS	17337464
Abnet (2008)	Gastric (non-cardia)	315	USA	0.99	[0.87; 1.12]	NIH-AARP	18221867
Sjodahl (2008)	Gastric (non-cardia)	207	Norway	1.09	[0.86; 1.37]	HUNT-I	18187390
Wei (2019)	Gastric (non-cardia)	231	China	0.87	[0.71; 1.07]	Kailun Cohort	32062909
Si (2020)	Gastric (non-cardia)	231	China	1.16	[0.78; 1.70]	SMCHBDP	32342643
Choi (2021)	Gastric (non-cardia)	42,441	S. Korea	1.03	[1.01; 1.04]	KNHIS	34033134
Liu (2021)	Gastric (non-cardia)	191	UK	1.07	[0.92; 1.26]	UK Biobank	33624430
HashemiMadani (2021)	Gastric (non-cardia)	282	Iran	1.07	[0.88; 1.29]	GCS	33783379
Jang (2022)	Gastric (non-cardia)	6,314	East Asia	0.92	[0.87; 0.97]	ACC	35793701
Lee (2022)	Gastric (non-cardia)	1,041	S. Korea	1.07	[0.95; 1.19]	HEXA	35120118
Sedlmeier (2023)	Gastric (non-cardia)	331	Europe	1.01	[0.88; 1.17]	EPIC	36460776
Sun (2024)	Gastric (non-cardia)	3,892	Sweden	0.92	[0.88; 0.97]	ODDS	39253735
Choi (2024)	Gastric (non-cardia)	4,277	S. Korea	1.08	[1.03; 1.13]	NHIS-HEALS	40703687
Saeed (2025)	Gastric (non-cardia)	3,661	Norway	1.03	[0.98; 1.08]	NTSP	40091197
Chan (2025)	Gastric (non-cardia)	2,957	China	0.81	[0.76; 0.87]	CKB	39737804
**Random effects model**				**0.98**	**[0.94; 1.03]**		

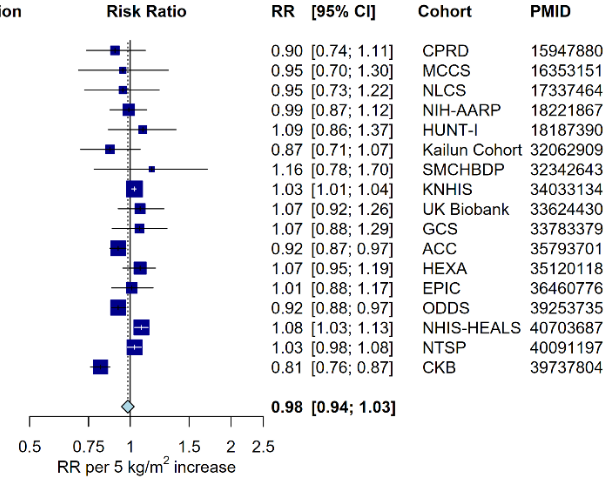

Heterogeneity: I^2 = 81.4%, τ^2 = 0.0043
Heterogeneity Q = 86, df = 16, p = 1.3 × 10^-11
Median RR (25th, 75th percentile) = 1.01 (0.92, 1.07)

Supplementary Figure 22: Meta-analysis of prospective studies on the risk of gastric cancer (non-cardia) in relation to BMI

Study-specific RRs are represented by squares (with their 95% CIs as lines). RRs were combined using weighted averages of the log RRs in the separate studies, with weights assigned based on within and between study variance, yielding a pooled risk estimate and its 95% CI (diamond). Heterogeneity across studies was assessed using I^2, tau^2, and the 25th and 75th percentile of the RRs. Further details of model adjustments, follow-up time, analytic population for each study are available from **Supplementary Data**.

Abbreviations: BMI=body mass index, CI=confidence interval, PMID=PubMed ID, RR=risk ratio

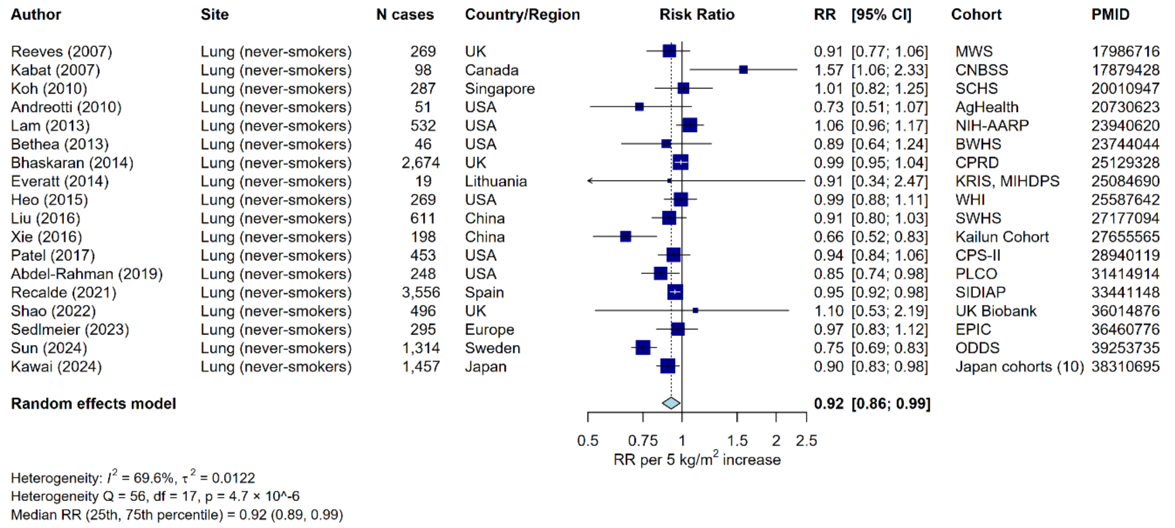

Supplementary Figure 23: Meta-analysis of prospective studies on the risk of lung cancer (restricted to never-smokers) in relation to BMI

Study-specific RRs are represented by squares (with their 95% CIs as lines). RRs were combined using weighted averages of the log RRs in the separate studies, with weights assigned based on within and between study variance, yielding a pooled risk estimate and its 95% CI (diamond). Heterogeneity across studies was assessed using I^2, tau^2, and the 25th and 75th percentile of the RRs. Further details of model adjustments, follow-up time, analytic population for each study are available from **Supplementary Data**.

Abbreviations: BMI=body mass index, CI=confidence interval, PMID=PubMed ID, RR=risk ratio

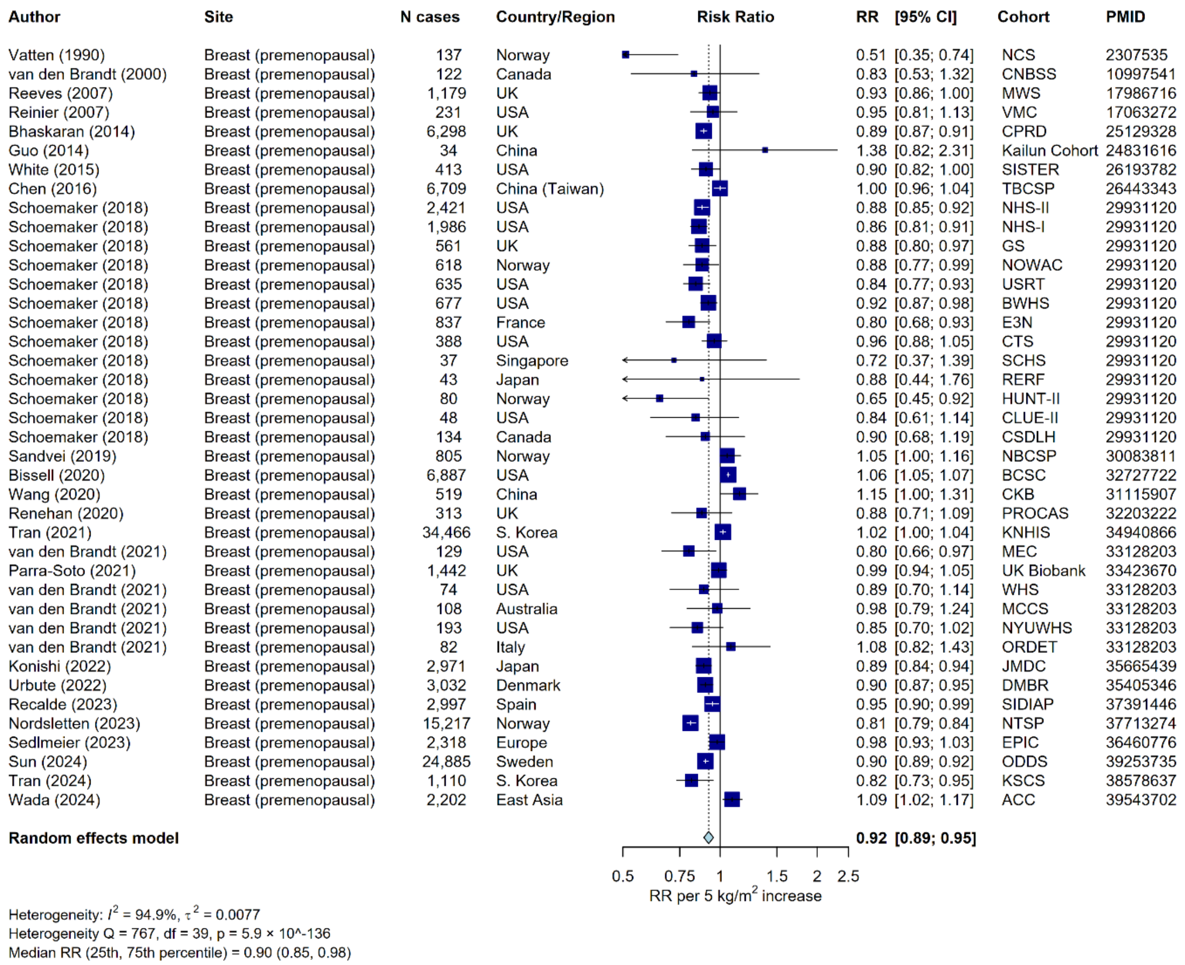

Supplementary Figure 24: Meta-analysis of prospective studies on the risk of breast cancer (premenopausal) in relation to BMI

Study-specific RRs are represented by squares (with their 95% CIs as lines). RRs were combined using weighted averages of the log RRs in the separate studies, with weights assigned based on within and between study variance, yielding a pooled risk estimate and its 95% CI (diamond). Heterogeneity across studies was assessed using I^2, tau^2, and the 25th and 75th percentile of the RRs. Further details of model adjustments, follow-up time, analytic population for each study are available from **Supplementary Data**.

Abbreviations: BMI=body mass index, CI=confidence interval, PMID=PubMed ID, RR=risk ratio

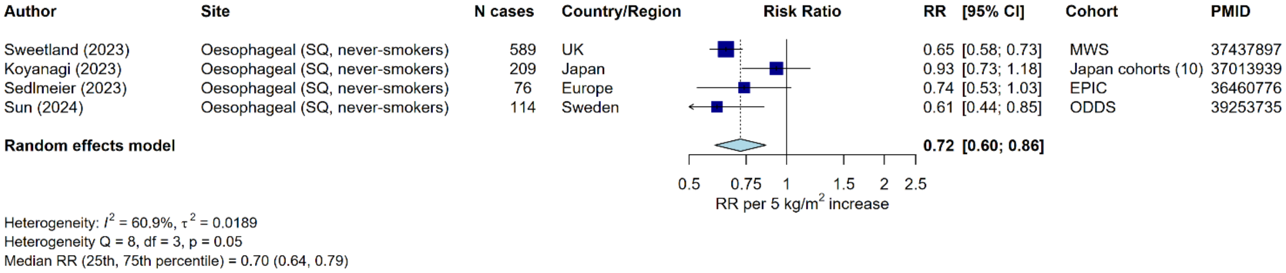

Supplementary Figure 25: Meta-analysis of prospective studies on the risk of oesophageal (SQ, restricted to never-smokers) in relation to BMI

Study-specific RRs are represented by squares (with their 95% CIs as lines). RRs were combined using weighted averages of the log RRs in the separate studies, with weights assigned based on within and between study variance, yielding a pooled risk estimate and its 95% CI (diamond). Heterogeneity across studies was assessed using I^2, tau^2, and the 25th and 75th percentile of the RRs. Further details of model adjustments, follow-up time, analytic population for each study are available from **Supplementary Data**.

Abbreviations: BMI=body mass index, CI=confidence interval, PMID=PubMed ID, RR=risk ratio, SQ=squamous cell carcinoma.

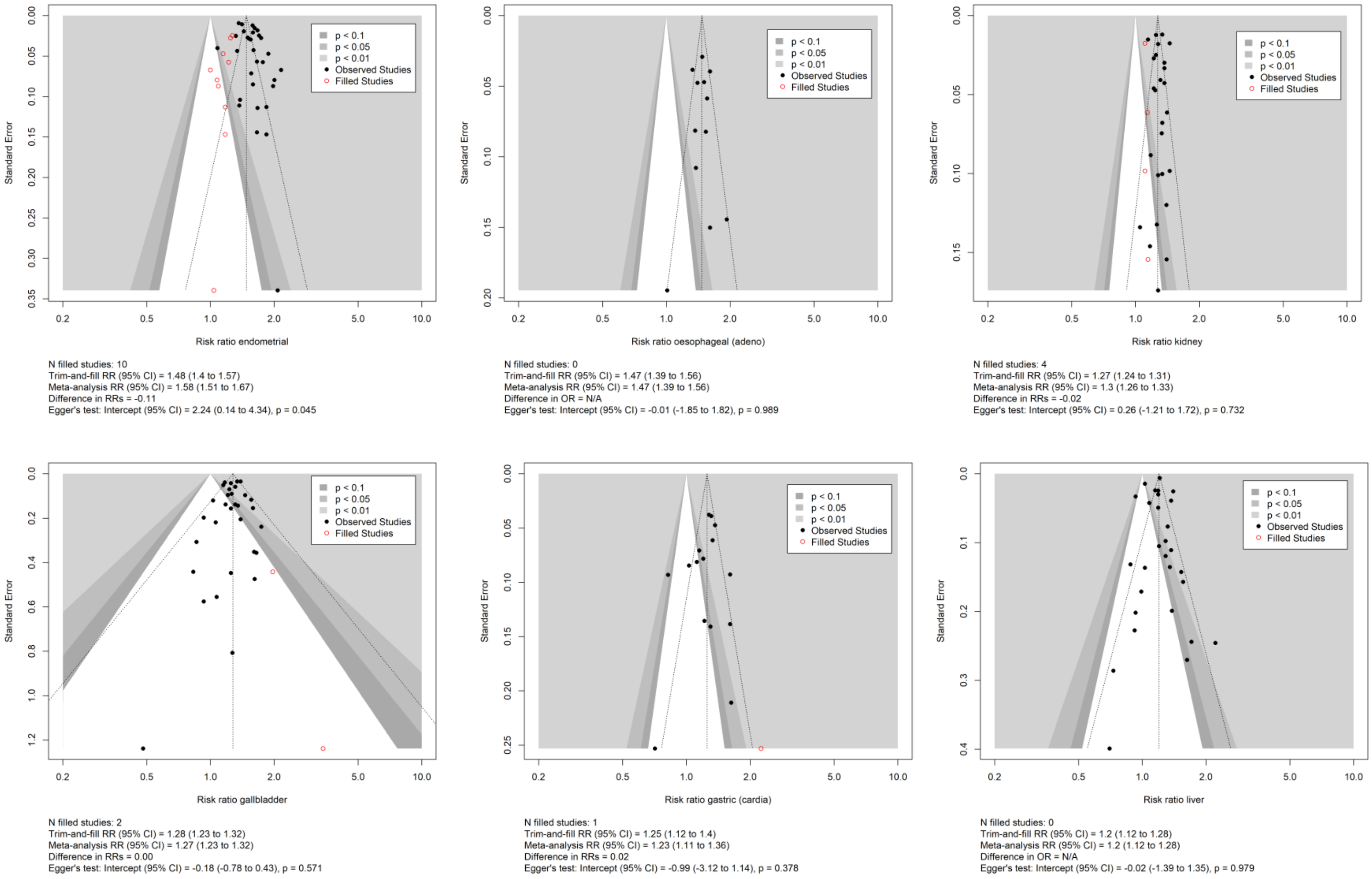

Supplementary Figure 26: Trim-and-fill for publication bias and Egger's intercept test for the 25 common cancers

RRs were combined using random effects meta-analysis. Small-study effects were assessed using Egger's regression test. The intercept (β_0) and corresponding t-statistic (df = k − 2) were estimated, with exact two-sided P values reported. No adjustment for multiple comparisons was applied.

Abbreviations: CI=confidence interval, RR= risk ratio

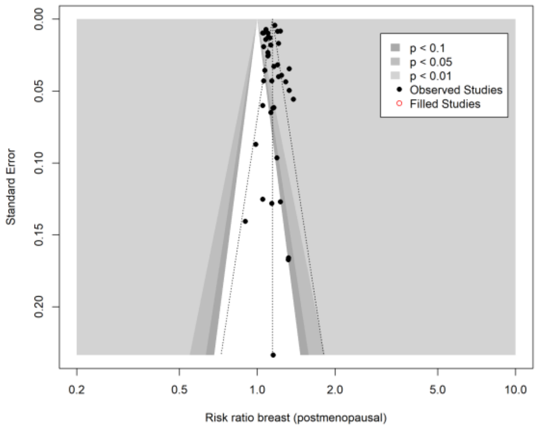

N filled studies: 0
Trim-and-fill RR (95% CI) = 1.14 (1.12 to 1.17)
Meta-analysis RR (95% CI) = 1.14 (1.12 to 1.17)
Difference in OR = N/A
Egger's test: Intercept (95% CI) = -0.09 (-1.63 to 1.46), p = 0.913

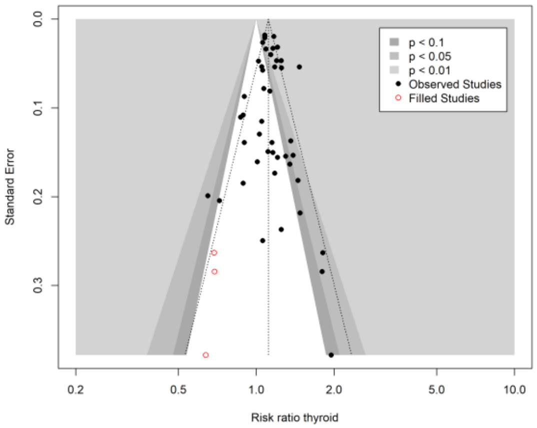

N filled studies: 3
Trim-and-fill RR (95% CI) = 1.11 (1.06 to 1.17)
Meta-analysis RR (95% CI) = 1.12 (1.07 to 1.17)
Difference in RRs = -0.01
Egger's test: Intercept (95% CI) = 0.25 (-0.49 to 0.98), p = 0.518

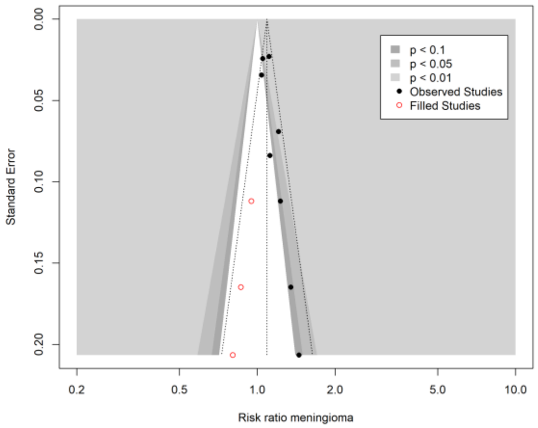

N filled studies: 3
Trim-and-fill RR (95% CI) = 1.09 (1.02 to 1.17)
Meta-analysis RR (95% CI) = 1.11 (1.05 to 1.16)
Difference in RRs = -0.01
Egger's test: Intercept (95% CI) = 1.46 (0.29 to 2.63), p = 0.05

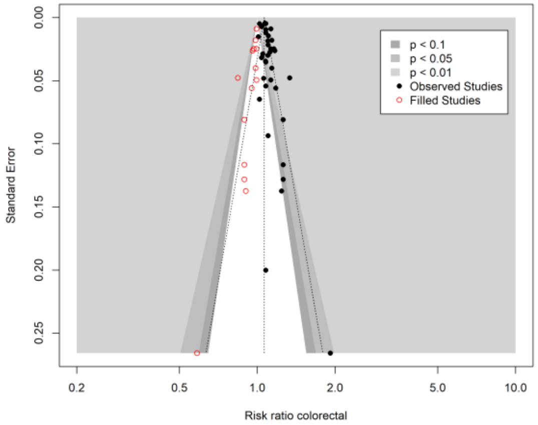

N filled studies: 14
Trim-and-fill RR (95% CI) = 1.06 (1.04 to 1.09)
Meta-analysis RR (95% CI) = 1.1 (1.08 to 1.12)
Difference in RRs = -0.04
Egger's test: Intercept (95% CI) = 1.66 (0.52 to 2.81), p = 0.007

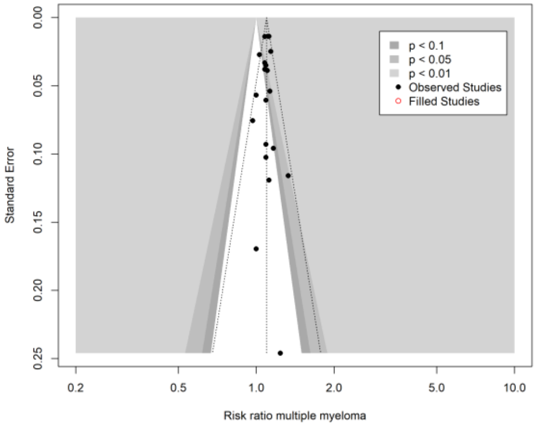

N filled studies: 0
Trim-and-fill RR (95% CI) = 1.1 (1.08 to 1.11)
Meta-analysis RR (95% CI) = 1.1 (1.08 to 1.11)
Difference in OR = N/A
Egger's test: Intercept (95% CI) = -0.17 (-0.93 to 0.6), p = 0.673

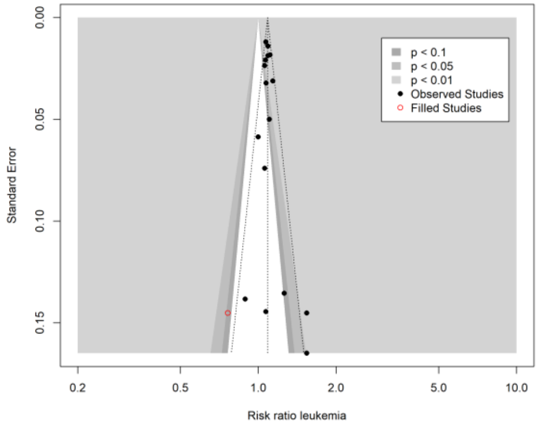

N filled studies: 1
Trim-and-fill RR (95% CI) = 1.09 (1.03 to 1.14)
Meta-analysis RR (95% CI) = 1.09 (1.05 to 1.13)
Difference in RRs = -0.00
Egger's test: Intercept (95% CI) = 0.57 (-0.41 to 1.55), p = 0.274

Supplementary Figure 26: Trim-and-fill for publication bias and Egger's intercept test for the 25 common cancers (continued)

RRs were combined using random effects meta-analysis. Small-study effects were assessed using Egger's regression test. The intercept (β_0) and corresponding t-statistic ($df = k - 2$) were estimated, with exact two-sided P values reported. No adjustment for multiple comparisons was applied.

Abbreviations: CI=confidence interval, RR= risk ratio

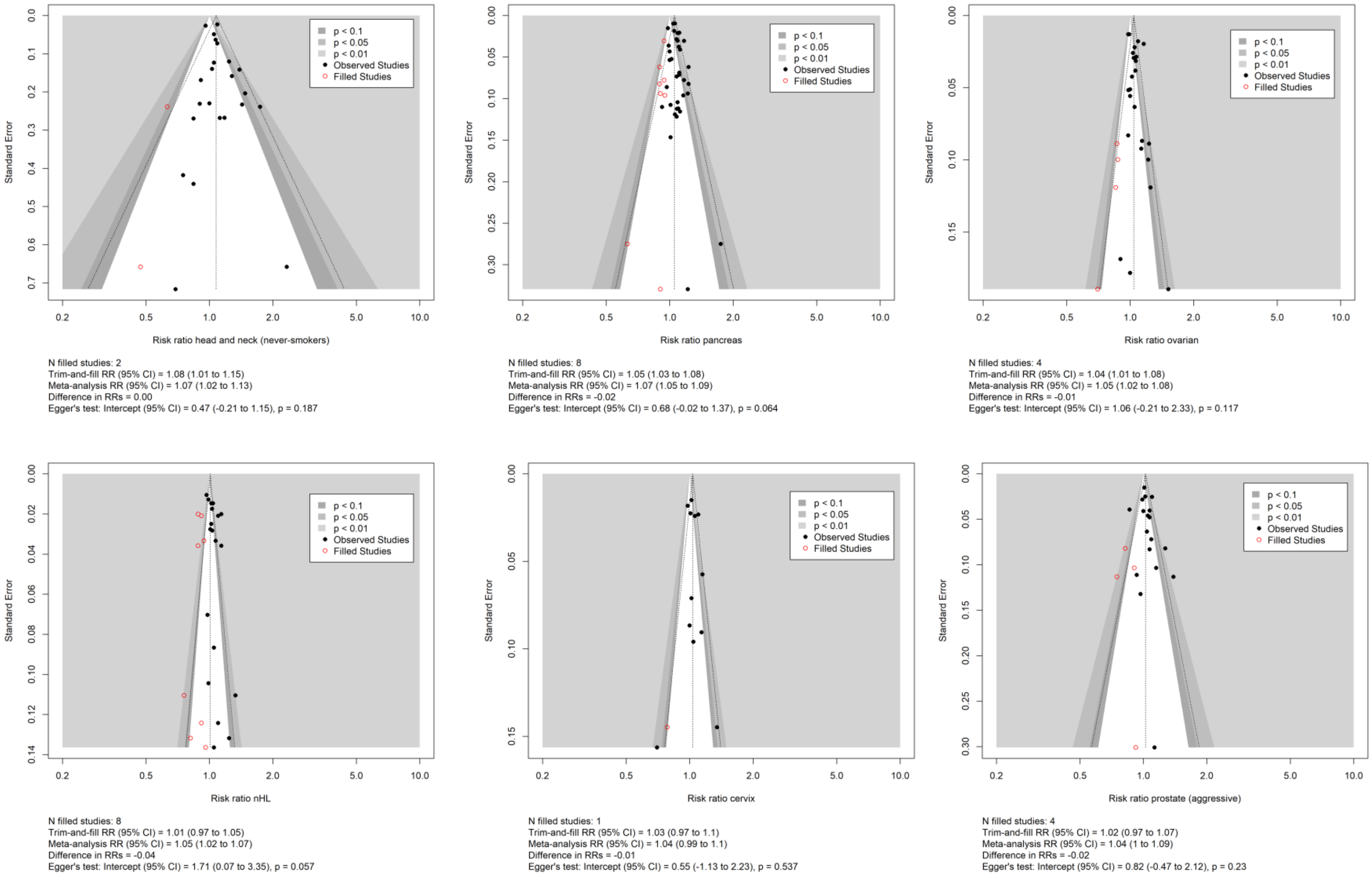

Supplementary Figure 26: Trim-and-fill for publication bias and Egger's intercept test for the 25 common cancers (continued)

RRs were combined using random effects meta-analysis. Small-study effects were assessed using Egger's regression test. The intercept (β_0) and corresponding t-statistic (df = k − 2) were estimated, with exact two-sided P values reported. No adjustment for multiple comparisons was applied.

Abbreviations: CI=confidence interval, RR= risk ratio

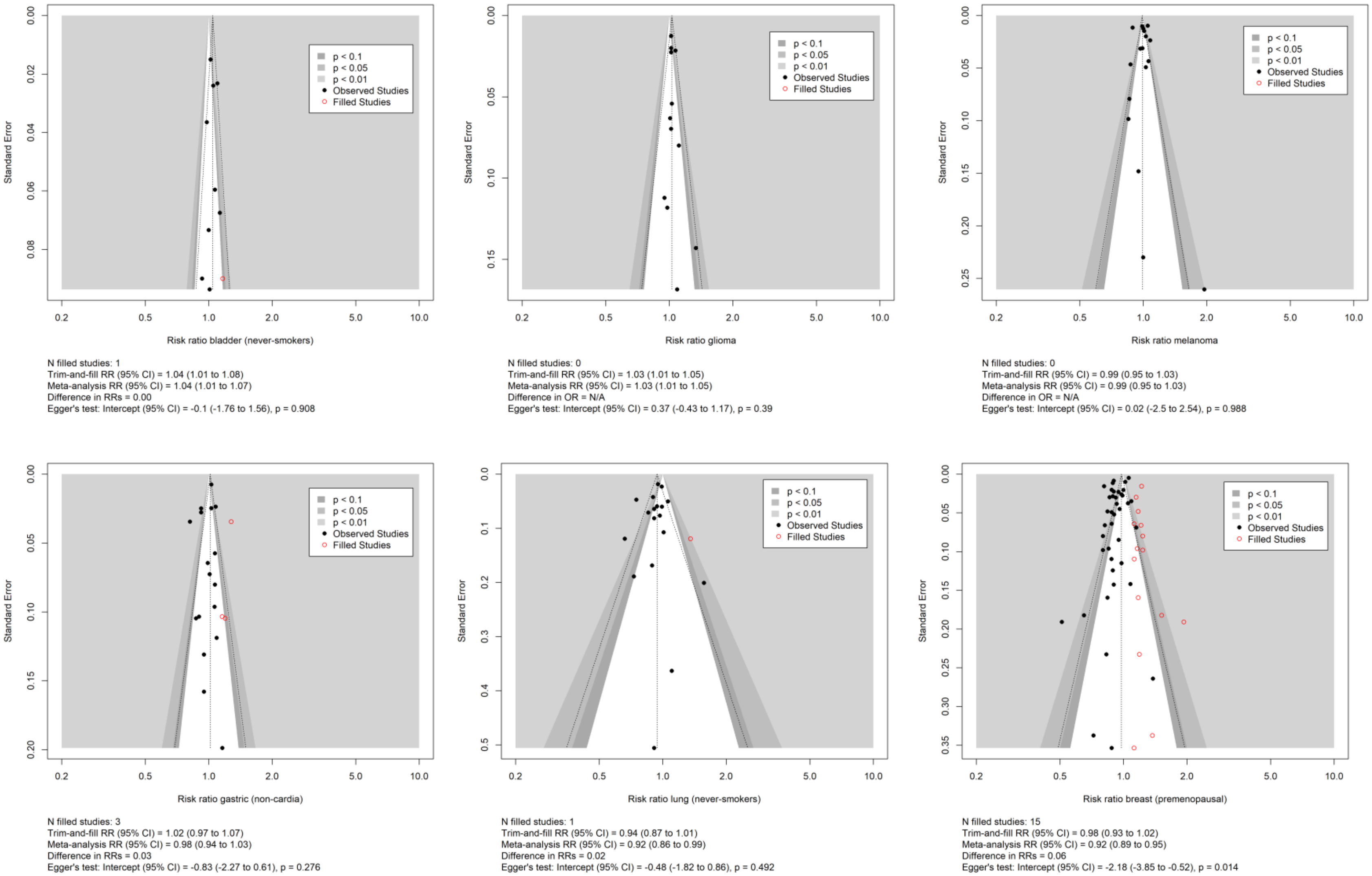

Supplementary Figure 26: Trim-and-fill for publication bias and Egger's intercept test for the 25 common cancers (continued)

RRs were combined using random effects meta-analysis. Small-study effects were assessed using Egger's regression test. The intercept (β_0) and corresponding t-statistic (df = k − 2) were estimated, with exact two-sided P values reported. No adjustment for multiple comparisons was applied.

Abbreviations: CI=confidence interval, RR= risk ratio

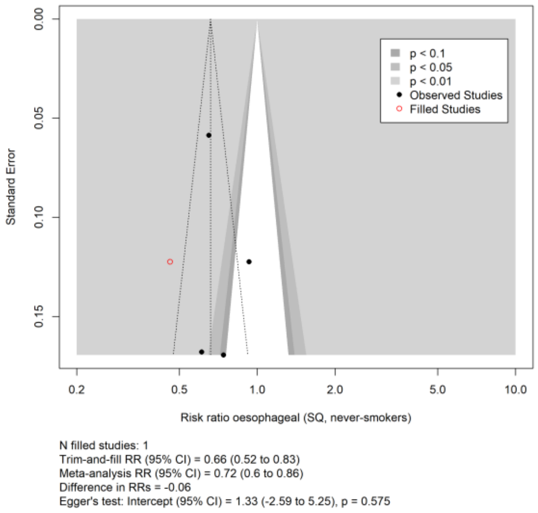

Supplementary Figure 26: Trim-and-fill for publication bias and Egger's intercept test for the 25 common cancers (continued)

RRs were combined using random effects meta-analysis. Small-study effects were assessed using Egger's regression test. The intercept (β_0) and corresponding t-statistic ($df = k - 2$) were estimated, with exact two-sided P values reported. No adjustment for multiple comparisons was applied.

Abbreviations: CI=confidence interval, RR= risk ratio

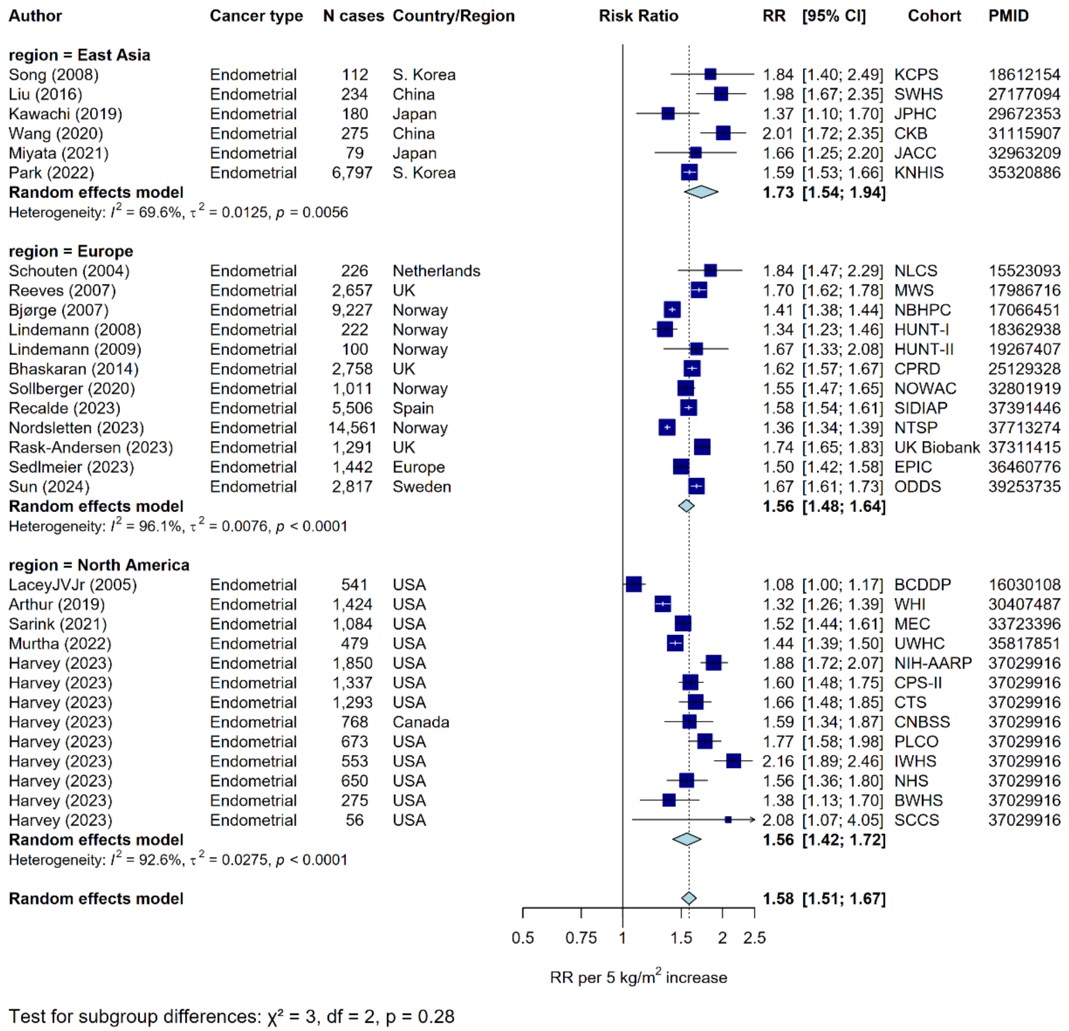

Supplementary Figure 27: Meta-analysis of prospective studies on the risk of endometrial cancer in relation to BMI, by region

Study-specific RRs are represented by squares (with their 95% CIs as lines). RRs were combined using random effects meta-analysis (represented by a diamond). Heterogeneity across studies was assessed using I^2 and tau^2 within region. Heterogeneity in the pooled estimates by region was assessed using the Q-statistic. Further details of model adjustments, follow-up time, analytic population for each study are available from **Supplementary Data**.

Abbreviations: BMI=body mass index, CI=confidence interval, PMID=PubMed ID, RR=risk ratio

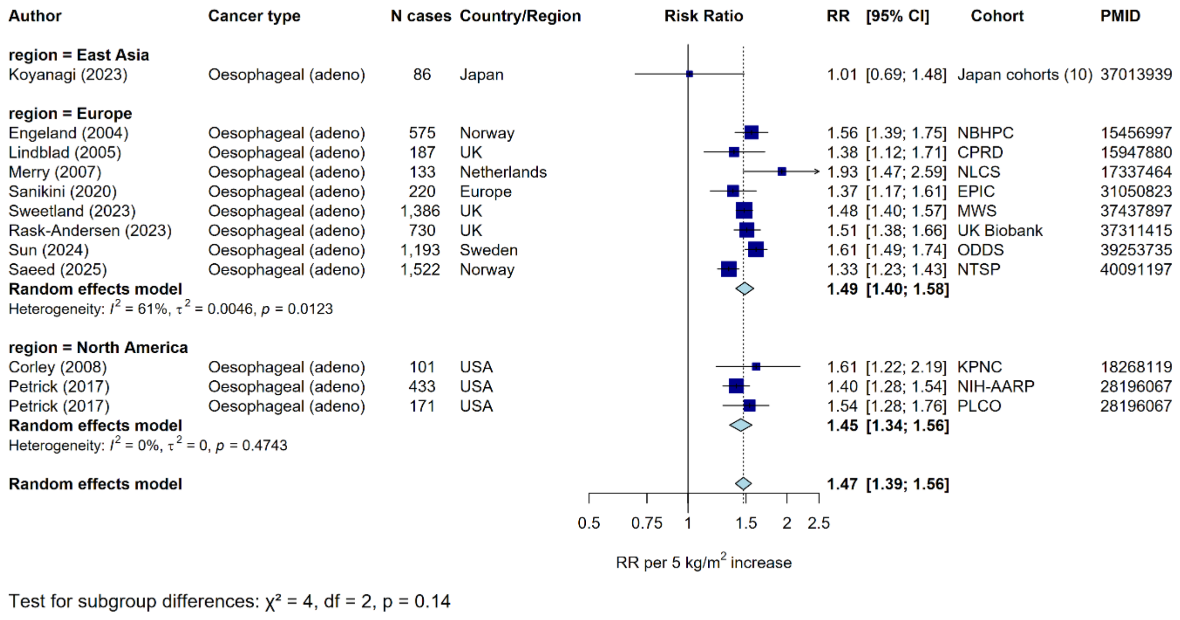

Supplementary Figure 28: Meta-analysis of prospective studies on the risk of oesophageal adenocarcinoma cancer in relation to BMI, by region

Study-specific RRs are represented by squares (with their 95% CIs as lines). RRs were combined using random effects meta-analysis (represented by a diamond). Heterogeneity across studies was assessed using I^2 and tau^2 within region. Heterogeneity in the pooled estimates by region was assessed using the Q-statistic. Further details of model adjustments, follow-up time, analytic population for each study are available from **Supplementary Data**.

Abbreviations: BMI=body mass index, CI=confidence interval, PMID=PubMed ID, RR=risk ratio

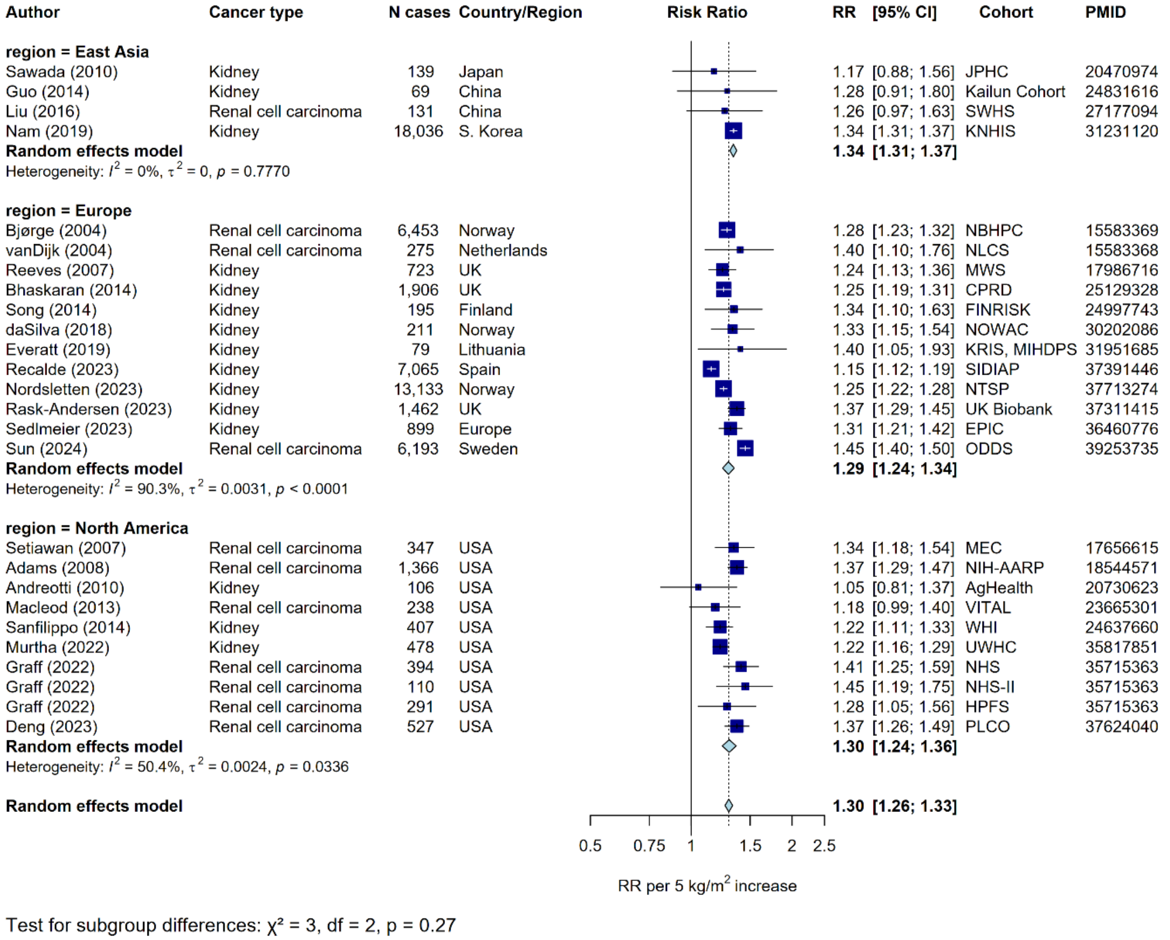

Supplementary Figure 29: Meta-analysis of prospective studies on the risk of kidney cancer in relation to BMI, by region

Study-specific RRs are represented by squares (with their 95% CIs as lines). RRs were combined using random effects meta-analysis (represented by a diamond). Heterogeneity across studies was assessed using I^2 and tau^2 within region. Heterogeneity in the pooled estimates by region was assessed using the Q-statistic. Further details of model adjustments, follow-up time, analytic population for each study are available from **Supplementary Data**.

Abbreviations: BMI=body mass index, CI=confidence interval, PMID=PubMed ID, RR=risk ratio

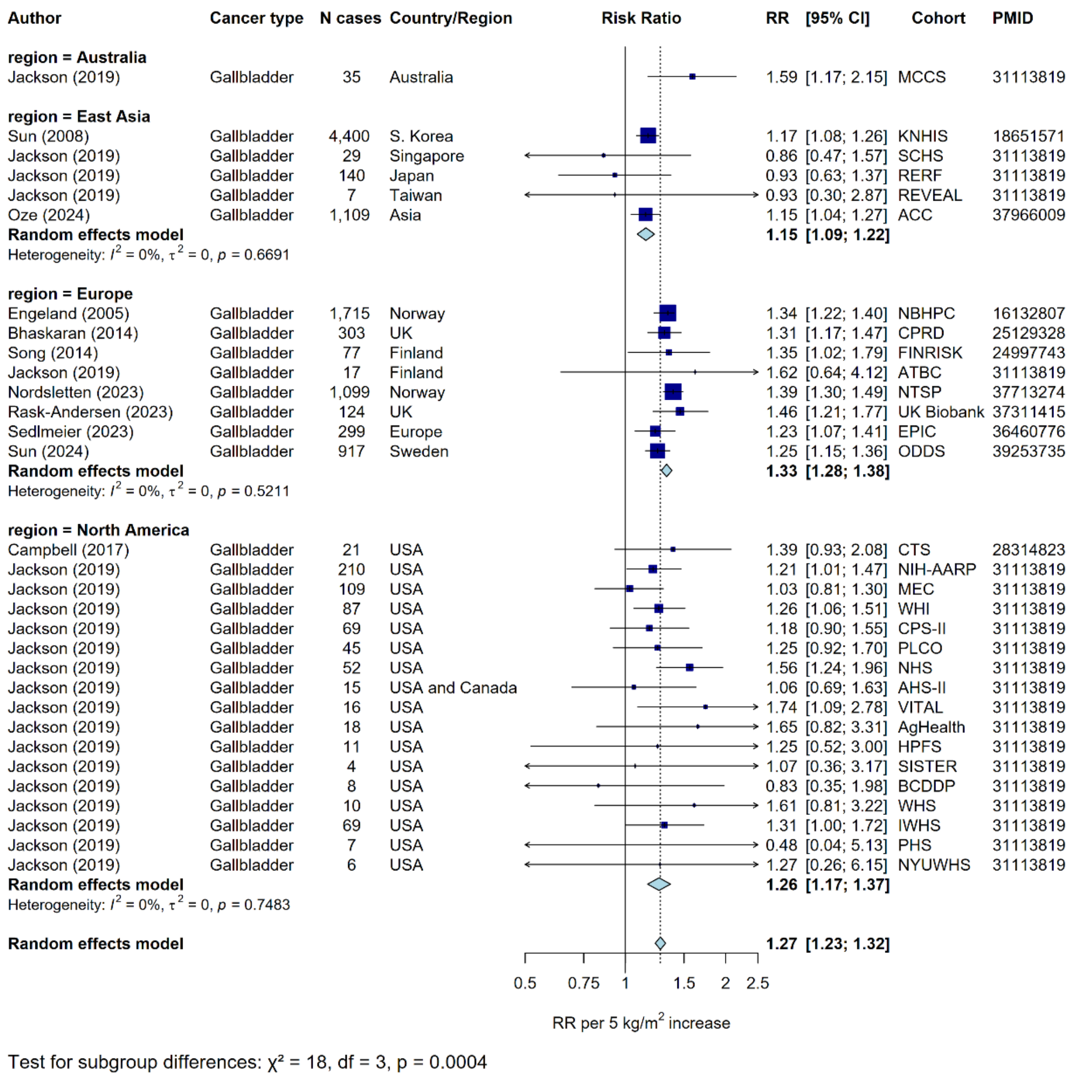

Supplementary Figure 30: Meta-analysis of prospective studies on the risk of gallbladder cancer in relation to BMI, by region

Study-specific RRs are represented by squares (with their 95% CIs as lines). RRs were combined using random effects meta-analysis (represented by a diamond). Heterogeneity across studies was assessed using I^2 and tau^2 within region. Heterogeneity in the pooled estimates by region was assessed using the Q-statistic. Further details of model adjustments, follow-up time, analytic population for each study are available from **Supplementary Data**.

Abbreviations: BMI=body mass index, CI=confidence interval, PMID=PubMed ID, RR=risk ratio

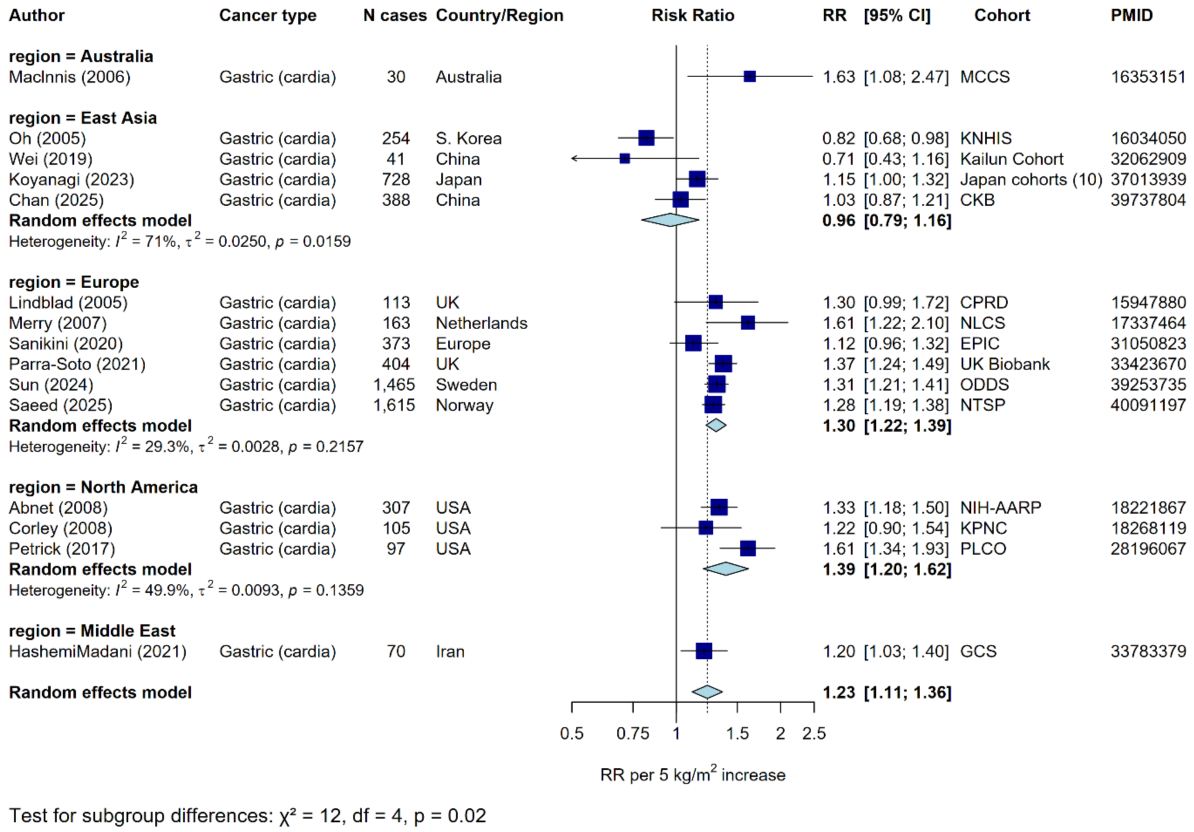

Supplementary Figure 31: Meta-analysis of prospective studies on the risk of gastric cancer (cardia) in relation to BMI, by region

Study-specific RRs are represented by squares (with their 95% CIs as lines). RRs were combined using random effects meta-analysis (represented by a diamond). Heterogeneity across studies was assessed using I^2 and tau^2 within region. Heterogeneity in the pooled estimates by region was assessed using the Q-statistic. Further details of model adjustments, follow-up time, analytic population for each study are available from **Supplementary Data**.

Abbreviations: BMI=body mass index, CI=confidence interval, PMID=PubMed ID, RR=risk ratio

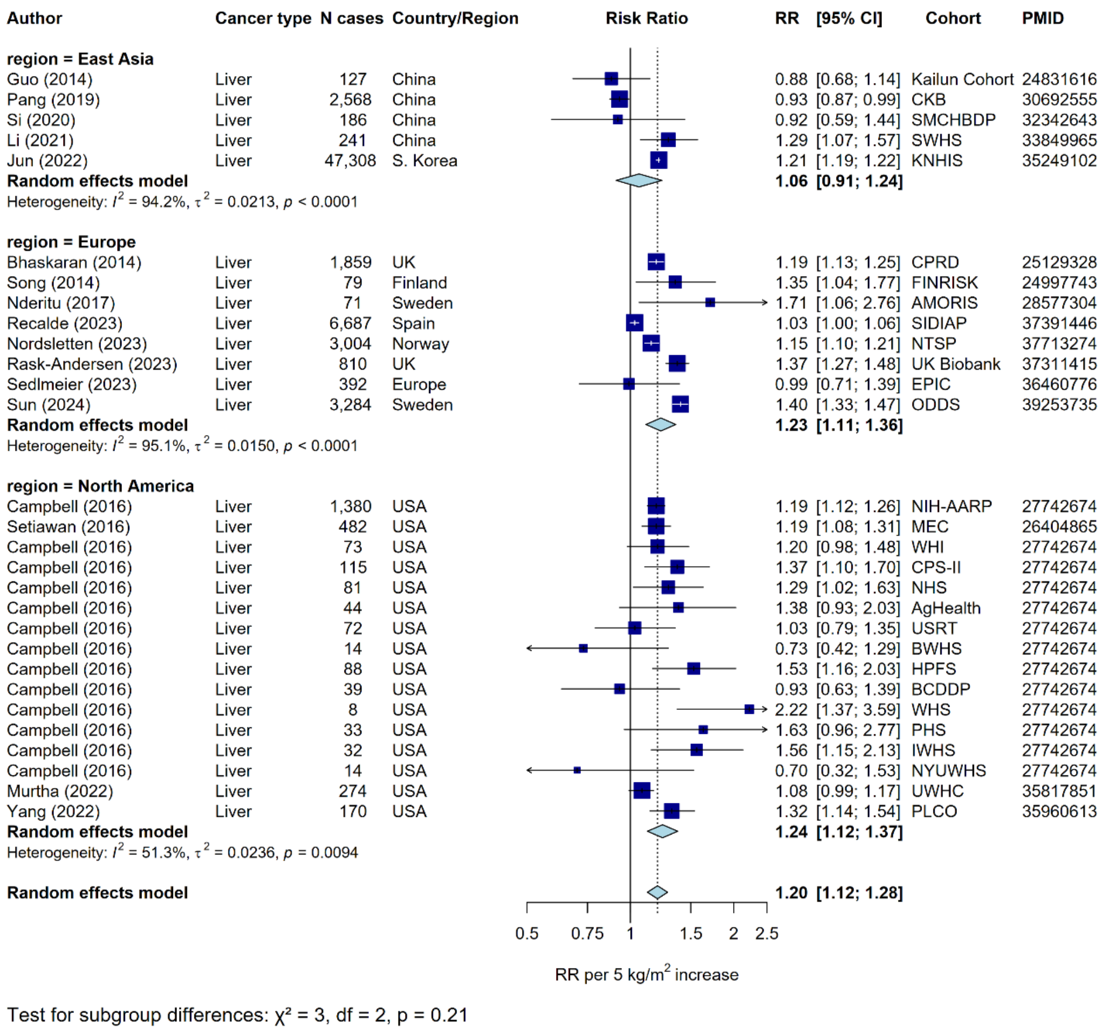

Supplementary Figure 32: Meta-analysis of prospective studies on the risk of liver cancer in relation to BMI, by region

Study-specific RRs are represented by squares (with their 95% CIs as lines). RRs were combined using random effects meta-analysis (represented by a diamond). Heterogeneity across studies was assessed using I^2 and tau^2 within region. Heterogeneity in the pooled estimates by region was assessed using the Q-statistic. Further details of model adjustments, follow-up time, analytic population for each study are available from **Supplementary Data**.

Abbreviations: BMI=body mass index, CI=confidence interval, PMID=PubMed ID, RR=risk ratio

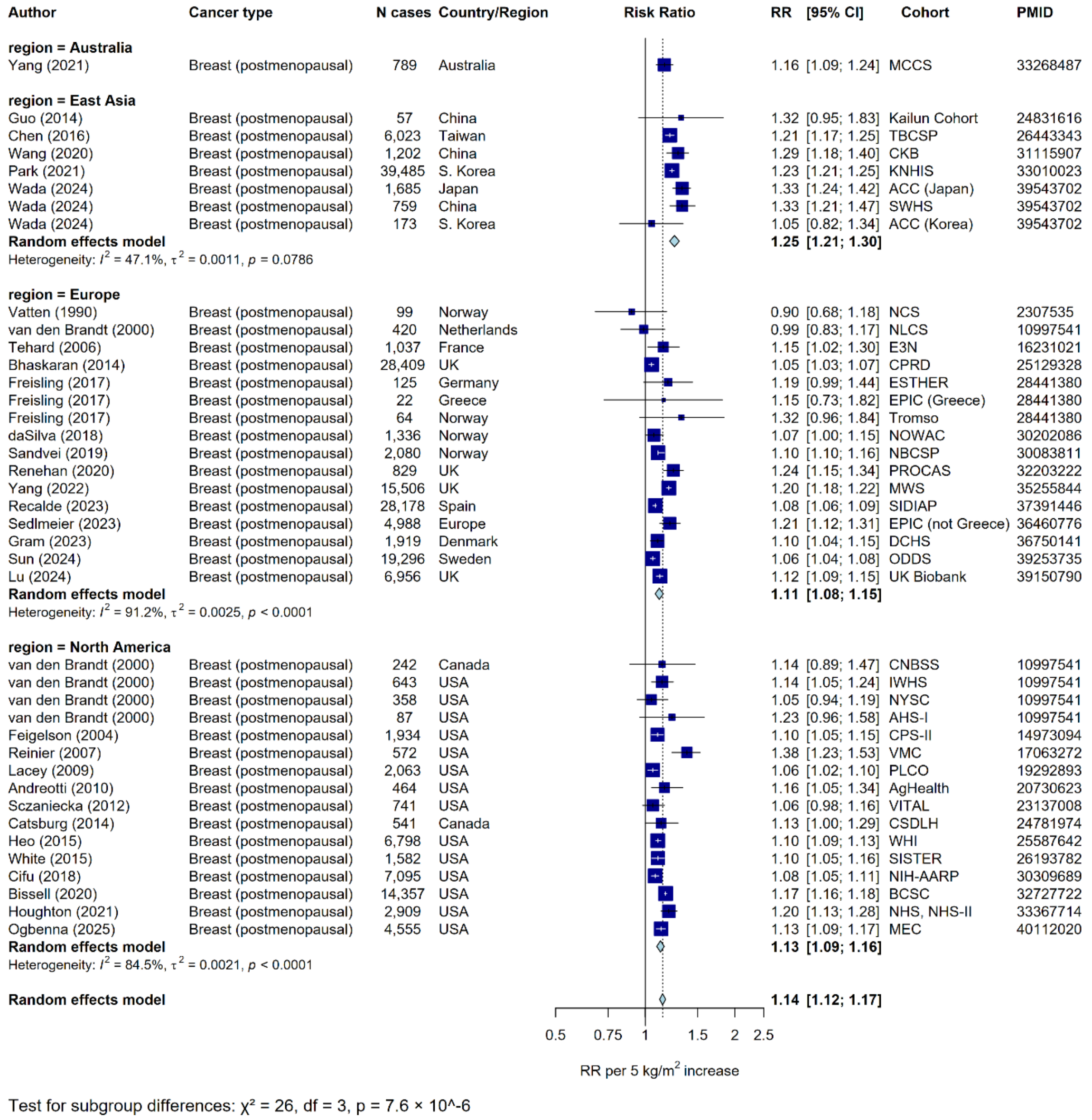

Supplementary Figure 33: Meta-analysis of prospective studies on the risk of breast cancer (postmenopausal) in relation to BMI, by region

Study-specific RRs are represented by squares (with their 95% CIs as lines). RRs were combined using random effects meta-analysis (represented by a diamond). Heterogeneity across studies was assessed using I^2 and tau^2 within region. Heterogeneity in the pooled estimates by region was assessed using the Q-statistic. Further details of model adjustments, follow-up time, analytic population for each study are available from **Supplementary Data**. Pooled estimates spanning multiple regions where region-specific RRs were not available were excluded[10].

Abbreviations: BMI=body mass index, CI=confidence interval, PMID=PubMed ID, RR=risk ratio

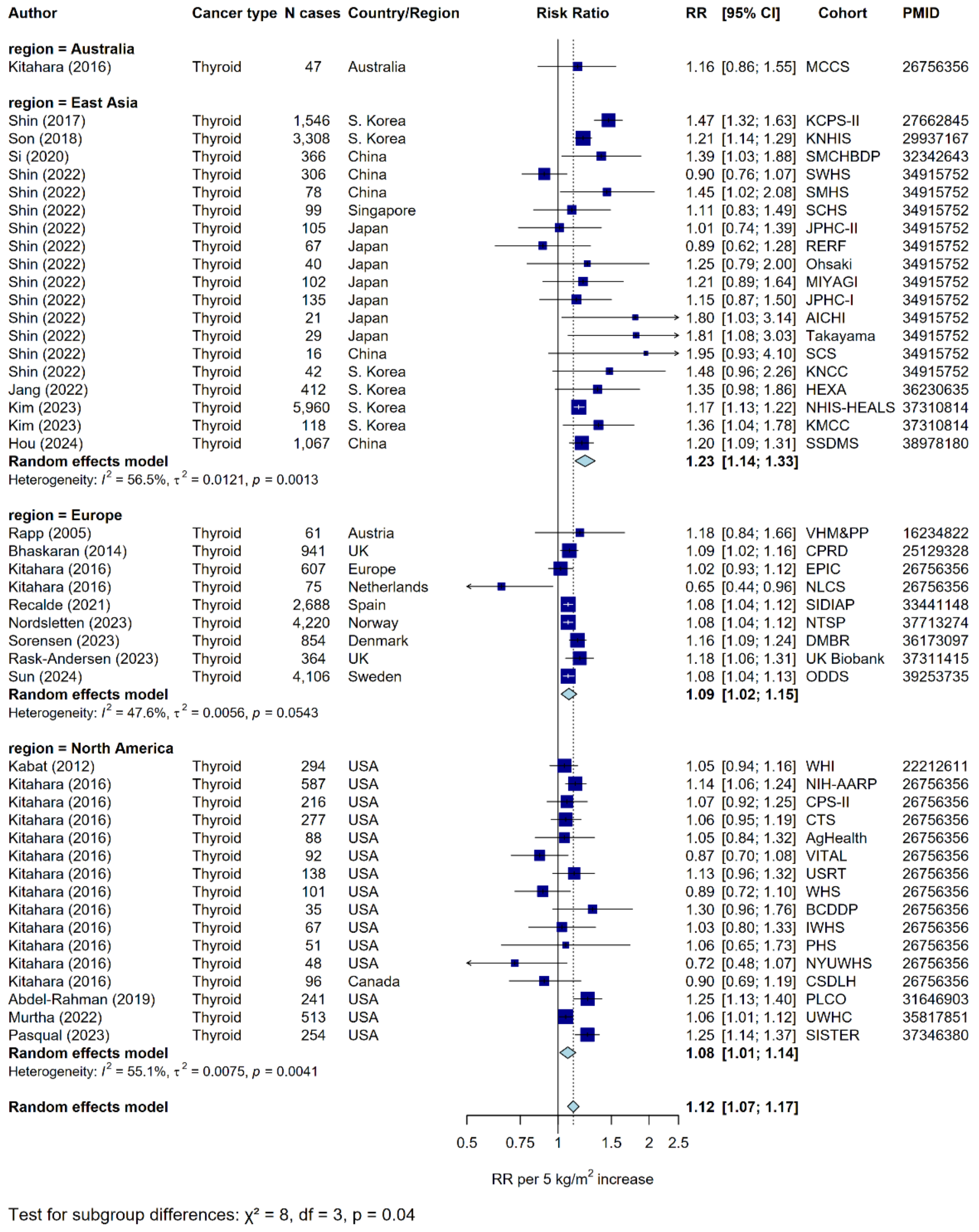

Supplementary Figure 34: Meta-analysis of prospective studies on the risk of thyroid cancer in relation to BMI, by region

Study-specific RRs are represented by squares (with their 95% CIs as lines). RRs were combined using random effects meta-analysis (represented by a diamond). Heterogeneity across studies was assessed using I^2 and tau^2 within region. Heterogeneity in the pooled estimates by region was assessed using the Q-statistic. Further details of model adjustments, follow-up time, analytic population for each study are available from **Supplementary Data**.

Abbreviations: BMI=body mass index, CI=confidence interval, PMID=PubMed ID, RR=risk ratio

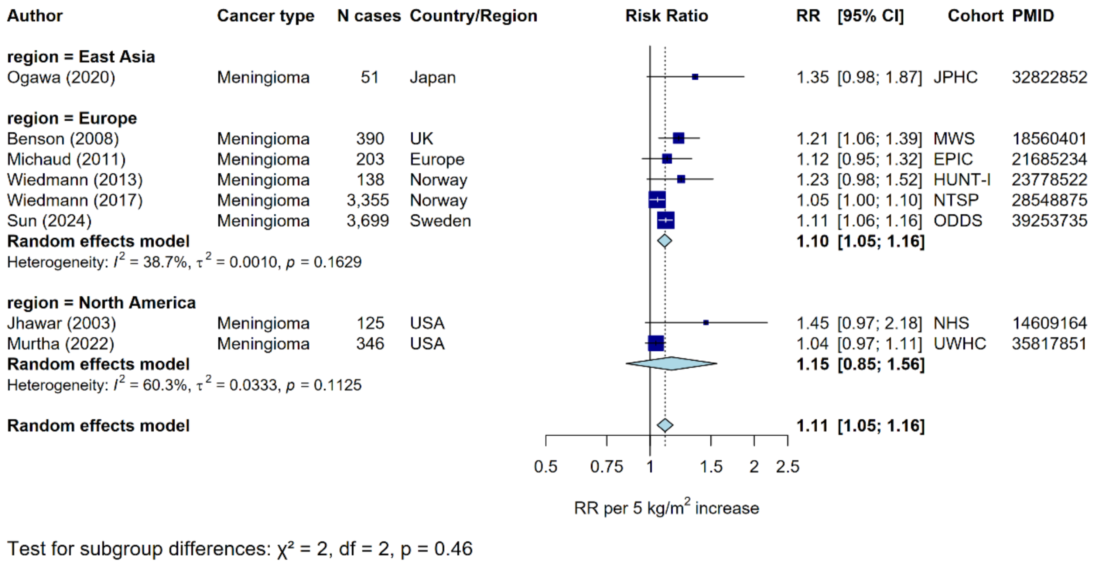

Supplementary Figure 35: Meta-analysis of prospective studies on the risk of meningioma cancer in relation to BMI, by region

Study-specific RRs are represented by squares (with their 95% CIs as lines). RRs were combined using random effects meta-analysis (represented by a diamond). Heterogeneity across studies was assessed using I^2 and tau^2 within region. Heterogeneity in the pooled estimates by region was assessed using the Q-statistic. Further details of model adjustments, follow-up time, analytic population for each study are available from **Supplementary Data**.

Abbreviations: BMI=body mass index, CI=confidence interval, PMID=PubMed ID, RR=risk ratio

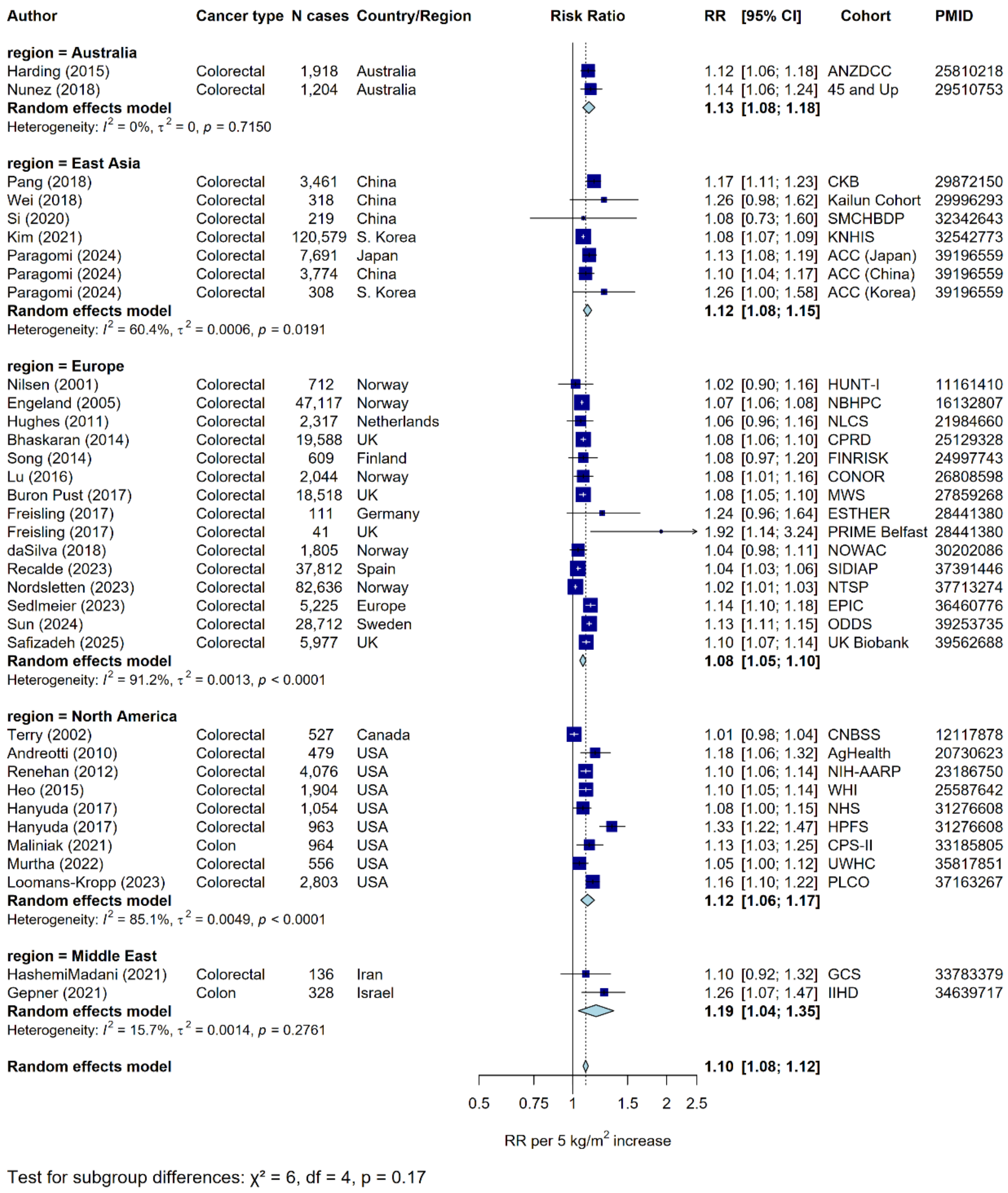

Supplementary Figure 36: Meta-analysis of prospective studies on the risk of colorectal cancer in relation to BMI, by region

Study-specific RRs are represented by squares (with their 95% CIs as lines). RRs were combined using random effects meta-analysis (represented by a diamond). Heterogeneity across studies was assessed using I^2 and tau^2 within region. Heterogeneity in the pooled estimates by region was assessed using the Q-statistic. Further details of model adjustments, follow-up time, analytic population for each study are available from **Supplementary Data**.

Abbreviations: BMI=body mass index, CI=confidence interval, PMID=PubMed ID, RR=risk ratio

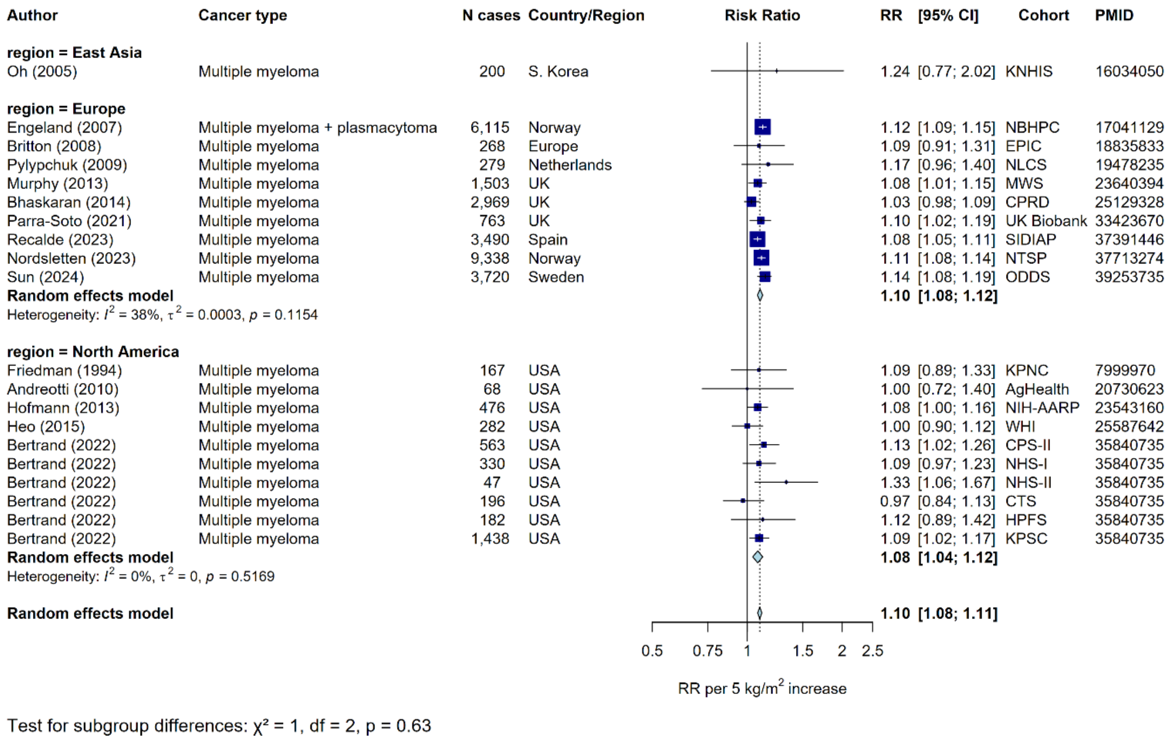

Supplementary Figure 37: Meta-analysis of prospective studies on the risk of multiple myeloma in relation to BMI, by region

Study-specific RRs are represented by squares (with their 95% CIs as lines). RRs were combined using random effects meta-analysis (represented by a diamond). Heterogeneity across studies was assessed using I^2 and tau^2 within region. Heterogeneity in the pooled estimates by region was assessed using the Q-statistic. Further details of model adjustments, follow-up time, analytic population for each study are available from **Supplementary Data**.

Abbreviations: BMI=body mass index, CI=confidence interval, PMID=PubMed ID, RR=risk ratio

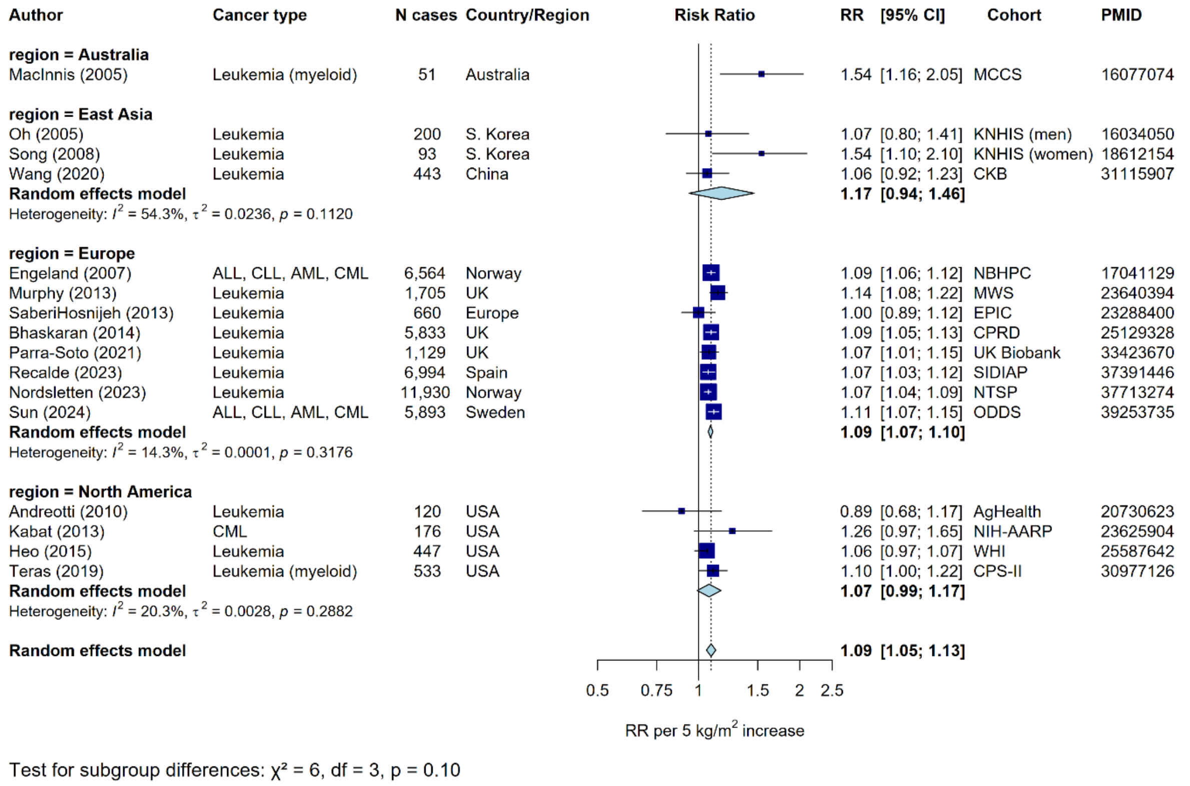

Supplementary Figure 38: Meta-analysis of prospective studies on the risk of leukaemia in relation to BMI, by region

Study-specific RRs are represented by squares (with their 95% CIs as lines). RRs were combined using random effects meta-analysis (represented by a diamond). Heterogeneity across studies was assessed using I^2 and tau^2 within region. Heterogeneity in the pooled estimates by region was assessed using the Q-statistic. Further details of model adjustments, follow-up time, analytic population for each study are available from **Supplementary Data**.

Abbreviations: ALL=acute lymphocytic leukaemia, AML=acute myeloid leukaemia, BMI=body mass index, CI=confidence interval, CLL=chronic lymphocytic leukaemia, CML=chronic myeloid leukaemia, PMID=PubMed ID, RR=risk ratio

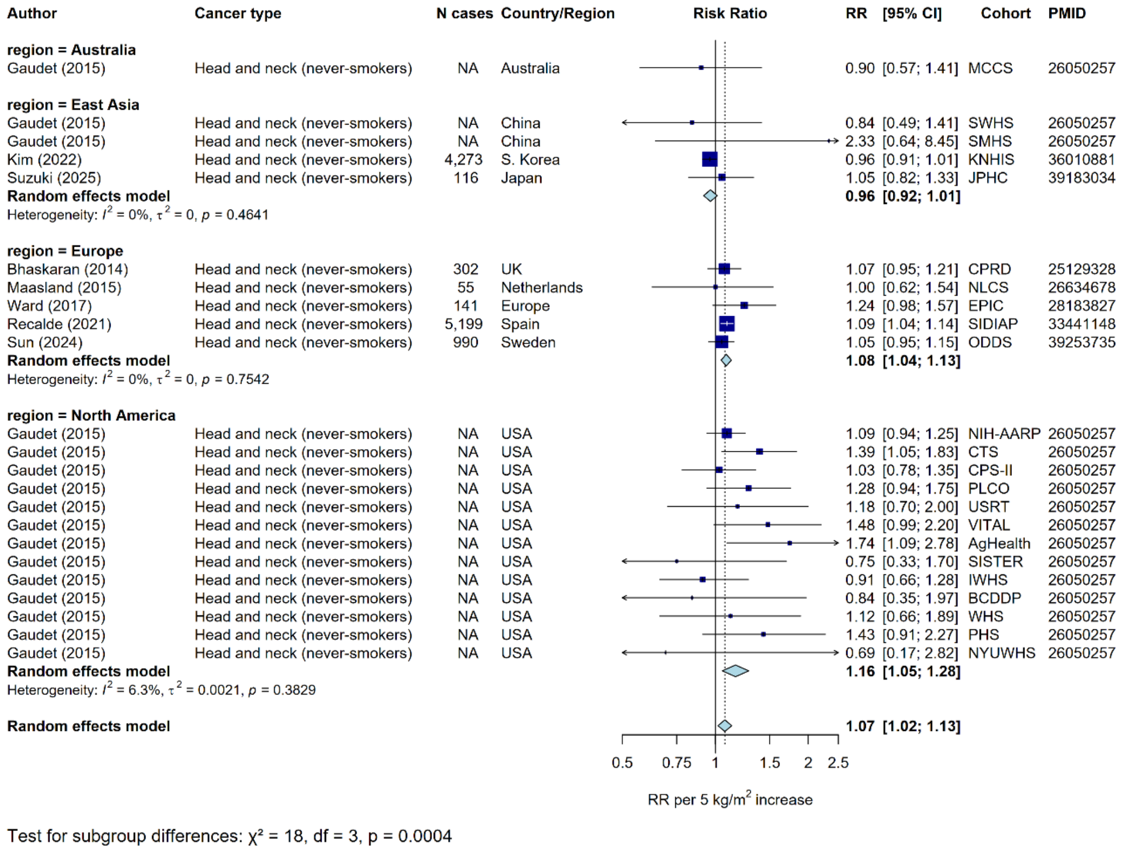

Supplementary Figure 39: Meta-analysis of prospective studies on the risk of head and neck cancer (restricted to never-smokers) in relation to BMI, by region

Study-specific RRs are represented by squares (with their 95% CIs as lines). RRs were combined using random effects meta-analysis (represented by a diamond). Heterogeneity across studies was assessed using I^2 and tau^2 within region. Heterogeneity in the pooled estimates by region was assessed using the Q-statistic. Further details of model adjustments, follow-up time, analytic population for each study are available from **Supplementary Data**. Cases numbers for never-smokers for each individual study were not specified in Gaudet et al., 2015 (total cases=796).

Abbreviations: BMI=body mass index, CI=confidence interval, PMID=PubMed ID, RR=risk ratio

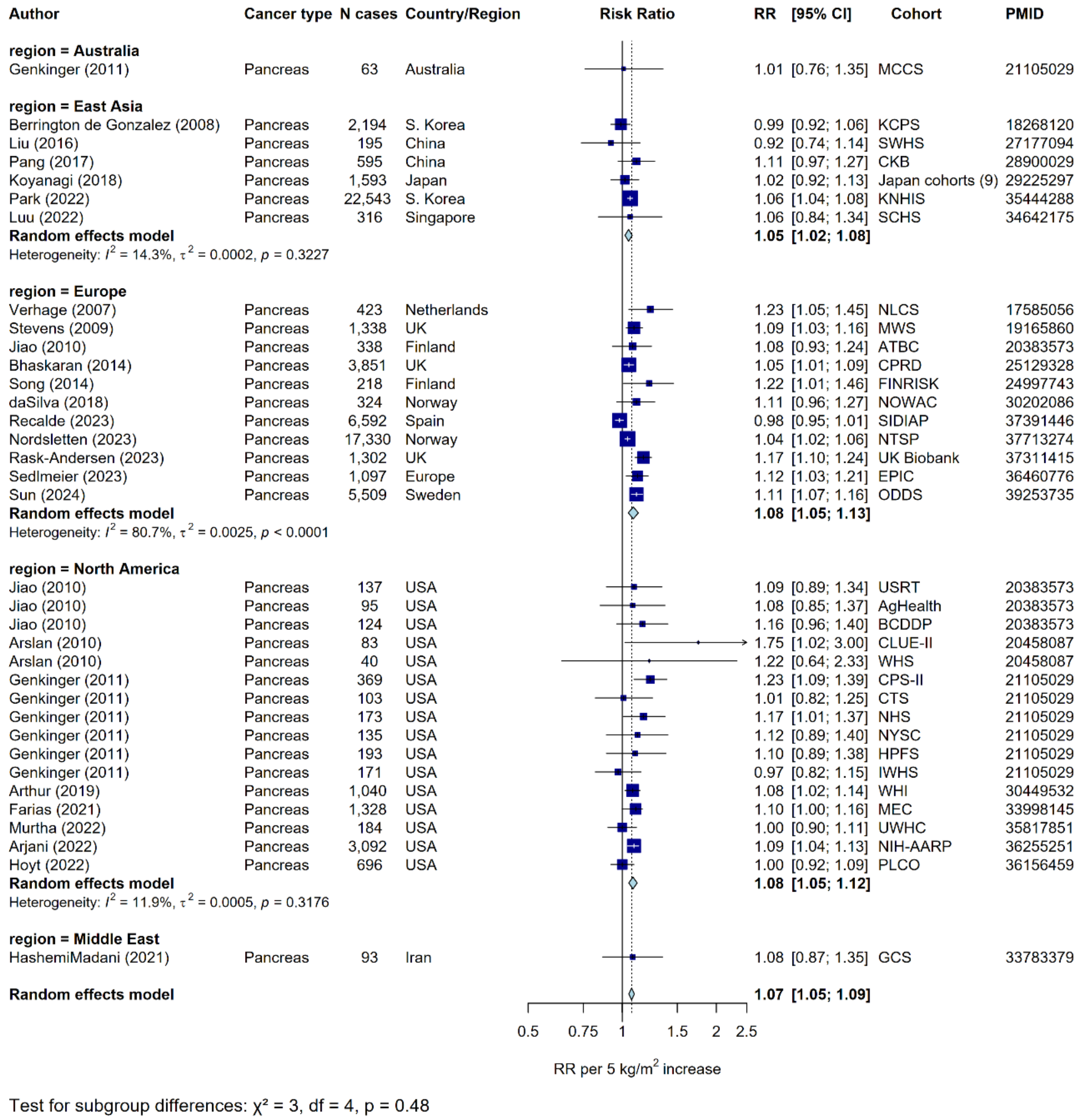

Supplementary Figure 40: Meta-analysis of prospective studies on the risk of pancreatic cancer in relation to BMI, by region

Study-specific RRs are represented by squares (with their 95% CIs as lines). RRs were combined using random effects meta-analysis (represented by a diamond). Heterogeneity across studies was assessed using I^2 and tau^2 within region. Heterogeneity in the pooled estimates by region was assessed using the Q-statistic. Further details of model adjustments, follow-up time, analytic population for each study are available from **Supplementary Data**.

Abbreviations: BMI=body mass index, CI=confidence interval, PMID=PubMed ID, RR=risk ratio

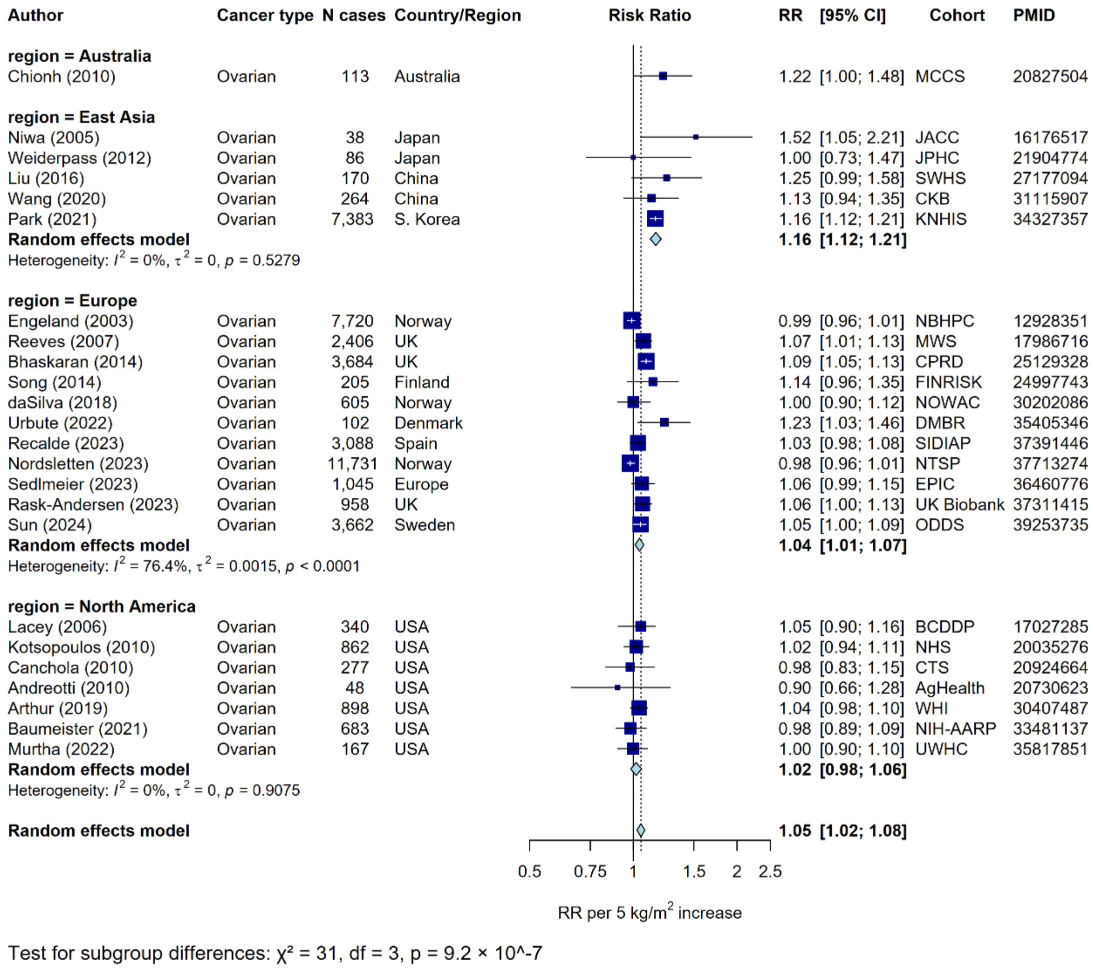

Supplementary Figure 41: Meta-analysis of prospective studies on the risk of ovarian cancer in relation to BMI, by region

Study-specific RRs are represented by squares (with their 95% CIs as lines). RRs were combined using random effects meta-analysis (represented by a diamond). Heterogeneity across studies was assessed using I^2 and tau^2 within region. Heterogeneity in the pooled estimates by region was assessed using the Q-statistic. Further details of model adjustments, follow-up time, analytic population for each study are available from **Supplementary Data**.

Abbreviations: BMI=body mass index, CI=confidence interval, PMID=PubMed ID, RR=risk ratio

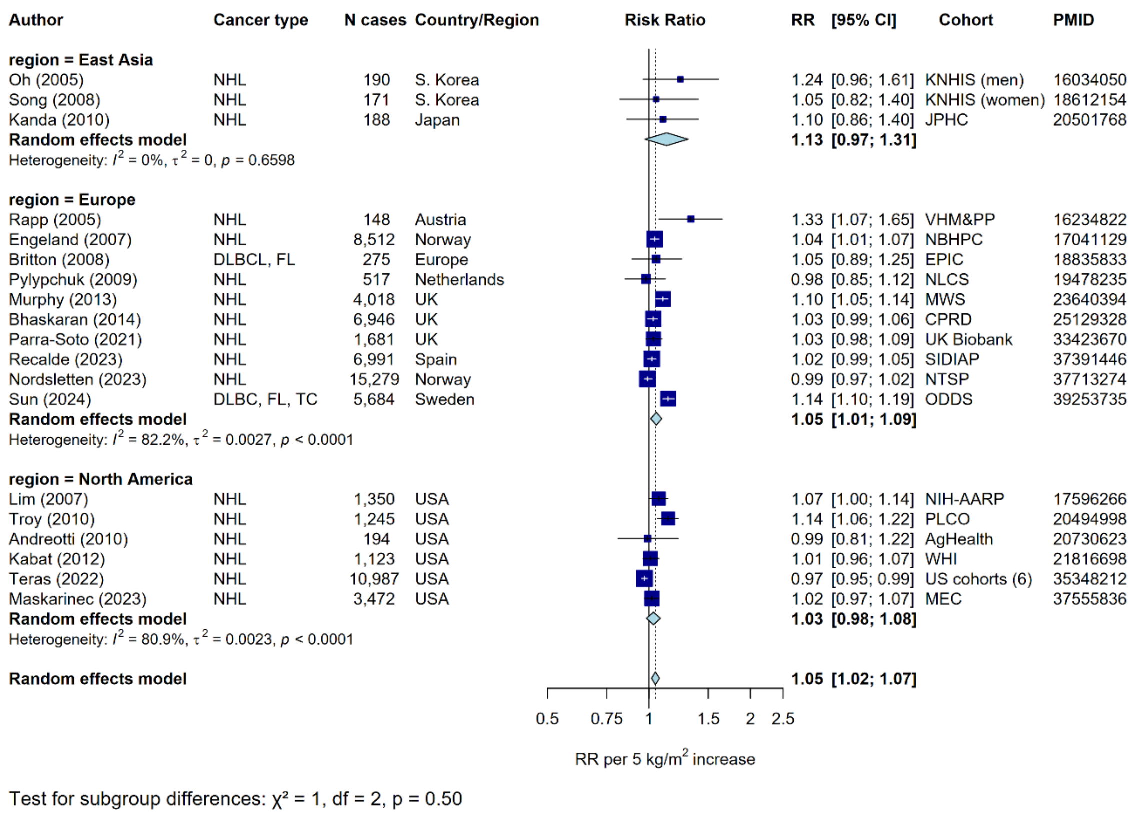

Supplementary Figure 42: Meta-analysis of prospective studies on the risk of NHL in relation to BMI, by region

Study-specific RRs are represented by squares (with their 95% CIs as lines). RRs were combined using random effects meta-analysis (represented by a diamond). Heterogeneity across studies was assessed using I^2 and tau^2 within region. Heterogeneity in the pooled estimates by region was assessed using the Q-statistic. Further details of model adjustments, follow-up time, analytic population for each study are available from **Supplementary Data**.

Abbreviations: BMI=body mass index, CI=confidence interval, DLBCL=diffuse large B-cell lymphoma, FL=follicular lymphoma, NHL=non-Hodgkin lymphoma, PMID=PubMed ID, RR=risk ratio, TC=T-cell lymphoma

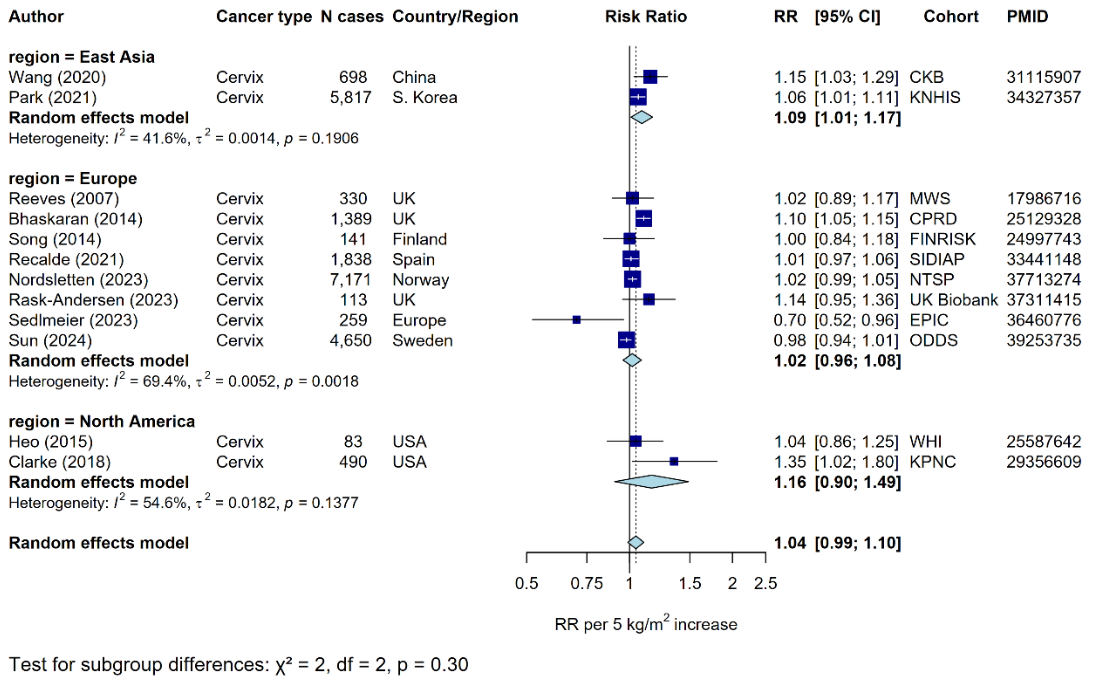

Supplementary Figure 43: Meta-analysis of prospective studies on the risk of cervical cancer in relation to BMI, by region

Study-specific RRs are represented by squares (with their 95% CIs as lines). RRs were combined using random effects meta-analysis (represented by a diamond). Heterogeneity across studies was assessed using I^2 and tau^2 within region. Heterogeneity in the pooled estimates by region was assessed using the Q-statistic. Further details of model adjustments, follow-up time, analytic population for each study are available from **Supplementary Data**.

Abbreviations: BMI=body mass index, CI=confidence interval, PMID=PubMed ID, RR=risk ratio

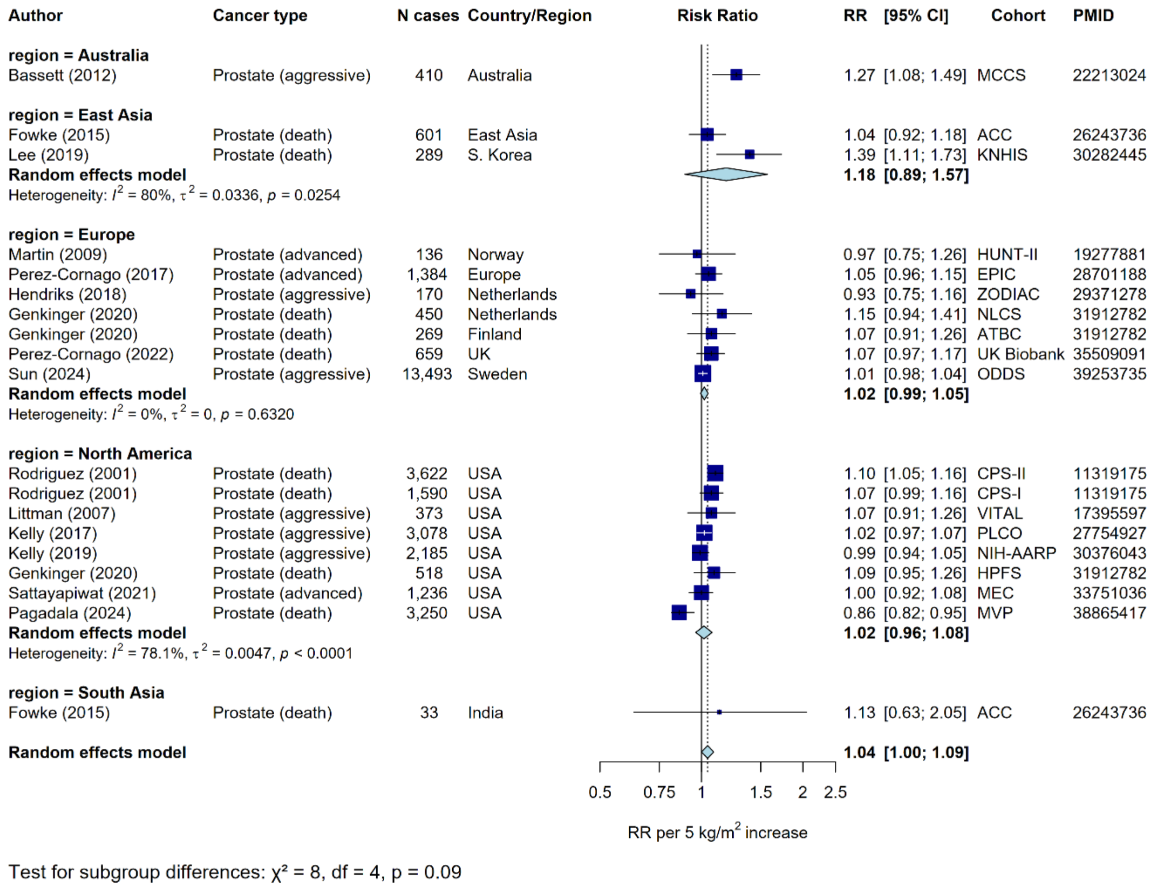

Supplementary Figure 44: Meta-analysis of prospective studies on the risk of aggressive prostate cancer in relation to BMI, by region

Aggressive prostate cancer was defined as any of stage 3-4 on the American Joint Committee on Cancer 1992 classification, advanced cancer, advanced or metastatic cancer; metastatic cancer; stage C or D on the Whitmore/Jewett scale; fatal cancer (prostate cancer-specific mortality); high stage or grade; Gleason grade ≥ 7). Study-specific RRs are represented by squares (with their 95% CIs as lines). RRs were combined using random effects meta-analysis (represented by a diamond). Heterogeneity across studies was assessed using I^2 and tau^2 within region. Heterogeneity in the pooled estimates by region was assessed using the Q-statistic. Further details of model adjustments, follow-up time, analytic population for each study are available from **Supplementary Data**.

Abbreviations: BMI=body mass index, CI=confidence interval, PMID=PubMed ID, RR=risk ratio

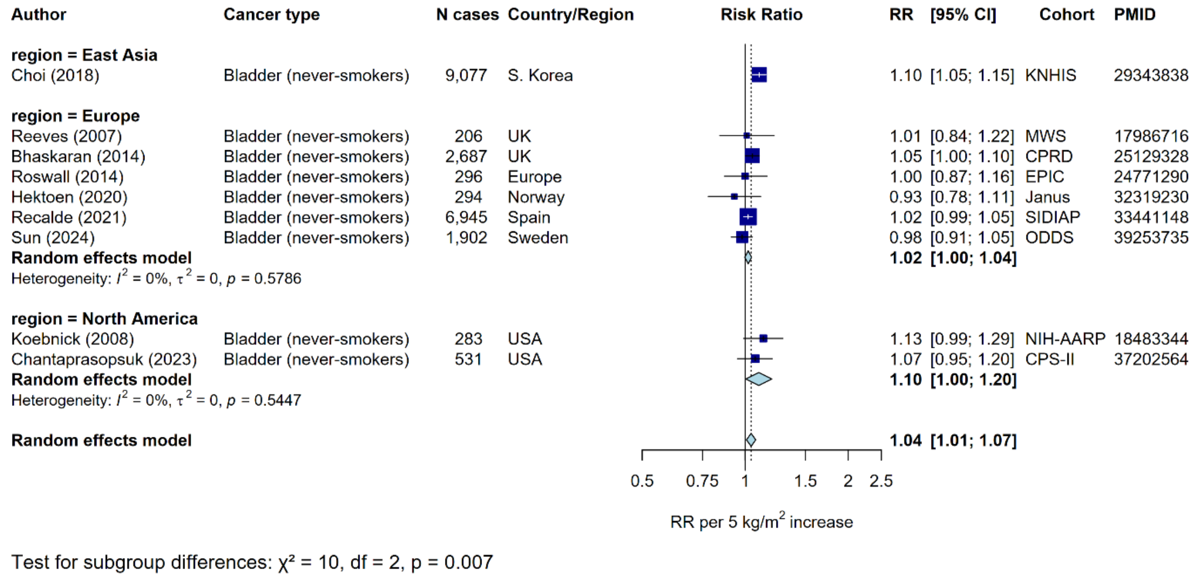

Supplementary Figure 45: Meta-analysis of prospective studies on the risk of bladder cancer (restricted to never-smokers) in relation to BMI, by region

Study-specific RRs are represented by squares (with their 95% CIs as lines). RRs were combined using random effects meta-analysis (represented by a diamond). Heterogeneity across studies was assessed using I^2 and tau^2 within region. Heterogeneity in the pooled estimates by region was assessed using the Q-statistic. Further details of model adjustments, follow-up time, analytic population for each study are available from **Supplementary Data**.

Abbreviations: BMI=body mass index, CI=confidence interval, PMID=PubMed ID, RR=risk ratio

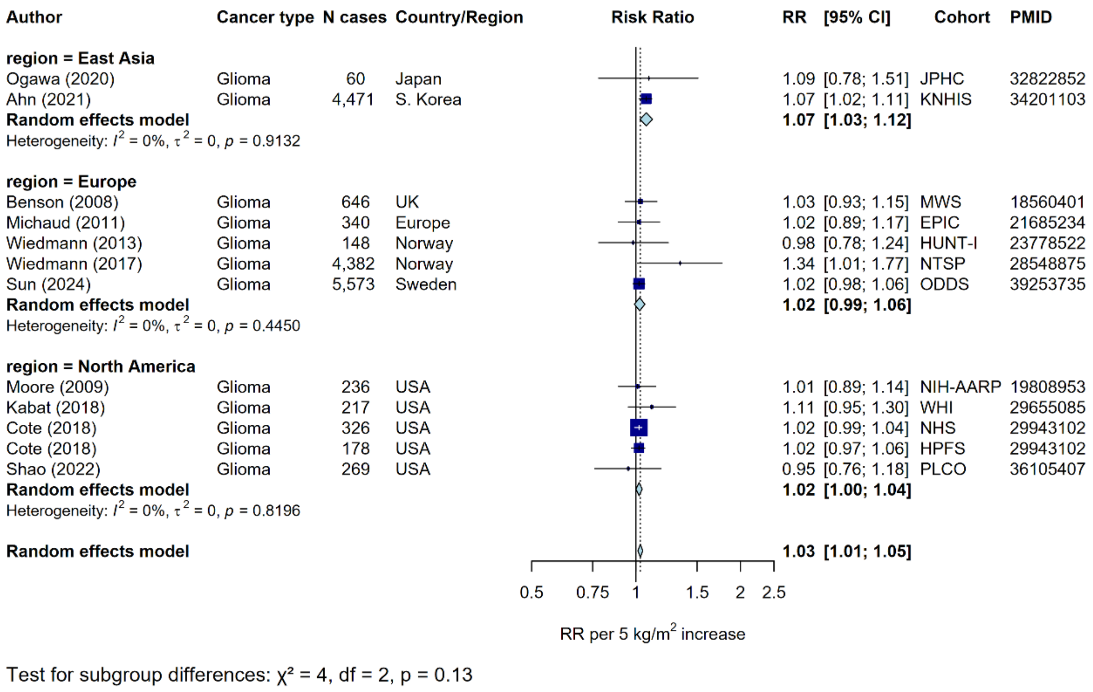

Supplementary Figure 46: Meta-analysis of prospective studies on the risk of glioma in relation to BMI, by region

Study-specific RRs are represented by squares (with their 95% CIs as lines). RRs were combined using random effects meta-analysis (represented by a diamond). Heterogeneity across studies was assessed using I^2 and tau^2 within region. Heterogeneity in the pooled estimates by region was assessed using the Q-statistic. Further details of model adjustments, follow-up time, analytic population for each study are available from **Supplementary Data**.

Abbreviations: BMI=body mass index, CI=confidence interval, PMID=PubMed ID, RR=risk ratio

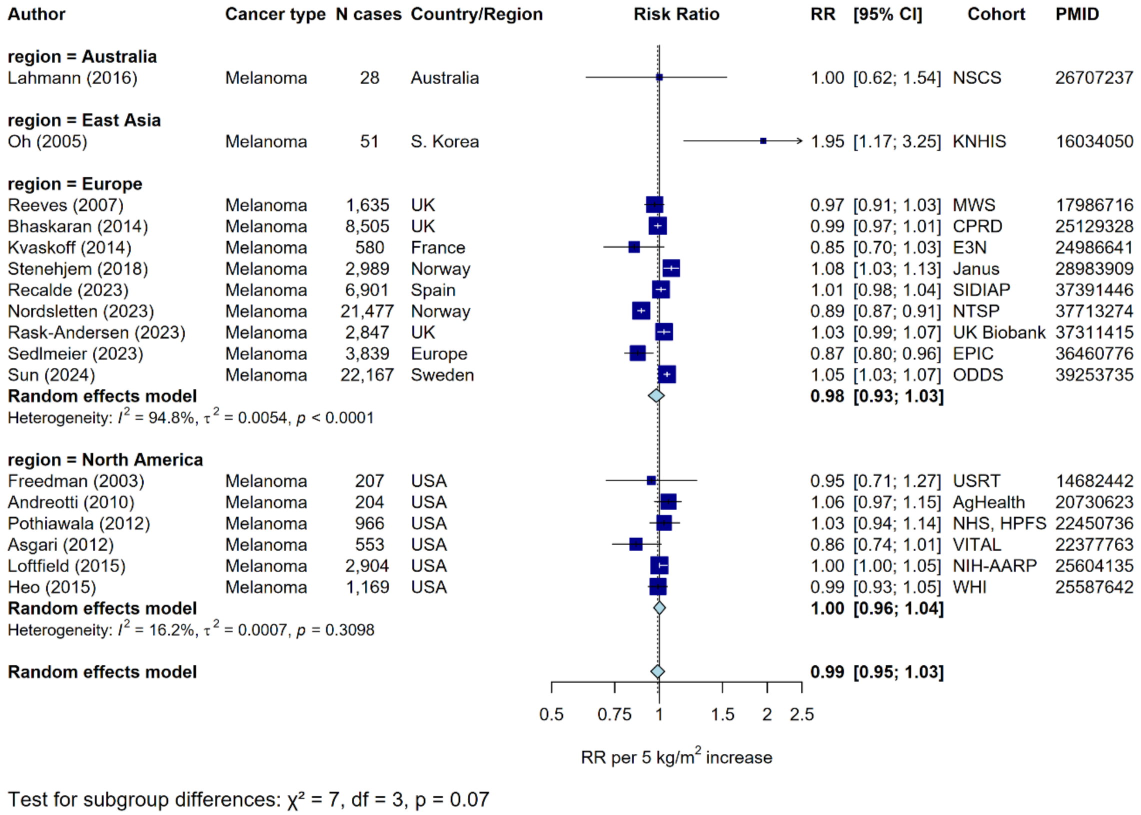

Supplementary Figure 47: Meta-analysis of prospective studies on the risk of melanoma in relation to BMI, by region

Study-specific RRs are represented by squares (with their 95% CIs as lines). RRs were combined using random effects meta-analysis (represented by a diamond). Heterogeneity across studies was assessed using I^2 and tau^2 within region. Heterogeneity in the pooled estimates by region was assessed using the Q-statistic. Further details of model adjustments, follow-up time, analytic population for each study are available from **Supplementary Data**.

Abbreviations: BMI=body mass index, CI=confidence interval, PMID=PubMed ID, RR=risk ratio

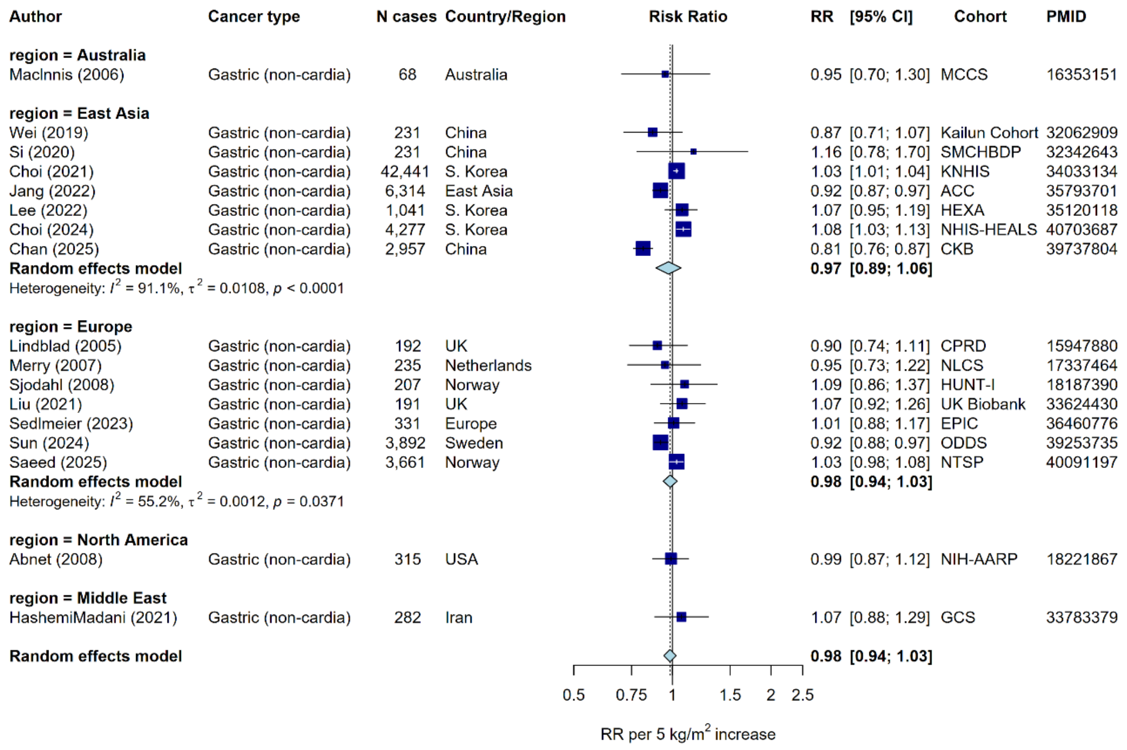

Test for subgroup differences: χ^2 = 1, df = 4, p = 0.94

Supplementary Figure 48: Meta-analysis of prospective studies on the risk of gastric cancer (non-cardia) in relation to BMI, by region

Study-specific RRs are represented by squares (with their 95% CIs as lines). RRs were combined using random effects meta-analysis (represented by a diamond). Heterogeneity across studies was assessed using I^2 and tau^2 within region. Heterogeneity in the pooled estimates by region was assessed using the Q-statistic. Further details of model adjustments, follow-up time, analytic population for each study are available from **Supplementary Data**.

Abbreviations: BMI=body mass index, CI=confidence interval, PMID=PubMed ID, RR=risk ratio

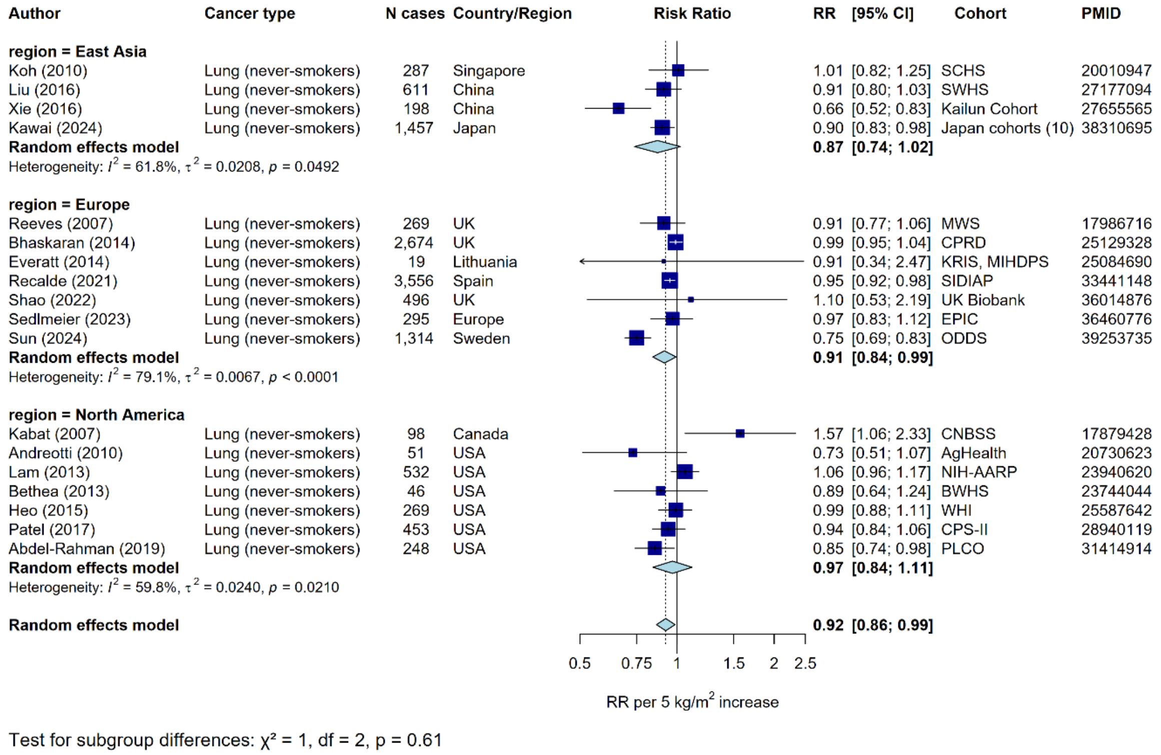

Supplementary Figure 49: Meta-analysis of prospective studies on the risk of lung cancer (restricted to never-smokers) in relation to BMI, by region

Study-specific RRs are represented by squares (with their 95% CIs as lines). RRs were combined using random effects meta-analysis (represented by a diamond). Heterogeneity across studies was assessed using I^2 and tau^2 within region. Heterogeneity in the pooled estimates by region was assessed using the Q-statistic. Further details of model adjustments, follow-up time, analytic population for each study are available from **Supplementary Data**.

Abbreviations: BMI=body mass index, CI=confidence interval, PMID=PubMed ID, RR=risk ratio

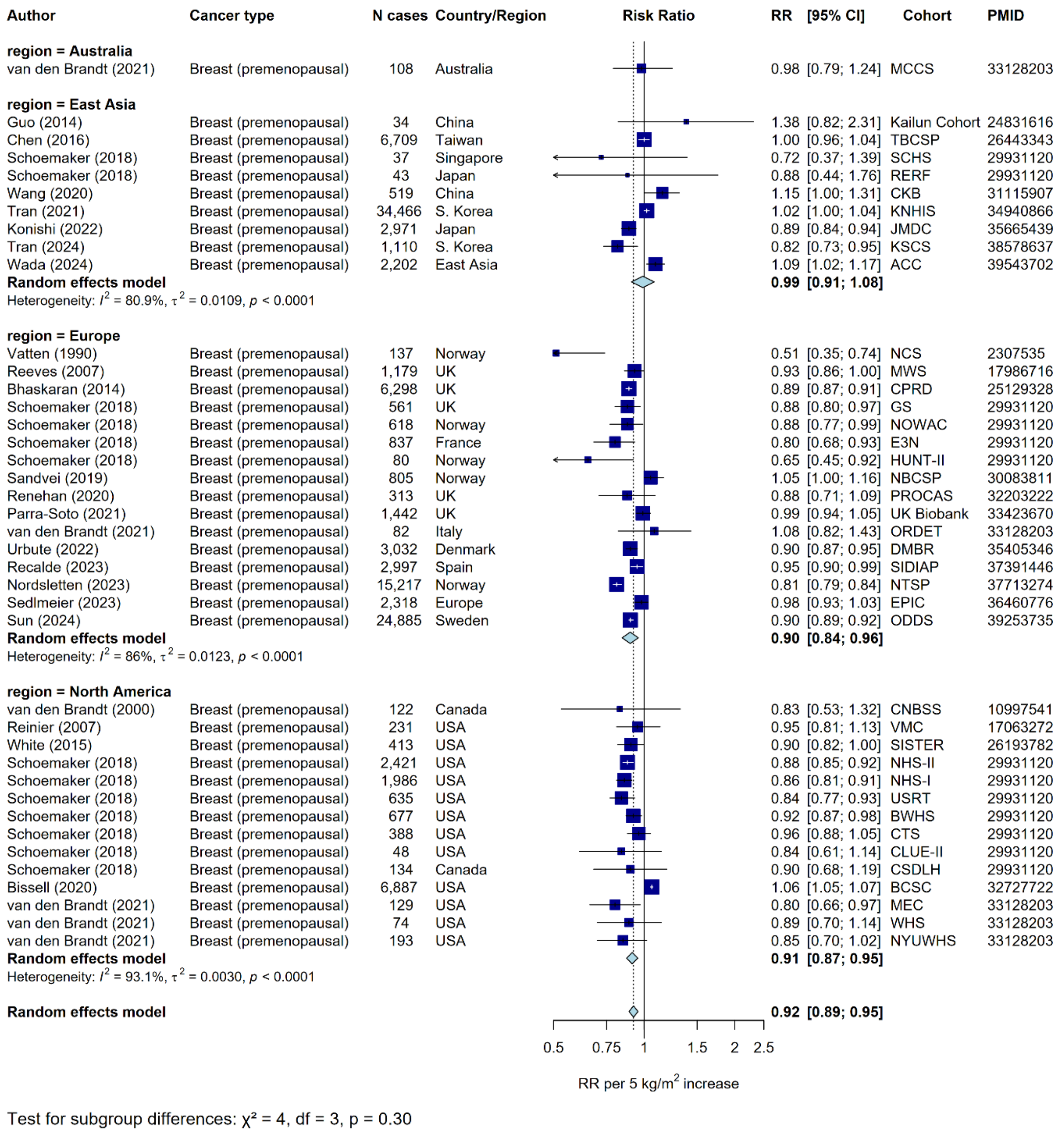

Supplementary Figure 50: Meta-analysis of prospective studies on the risk of breast cancer (premenopausal) in relation to BMI, by region

Study-specific RRs are represented by squares (with their 95% CIs as lines). RRs were combined using random effects meta-analysis (represented by a diamond). Heterogeneity across studies was assessed using I^2 and tau^2 within region. Heterogeneity in the pooled estimates by region was assessed using the Q-statistic. Further details of model adjustments, follow-up time, analytic population for each study are available from **Supplementary Data**.

Abbreviations: BMI=body mass index, CI=confidence interval, PMID=PubMed ID, RR=risk ratio

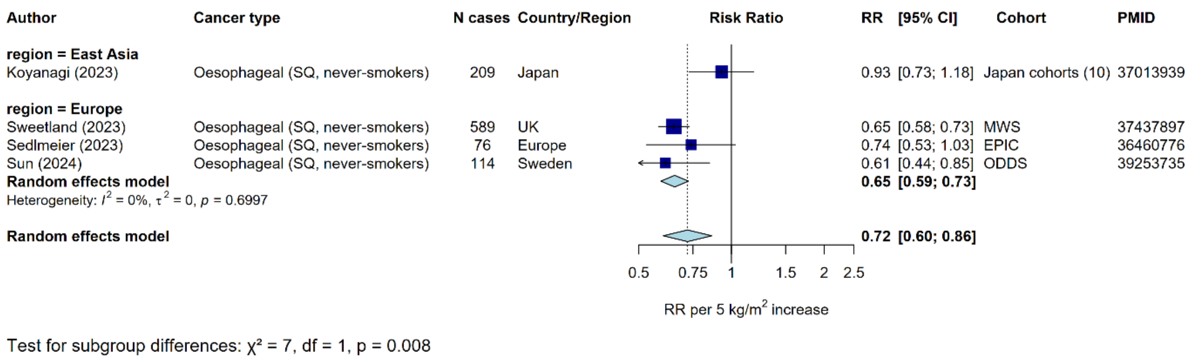

Supplementary Figure 51: Meta-analysis of prospective studies on the risk of oesophageal SQ in relation to BMI, by region

Study-specific RRs are represented by squares (with their 95% CIs as lines). RRs were combined using random effects meta-analysis (represented by a diamond). Heterogeneity across studies was assessed using I^2 and tau^2 within region. Heterogeneity in the pooled estimates by region was assessed using the Q-statistic. Further details of model adjustments, follow-up time, analytic population for each study are available from **Supplementary Data**.

Abbreviations: BMI=body mass index, CI=confidence interval, PMID=PubMed ID, RR=risk ratio, SQ=squamous cell carcinoma.

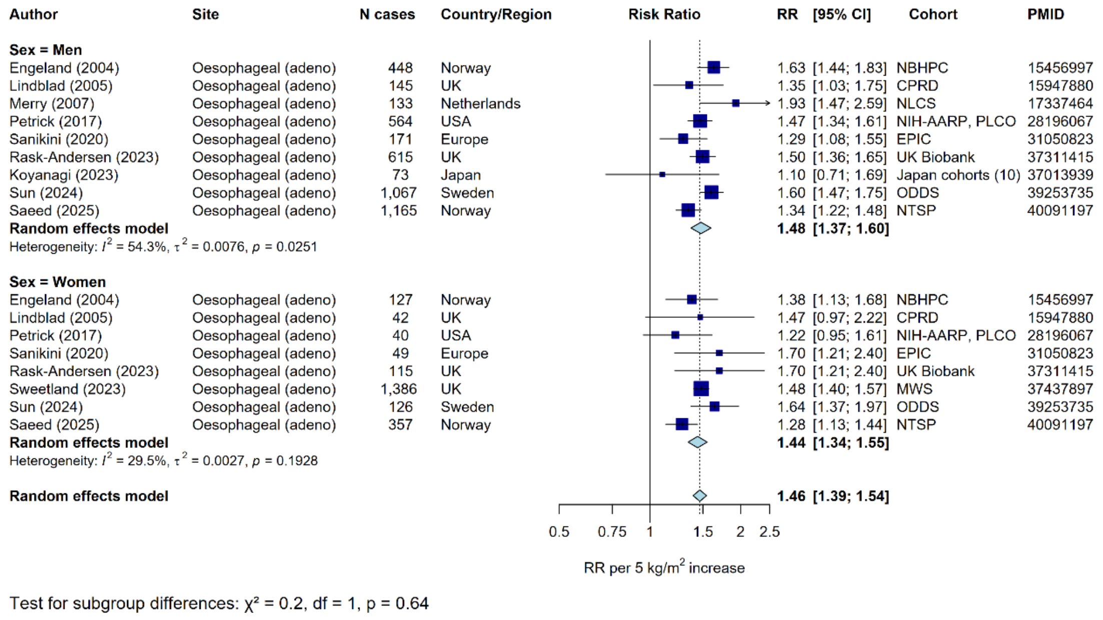

Supplementary Figure 52: Meta-analysis of prospective studies on the risk of oesophageal adenocarcinoma in relation to BMI, by sex

Study-specific RRs are represented by squares (with their 95% CIs as lines). RRs were combined using random effects meta-analysis (represented by a diamond). Heterogeneity across studies was assessed using I^2 and tau^2 within sex. Heterogeneity in the pooled estimates by region was assessed using the Q-statistic. Further details of model adjustments, follow-up time, analytic population for each study are available from **Supplementary Data**.

Abbreviations: BMI=body mass index, CI=confidence interval, PMID=PubMed ID, RR=risk ratio

Author	Site	N cases	Country/Region	RR	[95% CI]	Cohort	PMID
**Sex = Men**							
Bjørge (2004)	Renal cell carcinoma	3,821	Norway	1.28	[1.22; 1.34]	NBHPC	15583369
Setiawan (2007)	Renal cell carcinoma	220	USA	1.34	[1.10; 1.54]	MEC	17656615
Adams (2008)	Renal cell carcinoma	1,022	USA	1.32	[1.23; 1.42]	NIH-AARP	18544571
Andreotti (2010)	Kidney	78	USA	1.00	[0.70; 1.47]	AgHealth	20730623
Sawada (2010)	Kidney	101	Japan	1.18	[0.83; 1.68]	JPHC	20470974
Song (2014)	Kidney	107	Finland	1.51	[1.18; 1.95]	FINRISK	24997743
Guo (2014)	Kidney	69	China	1.28	[0.91; 1.80]	Kailun Cohort	24831616
Nam (2019)	Kidney	12,537	S. Korea	1.40	[1.35; 1.46]	KNHIS	31231120
Everatt (2019)	Kidney	79	Lithuania	1.40	[1.05; 1.93]	KRIS, MIHDPS	31951685
Graff (2022)	Renal cell carcinoma	291	USA	1.28	[1.05; 1.56]	HPFS	35715363
Rask-Andersen (2023)	Kidney	935	UK	1.32	[1.22; 1.42]	UK Biobank	37311415
Nordsletten (2023)	Kidney	7,713	Norway	1.25	[1.21; 1.30]	NTSP	37713274
Sun (2024)	Renal cell carcinoma	4,554	Sweden	1.44	[1.38; 1.50]	ODDS	39253735
**Random effects model**				**1.33**	**[1.29; 1.38]**		
Heterogeneity: $I^2 = 69.4\%$, $\tau^2 = 0.0014$, $p < 0.0001$							
**Sex = Women**							
Bjørge (2004)	Renal cell carcinoma	2,632	Norway	1.28	[1.22; 1.34]	NBHPC	15583369
Setiawan (2007)	Renal cell carcinoma	127	USA	1.40	[1.22; 1.54]	MEC	17656615
Reeves (2007)	Kidney	723	UK	1.24	[1.13; 1.36]	MWS	17986716
Adams (2008)	Renal cell carcinoma	344	USA	1.36	[1.24; 1.49]	NIH-AARP	18544571
Andreotti (2010)	Kidney	28	USA	1.10	[0.77; 1.61]	AgHealth	20730623
Sawada (2010)	Kidney	38	Japan	1.16	[0.71; 1.90]	JPHC	20470974
Song (2014)	Kidney	88	Finland	1.11	[0.81; 1.53]	FINRISK	24997743
Sanfilippo (2014)	Kidney	407	USA	1.22	[1.11; 1.33]	WHI	24637660
Liu (2016)	Renal cell carcinoma	131	China	1.26	[0.97; 1.63]	SWHS	27177094
daSilva (2018)	Kidney	211	Norway	1.33	[1.15; 1.54]	NOWAC	30202086
Nam (2019)	Kidney	7,989	S. Korea	1.20	[1.14; 1.25]	KNHIS	31231120
Graff (2022)	Renal cell carcinoma	110	USA	1.45	[1.19; 1.75]	NHS-II	35715363
Graff (2022)	Renal cell carcinoma	394	USA	1.41	[1.25; 1.59]	NHS	35715363
Rask-Andersen (2023)	Kidney	527	UK	1.43	[1.31; 1.55]	UK Biobank	37311415
Nordsletten (2023)	Kidney	5,420	Norway	1.24	[1.20; 1.28]	NTSP	37713274
Sun (2024)	Renal cell carcinoma	1,639	Sweden	1.46	[1.38; 1.54]	ODDS	39253735
**Random effects model**				**1.31**	**[1.26; 1.36]**		
Heterogeneity: $I^2 = 70.8\%$, $\tau^2 = 0.0027$, $p < 0.0001$							
**Random effects model**				**1.32**	**[1.28; 1.35]**		

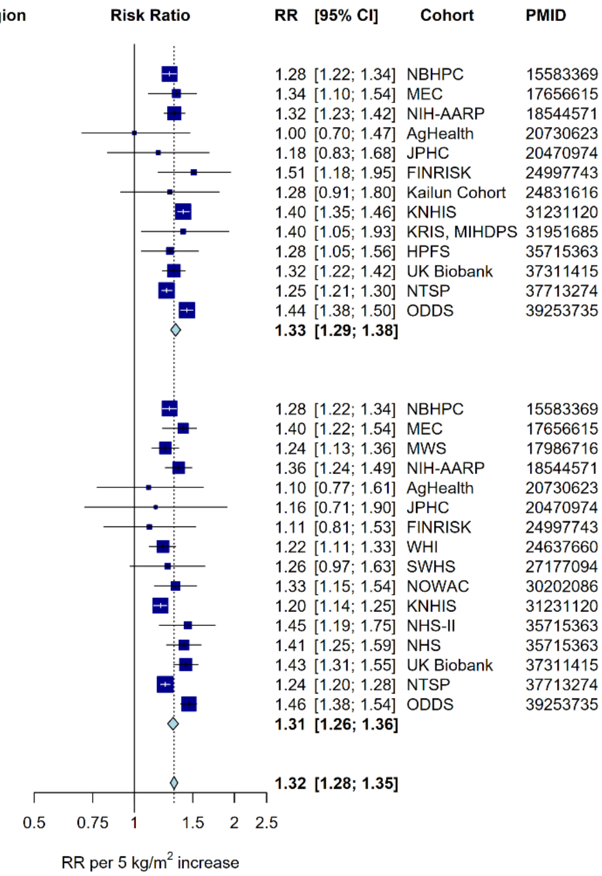

Test for subgroup differences: $\chi^2 = 0.5$, df = 1, p = 0.50

Supplementary Figure 53: Meta-analysis of prospective studies on the risk of kidney cancer in relation to BMI, by sex

Study-specific RRs are represented by squares (with their 95% CIs as lines). RRs were combined using random effects meta-analysis (represented by a diamond). Heterogeneity across studies was assessed using I^2 and tau^2 within sex. Heterogeneity in the pooled estimates by region was assessed using the Q-statistic. Further details of model adjustments, follow-up time, analytic population for each study are available from **Supplementary Data**.

Abbreviations: BMI=body mass index, CI=confidence interval, PMID=PubMed ID, RR=risk ratio

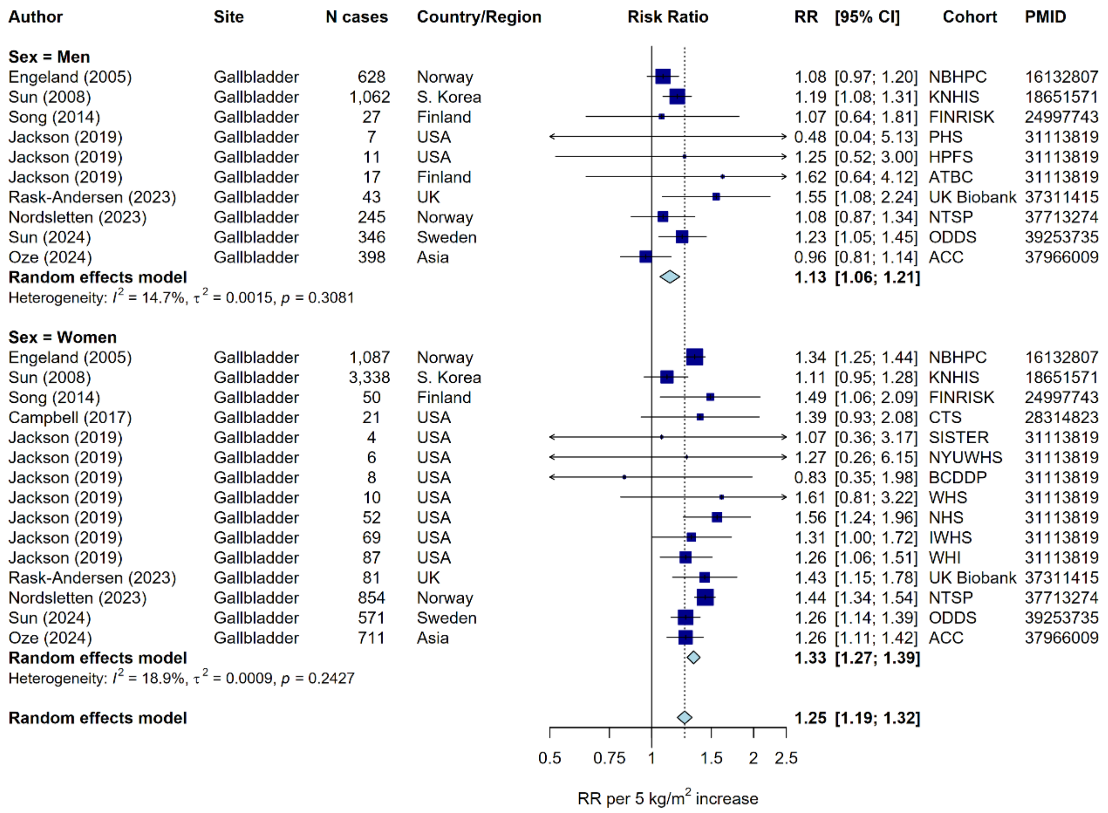

Test for subgroup differences: χ² = 15.2, df = 1, p = 9.5 × 10^-5

Supplementary Figure 54: Meta-analysis of prospective studies on the risk of gallbladder cancer in relation to BMI, by sex

Study-specific RRs are represented by squares (with their 95% CIs as lines). RRs were combined using random effects meta-analysis (represented by a diamond). Heterogeneity across studies was assessed using I^2 and tau^2 within sex. Heterogeneity in the pooled estimates by region was assessed using the Q-statistic. Further details of model adjustments, follow-up time, analytic population for each study are available from **Supplementary Data**.

Abbreviations: BMI=body mass index, CI=confidence interval, PMID=PubMed ID, RR=risk ratio

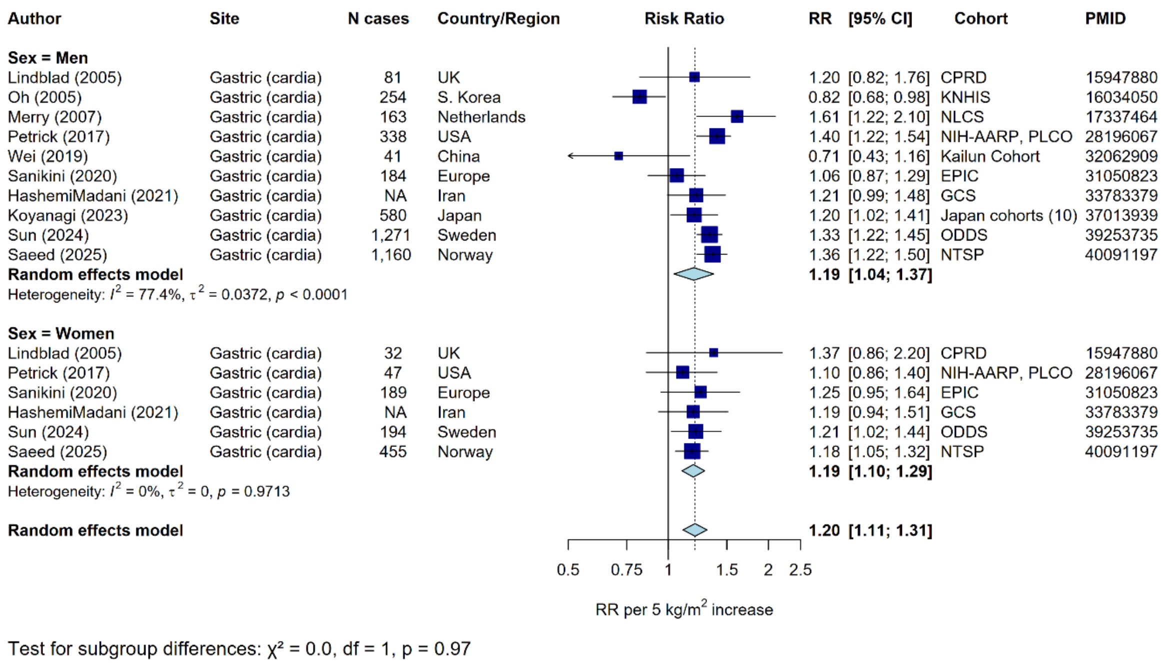

Supplementary Figure 55: Meta-analysis of prospective studies on the risk of gastric (cardia) cancer in relation to BMI, by sex

Study-specific RRs are represented by squares (with their 95% CIs as lines). RRs were combined using random effects meta-analysis (represented by a diamond). Heterogeneity across studies was assessed using I^2 and tau^2 within sex. Heterogeneity in the pooled estimates by region was assessed using the Q-statistic. Further details of model adjustments, follow-up time, analytic population for each study are available from **Supplementary Data**.

Abbreviations: BMI=body mass index, CI=confidence interval, PMID=PubMed ID, RR=risk ratio

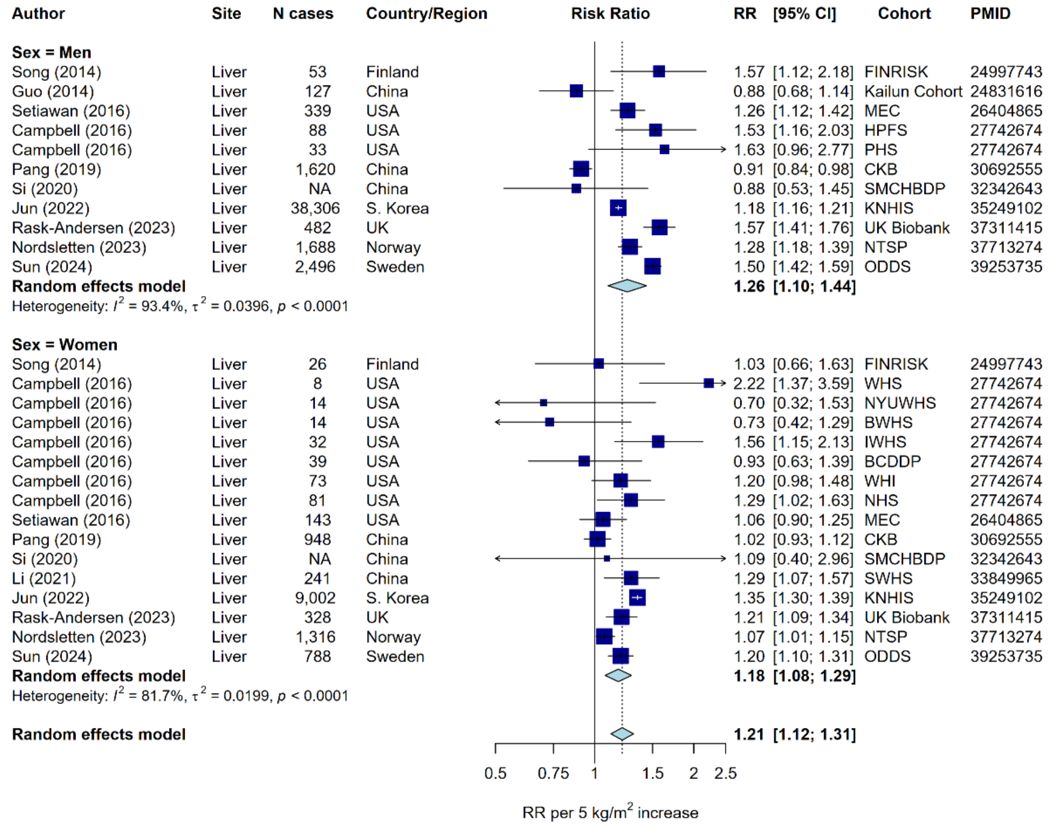

Test for subgroup differences: χ^2 = 0.6, df = 1, p = 0.45

Supplementary Figure 56: Meta-analysis of prospective studies on the risk of liver cancer in relation to BMI, by sex

Study-specific RRs are represented by squares (with their 95% CIs as lines). RRs were combined using random effects meta-analysis (represented by a diamond). Heterogeneity across studies was assessed using I^2 and tau^2 within sex. Heterogeneity in the pooled estimates by region was assessed using the Q-statistic. Further details of model adjustments, follow-up time, analytic population for each study are available from **Supplementary Data**.

Abbreviations: BMI=body mass index, CI=confidence interval, PMID=PubMed ID, RR=risk ratio

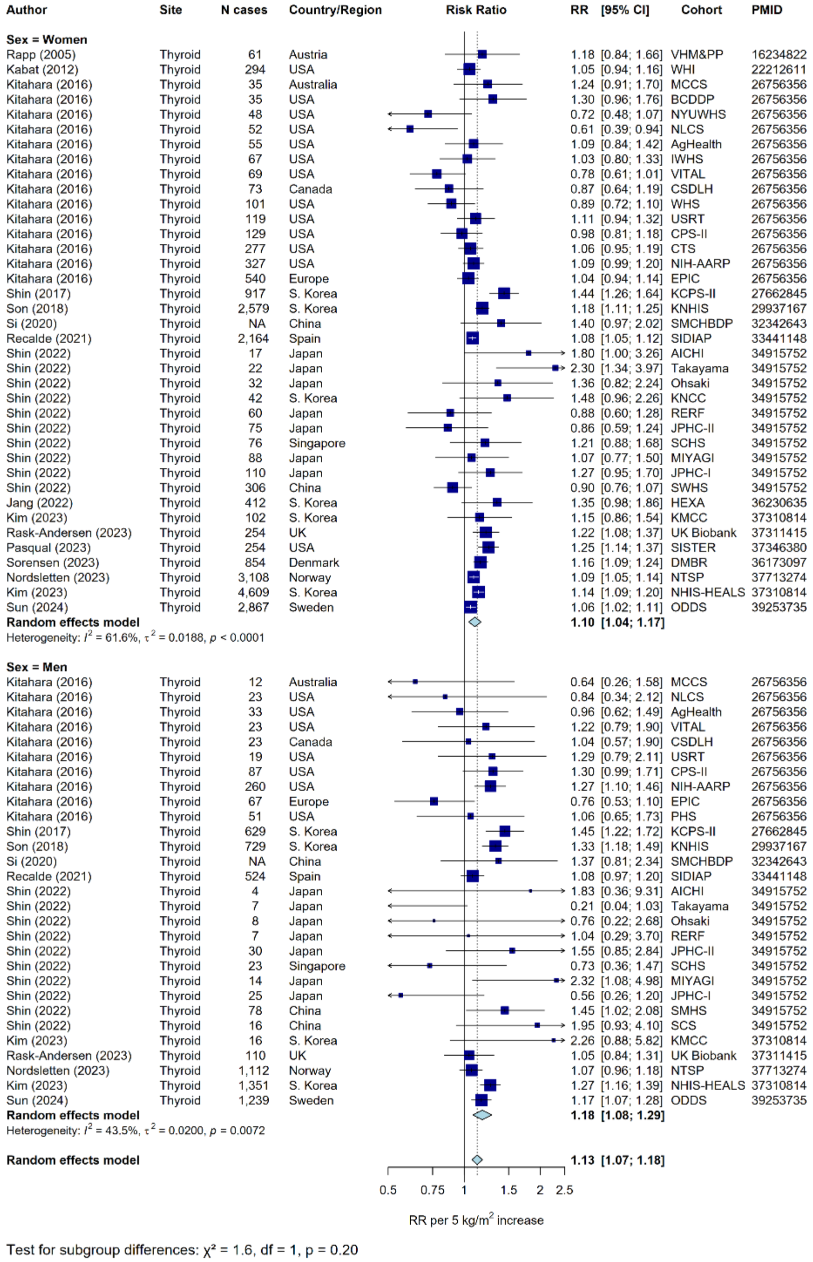

Supplementary Figure 57: Meta-analysis of prospective studies on the risk of thyroid cancer in relation to BMI, by sex

Study-specific RRs are represented by squares (with their 95% CIs as lines). RRs were combined using random effects meta-analysis (represented by a diamond). Heterogeneity across studies was assessed using I^2 and tau^2 within sex. Heterogeneity in the pooled estimates by region was assessed using the Q-statistic. Further details of model adjustments, follow-up time, analytic population for each study are available from **Supplementary Data**.

Abbreviations: BMI=body mass index, CI=confidence interval, PMID=PubMed ID, RR=risk ratio

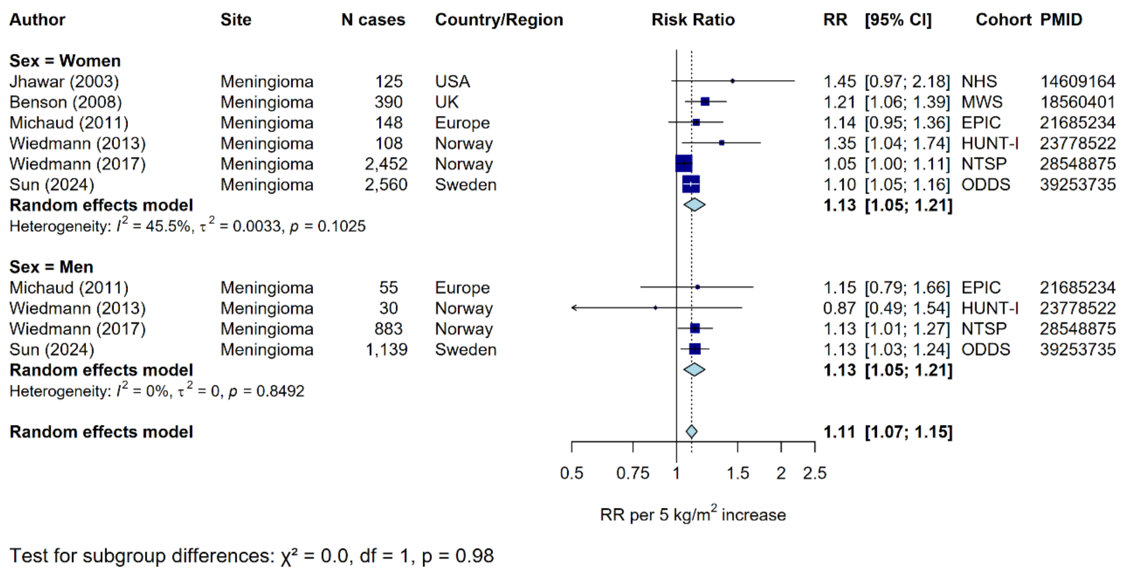

Supplementary Figure 58: Meta-analysis of prospective studies on the risk of meningioma cancer in relation to BMI, by sex

Study-specific RRs are represented by squares (with their 95% CIs as lines). RRs were combined using random effects meta-analysis (represented by a diamond). Heterogeneity across studies was assessed using I^2 and tau^2 within sex. Heterogeneity in the pooled estimates by region was assessed using the Q-statistic. Further details of model adjustments, follow-up time, analytic population for each study are available from **Supplementary Data**.

Abbreviations: BMI=body mass index, CI=confidence interval, PMID=PubMed ID, RR=risk ratio

Author	Site	N cases	Country/Region	RR	[95% CI]	Cohort	PMID
**Sex = Men**							
Nilsen (2001)	Colorectal	354	Norway	1.07	[0.88; 1.29]	HUNT-I	11161410
Engeland (2005)	Colorectal	22,987	Norway	1.15	[1.13; 1.17]	NBHPC	16132807
Andreotti (2010)	Colorectal	332	USA	1.29	[1.11; 1.49]	AgHealth	20730623
Hughes (2011)	Colorectal	1,211	Netherlands	1.25	[1.05; 1.46]	NLCS	21984660
Renehan (2012)	Colorectal	2,832	USA	1.14	[1.08; 1.20]	NIH-AARP	23186750
Song (2014)	Colorectal	303	Finland	0.95	[0.81; 1.11]	FINRISK	24997743
Harding (2015)	Colorectal	1,204	Australia	1.19	[1.11; 1.29]	ANZDCC	25810218
Lu (2016)	Colorectal	1,101	Norway	1.14	[1.02; 1.26]	CONOR	26808598
Hanyuda (2017)	Colorectal	963	USA	1.33	[1.22; 1.47]	HPFS	31276608
Pang (2018)	Colorectal	1,737	China	1.23	[1.14; 1.33]	CKB	29872150
Wei (2018)	Colorectal	318	China	1.26	[0.98; 1.62]	Kailun Cohort	29996293
Si (2020)	Colorectal	NA	China	1.12	[0.70; 1.80]	SMCHBDP	32342643
HashemiMadani (2021)	Colorectal	NA	Iran	1.05	[0.79; 1.39]	GCS	33783379
Nordsletten (2023)	Colorectal	39,809	Norway	1.12	[1.10; 1.14]	NTSP	37713274
Paragomi (2024)	Colorectal	164	S. Korea	1.26	[0.97; 1.65]	ACC (Korea)	39196559
Paragomi (2024)	Colorectal	2,048	China	1.13	[1.06; 1.21]	ACC (China)	39196559
Paragomi (2024)	Colorectal	4,507	Japan	1.18	[1.12; 1.24]	ACC (Japan)	39196559
Sun (2024)	Colorectal	18,422	Sweden	1.18	[1.15; 1.20]	ODDS	39253735
Safizadeh (2025)	Colorectal	3,421	UK	1.17	[1.13; 1.22]	UK Biobank	39562688
**Random effects model**				**1.17**	**[1.14; 1.20]**		
Heterogeneity: I^2 = 55.1%, τ^2 = 0.0013, p = 0.0020							
**Sex = Women**							
Nilsen (2001)	Colorectal	358	Norway	0.99	[0.83; 1.17]	HUNT-I	11161410
Terry (2002)	Colorectal	527	Canada	1.01	[0.98; 1.04]	CNBSS	12117878
Engeland (2005)	Colorectal	24,130	Norway	1.02	[1.01; 1.04]	NBHPC	16132807
Andreotti (2010)	Colorectal	147	USA	1.01	[0.83; 1.22]	AgHealth	20730623
Hughes (2011)	Colorectal	1,106	Netherlands	0.98	[0.88; 1.10]	NLCS	21984660
Renehan (2012)	Colorectal	1,244	USA	1.05	[0.99; 1.11]	NIH-AARP	23186750
Song (2014)	Colorectal	306	Finland	1.19	[1.04; 1.37]	FINRISK	24997743
Harding (2015)	Colorectal	714	Australia	1.06	[0.98; 1.14]	ANZDCC	25810218
Heo (2015)	Colorectal	1,904	USA	1.10	[1.05; 1.14]	WHI	25587642
Lu (2016)	Colorectal	943	Norway	1.06	[0.95; 1.19]	CONOR	26808598
Hanyuda (2017)	Colorectal	1,054	USA	1.08	[1.00; 1.15]	NHS	31276608
Buron Pust (2017)	Colorectal	18,518	UK	1.08	[1.05; 1.10]	MWS	27859268
Pang (2018)	Colorectal	1,724	China	1.12	[1.04; 1.20]	CKB	29872150
daSilva (2018)	Colorectal	1,805	Norway	1.04	[0.98; 1.11]	NOWAC	30202086
Si (2020)	Colorectal	NA	China	1.01	[0.50; 2.03]	SMCHBDP	32342643
Maliniak (2021)	Colon	964	USA	1.13	[1.03; 1.25]	CPS-II	33185805
HashemiMadani (2021)	Colorectal	NA	Iran	1.13	[0.89; 1.44]	GCS	33783379
Nordsletten (2023)	Colorectal	42,827	Norway	0.97	[0.97; 0.98]	NTSP	37713274
Paragomi (2024)	Colorectal	144	S. Korea	1.25	[0.96; 1.63]	ACC (Korea)	39196559
Paragomi (2024)	Colorectal	1,726	China	1.06	[0.98; 1.13]	ACC (China)	39196559
Paragomi (2024)	Colorectal	3,184	Japan	1.09	[1.03; 1.15]	ACC (Japan)	39196559
Sun (2024)	Colorectal	10,290	Sweden	1.08	[1.06; 1.11]	ODDS	39253735
Safizadeh (2025)	Colorectal	2,556	UK	1.08	[1.03; 1.14]	UK Biobank	39562688
**Random effects model**				**1.06**	**[1.04; 1.08]**		
Heterogeneity: I^2 = 92.3%, τ^2 = 0.0010, p < 0.0001							
**Random effects model**				**1.10**	**[1.08; 1.13]**		

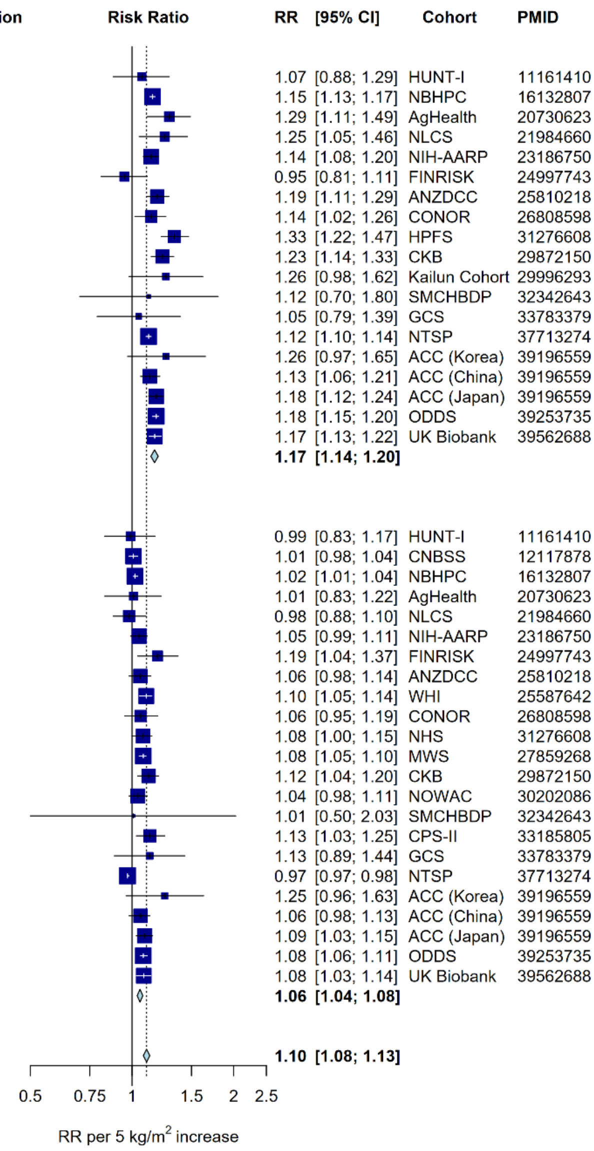

Test for subgroup differences: χ² = 37.5, df = 1, p = 8.9 × 10^-10

Supplementary Figure 59: Meta-analysis of prospective studies on the risk of colorectal cancer in relation to BMI, by sex

Study-specific RRs are represented by squares (with their 95% CIs as lines). RRs were combined using random effects meta-analysis (represented by a diamond). Heterogeneity across studies was assessed using I^2 and tau^2 within sex. Heterogeneity in the pooled estimates by region was assessed using the Q-statistic. Further details of model adjustments, follow-up time, analytic population for each study are available from **Supplementary Data**.

Abbreviations: BMI=body mass index, CI=confidence interval, PMID=PubMed ID, RR=risk ratio

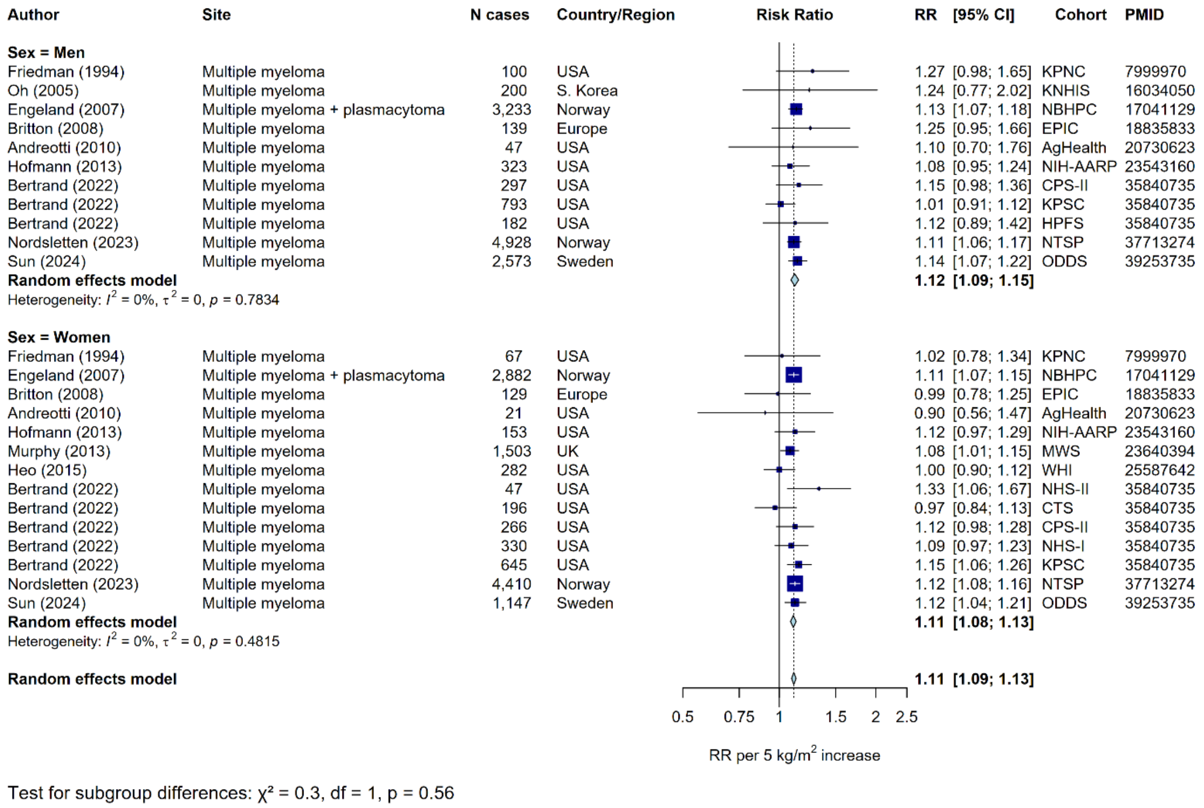

Supplementary Figure 60: Meta-analysis of prospective studies on the risk of multiple myeloma in relation to BMI, by sex

Study-specific RRs are represented by squares (with their 95% CIs as lines). RRs were combined using random effects meta-analysis (represented by a diamond). Heterogeneity across studies was assessed using I^2 and tau^2 within sex. Heterogeneity in the pooled estimates by region was assessed using the Q-statistic. Further details of model adjustments, follow-up time, analytic population for each study are available from **Supplementary Data**.

Abbreviations: BMI=body mass index, CI=confidence interval, PMID=PubMed ID, RR=risk ratio

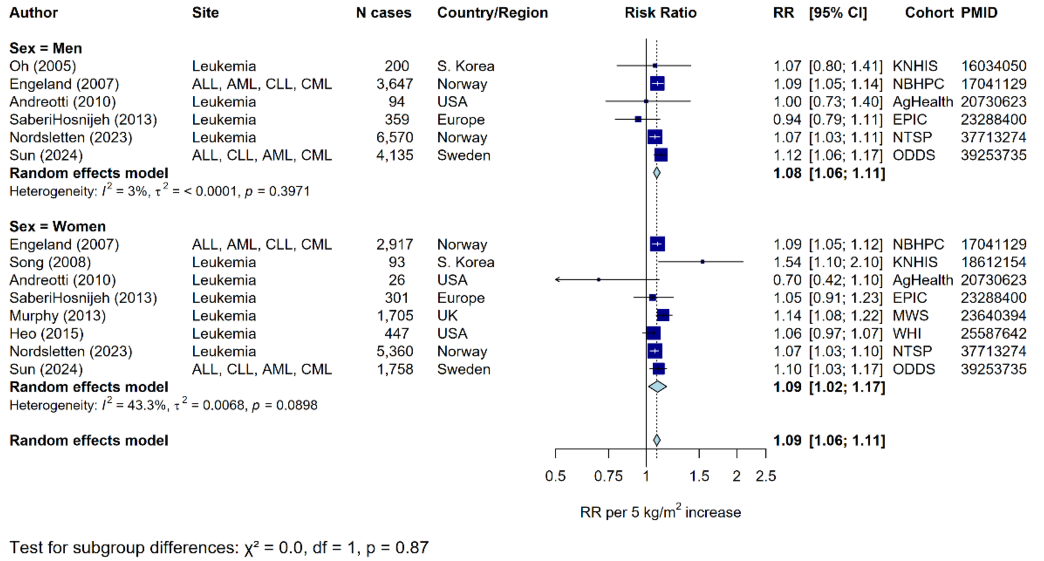

Supplementary Figure 61: Meta-analysis of prospective studies on the risk of leukaemia in relation to BMI, by sex

Study-specific RRs are represented by squares (with their 95% CIs as lines). RRs were combined using random effects meta-analysis (represented by a diamond). Heterogeneity across studies was assessed using I^2 and tau^2 within sex. Heterogeneity in the pooled estimates by region was assessed using the Q-statistic. Further details of model adjustments, follow-up time, analytic population for each study are available from **Supplementary Data**.

Abbreviations: AML=acute myeloid leukaemia, ALL=acute lymphoblastic leukaemia, BMI=body mass index, CI=confidence interval, CLL=chronic lymphocytic leukaemia, CML=chronic myeloid leukaemia, PMID=PubMed ID, RR=risk ratio

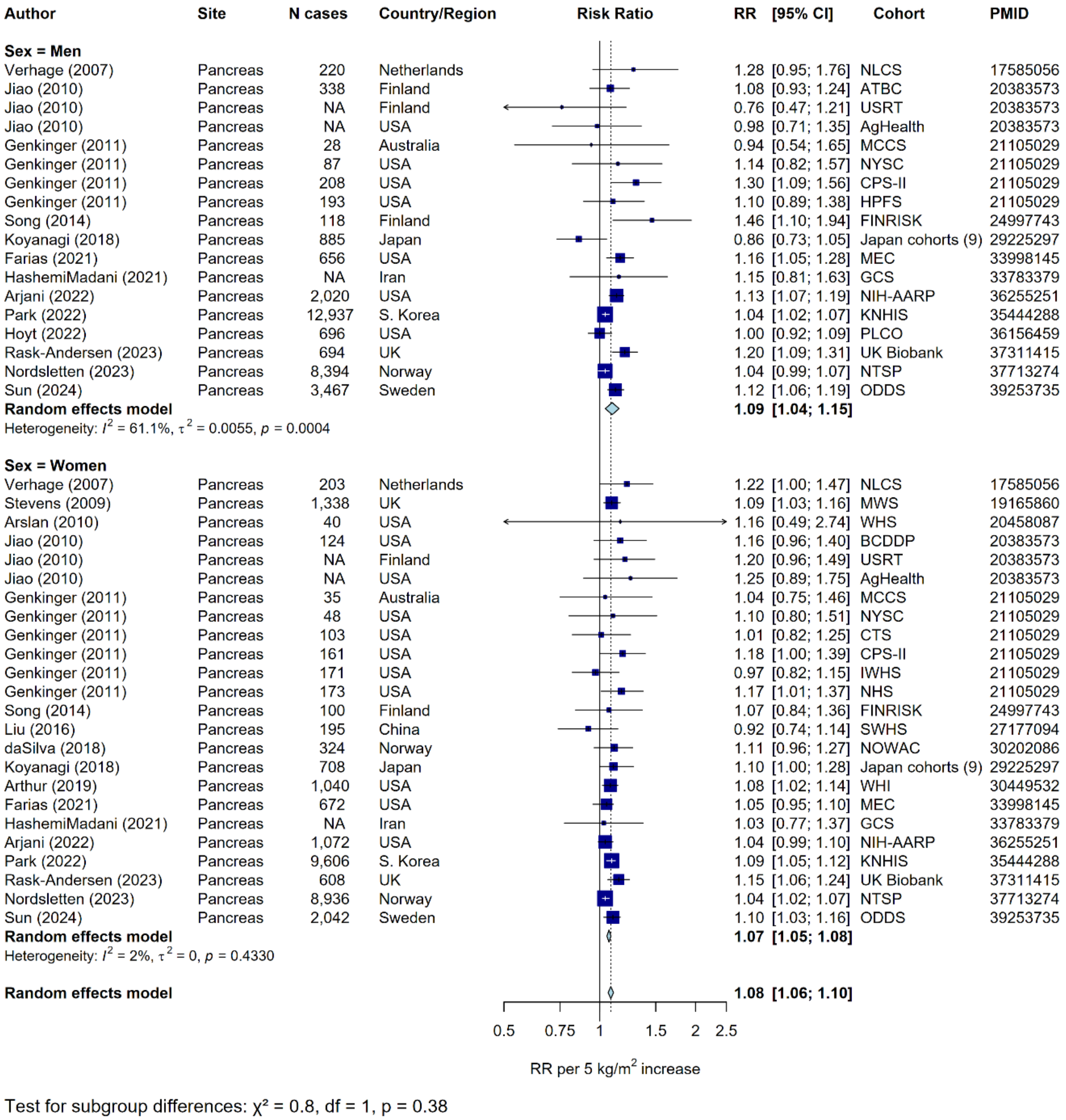

Supplementary Figure 62: Meta-analysis of prospective studies on the risk of pancreatic cancer in relation to BMI, by sex

Study-specific RRs are represented by squares (with their 95% CIs as lines). RRs were combined using random effects meta-analysis (represented by a diamond). Heterogeneity across studies was assessed using I^2 and tau^2 within sex. Heterogeneity in the pooled estimates by region was assessed using the Q-statistic. Further details of model adjustments, follow-up time, analytic population for each study are available from **Supplementary Data**.

Abbreviations: BMI=body mass index, CI=confidence interval, PMID=PubMed ID, RR=risk ratio

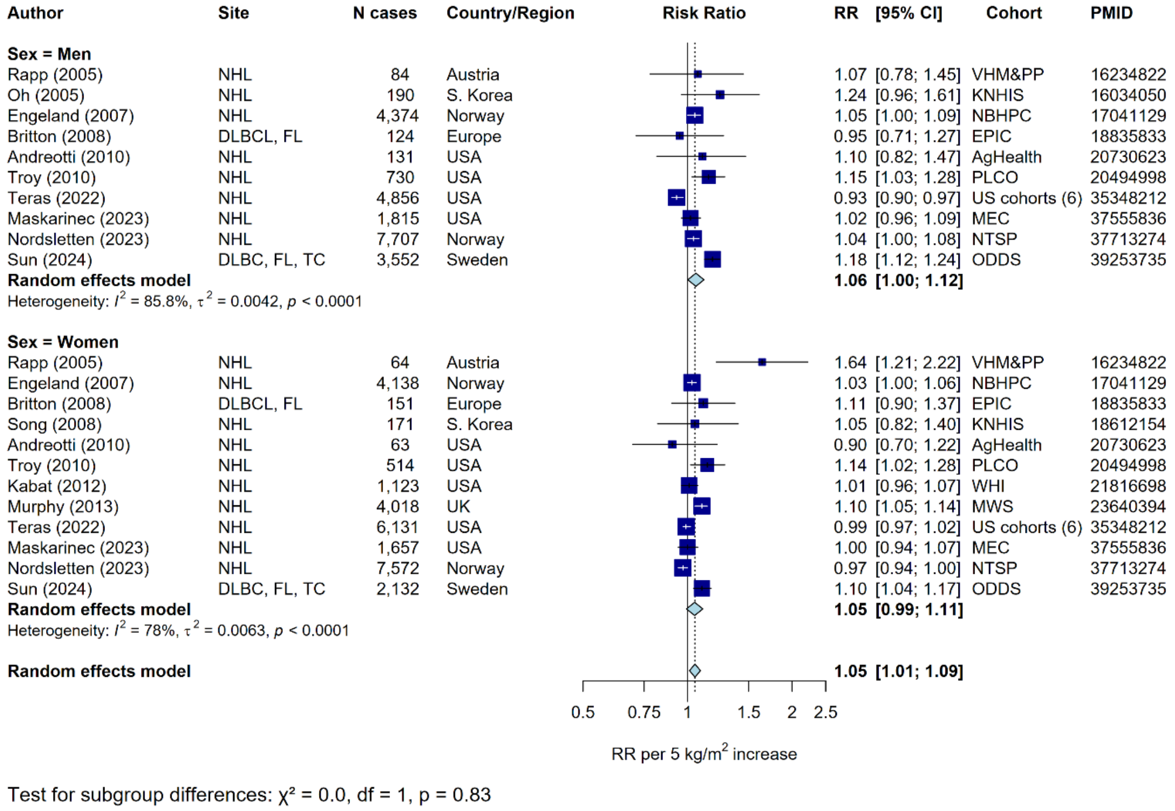

Supplementary Figure 63: Meta-analysis of prospective studies on the risk of NHL in relation to BMI, by sex

Study-specific RRs are represented by squares (with their 95% CIs as lines). RRs were combined using random effects meta-analysis (represented by a diamond). Heterogeneity across studies was assessed using I^2 and tau^2 within sex. Heterogeneity in the pooled estimates by region was assessed using the Q-statistic. Further details of model adjustments, follow-up time, analytic population for each study are available from **Supplementary Data**.

Abbreviations: BMI=body mass index, CI=confidence interval, DLBCL=diffuse large B-cell lymphoma, FL=follicular lymphoma, NHL=non-Hodgkin lymphoma, PMID=PubMed ID, RR=risk ratio, TC=t-cell lymphoma

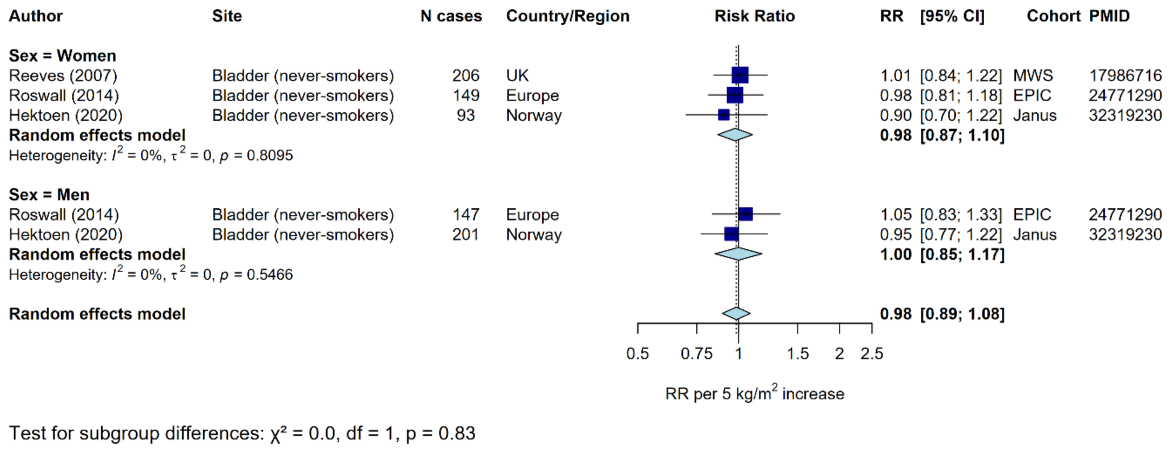

Supplementary Figure 64: Meta-analysis of prospective studies on the risk of bladder cancer (never-smokers) in relation to BMI, by sex

Study-specific RRs are represented by squares (with their 95% CIs as lines). RRs were combined using random effects meta-analysis (represented by a diamond). Heterogeneity across studies was assessed using I^2 and tau^2 within sex. Heterogeneity in the pooled estimates by region was assessed using the Q-statistic. Further details of model adjustments, follow-up time, analytic population for each study are available from **Supplementary Data**.

Abbreviations: BMI=body mass index, CI=confidence interval, PMID=PubMed ID, RR=risk ratio

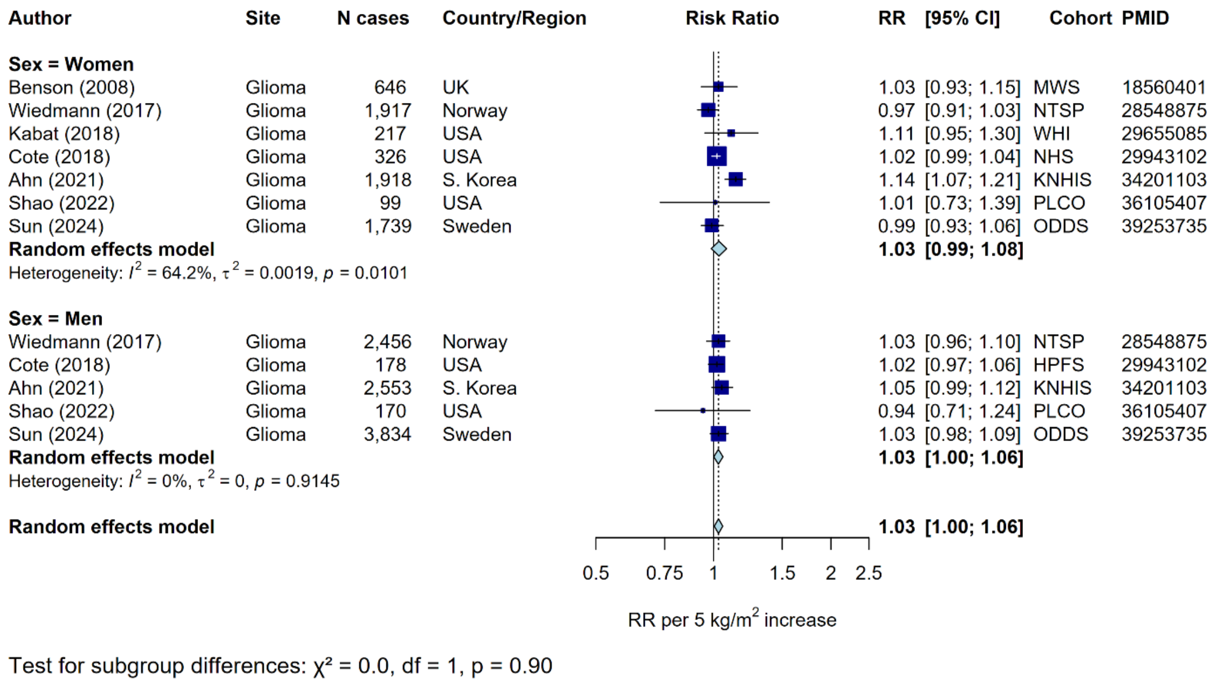

Supplementary Figure 65: Meta-analysis of prospective studies on the risk of glioma in relation to BMI, by sex

Study-specific RRs are represented by squares (with their 95% CIs as lines). RRs were combined using random effects meta-analysis (represented by a diamond). Heterogeneity across studies was assessed using I^2 and tau^2 within sex. Heterogeneity in the pooled estimates by region was assessed using the Q-statistic. Further details of model adjustments, follow-up time, analytic population for each study are available from **Supplementary Data**.

Abbreviations: BMI=body mass index, CI=confidence interval, PMID=PubMed ID, RR=risk ratio

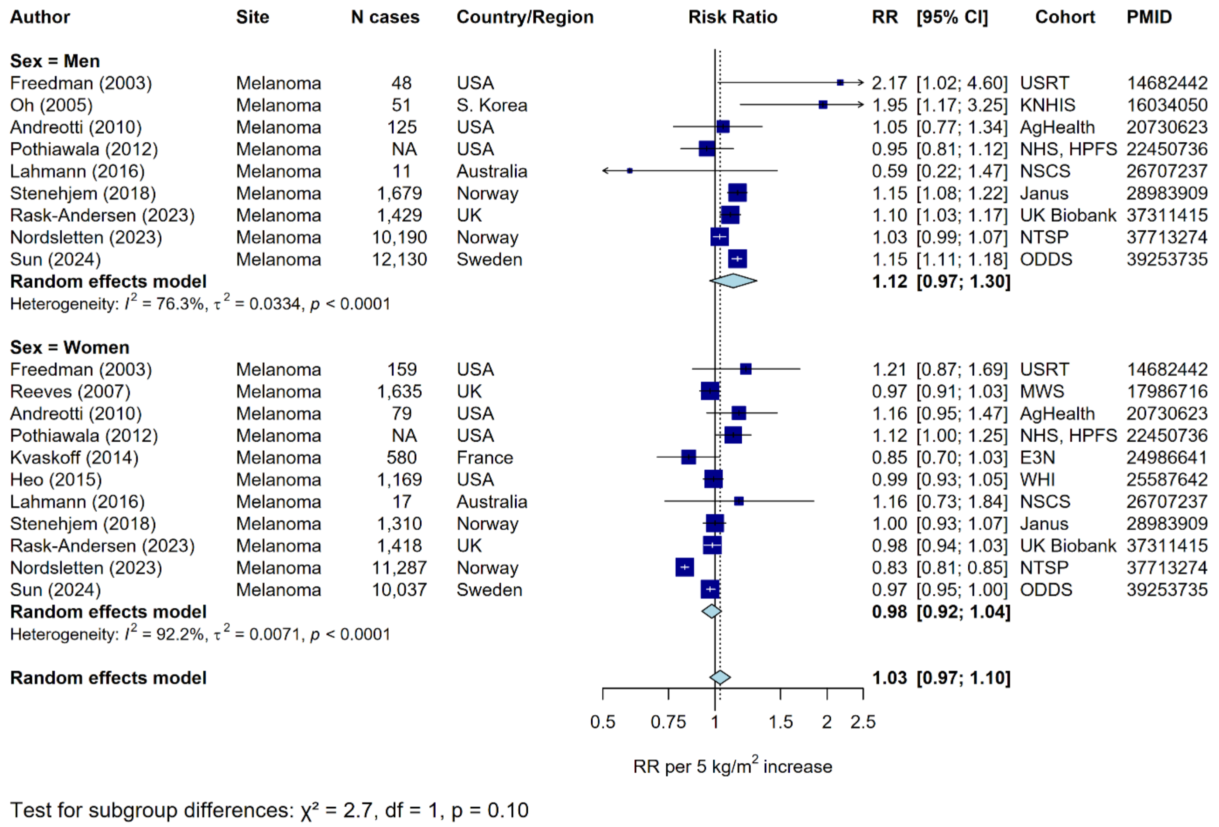

Supplementary Figure 66: Meta-analysis of prospective studies on the risk of melanoma in relation to BMI, by sex

Study-specific RRs are represented by squares (with their 95% CIs as lines). RRs were combined using random effects meta-analysis (represented by a diamond). Heterogeneity across studies was assessed using I^2 and tau^2 within sex. Heterogeneity in the pooled estimates by region was assessed using the Q-statistic. Further details of model adjustments, follow-up time, analytic population for each study are available from **Supplementary Data**.

Abbreviations: BMI=body mass index, CI=confidence interval, PMID=PubMed ID, RR=risk ratio

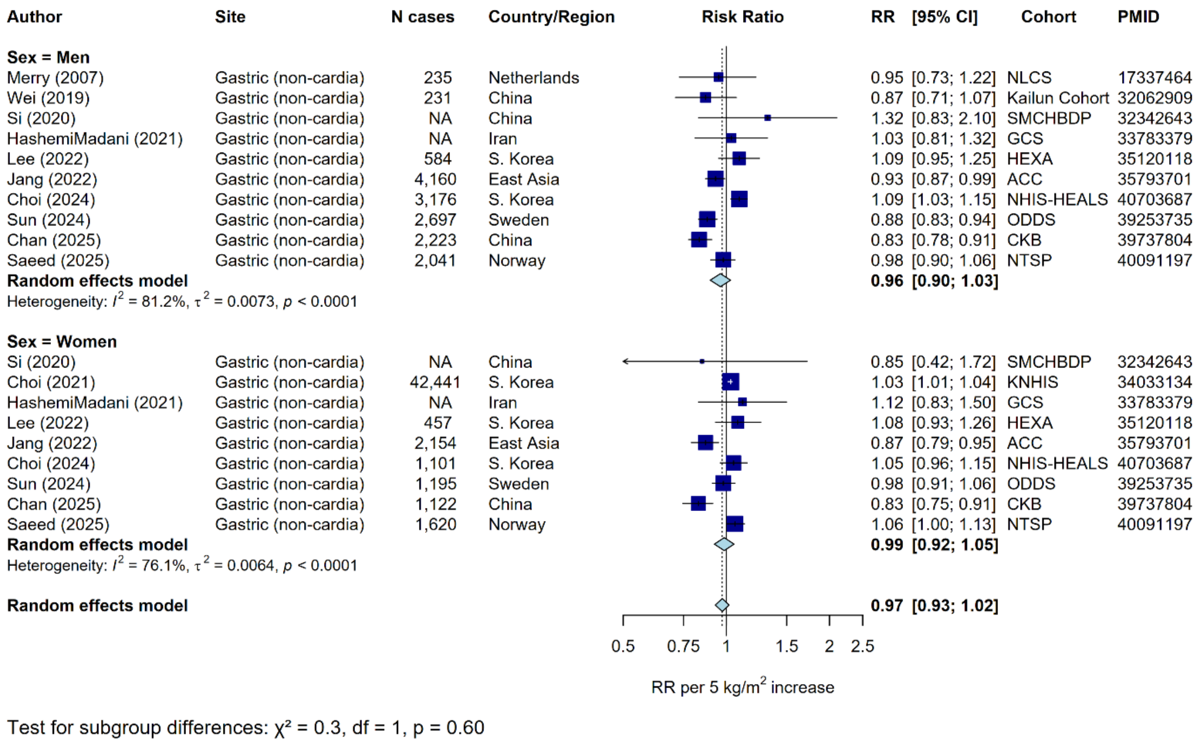

Supplementary Figure 67: Meta-analysis of prospective studies on the risk of gastric (non-cardia) cancer in relation to BMI, by sex

Study-specific RRs are represented by squares (with their 95% CIs as lines). RRs were combined using random effects meta-analysis (represented by a diamond). Heterogeneity across studies was assessed using I^2 and tau^2 within sex. Heterogeneity in the pooled estimates by region was assessed using the Q-statistic. Further details of model adjustments, follow-up time, analytic population for each study are available from **Supplementary Data**.

Abbreviations: BMI=body mass index, CI=confidence interval, PMID=PubMed ID, RR=risk ratio

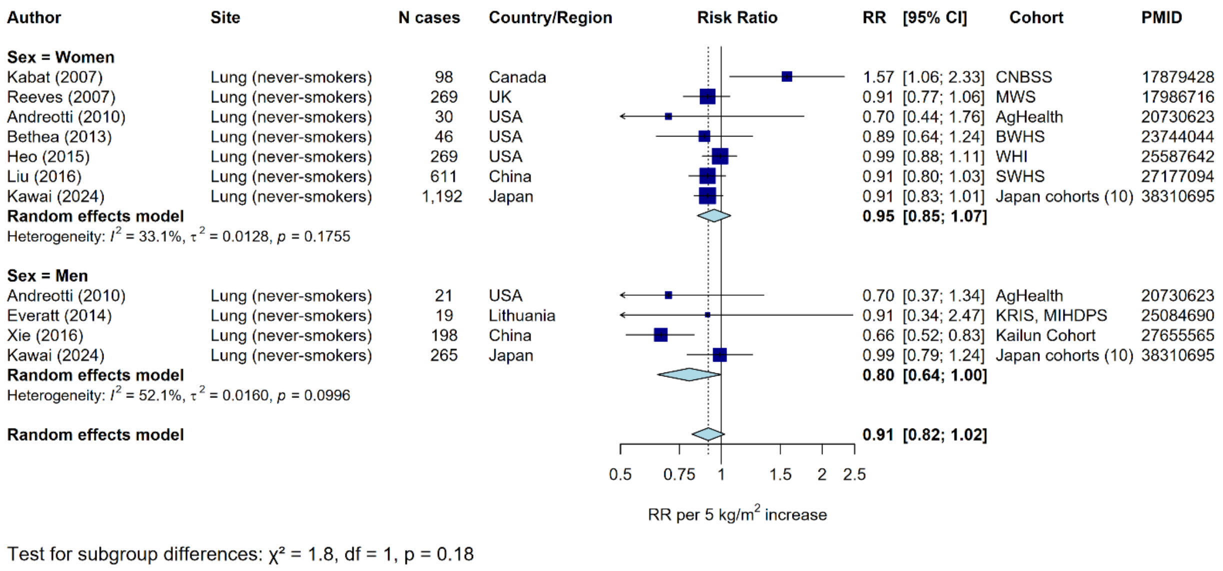

Supplementary Figure 68: Meta-analysis of prospective studies on the risk of lung cancer (never-smokers) in relation to BMI, by sex

Study-specific RRs are represented by squares (with their 95% CIs as lines). RRs were combined using random effects meta-analysis (represented by a diamond). Heterogeneity across studies was assessed using I^2 and tau^2 within sex. Heterogeneity in the pooled estimates by region was assessed using the Q-statistic. Further details of model adjustments, follow-up time, analytic population for each study are available from **Supplementary Data**.

Abbreviations: BMI=body mass index, CI=confidence interval, PMID=PubMed ID, RR=risk ratio

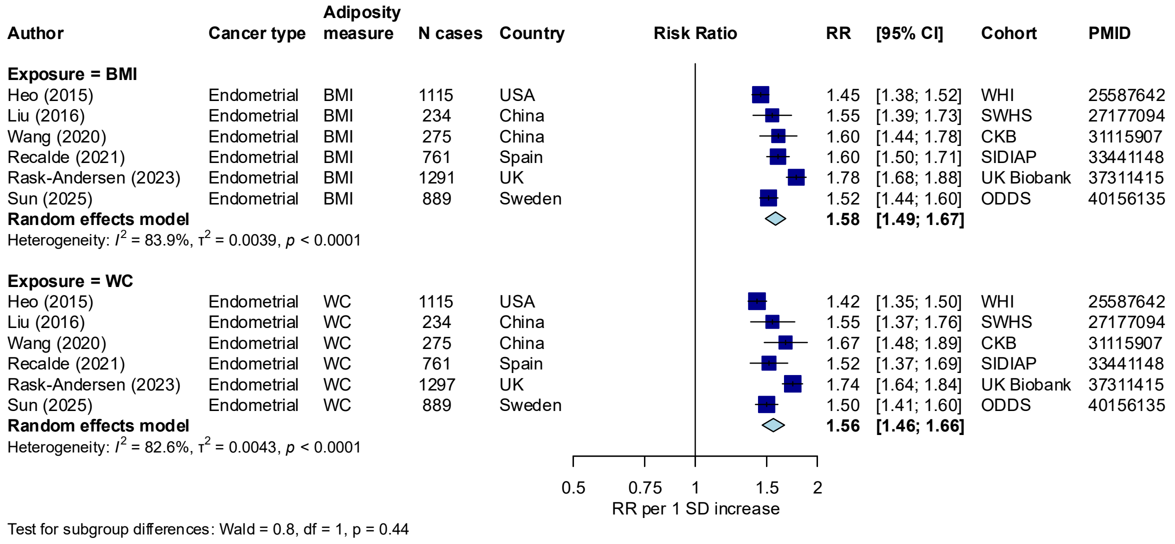

Supplementary Figure 69: Associations of BMI, waist circumference and endometrial cancer risk, per 1 SD increase

Study-specific RRs are represented by squares (with their 95% CIs as lines). RRs were combined using random effects meta-analysis (represented by a diamond). Heterogeneity across studies was assessed using I^2 and tau^2, within adiposity measure. Heterogeneity in the pooled estimates by adiposity measurement was assessed using the Wald statistic.

Abbreviations: BMI=body mass index, CI=confidence interval, PMID=PubMed ID, RR=risk ratio, SD=standard deviation, WC=waist circumference.

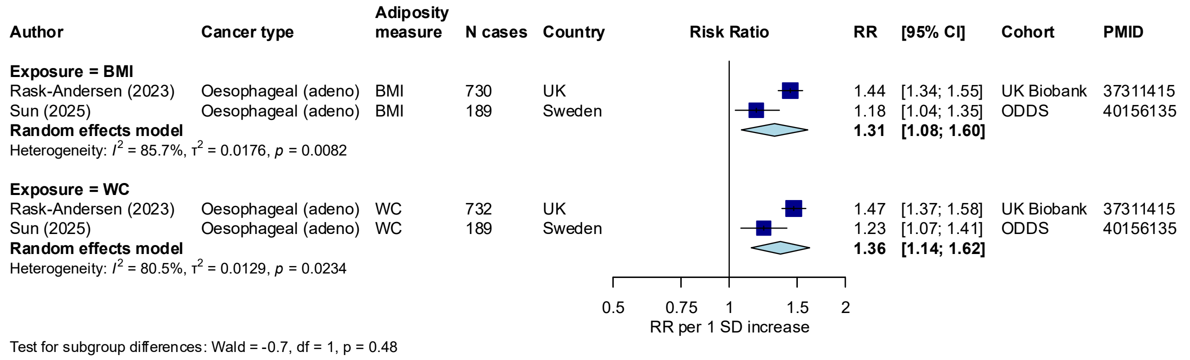

Supplementary Figure 70: Associations of BMI, waist circumference and oesophageal adenocarcinoma risk, per 1 SD increase

Study-specific RRs are represented by squares (with their 95% CIs as lines). RRs were combined using random effects meta-analysis (represented by a diamond). Heterogeneity across studies was assessed using I^2 and tau^2, within adiposity measure. Heterogeneity in the pooled estimates by adiposity measurement was assessed using the Wald statistic.

Abbreviations: BMI=body mass index, CI=confidence interval, PMID=PubMed ID, RR=risk ratio, SD=standard deviation, WC=waist circumference.

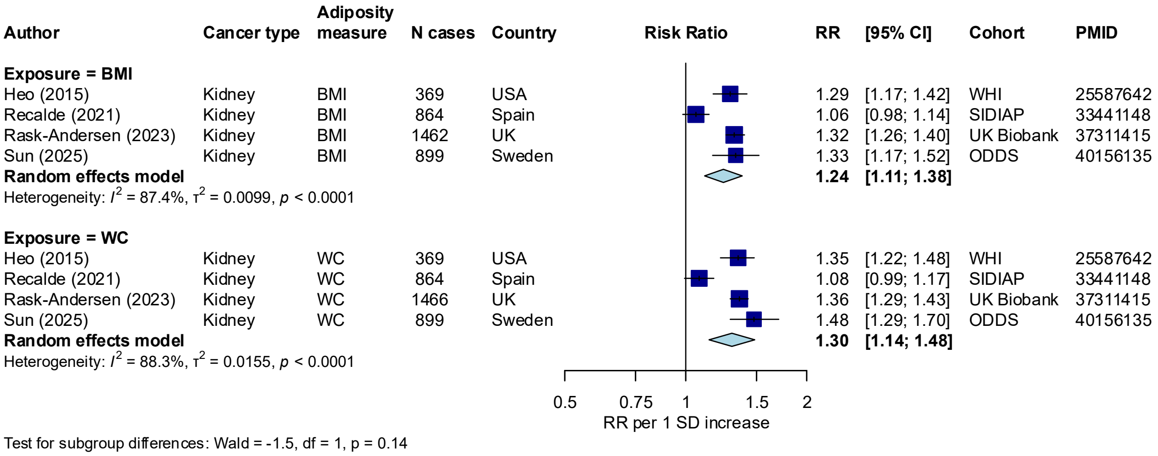

Supplementary Figure 71: Associations of BMI, waist circumference and kidney cancer risk, per 1 SD increase

Study-specific RRs are represented by squares (with their 95% CIs as lines). RRs were combined using random effects meta-analysis (represented by a diamond). Heterogeneity across studies was assessed using I^2 and tau^2, within adiposity measure. Heterogeneity in the pooled estimates by adiposity measurement was assessed using the Wald statistic.

Abbreviations: BMI=Body mass index, CI=confidence interval, PMID=PubMed ID, RR=risk ratio, SD=standard deviation, WC=waist circumference.

Author	Cancer type	Adiposity measure	N cases	Country	RR	[95% CI]	Cohort	PMID
**Exposure = BMI**								
Recalde (2021)	Gallbladder	BMI	208	Spain	1.12	[0.97; 1.30]	SIDIAP	33441148
Rask-Andersen (2023)	Gallbladder	BMI	124	UK	1.45	[1.21; 1.74]	UK Biobank	37311415
Sun (2025)	Gallbladder	BMI	209	Sweden	1.12	[1.02; 1.22]	ODDS	40156135
**Random effects model**					**1.21**	**[1.03; 1.42]**		
Heterogeneity: I^2 = 69.5%, τ^2 = 0.0152, p = 0.0377								
**Exposure = WC**								
Recalde (2021)	Gallbladder	WC	208	Spain	1.12	[0.94; 1.33]	SIDIAP	33441148
Rask-Andersen (2023)	Gallbladder	WC	124	UK	1.51	[1.25; 1.81]	UK Biobank	37311415
Sun (2025)	Gallbladder	WC	209	Sweden	1.11	[1.01; 1.21]	ODDS	40156135
**Random effects model**					**1.22**	**[1.01; 1.48]**		
Heterogeneity: I^2 = 77%, τ^2 = 0.0230, p = 0.0130								

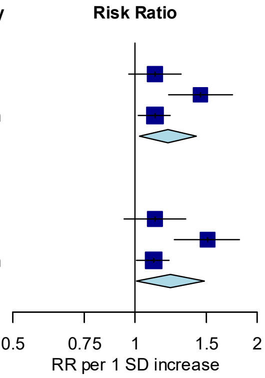

Test for subgroup differences: Wald = -0.3, df = 1, p = 0.77

Supplementary Figure 72: Associations of BMI, waist circumference and gallbladder cancer risk, per 1 SD increase

Study-specific RRs are represented by squares (with their 95% CIs as lines). RRs were combined using random effects meta-analysis (represented by a diamond). Heterogeneity across studies was assessed using I^2 and tau^2, within adiposity measure. Heterogeneity in the pooled estimates by adiposity measurement was assessed using the Wald statistic.

Abbreviations: BMI=Body mass index, CI=confidence interval, PMID=PubMed ID, RR=risk ratio, SD=standard deviation, WC=waist circumference.

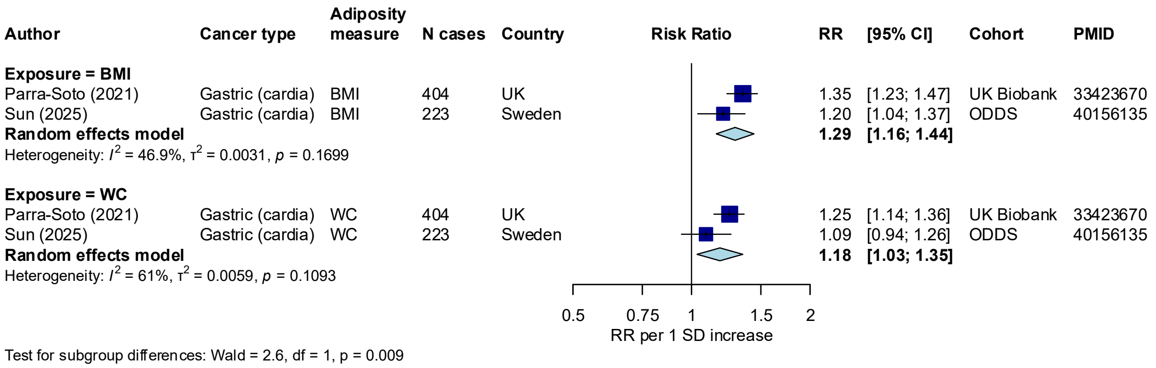

Supplementary Figure 73: Associations of BMI, waist circumference and gastric cancer (cardia) risk, per 1 SD increase

Study-specific RRs are represented by squares (with their 95% CIs as lines). RRs were combined using random effects meta-analysis (represented by a diamond). Heterogeneity across studies was assessed using I^2 and tau^2, within adiposity measure. Heterogeneity in the pooled estimates by adiposity measurement was assessed using the Wald statistic.

Abbreviations: BMI=Body mass index, CI=confidence interval, PMID=PubMed ID, RR=risk ratio, SD=standard deviation, WC=waist circumference.

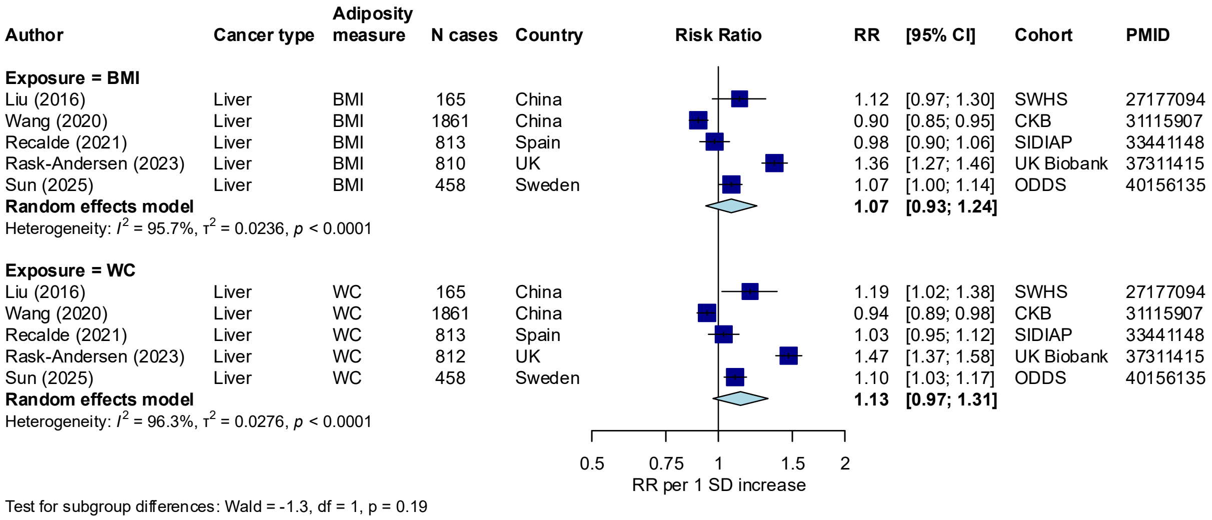

Supplementary Figure 74: Associations of BMI, waist circumference and liver cancer risk, per 1 SD increase

Study-specific RRs are represented by squares (with their 95% CIs as lines). RRs were combined using random effects meta-analysis (represented by a diamond). Heterogeneity across studies was assessed using I^2 and tau^2, within adiposity measure. Heterogeneity in the pooled estimates by adiposity measurement was assessed using the Wald statistic.

Abbreviations: BMI=Body mass index, CI=confidence interval, PMID=PubMed ID, RR=risk ratio, SD=standard deviation, WC=waist circumference.

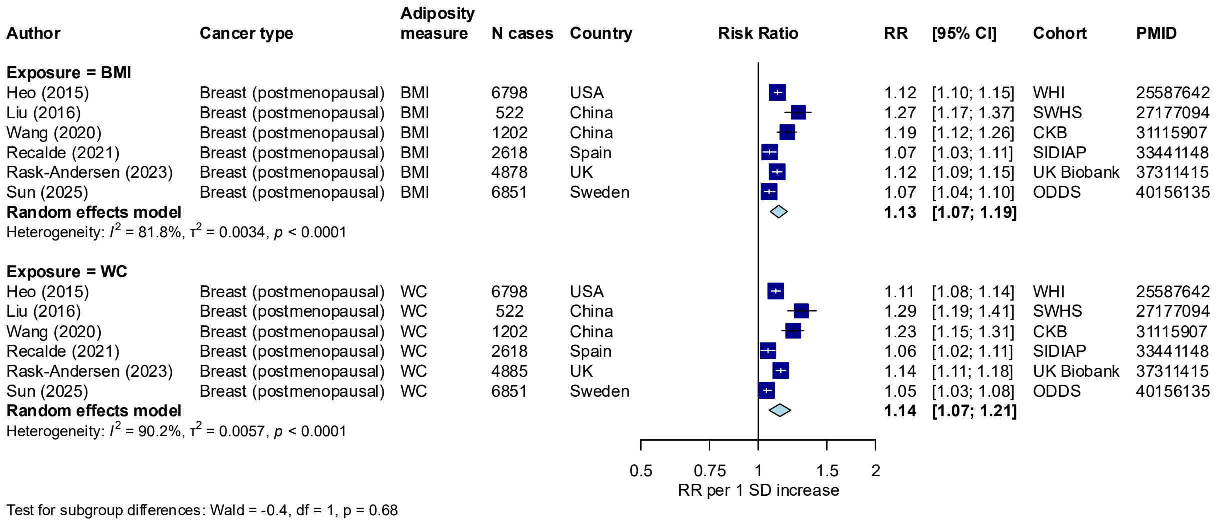

Supplementary Figure 75: Associations of BMI, waist circumference and breast (postmenopausal) cancer risk, per 1 SD increase

Study-specific RRs are represented by squares (with their 95% CIs as lines). RRs were combined using random effects meta-analysis (represented by a diamond). Heterogeneity across studies was assessed using I^2 and tau^2, within adiposity measure. Heterogeneity in the pooled estimates by adiposity measurement was assessed using the Wald statistic.

Abbreviations: BMI=Body mass index, CI=confidence interval, PMID=PubMed ID, RR=risk ratio, SD=standard deviation, WC=waist circumference.

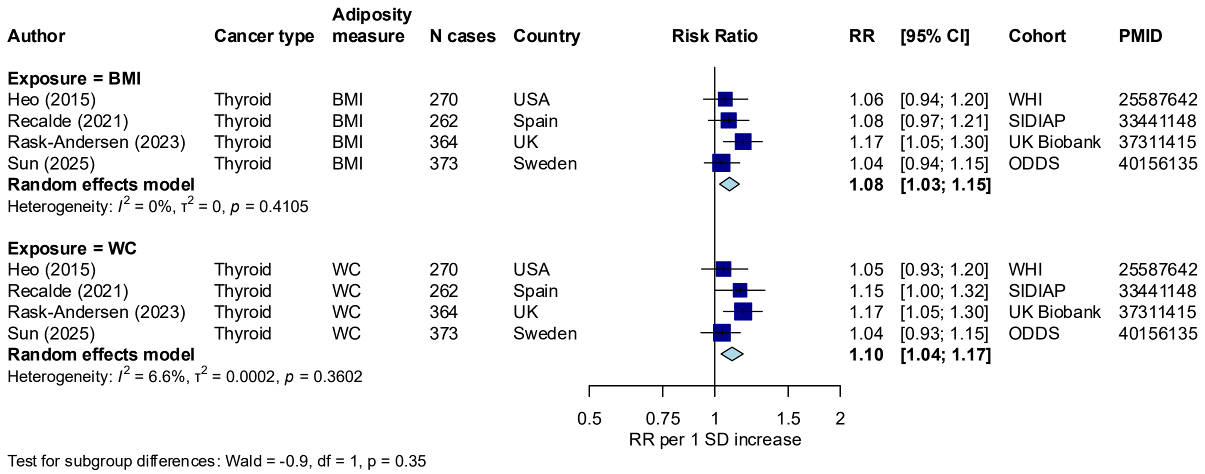

Supplementary Figure 76: Associations of BMI, waist circumference and thyroid cancer risk, per 1 SD increase

Study-specific RRs are represented by squares (with their 95% CIs as lines). RRs were combined using random effects meta-analysis (represented by a diamond). Heterogeneity across studies was assessed using I^2 and tau^2, within adiposity measure. Heterogeneity in the pooled estimates by adiposity measurement was assessed using the Wald statistic.

Abbreviations: BMI=Body mass index, CI=confidence interval, PMID=PubMed ID, RR=risk ratio, SD=standard deviation, WC=waist circumference.

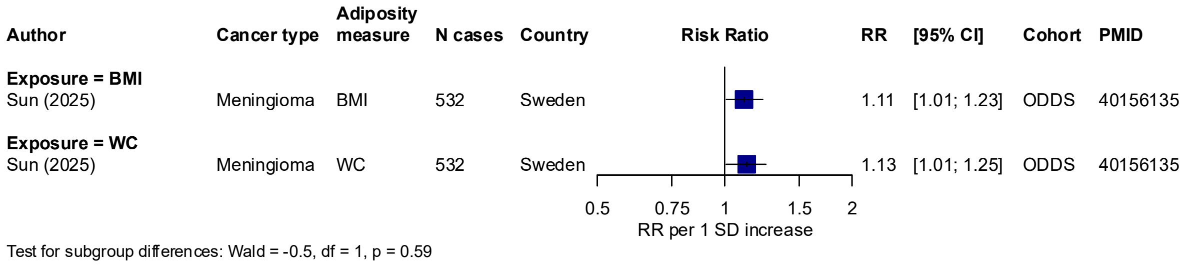

Supplementary Figure 77: Associations of BMI, waist circumference and meningioma risk, per 1 SD increase

Study-specific RRs are represented by squares (with their 95% CIs as lines). RRs were combined using random effects meta-analysis (represented by a diamond). Heterogeneity across studies was assessed using I^2 and tau^2, within adiposity measure. Heterogeneity in the pooled estimates by adiposity measurement was assessed using the Wald statistic.

Abbreviations: BMI=Body mass index, CI=confidence interval, PMID=PubMed ID, RR=risk ratio, SD=standard deviation, WC=waist circumference.

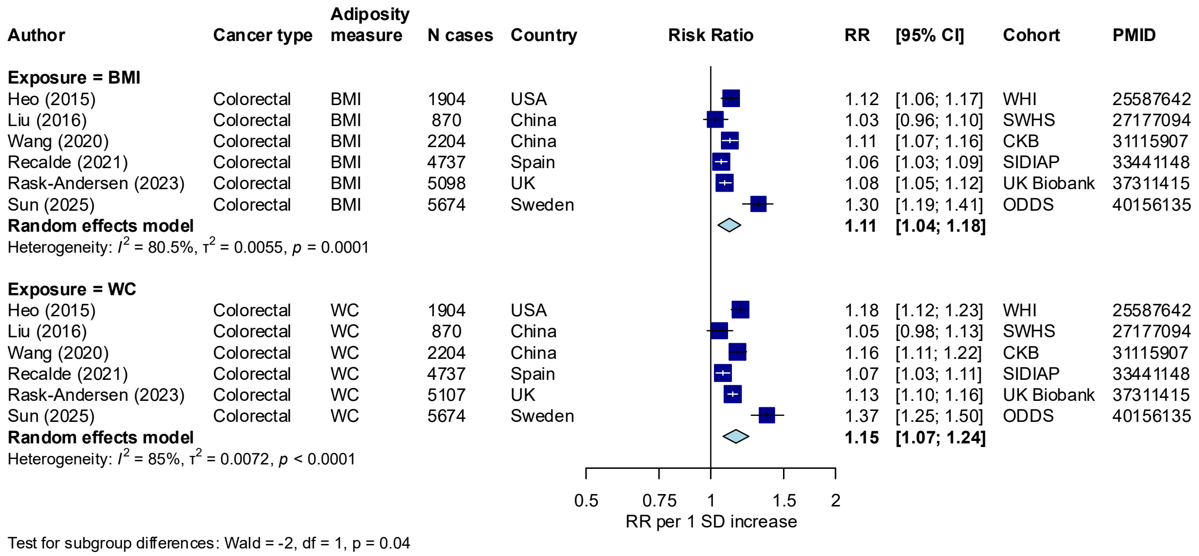

Supplementary Figure 78: Associations of BMI, waist circumference and colorectal cancer risk, per 1 SD increase

Study-specific RRs are represented by squares (with their 95% CIs as lines). RRs were combined using random effects meta-analysis (represented by a diamond). Heterogeneity across studies was assessed using I^2 and tau^2, within adiposity measure. Heterogeneity in the pooled estimates by adiposity measurement was assessed using the Wald statistic.

Abbreviations: BMI=Body mass index, CI=confidence interval, PMID=PubMed ID, RR=risk ratio, SD=standard deviation, WC=waist circumference.

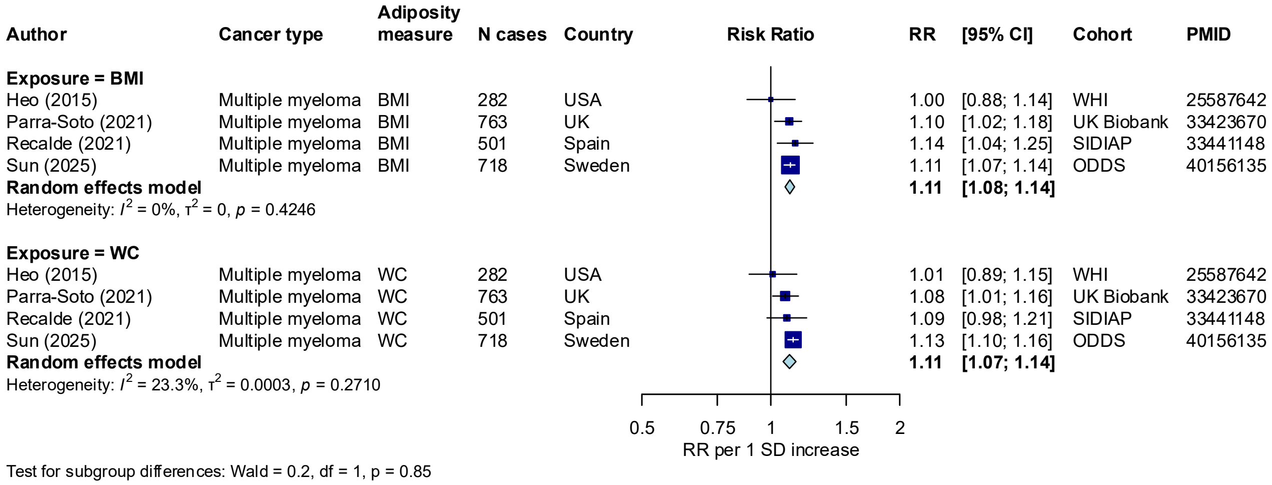

Supplementary Figure 79: Associations of BMI, waist circumference and multiple myeloma risk, per 1 SD increase

Study-specific RRs are represented by squares (with their 95% CIs as lines). RRs were combined using random effects meta-analysis (represented by a diamond). Heterogeneity across studies was assessed using I^2 and tau^2, within adiposity measure. Heterogeneity in the pooled estimates by adiposity measurement was assessed using the Wald statistic.

Abbreviations: BMI=Body mass index, CI=confidence interval, PMID=PubMed ID, RR=risk ratio, SD=standard deviation, WC=waist circumference.

Author	Cancer type	Adiposity measure	N cases	Country	RR	[95% CI]	Cohort	PMID
**Exposure = BMI**								
Heo (2015)	Leukemia	BMI	447	USA	1.07	[0.97; 1.08]	WHI	25587642
Wang (2020)	Leukemia	BMI	443	China	1.04	[0.95; 1.15]	CKB	31115907
Parra-Soto (2021)	Leukemia	BMI	1129	UK	1.07	[1.01; 1.14]	UK Biobank	33423670
Recalde (2021)	Leukemia	BMI	969	Spain	1.04	[0.97; 1.12]	SIDIAP	33441148
**Random effects model**					**1.06**	**[1.03; 1.10]**		
Heterogeneity: $I^2 = 0\%$, $\tau^2 = 0$, $p = 0.8788$								
**Exposure = WC**								
Heo (2015)	Leukemia	WC	447	USA	1.06	[0.96; 1.16]	WHI	25587642
Wang (2020)	Leukemia	WC	443	China	1.03	[0.93; 1.14]	CKB	31115907
Parra-Soto (2021)	Leukemia	WC	1129	UK	1.08	[1.02; 1.15]	UK Biobank	33423670
Recalde (2021)	Leukemia	WC	969	Spain	1.09	[1.01; 1.18]	SIDIAP	33441148
**Random effects model**					**1.07**	**[1.03; 1.11]**		
Heterogeneity: $I^2 = 0\%$, $\tau^2 = 0$, $p = 0.8314$								

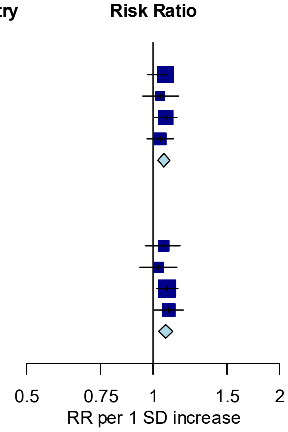

Test for subgroup differences: Wald = -1, df = 1, p = 0.33

Supplementary Figure 80: Associations of BMI, waist circumference and leukaemia risk, per 1 SD increase

Study-specific RRs are represented by squares (with their 95% CIs as lines). RRs were combined using random effects meta-analysis (represented by a diamond). Heterogeneity across studies was assessed using I^2 and tau^2, within adiposity measure. Heterogeneity in the pooled estimates by adiposity measurement was assessed using the Wald statistic.

Abbreviations: BMI=Body mass index, CI=confidence interval, PMID=PubMed ID, RR=risk ratio, SD=standard deviation, WC=waist circumference.

Author	Cancer type	Adiposity measure	N cases	Country	RR	[95% CI]	Cohort	PMID
**Exposure = BMI**								
Wang (2020)	Head and neck (overall)	BMI	422	China	0.91	[0.82; 1.01]	CKB	31115907
Recalde (2021)	Head and neck (overall)	BMI	672	Spain	0.95	[0.87; 1.04]	SIDIAP	33441148
Rask-Andersen (2023)	Head and neck (overall)	BMI	913	UK	0.86	[0.80; 0.92]	UK Biobank	37311415
Sun (2025)	Head and neck (overall)	BMI	421	Sweden	1.13	[1.05; 1.22]	ODDS	40156135
**Random effects model**					**0.96**	**[0.85; 1.08]**		
Heterogeneity: $I^2 = 89.1\%$, $\tau^2 = 0.0123$, $p < 0.0001$								
**Exposure = WC**								
Wang (2020)	Head and neck (overall)	WC	422	China	0.95	[0.85; 1.06]	CKB	31115907
Recalde (2021)	Head and neck (overall)	WC	672	Spain	1.01	[0.93; 1.10]	SIDIAP	33441148
Rask-Andersen (2023)	Head and neck (overall)	WC	913	UK	0.90	[0.85; 0.96]	UK Biobank	37311415
Sun (2025)	Head and neck (overall)	WC	421	Sweden	1.12	[1.03; 1.21]	ODDS	40156135
**Random effects model**					**0.99**	**[0.90; 1.09]**		
Heterogeneity: $I^2 = 84\%$, $\tau^2 = 0.0073$, $p = 0.0003$								

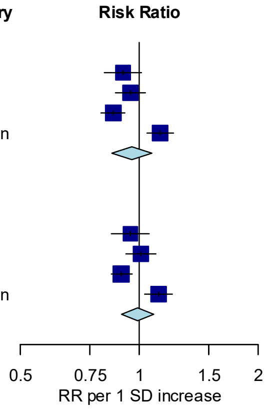

Test for subgroup differences: Wald = -1.1, df = 1, p = 0.26

Supplementary Figure 81: Associations of BMI, waist circumference and head and neck cancer risk, per 1 SD increase

Study-specific RRs are represented by squares (with their 95% CIs as lines). RRs were combined using random effects meta-analysis (represented by a diamond). Heterogeneity across studies was assessed using I^2 and tau^2, within adiposity measure. Heterogeneity in the pooled estimates by adiposity measurement was assessed using the Wald statistic.

Abbreviations: BMI=Body mass index, CI=confidence interval, PMID=PubMed ID, RR=risk ratio, SD=standard deviation, WC=waist circumference.

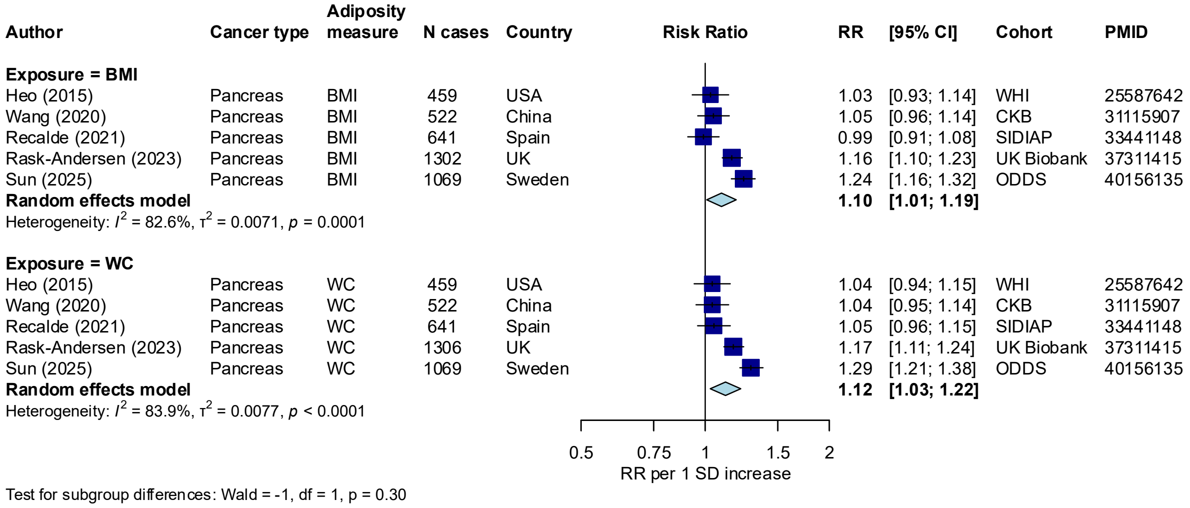

Supplementary Figure 82: Associations of BMI, waist circumference and pancreatic cancer risk, per 1 SD increase

Study-specific RRs are represented by squares (with their 95% CIs as lines). RRs were combined using random effects meta-analysis (represented by a diamond). Heterogeneity across studies was assessed using I^2 and tau^2, within adiposity measure. Heterogeneity in the pooled estimates by adiposity measurement was assessed using the Wald statistic.

Abbreviations: BMI=Body mass index, CI=confidence interval, PMID=PubMed ID, RR=risk ratio, SD=standard deviation, WC=waist circumference.

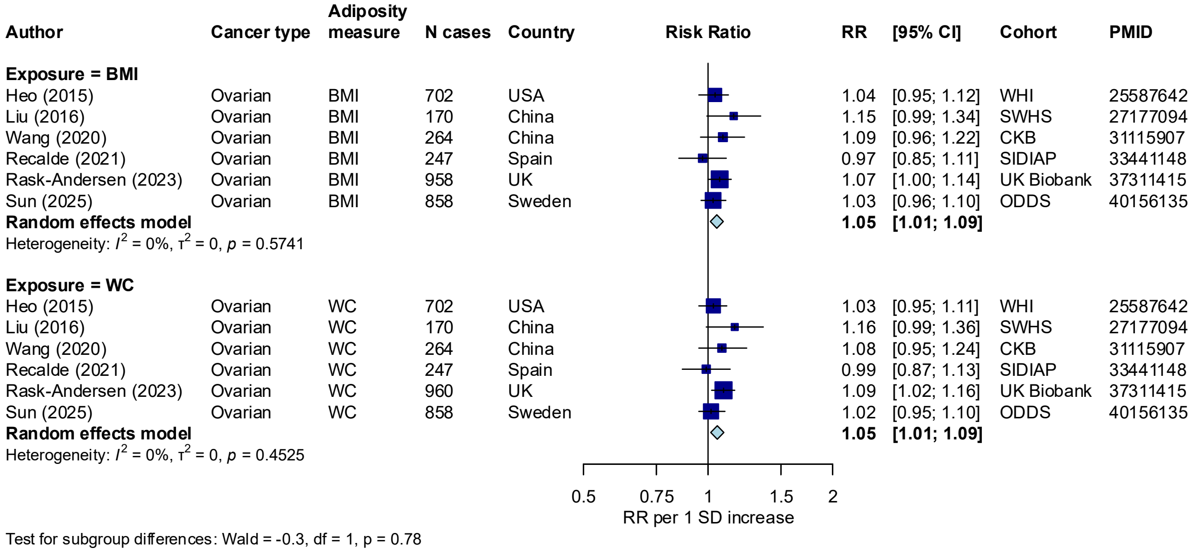

Supplementary Figure 83: Associations of BMI, waist circumference and ovarian cancer risk, per 1 SD increase

Study-specific RRs are represented by squares (with their 95% CIs as lines). RRs were combined using random effects meta-analysis (represented by a diamond). Heterogeneity across studies was assessed using I^2 and tau^2, within adiposity measure. Heterogeneity in the pooled estimates by adiposity measurement was assessed using the Wald statistic.

Abbreviations: BMI=Body mass index, CI=confidence interval, PMID=PubMed ID, RR=risk ratio, SD=standard deviation, WC=waist circumference.

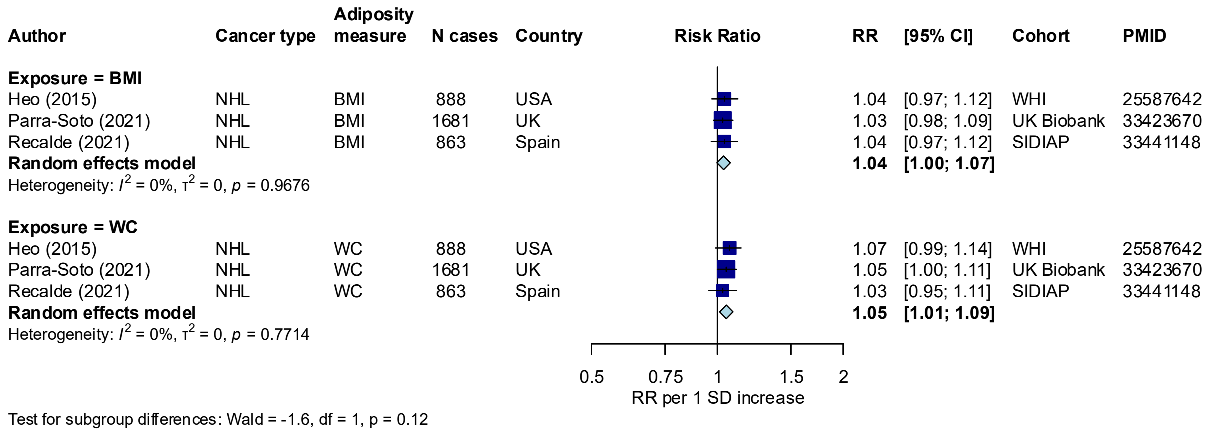

Supplementary Figure 84: Associations of BMI, waist circumference and NHL risk, per 1 SD increase

Study-specific RRs are represented by squares (with their 95% CIs as lines). RRs were combined using random effects meta-analysis (represented by a diamond). Heterogeneity across studies was assessed using I^2 and tau^2, within adiposity measure. Heterogeneity in the pooled estimates by adiposity measurement was assessed using the Wald statistic.

Abbreviations: BMI=Body mass index, CI=confidence interval, NHL=non-Hodgkin lymphoma, PMID=PubMed ID, RR=risk ratio, SD=standard deviation, WC=waist circumference.

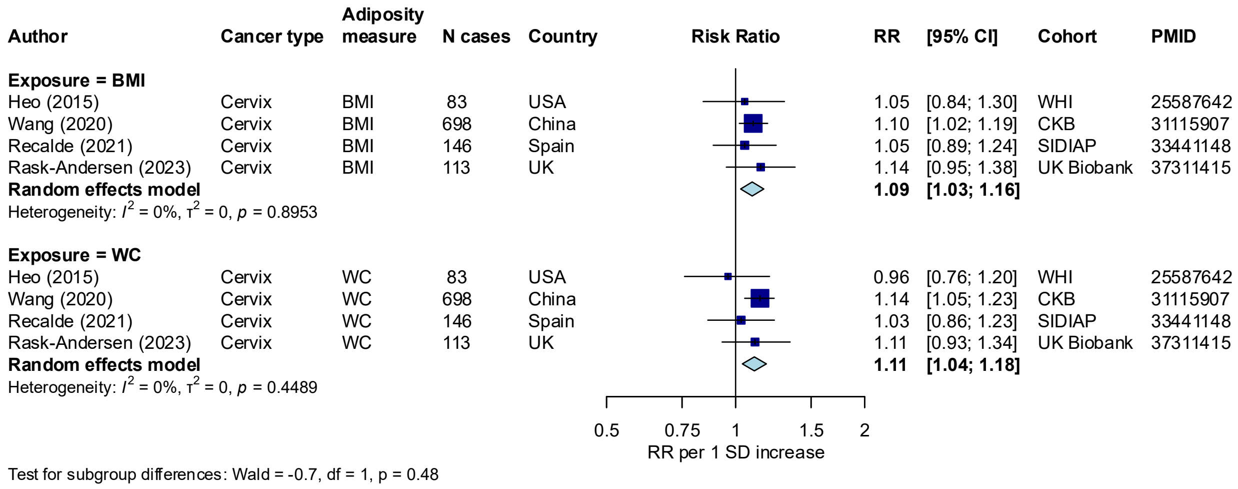

Supplementary Figure 85: Associations of BMI, waist circumference and cervical cancer risk, per 1 SD increase

Study-specific RRs are represented by squares (with their 95% CIs as lines). RRs were combined using random effects meta-analysis (represented by a diamond). Heterogeneity across studies was assessed using I^2 and tau^2, within adiposity measure. Heterogeneity in the pooled estimates by adiposity measurement was assessed using the Wald statistic.

Abbreviations: BMI=Body mass index, CI=confidence interval, PMID=PubMed ID, RR=risk ratio, SD=standard deviation, WC=waist circumference.

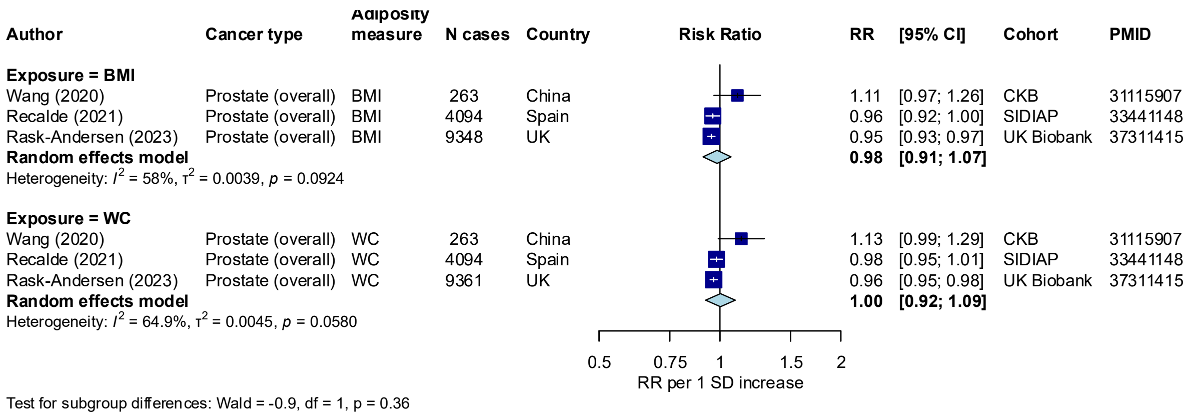

Supplementary Figure 86: Associations of BMI, waist circumference and prostate cancer risk, per 1 SD increase

Study-specific RRs are represented by squares (with their 95% CIs as lines). RRs were combined using random effects meta-analysis (represented by a diamond). Heterogeneity across studies was assessed using I^2 and tau^2, within adiposity measure. Heterogeneity in the pooled estimates by adiposity measurement was assessed using the Wald statistic.

Abbreviations: BMI=Body mass index, CI=confidence interval, PMID=PubMed ID, RR=risk ratio, SD=standard deviation, WC=waist circumference.

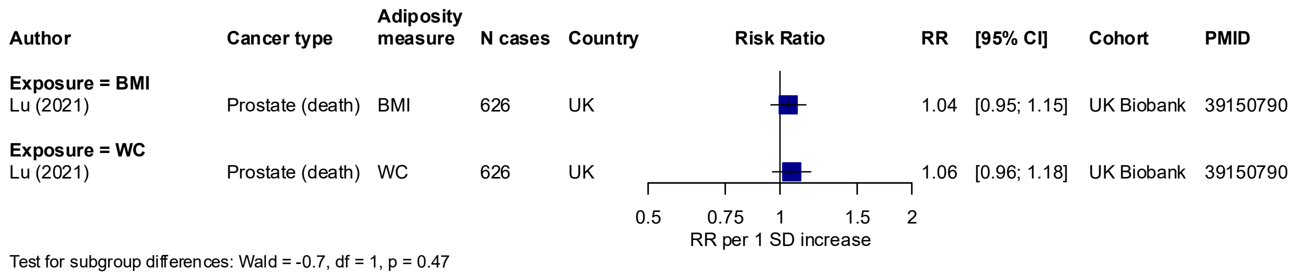

Supplementary Figure 87: Associations of BMI, waist circumference and fatal prostate cancer risk, per 1 SD increase

Study-specific RRs are represented by squares (with their 95% CIs as lines). RRs were combined using random effects meta-analysis (represented by a diamond). Heterogeneity across studies was assessed using I^2 and tau^2, within adiposity measure. Heterogeneity in the pooled estimates by adiposity measurement was assessed using the Wald statistic.

Abbreviations: BMI=Body mass index, CI=confidence interval, PMID=PubMed ID, RR=risk ratio, SD=standard deviation, WC=waist circumference.

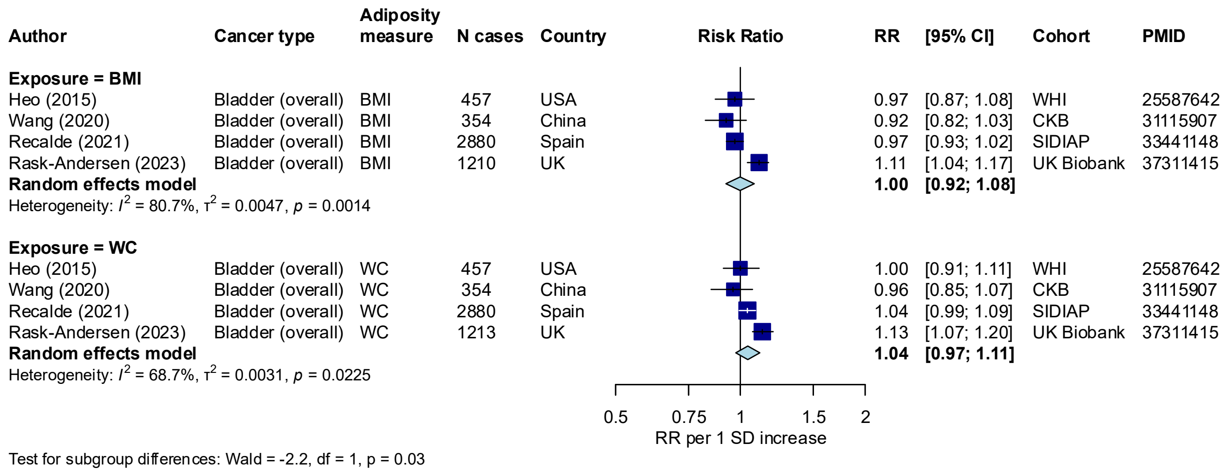

Supplementary Figure 88: Associations of BMI, waist circumference and bladder cancer risk, per 1 SD increase

Study-specific RRs are represented by squares (with their 95% CIs as lines). RRs were combined using random effects meta-analysis (represented by a diamond). Heterogeneity across studies was assessed using I^2 and tau^2, within adiposity measure. Heterogeneity in the pooled estimates by adiposity measurement was assessed using the Wald statistic.

Abbreviations: BMI=Body mass index, CI=confidence interval, PMID=PubMed ID, RR=risk ratio, SD=standard deviation, WC=waist circumference.

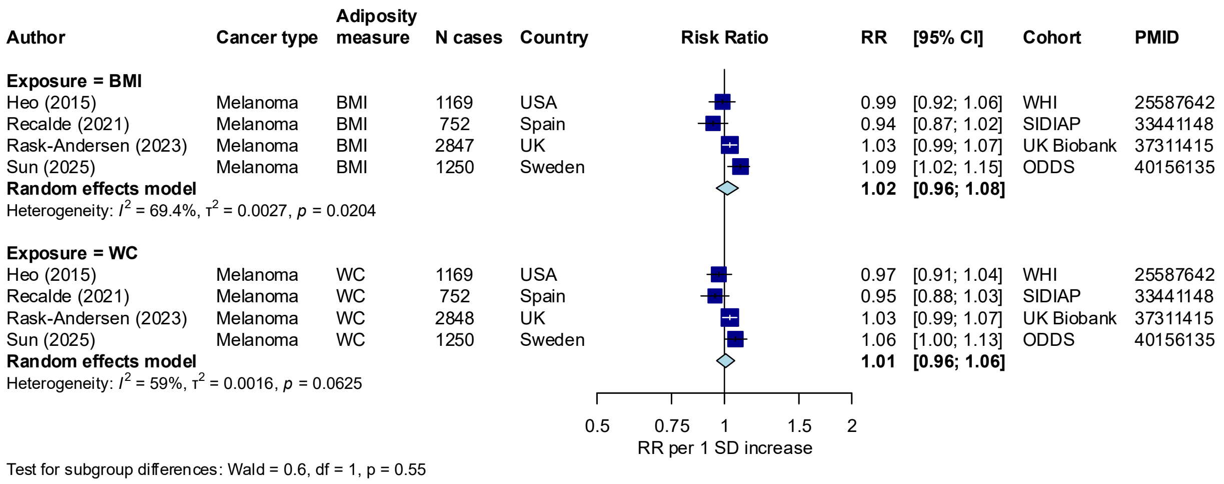

Supplementary Figure 89: Associations of BMI, waist circumference and melanoma risk, per 1 SD increase

Study-specific RRs are represented by squares (with their 95% CIs as lines). RRs were combined using random effects meta-analysis (represented by a diamond). Heterogeneity across studies was assessed using I^2 and tau^2, within adiposity measure. Heterogeneity in the pooled estimates by adiposity measurement was assessed using the Wald statistic.

Abbreviations: BMI=Body mass index, CI=confidence interval, PMID=PubMed ID, RR=risk ratio, SD=standard deviation, WC=waist circumference.

Author	Cancer type	Adiposity measure	N cases	Country	RR	[95% CI]	Cohort	PMID
**Exposure = BMI**								
Wang (2020)	Gastric (non-cardia)	BMI	2215	China	0.92	[0.87; 0.96]	CKB	31115907
Parra-Soto (2021)	Gastric (non-cardia)	BMI	187	UK	1.11	[0.97; 1.28]	UK Biobank	33423670
**Random effects model**					**1.00**	**[0.83; 1.21]**		
Heterogeneity: I^2 = 85.3%, τ^2 = 0.0162, p = 0.0092								
**Exposure = WC**								
Wang (2020)	Gastric (non-cardia)	WC	2215	China	0.92	[0.88; 0.96]	CKB	31115907
Parra-Soto (2021)	Gastric (non-cardia)	WC	187	UK	0.99	[0.86; 1.14]	UK Biobank	33423670
**Random effects model**					**0.93**	**[0.89; 0.97]**		
Heterogeneity: I^2 = 0%, τ^2 = 0, p = 0.3298								

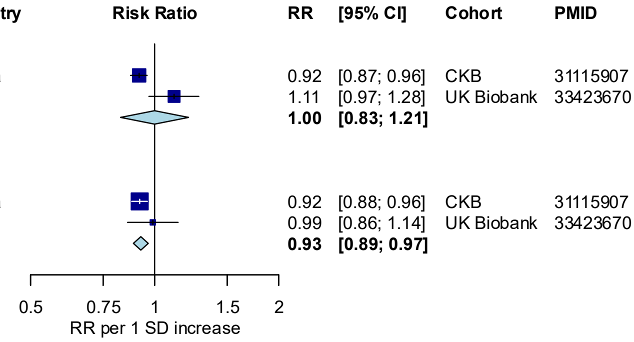

Test for subgroup differences: Wald = 1, df = 1, p = 0.33

Supplementary Figure 90: Associations of BMI, waist circumference and gastric cancer (non-cardia) risk, per 1 SD increase

Study-specific RRs are represented by squares (with their 95% CIs as lines). RRs were combined using random effects meta-analysis (represented by a diamond). Heterogeneity across studies was assessed using I^2 and tau^2, within adiposity measure. Heterogeneity in the pooled estimates by adiposity measurement was assessed using the Wald statistic.

Abbreviations: BMI=Body mass index, CI=confidence interval, PMID=PubMed ID, RR=risk ratio, SD=standard deviation, WC=waist circumference.

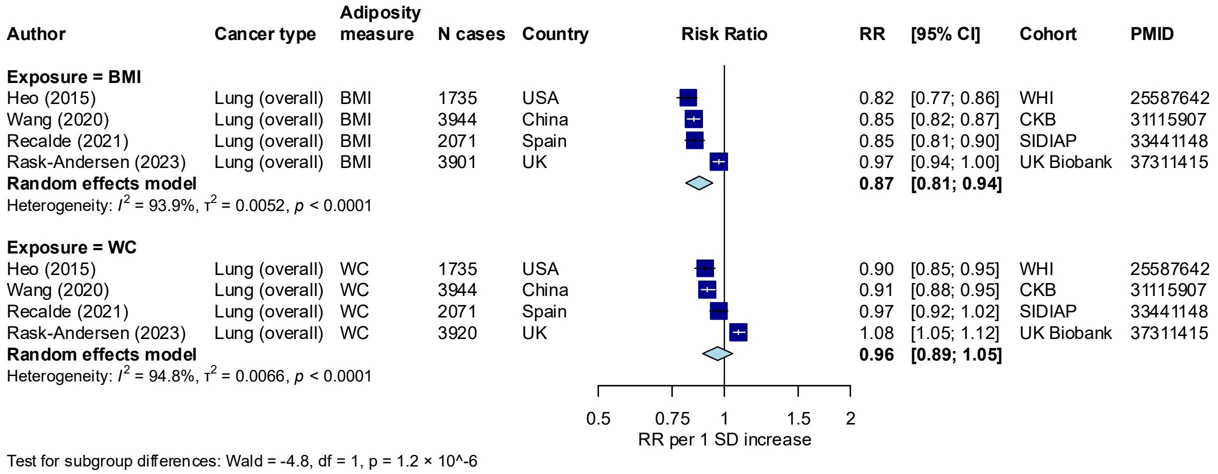

Supplementary Figure 91: Associations of BMI, waist circumference and lung cancer risk, per 1 SD increase

Study-specific RRs are represented by squares (with their 95% CIs as lines). RRs were combined using random effects meta-analysis (represented by a diamond). Heterogeneity across studies was assessed using I^2 and tau^2, within adiposity measure. Heterogeneity in the pooled estimates by adiposity measurement was assessed using the Wald statistic.

Abbreviations: BMI=Body mass index, CI=confidence interval, PMID=PubMed ID, RR=risk ratio, SD=standard deviation, WC=waist circumference.

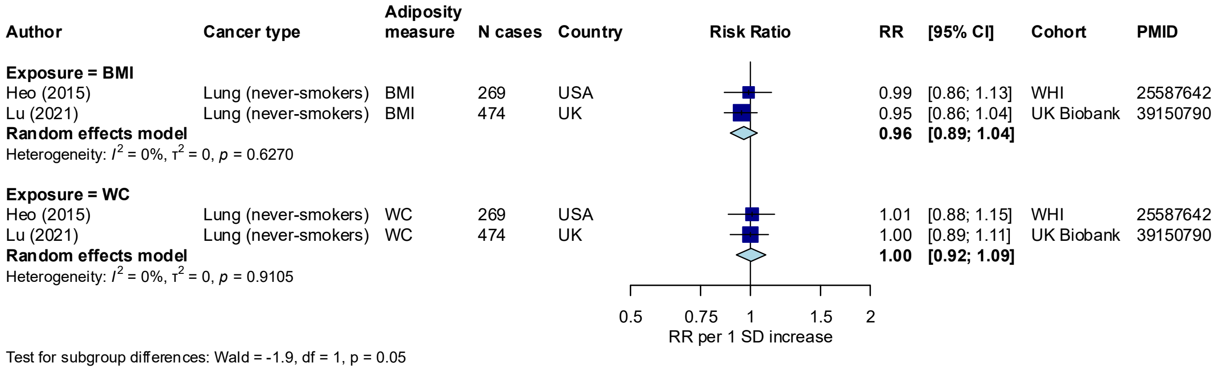

Supplementary Figure 92: Associations of BMI, waist circumference and lung cancer risk (restricted to never-smokers), per 1 SD increase

Study-specific RRs are represented by squares (with their 95% CIs as lines). RRs were combined using random effects meta-analysis (represented by a diamond). Heterogeneity across studies was assessed using I^2 and tau^2, within adiposity measure. Heterogeneity in the pooled estimates by adiposity measurement was assessed using the Wald statistic.

Abbreviations: BMI=Body mass index, CI=confidence interval, PMID=PubMed ID, RR=risk ratio, SD=standard deviation, WC=waist circumference.

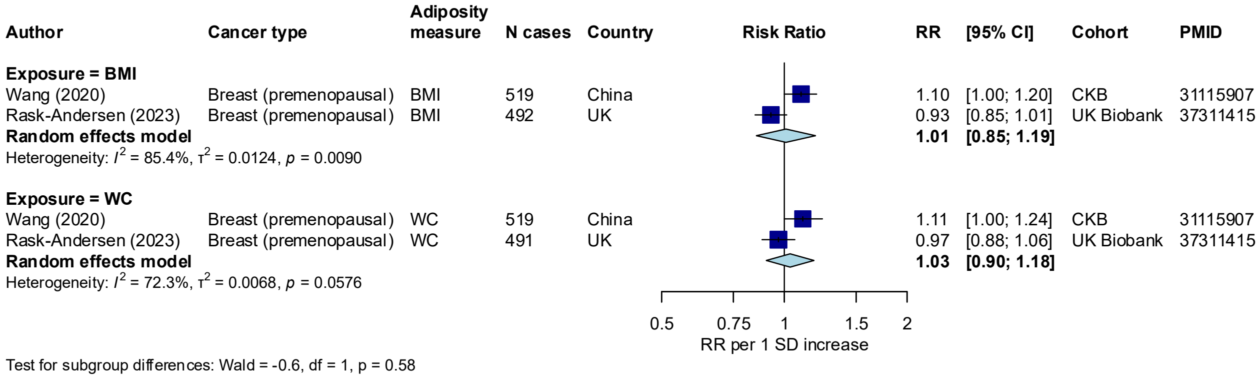

Supplementary Figure 93: Associations of BMI, waist circumference and breast (premenopausal) cancer risk, per 1 SD increase

Study-specific RRs are represented by squares (with their 95% CIs as lines). RRs were combined using random effects meta-analysis (represented by a diamond). Heterogeneity across studies was assessed using I^2 and tau^2, within adiposity measure. Heterogeneity in the pooled estimates by adiposity measurement was assessed using the Wald statistic.

Abbreviations: BMI=Body mass index, CI=confidence interval, PMID=PubMed ID, RR=risk ratio, SD=standard deviation, WC=waist circumference.

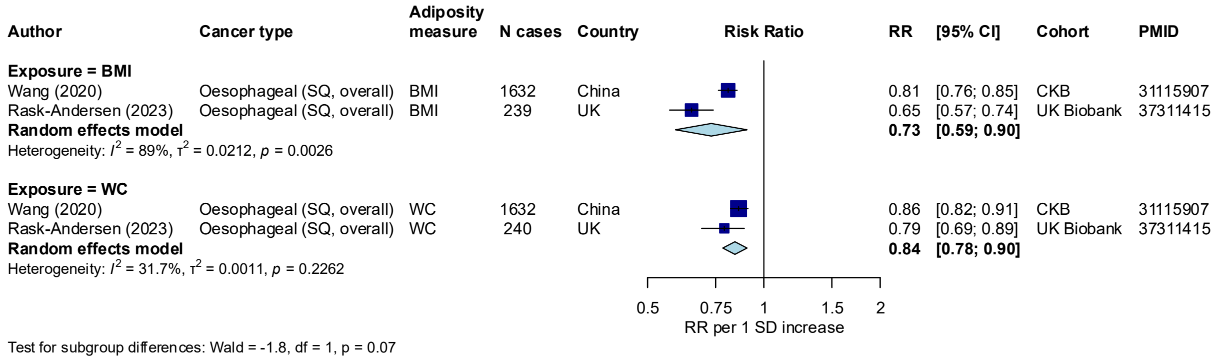

Supplementary Figure 94: Associations of BMI, waist circumference and oesophageal SQ cancer risk, per 1 SD increase

Study-specific RRs are represented by squares (with their 95% CIs as lines). RRs were combined using random effects meta-analysis (represented by a diamond). Heterogeneity across studies was assessed using I^2 and tau^2, within adiposity measure. Heterogeneity in the pooled estimates by adiposity measurement was assessed using the Wald statistic.

Abbreviations: BMI=Body mass index, CI=confidence interval, PMID=PubMed ID, RR=risk ratio, SD=standard deviation, SQ=squamous cell carcinoma, WC=waist circumference.

11. References

1. Freedman, N. D. *et al.* Impact of changing US cigarette smoking patterns on incident cancer: risks of 20 smoking-related cancers among the women and men of the NIH-AARP cohort. *Int. J. Epidemiol.* **45**, 846–856 (2016).
2. Tangen, C. M. *et al.* Biases in Recommendations for and Acceptance of Prostate Biopsy Significantly Affect Assessment of Prostate Cancer Risk Factors: Results From Two Large Randomized Clinical Trials. *J. Clin. Oncol.* **34**, 4338–4344 (2016).
3. WCRF/AICR. Diet, nutrition, physical activity and prostate cancer. (2018).
4. Larsson, S. C., Spyrou, N. & Mantzoros, C. S. Body fatness associations with cancer: evidence from recent epidemiological studies and future directions. *Metabolism* **137**, 155326 (2022).
5. Page, M. J. *et al.* The PRISMA 2020 statement: an updated guideline for reporting systematic reviews. *BMJ* **372**, n71 (2021).
6. Watts, E. L., Moore, S. C., Gunter, M. J. & Chatterjee, N. Adiposity and cancer: meta-analysis, mechanisms, and future perspectives. *medRxiv* 2024.02.16.24302944 (2024) doi:10.1101/2024.02.16.24302944.
7. Arnold, M., Ferlay, J., Henegouwen, M. I. van B. & Soerjomataram, I. Global burden of oesophageal and gastric cancer by histology and subsite in 2018. *Gut* **69**, 1564–1571 (2020).
8. Genkinger, J. M. *et al.* A pooled analysis of 14 cohort studies of anthropometric factors and pancreatic cancer risk. *Int. J. Cancer* **129**, 1708–1717 (2011).
9. Genkinger, J. M. *et al.* Measures of body fatness and height in early and mid-to-late adulthood and prostate cancer: risk and mortality in The Pooling Project of Prospective Studies of Diet and Cancer. *Ann. Oncol. Off. J. Eur. Soc. Med. Oncol.* **31**, 103–114 (2020).
10. van den Brandt, P. A. *et al.* Body size and weight change over adulthood and risk of breast cancer by menopausal and hormone receptor status: a pooled analysis of 20 prospective cohort studies. *Eur. J. Epidemiol.* **36**, 37–55 (2021).
11. Alaggio, R. *et al.* The 5th edition of the World Health Organization Classification of Haematolymphoid Tumours: Lymphoid Neoplasms. *Leukemia* **36**, 1720–1748 (2022).
12. Sun, M. *et al.* Comparing waist circumference with body mass index on obesity-related cancer risk: a pooled Swedish study. *J. Natl. Cancer Inst.* djaf075 (2025) doi:10.1093/jnci/djaf075.
13. Chiolero, A., Faeh, D., Paccaud, F. & Cornuz, J. Consequences of smoking for body weight, body fat distribution, and insulin resistance. *Am. J. Clin. Nutr.* **87**, 801–809 (2008).
14. Greenland, S. & Longnecker, M. P. Methods for trend estimation from summarized dose-response data, with applications to meta-analysis. *Am. J. Epidemiol.* **135**, 1301–1309 (1992).
15. Crippa, A. & Orsini, N. Multivariate Dose-Response Meta-Analysis: The **dosresmeta** *R* Package. *J. Stat. Softw.* **72**, (2016).
16. Higgins, J. P. T. Commentary: Heterogeneity in meta-analysis should be expected and appropriately quantified. *Int. J. Epidemiol.* **37**, 1158–1160 (2008).

17. Paule, R. C. & Mandel, J. Consensus Values and Weighting Factors. *J. Res. Natl. Bur. Stand. 1977* **87**, 377–385 (1982).
18. Christakoudi, S., Tsilidis, K. K., Evangelou, E. & Riboli, E. Association of body-shape phenotypes with imaging measures of body composition in the UK Biobank cohort: relevance to colon cancer risk. *BMC Cancer* **21**, 1106 (2021).
19. Berrington de Gonzalez, A. *et al.* Epidemiological Studies of Low-Dose Ionizing Radiation and Cancer: Rationale and Framework for the Monograph and Overview of Eligible Studies. *J. Natl. Cancer Inst. Monogr.* **2020**, 97–113 (2020).
20. Ding, P. & VanderWeele, T. J. Sensitivity Analysis Without Assumptions. *Epidemiol. Camb. Mass* **27**, 368–377 (2016).
21. Schottenfeld and Fraumeni. *Cancer Epidemiology and Prevention*. (Oxford University Press).
22. Khan, M. *et al.* Sex differences in cancer incidence: prospective analyses in the UK Biobank. *Br. J. Cancer* (2025) doi:10.1038/s41416-025-03028-y.
23. Risk Factors: Age - NCI. https://www.cancer.gov/about-cancer/causes-prevention/risk/age (2015).
24. Duval, S. & Tweedie, R. Trim and fill: A simple funnel-plot-based method of testing and adjusting for publication bias in meta-analysis. *Biometrics* **56**, 455–463 (2000).
25. Bhaskaran, K. *et al.* Body-mass index and risk of 22 specific cancers: a population-based cohort study of 5·24 million UK adults. *The Lancet* **384**, 755–765 (2014).
26. Arhi, C. S. *et al.* Comparison of cancer diagnosis recording between the Clinical Practice Research Datalink, Cancer Registry and Hospital Episodes Statistics. *Cancer Epidemiol.* **57**, 148–157 (2018).
27. Yang, M. S. *et al.* Validation of Cancer Diagnosis Based on the National Health Insurance Service Database versus the National Cancer Registry Database in Korea. *Cancer Res. Treat. Off. J. Korean Cancer Assoc.* **54**, 352–361 (2022).
28. Kim, D. B. *et al.* Waist circumference, body mass index, and colorectal cancer risk according to diabetes status: A Korean nationwide population-based cohort study. *J. Gastroenterol. Hepatol.* **36**, 397–405 (2021).
29. Sanderson, E. *et al.* Mendelian randomization. *Nat. Rev. Methods Primer* **2**, 6 (2022).
30. D, A. *et al.* Grading quality of evidence and strength of recommendations. *BMJ* **328**, (2004).
31. Yang, T. O. *et al.* Body size in early life and the risk of postmenopausal breast cancer. *BMC Cancer* **22**, 232 (2022).
32. Schwarzer, G., Carpenter, J. R. & Rücker, G. *Meta-Analysis with R*. (Springer International Publishing, Cham, 2015). doi:10.1007/978-3-319-21416-0.
33. Pacyga, D. C. *et al.* Associations of Pregnancy History with BMI and Weight Gain in 45-54-Year-Old Women. *Curr. Dev. Nutr.* **4**, nzz139 (2020).
34. Williams, H. & Crawford, D. H. Epstein-Barr virus: the impact of scientific advances on clinical practice. *Blood* **107**, 862–869 (2006).

35. Wee, C. C., Huang, A., Huskey, K. W. & McCarthy, E. P. Obesity and the likelihood of sexual behavioral risk factors for HPV and cervical cancer. *Obes. Silver Spring Md* **16**, 2552–2555 (2008).
36. Chen, D., Wang, S., Yang, W., Lu, H. & Ren, Q. Obesity, abdominal obesity, metabolic obesity phenotypes, and Helicobacter pylori infection: results from NHANES 1999-2000. *BMC Infect. Dis.* **24**, 676 (2024).
37. Xu, M.-Y., Liu, L., Yuan, B.-S., Yin, J. & Lu, Q.-B. Association of obesity with Helicobacter pylori infection: A retrospective study. *World J. Gastroenterol.* **23**, 2750–2756 (2017).
38. Turner, M. C. *et al.* Radon and nonrespiratory mortality in the American Cancer Society cohort. *Am. J. Epidemiol.* **176**, 808–814 (2012).
