# Supplementary material for: Adiposity and cancer: systematic review and meta-analysis
Source: Nat Metab. 2026 Jun 15;8(6):1426–39. doi: 10.1038/s42255-026-01542-8 (PMC13303081; doi:10.1038/s42255-026-01542-8)
Supplement: Supplementary file 2 — Reporting Summary [file 42255_2026_1542_MOESM2_ESM.pdf]

nature portfolio

Corresponding author(s): Eleanor L Watts, Nilanjan Chatterjee, Marc Gunter, Steven Moore

Last updated by author(s): Mar 30, 2026

Reporting Summary

Nature Portfolio wishes to improve the reproducibility of the work that we publish. This form provides structure for consistency and transparency in reporting. For further information on Nature Portfolio policies, see our Editorial Policies and the Editorial Policy Checklist.

Statistics

For all statistical analyses, confirm that the following items are present in the figure legend, table legend, main text, or Methods section.

n/a	Confirmed	
☐	☒	The exact sample size (*n*) for each experimental group/condition, given as a discrete number and unit of measurement
☒	☐	A statement on whether measurements were taken from distinct samples or whether the same sample was measured repeatedly
☐	☒	The statistical test(s) used AND whether they are one- or two-sided<br>*Only common tests should be described solely by name; describe more complex techniques in the Methods section.*
☐	☒	A description of all covariates tested
☒	☐	A description of any assumptions or corrections, such as tests of normality and adjustment for multiple comparisons
☐	☒	A full description of the statistical parameters including central tendency (e.g. means) or other basic estimates (e.g. regression coefficient) AND variation (e.g. standard deviation) or associated estimates of uncertainty (e.g. confidence intervals)
☐	☒	For null hypothesis testing, the test statistic (e.g. *F*, *t*, *r*) with confidence intervals, effect sizes, degrees of freedom and *P* value noted<br>*Give P values as exact values whenever suitable.*
☒	☐	For Bayesian analysis, information on the choice of priors and Markov chain Monte Carlo settings
☒	☐	For hierarchical and complex designs, identification of the appropriate level for tests and full reporting of outcomes
☐	☒	Estimates of effect sizes (e.g. Cohen's *d*, Pearson's *r*), indicating how they were calculated

Our web collection on statistics for biologists contains articles on many of the points above.

Software and code

Policy information about availability of computer code

Data collection	The search strategy was conducted using PubMed, EMBASE and Scopus with search terms available in the Supplementary Table 5. We utilised Covidence and Microsoft Excel for the screening process. More details are available in the Methods and Supplementary Methods.
Data analysis	Statistical analyses were conducted in R (open source) and scripts are publicly available from: https://github.com/elliewatts90/BMI_cancer_meta_analysis

For manuscripts utilizing custom algorithms or software that are central to the research but not yet described in published literature, software must be made available to editors and reviewers. We strongly encourage code deposition in a community repository (e.g. GitHub). See the Nature Portfolio guidelines for submitting code & software for further information.

Data

Policy information about availability of data

All manuscripts must include a data availability statement. This statement should provide the following information, where applicable:

- Accession codes, unique identifiers, or web links for publicly available datasets
- A description of any restrictions on data availability
- For clinical datasets or third party data, please ensure that the statement adheres to our policy

All data synthesised in this meta-analysis are provided in the Supplementary Data files

Research involving human participants, their data, or biological material

Policy information about studies with human participants or human data. See also policy information about sex, gender (identity/presentation), and sexual orientation and race, ethnicity and racism.

Reporting on sex and gender	We use sex throughout to refer to biological sex. This is a secondary analysis so sex was defined by individual studies and was based on self-report or medical records. We carried out a sex-stratified analysis (Page 7, Figure 6).
Reporting on race, ethnicity, or other socially relevant groupings	Our paper is a secondary analysis of published studies therefore we were not able to account for race or ethnicity or how each study reported their groupings, though we have taken care to use the correct terminology for our reporting. Where there are established differences in cancer risk between racial groups (ie. oesphageal, multiple myeloma, melanoma or liver cancer), we penalized studies with <80% racial homogeneity for not accounting for race as an adjustment variable as part of our bias assessment (Supplementary Table 2).
Population characteristics	Data are available in the Supplementary Data
Recruitment	Our paper is a secondary analysis therefore no participants were recruited
Ethics oversight	Our paper is a secondary analysis of published studies, so no ethics approval was required

Note that full information on the approval of the study protocol must also be provided in the manuscript.

Field-specific reporting

Please select the one below that is the best fit for your research. If you are not sure, read the appropriate sections before making your selection.

☒ Life sciences ☐ Behavioural & social sciences ☐ Ecological, evolutionary & environmental sciences

For a reference copy of the document with all sections, see nature.com/documents/nr-reporting-summary-flat.pdf

Life sciences study design

All studies must disclose on these points even when the disclosure is negative.

Sample size	Our sample size was based on whether studies identified in our literature search were eligible for inclusion based on our prespecified inclusion criteria (outlined in the manuscript)
Data exclusions	Studies identified in our search strategy were excluded based on criteria outlined in the Methods and Supplementary Methods
Replication	We assessed consistency of our risk estimates between studies for each cancer type using the IQR, tau-squared and the I-squared (see Methods and Results).
Randomization	Not relevant as a secondary analysis of published studies and adiposity is not an intervention
Blinding	Not relevant as a secondary analysis of published studies and adiposity cannot be blinded

Reporting for specific materials, systems and methods

We require information from authors about some types of materials, experimental systems and methods used in many studies. Here, indicate whether each material, system or method listed is relevant to your study. If you are not sure if a list item applies to your research, read the appropriate section before selecting a response.

Materials & experimental systems

n/a	Involved in the study	
☒	☐	Antibodies
☒	☐	Eukaryotic cell lines
☒	☐	Palaeontology and archaeology
☒	☐	Animals and other organisms
☒	☐	Clinical data
☒	☐	Dual use research of concern
☒	☐	Plants

Methods

n/a	Involved in the study	
☒	☐	ChIP-seq
☒	☐	Flow cytometry
☒	☐	MRI-based neuroimaging

Plants

Seed stocks	N/A
Novel plant genotypes	N/A
Authentication	N/A
